# HCl-mediated silylation of C–H bonds in (hetero)arenes with trialkylsilanes

**DOI:** 10.1039/d5sc06196b

**Published:** 2025-12-16

**Authors:** You Su, Meiqi Zhu, João C. A. Oliveira, Xiangnan Zhang, Zheng-Jun Wang, Lutz Ackermann, Dingyi Wang

**Affiliations:** a College of Sciences, Northeastern University Shenyang 110004 China wangdingyi@mail.neu.edu.cn; b Wöhler-Research Institute for Sustainable Chemistry, Georg-August-Universität Göttingen Göttingen 37077 Germany Lutz.Ackermann@chemie.uni-goettingen.de; c Key Laboratory of Carbon Materials of Zhejiang Province, Institute of New Materials and Industrial Technologies, Wenzhou University Wenzhou Zhejiang 325035 China; d State Key Laboratory of Coordination Chemistry, Nanjing University Nanjing 210023 China; e Key Laboratory of the Ministry of Education for Advanced Catalysis Materials, Zhejiang Normal University Jinhua 321004 China

## Abstract

Direct C–H silylation of (hetero)arenes provides a straightforward route for synthesizing valuable organosilicon molecules. Contrasting with traditional methods that rely on costly photocatalysts, toxic metal reagents, excess strong oxidants, and high temperatures. Herein, our study presents a practical, eco-friendly electron donor–acceptor (EDA) strategy that enables highly efficient C–H silylation of (hetero)arenes by using affordable and readily available HCl as the only additive under visible-light excitation. Substrate classes previously challenging to activate are efficiently silylated with high yields and excellent regioselectivity. Furthermore, this protocol enables the direct silylation of active pharmaceutical ingredients. The resulting heteroarylsilane products undergo versatile transformations, enabling advanced synthetic strategies for heteroaromatic functionalization. This synthetic strategy shows great potential for industrial-scale applications, attributed to readily available raw materials, simple reaction conditions, air and water tolerance, broad substrate scope, and excellent scalability. Notably, this reaction system facilitates other versatile functionalizations of heteroarenes, including trifluoromethylation, acylation, and alkylation. More significantly, the quinoline salt derived from quinoline and hydrochloric acid serves as a novel photocatalyst, converting silanes or alkyl trifluoroborates into corresponding radicals for diverse chemical bond construction. In future studies, these results provide valuable insights for achieving new chemical transformations with quinoline hydrochloride, an EDA complex, as a photocatalyst or additive.

## Abstract 2

Our study presents a practical, eco-friendly electron donor–acceptor (EDA) strategy that enables highly efficient C–H silylation of (hetero)arenes by using affordable and readily available HCl as the only additive under visible-light excitation.

## Graphical abstract

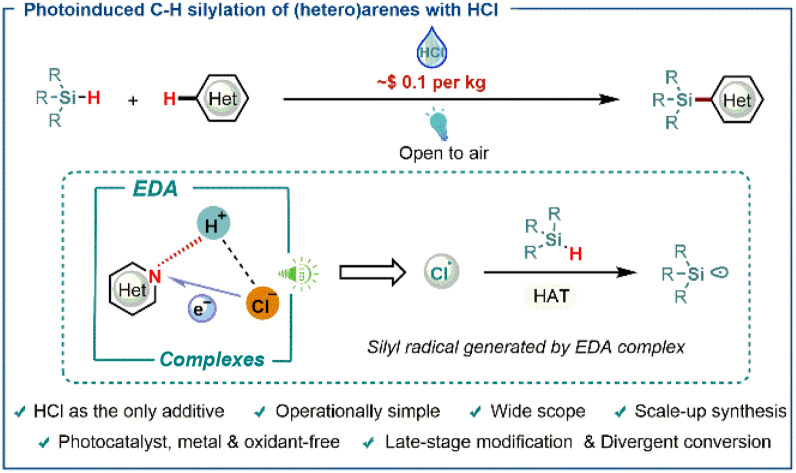

## Introduction

**Scheme 1:**
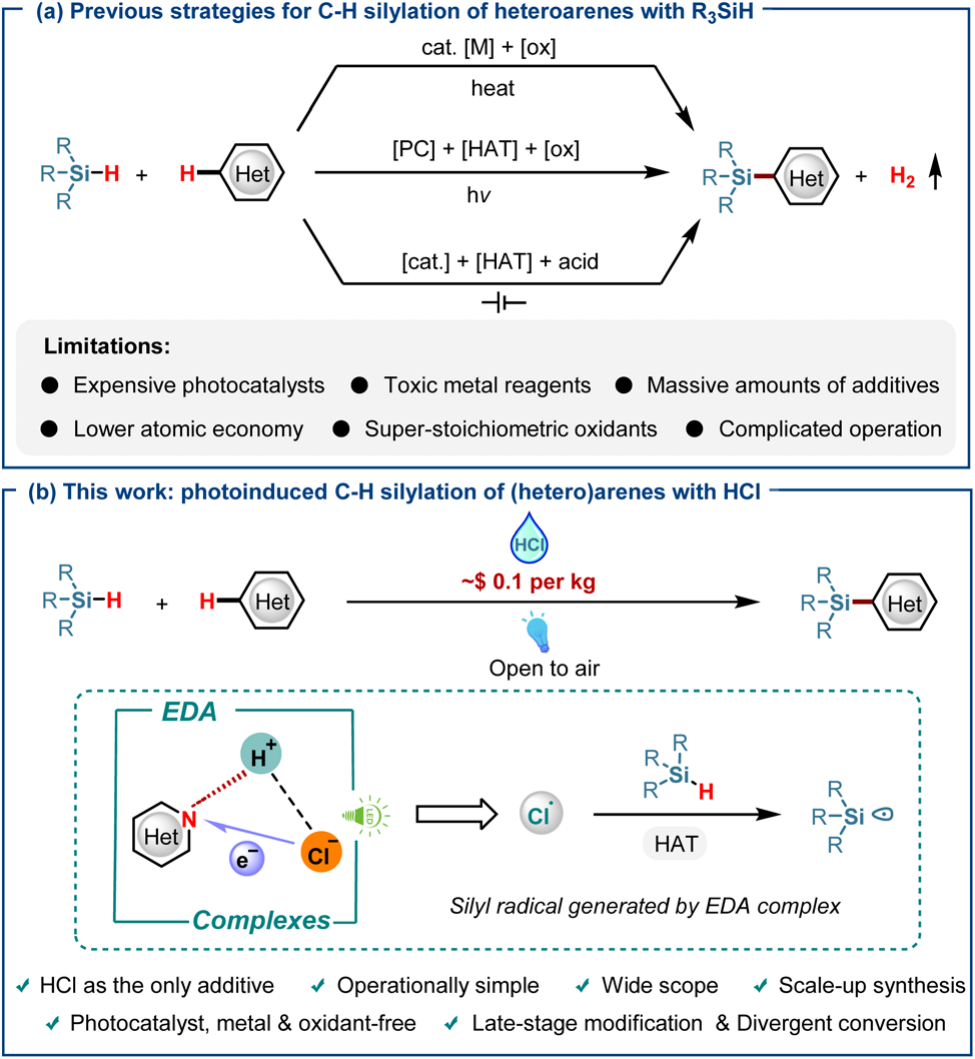
Strategies for the direct C–H silylation of heteroarenes. (a) Previous strategies for C–H silylation of heteroarenes with R_3_SiH. (b) This work: photoinduced C–H silylation of (hetero)arenes with HCl.

Organosilicon compounds, with their distinctive physicochemical properties and reactivity in transformation reactions, are widely applied in materials science, medicinal chemistry, biochemistry, and synthetic chemistry.^1^ Consequently, developing efficient methods for synthesizing organosilicon compounds has emerged as a crucial field of study. Traditional approaches for introducing silyl groups rely on intercepting heteroaryl lithium or magnesium reagents with silicon electrophiles, which suffer from low atom economy and generate significant environmental pollution.^2^ In this context, direct C–H silylation emerges as a compelling alternative, bypassing the need for heteroarene prefunctionalization and enabling the synthesis of valuable heteroaryltrialkylsilanes with various silylated reagents under thermal, electrochemical, and photocatalytic conditions (Scheme 1a).^3–8^ For example, Zhang and co-workers recently introduced an elegant photocatalytic technique for the C–H silylation of heteroarenes using silanes.^9^ Wu and his team have reported the diverse functionalization of hydrosilanes employing neutral eosin Y as the photocatalyst.^10^ Unlike conventional approaches, this strategy offers cost-effective, efficient, and direct routes to C–H silylation, with the incorporation of active functional groups facilitating broader drug screening possibilities, eliminating the need for *de novo* synthesis. However, as a photocatalyst-, metal- and oxidant-free process, the electron donor–acceptor (EDA) strategy has never been reported.

In 2023, the Wang group reported a photoelectrochemical method for the dehydrogenative silylation of heterocycles. The mechanism involves anodic oxidation of chloride to chlorine gas, which undergoes photolysis to generate chlorine radicals that drive the reaction.^11^ This approach provides access to silicon-containing heterocycles in good yields without oxidants or metal catalysts, representing a valuable contribution to synthetic methodology. Nevertheless, the practical utility of this method is constrained by poor atom economy due to large quantities of silylating and HCl reagents. Moreover, the reaction suffers from a substantial loss in yield upon scale-up. These limitations underscore the demand for streamlined, efficient, and general synthetic platforms to access novel silicon-containing heterocyclic scaffolds. Herein, we report a photo-promoted direct silylation of (hetero)arenes that eliminates the need for photo- and metal catalysts, utilizing a novel chlorine radical generation strategy (Scheme 1b). A key feature of this work is the use of inexpensive and readily available HCl as the sole additive, forming an EDA complex with nitrogen-containing heterocycles. This complex undergoes a proton-coupled electron transfer (PCET) process under near-UV irradiation to generate chlorine radicals, enabling highly efficient silylation of (hetero)arenes and scalable transformations of feedstock chemicals. This transformation applies to substrates containing free NH groups, which were previously unsuitable for existing methods.

## Results and discussion

Our investigation began by employing 4-methylquinoline 1a and (*t*-Bu)Me_2_SiH 2a as the template substrates (Table 1). The screening revealed that the C-2 silylation product 3 was obtained in a 73% isolated yield, using only hydrochloric acid as the additive under visible light excitation (entry 1). Examination of other visible light with longer wavelengths such as 390 nm and 420 nm showed lower efficiency (entries 2 and 3). When HCl was replaced with other Brønsted acids such as AcOH or TFA, the yield of the desired product dropped dramatically (entries 4 and 5). Altering solvents, including EA, DMSO, and acetone, reduced the efficiency of this transformation, occasionally yielding no product (entries 6–8). The use of mixed solvents, such as MeCN/acetone (1 : 1) or MeCN/DMSO (1 : 1), failed to enhance reactivity (entries 9 and 10). Finally, the control experiments results show that the HCl and light irradiation were essential to this transformation (entries 11 and 12).

**Table 1:** Reaction development^a^

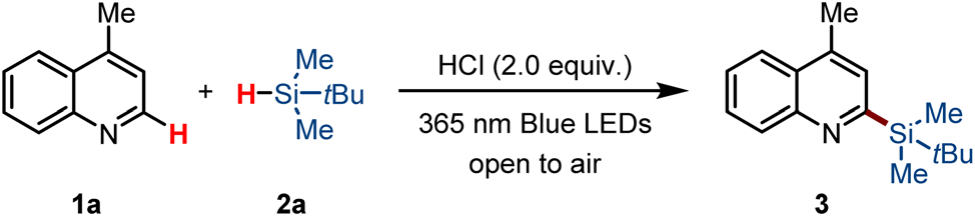		
Entry	Variation from “standard conditions”	Yield of 3^b^ (%)
1	None	76(73)^c^
2	390 nm, 20 W	53
3	420 nm, 20 W	11
4	TFA instead of HCl	51
5	AcOH instead of HCl	55
6	Ethyl acetate instead of MeCN	<5
7	Acetone instead of MeCN	66
8	DMSO instead of MeCN	Trace
9	Acetone : MeCN = 1 : 1	66
10	DMSO : MeCN = 1 : 1	69
11	No light	0
12	No HCl	0

a Reaction conditions: 1a (0.2 mmol), 2a (1.0 mmol) and HCl (0.4 mmol) in MeCN (4.0 mL) at room temperature under irradiation of LEDs (20 W, *λ* = 365 nm) for 12 hours, air.

b Determined by GC.

c Isolated yield after chromatography. TFA = trifluoroacetic acid.

**Scheme 2:**
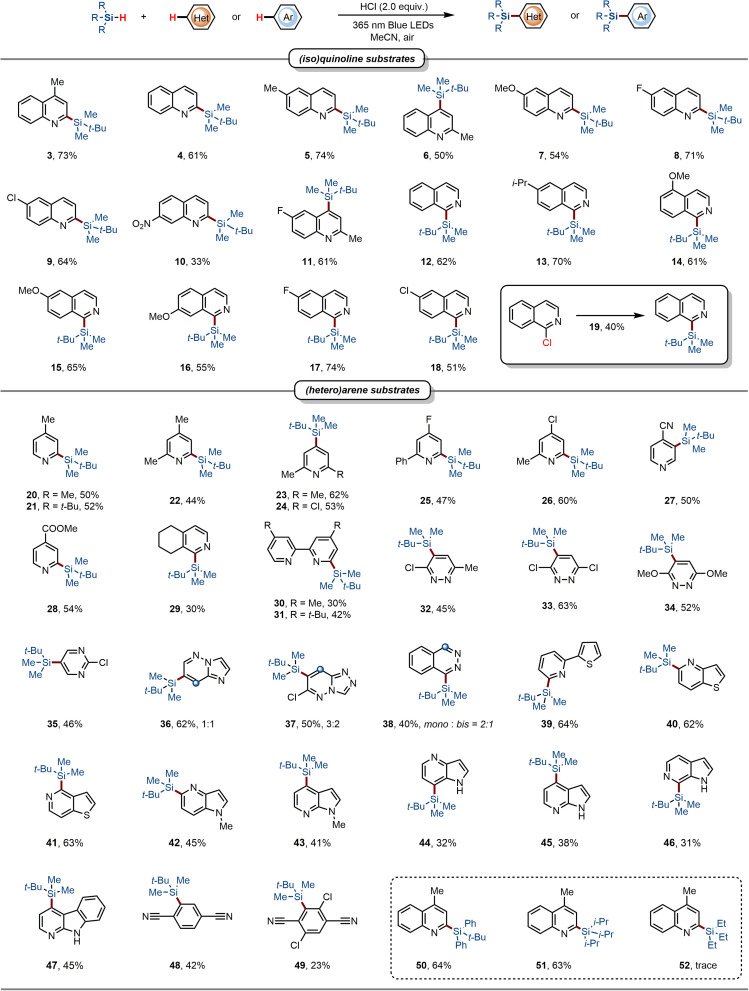
Substrate scope.

After establishing the optimal conditions of the silylation reaction, we explored the process for photoinduced silylation of (hetero)arenes (Scheme 2). We first assessed the generality of quinoline substrates in synthesizing the silylation product. As shown, various quinoline and its derivatives with both electron-withdrawing and electron-donating groups at the different positions, such as C4–Me (3, 73% yield), C6–Me (5, 74% yield), C2–Me (6, 50% yield), C6–MeO (7, 54% yield), C6–F (8, 71% yield), C6–Cl (9, 64% yield), C7–NO_2_ (10, 33% yield) as well as 2,6-disubstituted quinolines also reacted successfully with *tert*-butylsilanes 2a with the assistance of HCl, producing heteroarylsilanes 11 in 61% yields. This approach is quite tolerated with isoquinolines bearing various substituted groups (*e.g.* iPr, OMe, F) at the different positions, yielding a range of silylated isoquinolines (12–17, 55–74% yield). Notably, employing the 6-chloroisoquinoline as a substrate yielded the desired product 18 at 51%, a target traditionally challenging to synthesize *via* conventional quinoline silylation. The dechlorosilylation reaction was observed when 1-chloroisoquinoline was employed as a substrate. Next, we tried to install silyl groups onto other heteroarenes. Pyridines substituted with diverse groups, including Me, *t*-Bu, F, Cl, CN, and COOMe, facilitated the synthesis of a broad array of silylated pyridines (20–29, yields 30–62%). Widely used nitrogen-containing ligands, including 4,4-di-*tert*-butyl bipyridines and 4,4′-dimethyl-2,2′-bipyridyl were suitable for this transformation as well, and modest yields were obtained (30 and 31). Other monocyclic heteroarenes, including 3-chloro-6-methylpyridazines, 3,6-dichloropyridazines, 3,6-dimethoxy-pyridazines, and 2-chloropyrimidines, underwent the reaction smoothly, yielding C–H silylation products (32–35, yields 45–63%). Additionally, various pharmaceutically significant heterocycles such as imidazo[1,2-*b*]pyridazines, 6-chloro-[1,2,4]triazolo[4,3-*b*]pyridaz-ines, and phthalazines reacted smoothly under identical conditions to yield the desired coupling products (36–38, yields 40–62%). However, some substrates exhibited poor regioselectivity, resulting in the formation of two distinct silylation products 38. The reaction scope also extended to pyridine derivatives featuring pyrrole or thiophene backbones (39–43), which provided the product in moderate yields with high regioselectivity, as well as to indoles and carbazoles bearing free NH groups (44–47). Notably, electron-deficient arenes, including 1,4-dicyanobenzenes and 2,5-dichloroterephthalonitriles, were effective substrates, delivering the corresponding products (48 and 49) with satisfactory yields. Furthermore, other silylation reagents such as triisopropylsilanes and di-*tert*-butylsilanes reacted effectively with 4-methylquinolines 1a to yield the corresponding C–H silylation products (50 and 51). The light-mediated approach proves incompatible with smaller trialkylsilanes, such as triethylsilanes. This result might also be attributed to the instability of triethylsilyl radicals in the presence of the strong acid.

**Scheme 3:**
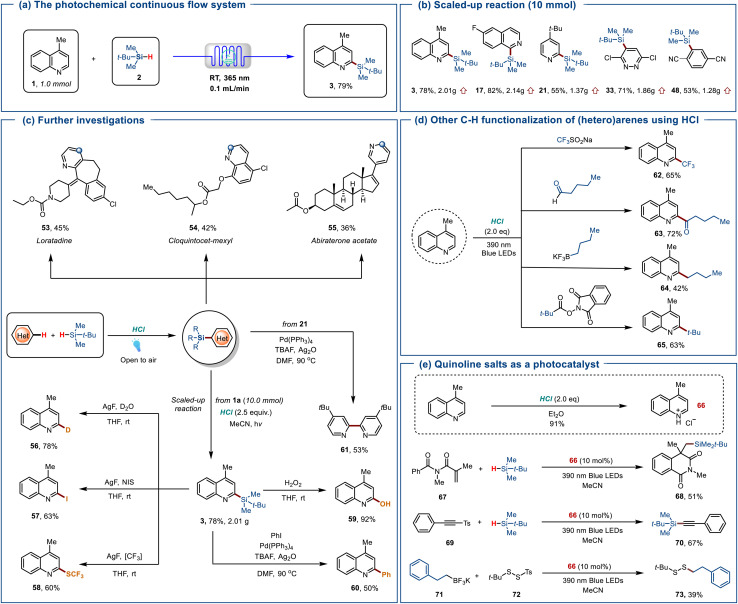
Further investigations. (a) The photochemical continuous flow system. (b) Scaled-up reaction (10 mmol). (c) Further investigations. (d) Other C–H functionalization of (hetero)arenes using HCl. (e) Quinoline salts as a photocatalyst.

A photochemical continuous flow technique was successfully implemented to demonstrate the practical utility of this strategy for constructing C–Si bonds (Scheme 3a). The reactants were dissolved in MeCN and introduced into the microchannel reactor *via* an injection pump. Compared to the batch reaction, the continuous flow reaction yielded C–H silylation 3 with excellent efficiency at a flow rate of 0.1 mL min^−1^, increasing the reaction yield from 73% to 79%. Then we conducted gram-scale silylation of various heterocycles (Scheme 3b). Surprisingly, scaling up the reaction increased the yield of target products compared to the small-scale reaction. This finding further indicates that the developed method holds significant potential for industrial applications. Moreover, our method enables the regioselective silylation of various natural products and drugs, including loratadine, cloquintocet-mexyl, and abiraterone acetate (53–55) (Scheme 3c). Subsequently, downstream transformations, detailed in Scheme 3d, were explored to demonstrate the practical utility of this protocol. The trialkylsilyl group at the C-2 position of 4-methylquinolines was efficiently converted to D, I, and SCF_3_, achieving 60–78% yields when the silylated product 3 was treated with silver fluoride (AgF). In addition to the nucleophilic substitution reaction, C-2 Si-directed Fleming–Tamao oxidation could be achieved to afford the 4-methylquinolin-2-ol 59 by employing H_2_O_2_ as an oxidizing reagent. In addition, the 2-arylquinoline 60 and bipyridine ligand 61 were synthesized *via* Pd-catalyzed Hiyama–Denmark cross-coupling reaction.

To demonstrate the broad applicability of this synthetic strategy, we extended the reaction system to other functionalization reactions of heterocycles (Scheme 3d). Trifluoromethylation, acylation, and alkylation of quinoline at the C2 position were achieved using sodium trifluoromethylbenzenesulfinate, diphenylphosphine oxide, aliphatic aldehydes, and *tert*-butyl *N*-(acyloxy)-phthalimides as radical precursors, respectively (62–65). Furthermore, regioselective alkylation of quinolines was accomplished using primary alkyl trifluoroborates, a transformation unattainable by conventional methods. Remarkably, further research revealed that readily available quinoline hydrochloride 66 itself acts as a photocatalysts or initiator, facilitating the generation of free radicals from alkyl trifluoroborate esters or silanes and enabling the construction of diverse chemical bonds (Scheme 3e). For instance, employing quinoline hydrochloride as a catalyst and unsaturated benzamides or ethynyl phenyl sulfones (EPSs) as substrates, lactams (68) and alkynyl silanes (70) were obtained in moderate yields under visible light irradiation. And the silylated quinoline adduct 3 was obtained in these transformation. Using alkyl trifluoroborate as the alkylating reagent and acyl sulfur as the radical acceptor under the above conditions yields thioether analogues (73). Furthermore, while catalytic silylation of quinoline with trialkylsilanes was achieved using quinoline hydrochloride, the reaction efficiency was substantially lower than that under stoichiometric HCl conditions. Based on further investigation (please see the SI), we propose that this catalytic pathway is hampered by several factors: the low concentration and instability of chlorine radicals, steric encumbrance from the silane, and the differential reactivity of the acceptors. These results provide valuable insights for achieving new chemical transformations with quinoline hydrochloride as an initiator, a catalyst or substrate in future studies.

**Scheme 4:**
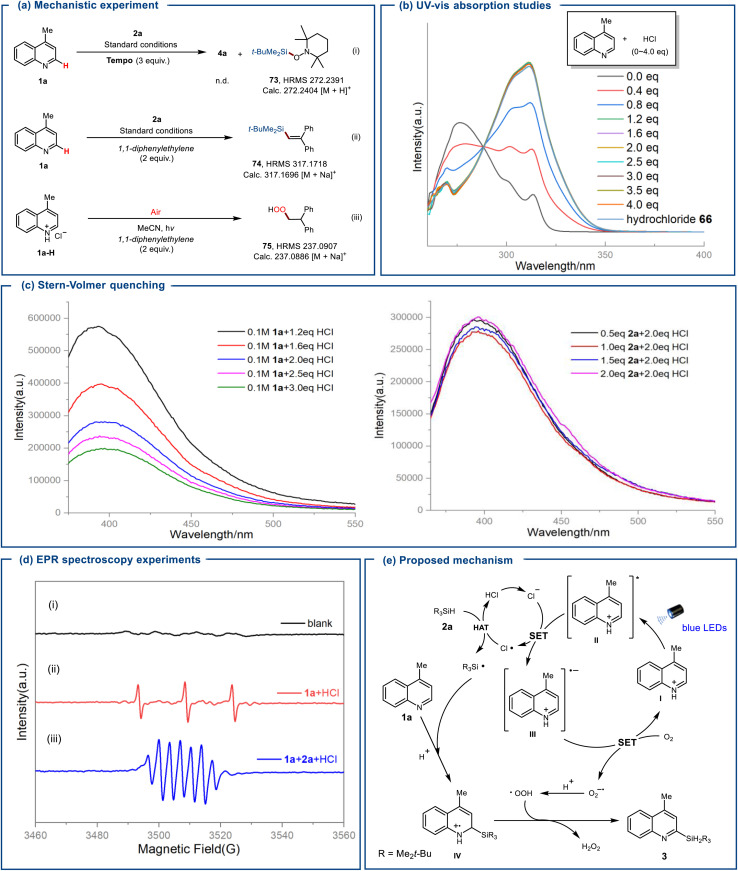
Mechanistic investigations. (a) Mechanistic experiment. (b) UV-vis absorption studies. (c) Stern–Volmer quenching. (d) EPR spectroscopy experiments. (e) Proposed mechanism.

We conducted mechanistic studies to elucidate the coupling reaction pathway, as illustrated in Scheme 4a. The addition of TEMPO (3.0 equiv.) significantly inhibited these transformations, preventing the observation of the corresponding products. Instead, the *tert*-butyldimethylsilane-addition product 73 was detected by HRMS, which verifies the formation of silyl radicals during this transformation. Upon adding 1,1-diphenylethylene 74 to the system when hydrochlorides 1a-H were selected as a substrate instead of 4-methylquinoline 1a, HRMS detected the add-on product 75, indicating the generation of HOO˙ radicals. Moreover, we carried out spectroscopic experiments to further investigate the reaction mechanism. In the UV-Vis absorption spectrum experiments, adding hydrochloric acid to 4-methylquinolines 1a resulted in a noticeable bathochromic shift and a new absorption peak at 312 nm (Scheme 4b). This result suggests the possible formation of an EDA complex between the nitrogen-containing heterocycles and hydrochloric acid. Stern–Volmer quenching studies revealed that excited 1a-H fluorescence was quenched more effectively by HCl than by trialkylsilane 2a (Scheme 4c). These findings indicate that excited 1a-H activates HCl to produce chlorine radicals through a SET mechanism, rather than directly generating silyl radicals from silanes. When irradiating a mixture of quinoline 1a, HCl, and 5,5-dimethyl-1-pyrroline *N*-oxide (DMPO) with blue LEDs (*λ* = 365 nm), the production of ^1^O_2_ was observed (Scheme 4d, ii). When trialkylsilane 2a was added to a mixture of 1a, HCl, and DMPO, a silyl radical and ^1^O_2_ simultaneously appeared (Scheme 4d, iii).^13^

Based on our investigation and prior research, we propose a plausible mechanism for the HCl-mediated silylation of (hetero)arenes, as depicted in Scheme 4e.^3,4,11–15^ Initially, upon irradiation, protonated 4-methylquinoline I transitions to the singlet state II and undergoes electron transfer with Cl^−^, forming intermediate III and Cl˙. The chlorine radical then abstracts a hydrogen atom from trialkylsilane 2a, producing silyl radicals and HCl. These silyl radicals then undergo intermolecular radical addition to I, forming an aminyl radical cation intermediate IV. This intermediate facilitates an oxidative aromatization process catalyzed by HOO˙, leading to deprotonation and the formation of the target silylated product 3, with *in situ* release of H_2_O_2_. Moreover, we propose plausible reaction pathways for the trifluoromethylation, acylation, and alkylation reactions (please see the SI). Specifically, sodium trifluoromethanesulfonates, alkyl boronic esters, and alkyl esters each undergo electron transfer with the excited state of protonated quinoline II to generate the corresponding radical species. Importantly, generated chlorine radicals are essential for generating the corresponding radical species, contributing to this process. The subsequent functionalization steps follow a mechanism analogous to that of quinoline silylation.

## Conclusions

In summary, detailed mechanistic studies and theoretical calculations revealed a novel pathway for generating chlorine radicals *via* an EDA complex formed by HCl and nitrogen-containing heterocycles. The generated chlorine radicals activate trialkylsilanes through the HAT process, which subsequently couples with various (hetero)arenes. This strategy has been successfully applied to diverse privileged heteroaromatic scaffolds, aromatic moieties bearing electron-withdrawing groups, and unsaturated benzamides. The method enables the synthesis of diverse heteroarylsilanes under ambient air conditions using abundant, stable, inexpensive, and commercially available hydrochloric acid as the sole additive, offering promising potential for industrial-scale applications.

## Author contributions

D. W., and L. A. conceived the project. Y. S., Z.-J. W. X. Z. and M. Z. conducted the experiments. J. C. A. O. conducted the DFT studies. D. W. wrote the manuscript. All authors discussed the results.

## Conflicts of interest

The authors declare no competing financial interests.

## Supplementary Material

SC-OLF-D5SC06196B-s001

## Data Availability

The raw data are available in the source data file. Source data are provided in this paper. All data generated in this study are provided in the main text or the supplementary information (SI). Supplementary information is available. See DOI: https://doi.org/10.1039/d5sc06196b.
