# Supplementary material for: HCl-mediated silylation of C–H bonds in (hetero)arenes with trialkylsilanes
Source: Chem Sci. 2025 Dec 16;17(6):3084–91. doi: 10.1039/d5sc06196b (PMC12721400; doi:10.1039/d5sc06196b)
Supplement: SC-017-D5SC06196B-s001 [file SC-017-D5SC06196B-s001.pdf]

Supplementary Information (SI) for Chemical Science.
This journal is © The Royal Society of Chemistry 2025

Supplementary Information for

HCl-mediated Silylation of C-H Bonds in (Hetero)arenes with Trialkylsilanes

You Su,[a]† Meiqi Zhu,[a]† João C. A. Oliveira,[b] Xiangnan Zhang,[a] Zheng-Jun Wang,[c] Lutz Ackermann[b,]* and Dingyi Wang[a,d,e]*

[a]College of Sciences, Institute of Dynamics and Smart Disaster Prevention, Northeastern University, Shenyang 110004, China

[b]Wöhler-Research Institute for Sustainable Chemistry, Georg-August-Universität Göttingen, Göttingen 37077, Germany

[c]Key Laboratory of Carbon Materials of Zhejiang Province, Institute of New Materials and Industrial Technologies, Wenzhou University, Wenzhou, Zhejiang 325035, China

[d]State Key Laboratory of Coordination Chemistry, Nanjing University, Nanjing 210023, China

[e]Key Laboratory of the Ministry of Education for Advanced Catalysis Materials, Zhejiang Normal University, Jinhua 321004, China

†These authors contributed equally: You Su, Meiqi Zhu

*E-mail: wangdingyi@mail.neu.edu.cn; Lutz.Ackermann@chemie.uni-goettingen.de

TABLE OF CONTENTS

1. General Experimental Details.......... 3

2. Table of Reaction Optimization 4

3. Substrates preparation 6

4. General experimental procedure.......... 6

5. Characterization data for compounds.......... 6

6. Mechanism study.......... 26

7. Synthetic Transformations of Products 36

8. DFT calculations 42

9. NMR Spectra.......... 56

10. References 122

1. General Experimental Details

Photocatalytic reactions were performed in 10 mL Schlenk tubes at the indicated temperature under irradiation with a 20 W blue LEDs lamp (λmax = 365 nm; commercial supplier: Fa Cai led Lighting Co. Ltd.). All reagents, unless otherwise stated, were directly used as received from commercial suppliers (Alfa, Tci, Innochem, Aladdin, Energy Chemical, etc.) without further purification. Commercial solvents were used without further purification with the following exceptions: tetrahydrofuran and dichloromethane were dried using an Innovative Technology PURE SOLV solvent purification system. All new compounds were fully characterized. NMR-spectra were recorded on Bruker ARX-400 MHz. ^{1}H NMR spectra data were reported as δ values in ppm relative to chloroform (δ 7.26) if collected in $CDCl_3$. ^{13}C NMR spectra data were reported as δ values in ppm relative to chloroform (δ 77.00) if collected in $CDCl_3$. ^{1}H NMR coupling constants were reported in Hz, and multiplicity was indicated as follows: s (singlet); d (doublet); t (triplet); q (quartet); quint (quintet); m (multiplet); dd (doublet of doublets); ddd (doublet of doublet of doublets); dddd (doublet of doublet of doublet of doublets); dt (doublet of triplets); td (triplet of doublets); ddt (doublet of doublet of triplets); dq (doublet of quartets); app (apparent); br (broad). Mass spectra were conducted at Micromass Q-Tof instrument (ESI) and Agilent Technologies 5973N (EI).

2. Table of Reaction Optimization

Table S1. Screening of the wavelength

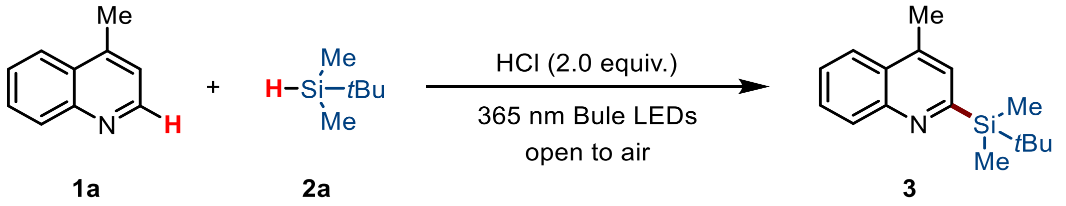

Entry	Wavelength (nm)	Yield (%)[b]
1	450	<5
2	420	11
3	390	53
4	365	76 (73)[c]

Reaction conditions: **1a** (0.2 mmol), **2a** (1.0 mmol), and HCl (0.4 mmol) in MeCN (4 mL) at room temperature under irradiation of blue LEDs (20 W) for 12 hours, air. [b]Determined by GC. [c]Isolated yield after chromatography.

Table S2. Screening of the acid

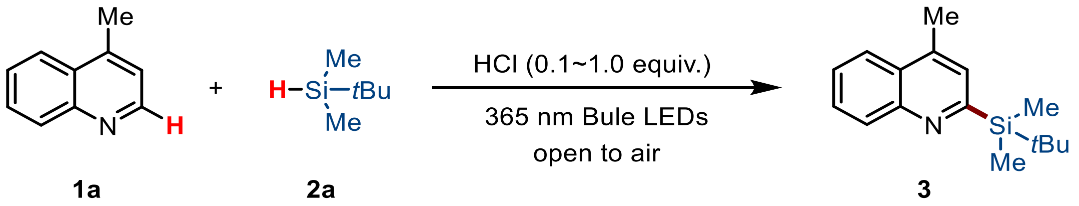

Entry	HCl (mol%)	Time (h)	Yield (%)[b]
1	0.1	12	12
2	0.1	24	23
3	0.1	36	24
4	0.5	12	35
5	0.5	24	61
6	0.5	36	60
7	1.0	12	57

8	1.0	24	62
9	1.0	36	62

Reaction conditions: **1a** (0.2 mmol), **2a** (1.0 mmol), and acid in MeCN (4 mL) at room temperature under irradiation of LEDs (20 W, λ = 365 nm) for 12 hours, air. [b]Determined by GC. [c]Isolated yield after chromatography.

The results confirm that catalytic turnover for the silylation of quinoline is achievable with quinoline hydrochloride upon extended reaction time, although the yield is substantially lower than that with stoichiometric HCl. We propose that this catalytic pathway is hampered by several factors: low concentration and unstable of chlorine radicals, steric shielding from the silane, and differential reactivity of the acceptors. Furthermore, the reaction system involves a dynamic equilibrium between hydrochloric acid, quinoline salts, and the silylated quinoline products.

Table S3 Screening of the solvent

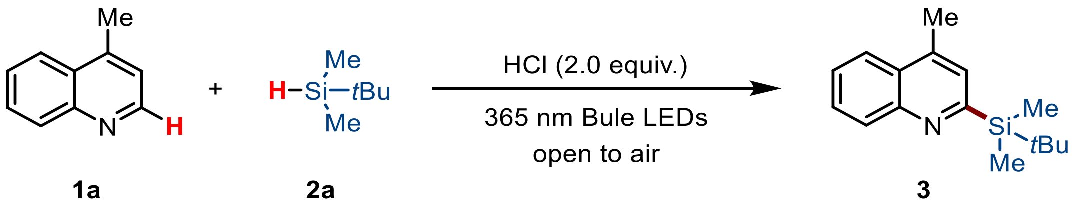

Entry	Solvent (mL)	Yield (%)[b]
1	MeCN	76 (73)[c]
2	Acetone	66
3	EA	<5
4	MeCN:acetone = 1:1	66
5	MeCN:DMSO = 1:1	69

Reaction conditions: **1a** (0.2 mmol), **2a** (1.0 mmol) and HCl (0.4 mmol) in solvent (4 mL) at room temperature under irradiation of LEDs (20 W, λ = 365 nm) for 12 hours, air. [b]Determined by GC. [c]Isolated yield after chromatography.

3. Substrates preparation

General procedure for the synthesis of N-methacryloyl-N-methylbenzamide

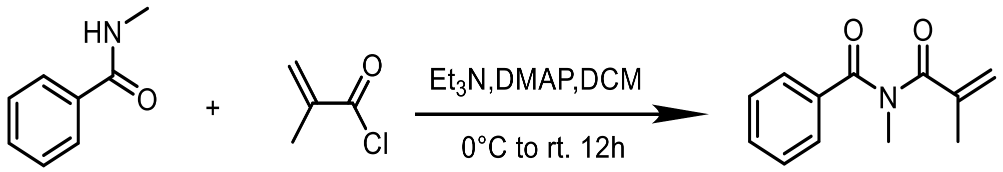

Charge a dried round-bottom flask with a magnetic stirring bar, substituted benzamides (5 mmol), triethylamines (10 mmol), DMAP (0.5 mmol) and DCM (30 mL). Add methacryloyl chloride (10 mmol) dissolved in DCM (10 mL) slowly to the mixture for 5 minutes at 0 °C. Stir the mixture for 12 hours at room temperature. Monitor the reaction by TLC. After completion, quench the reaction with saturated NaCl solution (100 mL). Extract the mixture with DCM (30 mL). Dry the organic phase over anhydrous Na_2SO_4. Concentrate the organic phase under reduced pressure. Purify the reaction mixture *via* column chromatography.

4. General experimental procedure

A 10 mL tube equipped with a magnetic stir bar was charged with (hetero)arenes (0.2 mmol, 1 equiv.), trialkylsilanes (1.0 mmol, 5.0 equiv.), HCl (12 mol/L, 33 μL, 0.4 mmol, 2 equiv.), and 4 mL of CH_3CN. The mixture was irradiated by blue LEDs (20W, λ = 365 nm) for 12 h in the air at room temperature. After reaction, the mixture was diluted with 1.0 mL of aqueous 1 M NaOH solution and extracted with ethyl acetate. Then the organic phase was combined and washed with brine and dried over anhydrous sodium sulfate. Upon removal of solvent under vacuum, the residue was purified by chromatography on silica gel (petroleum ether/ethyl acetate = 100:1–5:1) to give the desired product.

5. Characterization data for compounds

2-(tert-butyldimethylsilyl)-4-methylquinoline (3)[1]

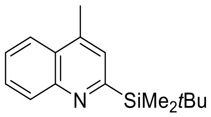

According to the general procedure, using 4-methylquinoline (28.6 mg, 0.2 mmol), tert-butyldimethylsilane (116.1 mg, 1.0 mmol), HCl (12 mol/L, 33 μL, 0.4 mmol) in MeCN (4 mL), and was purified by silica gel column chromatography (petroleum ether/ethyl acetate = 20:1) to obtain **3** as colorless oil (37.5 mg, 73% yield). **^{1}H NMR (400 MHz, $CDCl_3$) δ** 8.17 (d, *J* = 8.1 Hz, 1H), 7.97 (d, *J* = 8.3 Hz, 1H), 7.70 – 7.65 (m, 1H), 7.56 – 7.50 (m, 1H), 7.41 (s, 1H), 2.73 – 2.66 (m, 3H), 0.99 (s, 9H), 0.41 (s, 6H). **^{13}C NMR (101 MHz, $CDCl_3$) δ** 168.5, 148.4, 140.2, 130.8, 128.4, 127.3, 126.0, 123.6, 123.5, 26.7, 18.6, 17.2, -6.1.

2-(tert-butyldimethylsilyl)quinoline (4)[1]

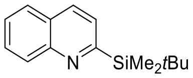

According to the general procedure, using quinoline (25.8 mg, 0.2 mmol), tert-butyldimethylsilane (116.1 mg, 1.0 mmol), HCl (12 mol/L, 33 μL, 0.4 mmol) in MeCN (4 mL), and was purified by silica gel column chromatography (petroleum ether/ethyl acetate = 20:1) to obtain **4** as colorless oil (29.6 mg, 61% yield). **^{1}H NMR (400 MHz, $CDCl_3$) δ** 8.22 – 8.12 (m, 1H), 8.06 – 7.99 (m, 1H), 7.77 (d, *J* = 8.1 Hz, 1H), 7.72 – 7.65 (m, 1H), 7.59 (d, *J* = 8.3 Hz, 1H), 7.51 (t, *J* = 7.4 Hz, 1H), 0.97 (s, 9H), 0.42 (s, 6H). **^{13}C NMR (101 MHz, $CDCl_3$) δ** 169.2, 149.2, 132.7, 130.2, 127.6, 127.2, 126.3, 126.1, 26.7, 17.2, -6.1.

2-(tert-butyldimethylsilyl)-6-methylquinoline (5)[2]

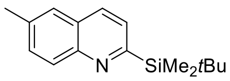

According to the general procedure, using 6-methylquinoline (28.6 mg, 0.2 mmol), tert-butyldimethylsilane (116.1 mg, 1.0 mmol), HCl (12 mol/L, 33 μL, 0.4 mmol) in MeCN (4 mL), and was purified by silica gel column chromatography (petroleum ether/ethyl acetate = 20:1) to obtain **5** as colorless oil (38.0 mg, 74% yield). **^{1}H NMR (400 MHz, $CDCl_3$) δ** 8.17 – 8.03 (m, 1H), 8.01 – 7.87 (m, 1H), 7.60 – 7.42 (m, 3H), 2.53 (s, 3H), 0.96 (s, 9H), 0.41 (s, 6H). **^{13}C NMR (101 MHz, $CDCl_3$) δ** 135.2, 132.0, 131.2, 131.1, 129.9, 129.0, 126.4, 126.1, 118.2, 26.7, 21.6, 17.2, -6.2.

4-(tert-butyldimethylsilyl)-2-methylquinoline (6)[3]

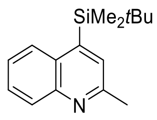

According to the general procedure, using 2-methylquinoline (28.6 mg, 0.2 mmol), tert-butyldimethylsilane (116.1 mg, 1.0 mmol), HCl (12 mol/L, 33 μL, 0.4 mmol) in MeCN (4 mL), and was purified by silica gel column chromatography (petroleum ether/ethyl acetate = 50:1) to obtain **6** as colorless solid (22 mg, 40% yield). **^{1}H NMR (400 MHz, CDCl$_3$) δ** 8.22 (s, 1H), 7.99 (d, *J* = 8.5 Hz, 1H), 7.77 (d, *J* = 8.1 Hz, 1H), 7.72 – 7.63 (m, 1H), 7.48 (t, *J* = 7.9 Hz, 1H), 2.84 (s, 3H), 0.94 (s, 9H), 0.45 (s, 6H). **^{13}C NMR (101 MHz, CDCl$_3$) δ** 157.1, 147.4, 146.9, 130.6, 130.3, 129.5, 128.9, 128.6, 125.0, 27.1, 25.3, 17.7, -3.3.

2-(tert-butyldimethylsilyl)-6-methoxyquinoline (7)[4]

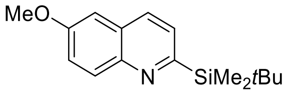

According to the general procedure, using 6-methoxyquinoline (31.8 mg, 0.2 mmol), tert-butyldimethylsilane (116.1 mg, 1.0 mmol), HCl (12 mol/L, 33 μL, 0.4 mmol) in MeCN (4 mL), and was purified by silica gel column chromatography (petroleum ether/ethyl acetate = 20:1) to obtain **7** as colorless oil (29.4 mg, 54% yield). **^{1}H NMR (400 MHz, CDCl$_3$) δ** 8.05 (d, *J* = 9.2 Hz, 1H), 7.91 (d, *J* = 8.2 Hz, 1H), 7.53 (d, *J* = 8.2 Hz, 1H), 7.37 – 7.29 (m, 1H), 7.02 (d, *J* = 2.8 Hz, 1H), 3.93 (s, 3H), 0.95 (s, 9H), 0.39 (s, 6H). **^{13}C NMR (101 MHz, CDCl$_3$) δ** 175.9, 131.7, 131.5, 126.5, 121.6, 104.8, 55.5, 26.7, 17.2, -6.2.

2-(tert-butyldimethylsilyl)-6-fluoroquinoline (8)[5]

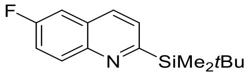

According to the general procedure, using 6-fluoroquinoline (29.4 mg, 0.2 mmol), tert-butyldimethylsilane (116.1 mg, 1.0 mmol), HCl (12 mol/L, 33 μL, 0.4 mmol) in MeCN (4 mL), and was purified by silica gel column chromatography (petroleum ether/ethyl acetate = 20:1) to obtain **8** as colorless oil (37.0 mg, 71% yield). **^{1}H NMR (400 MHz, CDCl$_3$) δ** 8.69 (d, *J* = 5.7 Hz, 1H), 8.31 – 8.23 (m, 1H), 7.52 (d, *J* = 5.7 Hz, 1H), 7.43 – 7.36 (m, 1H), 7.36 – 7.28 (m, 1H), 0.95 (s, 9H), 0.55 (s, 6H). **^{13}C NMR (101 MHz, CDCl$_3$)** *δ* 170.2, 162.2 (d, *J* = 251.7 Hz), 143.1, 136.2 (d, *J* = 10.1 Hz), 131.6 (d, *J* = 9.4 Hz), 131.3, 119.5 (d, *J* = 5.2 Hz), 116.8 (d, *J* = 25.1 Hz), 110.5 (d, *J*

= 20.2 Hz), 27.0, 17.9, -3.1. **^{19}F NMR (376 MHz, $CDCl_3$) δ** -109.05.

2-(tert-butyldimethylsilyl)-6-chloroquinoline (9)[5]

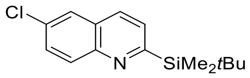

According to the general procedure, using 6-chloroquinoline (32.6 mg, 0.2 mmol), tert-butyldimethylsilane (116.1 mg, 1.0 mmol), HCl (12 mol/L, 33 μL, 0.4 mmol) in MeCN (4 mL), and was purified by silica gel column chromatography (petroleum ether/ethyl acetate = 20:1) to obtain **9** as colorless oil (35.4 mg, 64% yield). **^{1}H NMR (400 MHz, $CDCl_3$)** δ 8.23 – 8.13 (m, 2H), 7.78 – 7.68 (m, 1H), 7.65 – 7.57 (m, 2H), 0.97 (s, 9H), 0.40 (s, 6H). **^{13}C NMR (101 MHz, $CDCl_3$)** δ 134.9, 131.9, 131.0, 129.8, 129.7, 126.8, 126.2, 125.9, 119.1, 26.6, 17.2, -6.2.

2-(tert-butyldimethylsilyl)-7-nitroquinoline (10)

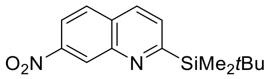

According to the general procedure, using 2-methyl-7-nitroquinoline (37.6 mg, 0.2 mmol), tert-butyldimethylsilane (116.1 mg, 1.0 mmol), HCl (12 mol/L, 33 μL, 0.4 mmol) in MeCN (4 mL), and was purified by silica gel column chromatography (petroleum ether/ethyl acetate = 30:1) to obtain **10** as colorless oil (19 mg, 33% yield). **^{1}H NMR (400 MHz, $CDCl_3$)** δ 9.09 (s, 1H), 8.32 – 8.26 (m, 1H), 8.12 (d, J = 8.3 Hz, 1H), 7.92 (d, J = 9.0 Hz, 1H), 7.74 (d, J = 8.3 Hz, 1H), 0.97 (s, 9H), 0.43 (s, 6H). **^{13}C NMR (101 MHz, $CDCl_3$)** δ 132.4, 129.3, 128.5, 126.6, 119.7, 26.6, 17.2, -6.3. **HR-MS** (ESI) $C_{15}H_{21}N_2O_2Si^+$ $(M+H)^+$ 289.1367, found 289.1366.

4-(tert-butyldimethylsilyl)-6-fluoro-2-methylquinoline (11)[1]

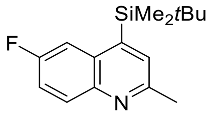

According to the general procedure, using 6-fluoro-2-methylquinoline (32.2 mg, 0.2 mmol), tert-butyldimethylsilane (116.1 mg, 1.0 mmol), HCl (12 mol/L, 33 μL, 0.4 mmol) in MeCN (4 mL), and was purified by silica gel column chromatography (petroleum ether/ethyl acetate = 20:1) to obtain **11** as colorless oil (33.5 mg, 61% yield). **^{1}H NMR (500 MHz, $CDCl_3$)** δ 8.06 – 7.97 (m, 1H), 7.67 – 7.60 (m, 1H), 7.45 – 7.38 (m, 2H), 2.72 (s, 3H), 0.94 (s, 9H), 0.51 (s, 6H). **^{13}C NMR (126 MHz, $CDCl_3$)** δ 169.6, 159.5 (d, J = 245.4 Hz), 156.5 (d, J = 2.7 Hz), 146.2, 144.6, 131.7 (d, J = 9.2 Hz), 130.9, 118.5 (d, J = 25.3 Hz), 112.2 (d, J = 21.9 Hz), 27.0, 25.2, 17.5, -3.5. **^{19}F NMR (376 MHz,**

$CDCl_3$) δ -114.78.

1-(tert-butyldimethylsilyl)isoquinoline (12)[1]

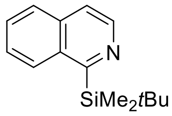

According to the general procedure, using isoquinoline (25.8 mg, 0.2 mmol), tert-butyldimethylsilane (116.1 mg, 1.0 mmol), HCl (12 mol/L, 33 μL, 0.4 mmol) in MeCN (4 mL), and was purified by silica gel column chromatography (petroleum ether/ethyl acetate = 50:1) to obtain **12** as colorless oil (30.1 mg, 62% yield). **^{1}H NMR (400 MHz, $CDCl_3$)** δ 8.71 (d, J = 5.6 Hz, 1H), 8.25 (d, J = 8.5 Hz, 1H), 7.80 (d, J = 8.1 Hz, 1H), 7.67 – 7.61 (m, 1H), 7.59 – 7.53 (m, 2H), 0.97 (s, 9H), 0.56 (s, 6H). **^{13}C NMR (101 MHz, $CDCl_3$) δ** 170.2, 142.5, 134.5, 134.0, 129.1, 128.6, 127.6, 126.3, 119.8, 27.1, 18.0, -3.0.

1-(tert-butyldimethylsilyl)-6-isopropylisoquinoline (13)

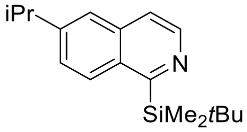

According to the general procedure, using 6-isopropylisoquinoline (34.2 mg, 0.2 mmol), tert-butyldimethylsilane (116.1 mg, 1.0 mmol), HCl (12 mol/L, 33 μL, 0.4 mmol) in MeCN (4 mL), and was purified by silica gel column chromatography (petroleum ether/ethyl acetate = 30:1) to obtain **13** as colorless oil (39.9 mg, 70% yield). **^{1}H NMR (400 MHz, $CDCl_3$) δ** 8.66 (d, J = 5.6 Hz, 1H), 8.17 (d, J = 8.7 Hz, 1H), 7.60 – 7.57 (m, 1H), 7.51 (d, J = 5.6 Hz, 1H), 7.48 – 7.45 (m, 1H), 3.16 – 3.02 (m, 1H), 1.36 (s, 3H), 1.34 (s, 3H), 0.97 (s, 9H), 0.54 (s, 6H). **^{13}C NMR (101 MHz, $CDCl_3$) δ** 169.5, 142.6, 128.6, 127.3, 126.3, 125.5, 124.3, 123.6, 119.8, 34.3, 27.1, 23.6, 18.0, -3.1. **HR-MS** (ESI) $C_{18}H_{28}NSi^+$ $(M+H)^+$ 286.1986, found 286.1990.

1-(tert-butyldimethylsilyl)-5-methoxyisoquinoline (14)[1]

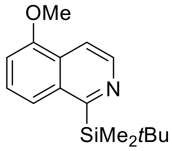

According to the general procedure, using 5-methoxyisoquinoline (31.8 mg, 0.2 mmol), tert-butyldimethylsilane (116.1 mg, 1.0 mmol), HCl (12 mol/L, 33 μL, 0.4 mmol) in MeCN (4 mL), and

was purified by silica gel column chromatography (petroleum ether/ethyl acetate = 10:1) to obtain **14** as colorless oil (33.3 mg, 61% yield). **^{1}H NMR (400 MHz, CDCl$_3$) δ** 8.77 – 8.68 (m, 1H), 8.00 – 7.94 (m, 1H), 7.81 (d, *J* = 8.5 Hz, 1H), 7.45 (t, *J* = 8.1 Hz, 1H), 6.95 (d, *J* = 7.7 Hz, 1H), 4.00 (s, 3H), 0.96 (s, 9H), 0.55 (s, 6H). **^{13}C NMR (101 MHz, CDCl$_3$) δ** 154.9, 142.3, 134.6, 127.4, 127.2, 126.2, 120.6, 113.9, 106.5, 55.6, 27.1, 18.0, -3.0.

1-(tert-butyldimethylsilyl)-6-methoxyisoquinoline (15)[1]

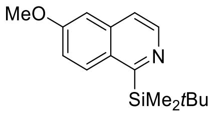

According to the general procedure, using 6-methoxyisoquinoline (31.8 mg, 0.2 mmol), tert-butyldimethylsilane (116.1 mg, 1.0 mmol), HCl (12 mol/L, 33 μL, 0.4 mmol) in MeCN (4 mL), and was purified by silica gel column chromatography (petroleum ether/ethyl acetate = 10:1) to obtain **15** as colorless oil (35.5 mg, 65% yield). **^{1}H NMR (400 MHz, CDCl$_3$) δ** 8.62 (d, *J* = 5.6 Hz, 1H), 8.14 (d, *J* = 9.2 Hz, 1H), 7.46 (d, *J* = 5.6 Hz, 1H), 7.22 – 7.15 (m, 1H), 7.03 (s, 1H), 3.94 (s, 3H), 0.95 (s, 9H), 0.54 (s, 6H). **^{13}C NMR (101 MHz, CDCl$_3$) δ** 169.6, 169.0, 159.7, 143.0, 136.6, 130.4, 119.3, 119.2, 104.8, 55.4, 27.1, 18.0, -3.0.

1-(tert-butyldimethylsilyl)-7-methoxyisoquinoline (16)[1]

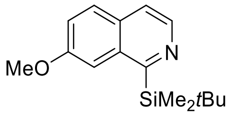

According to the general procedure, using 7-methoxyisoquinoline (31.8 mg, 0.2 mmol), tert-butyldimethylsilane (116.1 mg, 1.0 mmol), HCl (12 mol/L, 33 μL, 0.4 mmol) in MeCN (4 mL), and was purified by silica gel column chromatography (petroleum ether/ethyl acetate = 10:1) to obtain **16** as colorless oil (30.0 mg, 55% yield). **^{1}H NMR (400 MHz, CDCl$_3$) δ** 8.61 (d, *J* = 5.5 Hz, 1H), 7.70 (d, *J* = 9.0 Hz, 1H), 7.57 – 7.54 (m, 1H), 7.49 (d, *J* = 5.5 Hz, 1H), 7.32 – 7.27 (m, 1H), 3.94 (s, 3H), 0.98 (s, 9H), 0.57 (s, 6H). **^{13}C NMR (101 MHz, CDCl$_3$) δ** 167.8, 157.5, 141.2, 135.1, 130.0, 128.9, 122.2, 119.6, 106.6, 55.3, 27.2, 18.3, -3.2.

1-(tert-butyldimethylsilyl)-6-fluoroisoquinoline (17)[3]

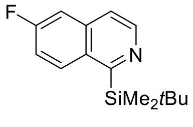

According to the general procedure, using 6-fluoroisoquinoline (29.4 mg, 0.2 mmol), tert-butyldimethylsilane (116.1 mg, 1.0 mmol), HCl (12 mol/L, 33 μL, 0.4 mmol) in MeCN (4 mL), and was purified by silica gel column chromatography (petroleum ether/ethyl acetate = 50:1) to obtain **17** as colorless oil (38.6 mg, 74% yield). **^{1}H NMR (400 MHz, CDCl$_3$) δ** 8.69 (d, *J* = 5.6 Hz, 1H), 8.30 – 8.22 (m, 1H), 7.51 (d, *J* = 5.7 Hz, 1H), 7.41 – 7.37 (m, 1H), 7.35 – 7.29 (m, 1H), 0.95 (s, 9H), 0.55 (s, 6H). **^{13}C NMR (101 MHz, CDCl$_3$) δ** 170.2, 162.2 (d, *J* = 251.6 Hz), 143.2, 136.2 (d, *J* = 10.2 Hz), 131.6 (d, *J* = 9.4 Hz), 131.3, 119.4 (d, *J* = 5.1 Hz), 116.8 (d, *J* = 25.0 Hz), 110.5 (d, *J* = 20.2 Hz), 27.0, 17.9, -3.1. **^{19}F NMR (376 MHz, CDCl$_3$) δ** -109.1.

1-(tert-butyldimethylsilyl)-6-chloroisoquinoline (18)[6]

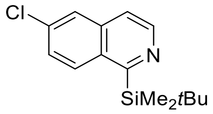

According to the general procedure, using 6-chloroisoquinoline (32.6 mg, 0.2 mmol), tert-butyldimethylsilane (116.1 mg, 1.0 mmol), HCl (12 mol/L, 33 μL, 0.4 mmol) in MeCN (4 mL), and was purified by silica gel column chromatography (petroleum ether/ethyl acetate = 50:1) to obtain **18** as colorless oil (28.2 mg, 51% yield). **^{1}H NMR (400 MHz, CDCl$_3$) δ** 8.71 (d, *J* = 5.7 Hz, 1H), 8.18 (d, *J* = 9.0 Hz, 1H), 7.78 (d, *J* = 2.1 Hz, 1H), 7.52 – 7.45 (m, 2H), 0.95 (s, 9H), 0.54 (s, 6H). **^{13}C NMR (101 MHz, CDCl$_3$) δ** 143.4, 141.7, 130.3, 129.0, 127.4, 126.8, 126.6, 126.3, 118.9, 31.2, 27.0, -3.1.

2-(tert-butyldimethylsilyl)-4-methylpyridine (20)[3]

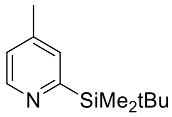

According to the general procedure, using 4-methylpyridine (18.6 mg, 0.2 mmol), tert-butyldimethylsilane (116.1 mg, 1.0 mmol), HCl (12 mol/L, 33 μL, 0.4 mmol) in MeCN (4 mL), and was purified by silica gel column chromatography (petroleum ether/ethyl acetate = 20:1) to obtain **20** as colorless oil (20.7 mg, 50% yield). **^{1}H NMR (400 MHz, CDCl$_3$) δ** 8.63 (d, *J* = 5.0 Hz, 1H), 7.30 (s, 1H), 7.03 – 6.97 (m, 1H), 2.32 (s, 3H), 0.91 (s, 9H), 0.31 (s, 6H). **^{13}C NMR (101 MHz, CDCl$_3$) δ** 165.9, 149.6, 144.2, 131.1, 123.5, 26.6, 21.1, 16.9, -6.3.

4-(tert-butyl)-2-(tert-butyldimethylsilyl)pyridine (21)[1]

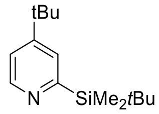

According to the general procedure, using 4-(tert-butyl)pyridine (27.0 mg, 0.2 mmol), tert-butyldimethylsilane (116.1 mg, 1.0 mmol), HCl (12 mol/L, 33 μL, 0.4 mmol) in MeCN (4 mL), and was purified by silica gel column chromatography (petroleum ether/ethyl acetate = 20:1) to obtain **21** as colorless oil (25.9 mg, 52% yield). **^{1}H NMR (400 MHz, $CDCl_3$) δ** 8.67 (d, J = 5.3 Hz, 1H), 7.48 (d, J = 2.0 Hz, 1H), 7.19 – 7.16 (m, 1H), 1.31 (s, 9H), 0.91 (s, 9H), 0.33 (s, 6H). **^{13}C NMR (101 MHz, $CDCl_3$) δ** 165.9, 156.9, 149.7, 126.9, 119.5, 30.5, 26.6, 16.9, -6.3.

2-(tert-butyldimethylsilyl)-4,6-dimethylpyridine (22)[3]

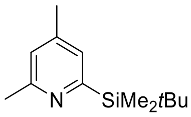

According to the general procedure, using 2,4-dimethylpyridine (21.4 mg, 0.2 mmol), tert-butyldimethylsilane (116.1 mg, 1.0 mmol), HCl (12 mol/L, 33 μL, 0.4 mmol) in MeCN (4 mL), and was purified by silica gel column chromatography (petroleum ether/ethyl acetate = 20:1) to obtain **22** as colorless oil (19.4 mg, 44% yield). **^{1}H NMR (400 MHz, $CDCl_3$) δ** 7.10 (s, 1H), 6.86 (s, 1H), 2.51 (s, 3H), 2.27 (s, 3H), 0.92 (s, 9H), 0.28 (s, 6H). **^{13}C NMR (101 MHz, $CDCl_3$) δ** 165.3, 157.7, 144.0, 128.1, 123.0, 26.7, 24.7, 20.9, 16.9, -6.1.

4-(tert-butyldimethylsilyl)-2,6-dimethylpyridine (23)[3]

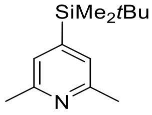

According to the general procedure, using 2,6-dimethylpyridine (21.4 mg, 0.2 mmol), tert-butyldimethylsilane (116.1 mg, 1.0 mmol), HCl (12 mol/L, 33 μL, 0.4 mmol) in MeCN (4 mL), and was purified by silica gel column chromatography (petroleum ether/ethyl acetate = 20:1) to obtain **23** as colorless oil (27.4 mg, 62% yield). **^{1}H NMR (400 MHz, $CDCl_3$) δ** 7.03 (s, 2H), 2.52 (s, 6H), 0.88 (s, 9H), 0.26 (s, 6H). **^{13}C NMR (101 MHz, $CDCl_3$) δ** 156.0, 148.1, 125.7, 26.4, 24.5, 16.7, -6.6.

2-(tert-butyldimethylsilyl)-4-fluoro-6-phenylpyridine (24)

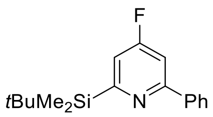

According to the general procedure, using 4-fluoro-2-phenylpyridine (34.6 mg, 0.2 mmol), tert-butyldimethylsilane (116.1 mg, 1.0 mmol), HCl (12 mol/L, 33 μL, 0.4 mmol) in MeCN (4 mL), and was purified by silica gel column chromatography (petroleum ether/ethyl acetate = 50:1) to obtain **24** as colorless oil (27.0 mg, 47% yield). **^{1}H NMR (400 MHz, $CDCl_3$) δ** 8.12 – 8.04 (m, 2H), 7.51 – 7.40 (m, 3H), 7.40 – 7.33 (m, 1H), 7.19 – 7.12 (m, 1H), 0.97 (s, 9H), 0.35 (s, 6H). **^{13}C NMR (101 MHz, $CDCl_3$) δ** 170.6, 168.6 (d, J = 262.4 Hz), 159.4 (d, J = 5.5 Hz), 138.8 (d, J = 3.3 Hz), 129.3, 128.7, 126.9, 115.5 (d, J = 12.2 Hz), 106.4 (d, J = 16.9 Hz), 26.6, 16.9, -6.3. **^{19}F NMR (376 MHz, $CDCl_3$) δ** -106.33. **HR-MS** (ESI) $C_{17}H_{23}FNSi^{+}$ $(M+H)^{+}$ 288.1578, found 288.1580.

4-(tert-butyldimethylsilyl)-2-chloro-6-methylpyridine (25)

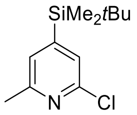

According to the general procedure, using 2-chloro-6-methylpyridine (25.4 mg, 0.2 mmol), tert-butyldimethylsilane (116.1 mg, 1.0 mmol), HCl (12 mol/L, 33 μL, 0.4 mmol) in MeCN (4 mL), and was purified by silica gel column chromatography (petroleum ether/ethyl acetate = 20:1) to obtain **25** as colorless oil (25.5 mg, 53% yield). **^{1}H NMR (400 MHz, $CDCl_3$) δ** 7.18 (s, 1H), 7.11 (s, 1H), 2.52 (s, 3H), 0.87 (s, 9H), 0.27 (s, 6H). **^{13}C NMR (101 MHz, $CDCl_3$) δ** 157.8, 152.4, 150.2, 127.0, 126.2, 26.3, 24.1, 16.7, -6.6. **HR-MS** (ESI) $C_{12}H_{21}ClNSi^{+}$ $(M+H)^{+}$ 242.1126, found 242.1128.

2-(tert-butyldimethylsilyl)-4-chloro-6-methylpyridine (26)[3]

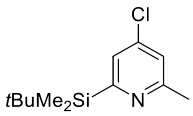

According to the general procedure, using 4-chloro-2-methylpyridine (25.4 mg, 0.2 mmol), tert-butyldimethylsilane (116.1 mg, 1.0 mmol), HCl (12 mol/L, 33 μL, 0.4 mmol) in MeCN (4 mL), and was purified by silica gel column chromatography (petroleum ether/ethyl acetate = 20:1) to obtain **26** as colorless oil (28.9 mg, 60% yield). **^{1}H NMR (400 MHz, $CDCl_3$) δ** 7.26 (d, J = 2.0 Hz, 1H), 7.05 (d, J = 1.9 Hz, 1H), 2.54 (s, 3H), 0.91 (s, 9H), 0.28 (s, 6H). **^{13}C NMR (101 MHz, $CDCl_3$) δ** 168.1, 159.6, 142.5, 126.8, 122.1, 26.6, 24.7, 16.9, -6.3.

3-(tert-butyldimethylsilyl)isonicotinonitrile (27)[6]

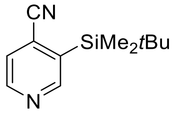

According to the general procedure, using isonicotinonitrile (20.8 mg, 0.2 mmol), tert-butyldimethylsilane (116.1 mg, 1.0 mmol), HCl (12 mol/L, 33 μL, 0.4 mmol) in MeCN (4 mL), and was purified by silica gel column chromatography (petroleum ether/ethyl acetate = 20:1) to obtain **27** as colorless oil (21.8 mg, 50% yield). **^{1}H NMR (400 MHz, $CDCl_3$) δ** 9.02 – 8.88 (m, 1H), 7.67 (s, 1H), 7.50 – 7.31 (m, 1H), 0.92 (s, 9H), 0.34 (s, 6H). **^{13}C NMR (101 MHz, $CDCl_3$) δ** 169.6, 150.3, 130.4, 123.5, 118.4, 117.1, 26.4, 16.9, -6.5.

Methyl 2-(tert-butyldimethylsilyl)isonicotinate (28)[1]

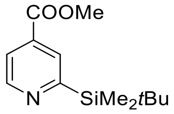

According to the general procedure, using methyl isonicotinate (27.4 mg, 0.2 mmol), tert-butyldimethylsilane (116.1 mg, 1.0 mmol), HCl (12 mol/L, 33 μL, 0.4 mmol) in MeCN (4 mL), and was purified by silica gel column chromatography (petroleum ether/ethyl acetate = 20:1) to obtain **28** as colorless oil (27.1 mg, 54% yield). **^{1}H NMR (400 MHz, $CDCl_3$) δ** 9.14 – 8.75 (m, 1H), 8.12 – 7.89 (m, 1H), 7.85 – 7.62 (m, 1H), 3.96 (s, 3H), 0.92 (s, 9H), 0.35 (s, 6H). **^{13}C NMR (101 MHz, $CDCl_3$) δ** 168.4, 150.5, 134.7, 128.2, 121.4, 52.6, 26.5, 16.9, -6.3.

1-(tert-butyldimethylsilyl)-5,6,7,8-tetrahydroisoquinoline (29)

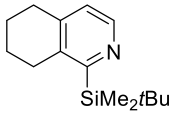

According to the general procedure, using methyl 5,6,7,8-tetrahydroisoquinoline (26.6 mg, 0.2 mmol), tert-butyldimethylsilane (116.1 mg, 1.0 mmol), HCl (12 mol/L, 33 μL, 0.4 mmol) in MeCN (4 mL), and was purified by silica gel column chromatography (petroleum ether/ethyl acetate = 30:1) to obtain **29** as colorless oil (14.8 mg, 30% yield). **^{1}H NMR (400 MHz, $CDCl_3$) δ** 8.46 (s, 1H), 7.16 (s, 1H), 2.77 – 2.67 (m, 4H), 1.86 – 1.76 (m, 4H), 0.91 (s, 9H), 0.29 (s, 6H). **^{13}C NMR (101 MHz, $CDCl_3$) δ** 155.0, 150.8, 148.0, 130.6, 127.8, 29.7, 28.5, 26.6, 26.2, 22.6, 22.5, -6.2. **HR-MS** (ESI) $C_{15}H_{26}NSi^+$ $(M+H)^+$ 248.1829, found 248.1833.

6-(tert-butyldimethylsilyl)-4,4'-dimethyl-2,2'-bipyridine (30)

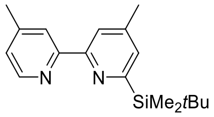

According to the general procedure, using methyl 4,4'-dimethyl-2,2'-bipyridine (36.8 mg, 0.2 mmol), tert-butyldimethylsilane (116.1 mg, 1.0 mmol), HCl (12 mol/L, 33 μL, 0.4 mmol) in MeCN (4 mL), and was purified by silica gel column chromatography (petroleum ether/ethyl acetate = 20:1) to obtain **30** as colorless oil (18 mg, 30% yield). **^{1}H NMR (400 MHz, CDCl$_3$) δ** 8.52 (d, *J* = 5.0 Hz, 1H), 8.36 (s, 1H), 8.17 (s, 1H), 7.31 (s, 1H), 7.12 (d, *J* = 4.5 Hz, 1H), 2.45 (s, 3H), 2.41 (s, 3H), 0.97 (s, 9H), 0.35 (s, 6H). **^{13}C NMR (101 MHz, CDCl$_3$) δ** 148.6, 145.2, 143.2, 138.4, 135.5, 130.7, 129.9, 124.4, 122.2, 120.7, 29.4, 29.3, 26.7, 21.4, -6.1. **HR-MS** (ESI) $C_{16}H_{27}N_2Si^+$ $(M+H)^+$ 299.1938, found 299.1941.

4,4'-di-tert-butyl-6-(tert-butyldimethylsilyl)-2,2'-bipyridine (31)[1]

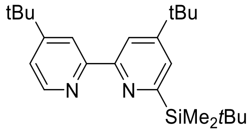

According to the general procedure, using methyl 4,4'-di-tert-butyl-2,2'-bipyridine (53.6 mg, 0.2 mmol), tert-butyldimethylsilane (116.1 mg, 1.0 mmol), HCl (12 mol/L, 33 μL, 0.4 mmol) in MeCN (4 mL), and was purified by silica gel column chromatography (petroleum ether/ethyl acetate = 20:1) to obtain **31** as colorless oil (32.1 mg, 42% yield). **^{1}H NMR (400 MHz, CDCl$_3$) δ** 8.66 (s, 1H), 8.57 (s, 1H), 8.33 (s, 1H), 7.48 (s, 1H), 7.29 – 7.24 (m, 1H), 1.37 (s, 18H), 1.00 (s, 9H), 0.36 (s, 6H). **^{13}C NMR (101 MHz, CDCl$_3$) δ** 165.3, 160.5, 158.1, 157.2, 155.5, 148.8, 126.4, 120.4, 118.6, 116.6, 34.9, 34.8, 30.7, 30.5, 26.7, 17.0, -6.1.

4-(tert-butyldimethylsilyl)-3-chloro-6-methylpyridazine (32)

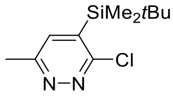

According to the general procedure, using methyl 3-chloro-6-methylpyridazine (25.6 mg, 0.2 mmol), tert-butyldimethylsilane (116.1 mg, 1.0 mmol), HCl (12 mol/L, 33 μL, 0.4 mmol) in MeCN (4 mL), and was purified by silica gel column chromatography (petroleum ether/ethyl acetate = 10:1) to obtain **32** as colorless oil (14 mg, 29% yield). **^{1}H NMR (400 MHz, CDCl$_3$) δ** 7.32 (s, 1H), 2.67

(s, 3H), 0.94 (s, 9H), 0.42 (s, 6H). **^{13}C NMR (101 MHz, CDCl$_3$) δ** 159.9, 157.8, 138.8, 136.6, 26.9, 21.5, 17.8, -4.6. $C_{11}H_{20}ClN_2Si^+$ (M+H)$^+$ 243.1079, found 243.1079.

4-(tert-butyldimethylsilyl)-3,6-dichloropyridazine (33)[7]

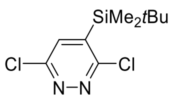

According to the general procedure, using methyl 3,6-dichloropyridazine (29.6 mg, 0.2 mmol), tert-butyldimethylsilane (116.1 mg, 1.0 mmol), HCl (12 mol/L, 33 μL, 0.4 mmol) in MeCN (4 mL), and was purified by silica gel column chromatography (petroleum ether/ethyl acetate = 10:1) to obtain **33** as white solid (33.1 mg, 63% yield). **^{1}H NMR (400 MHz, CDCl$_3$) δ** 7.48 (s, 1H), 0.96 (s, 9H), 0.44 (s, 6H). **^{13}C NMR (101 MHz, CDCl$_3$) δ** 160.7, 155.6, 143.1, 137.1, 26.8, 17.9, -4.8.

4-(tert-butyldimethylsilyl)-3,6-dimethoxypyridazine (34)

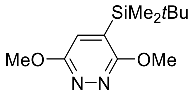

According to the general procedure, using methyl 3,6-dimethoxypyridazine (28.0 mg, 0.2 mmol), tert-butyldimethylsilane (116.1 mg, 1.0 mmol), HCl (12 mol/L, 33 μL, 0.4 mmol) in MeCN (4 mL), and was purified by silica gel column chromatography (petroleum ether/ethyl acetate = 10:1) to obtain **34** as white solid (26.4 mg, 52% yield). **^{1}H NMR (400 MHz, CDCl$_3$) δ** 6.96 (s, 1H), 4.03 (s, 3H), 4.00 (s, 3H), 0.88 (s, 9H), 0.27 (s, 6H). **^{13}C NMR (101 MHz, CDCl$_3$) δ** 164.8, 161.7, 133.1, 128.3, 54.3, 54.1, 26.8, 17.2, -5.8. **HR-MS** (ESI) $C_{12}H_{23}N_2O_2Si^+$ (M+H)$^+$ 255.1523, found 255.1525.

5-(tert-butyldimethylsilyl)-2-chloropyrimidine (35)

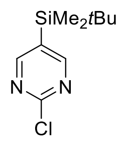

According to the general procedure, using methyl 2-chloropyrimidine (22.8 mg, 0.2 mmol), tert-butyldimethylsilane (116.1 mg, 1.0 mmol), HCl (12 mol/L, 33 μL, 0.4 mmol) in MeCN (4 mL), and was purified by silica gel column chromatography (petroleum ether/ethyl acetate = 10:1) to obtain **35** as white solid (20.9 mg, 46% yield). **^{1}H NMR (400 MHz, CDCl$_3$) δ** 8.49 (d, J = 4.6 Hz, 1H), 7.39 (d, J = 4.7 Hz, 1H), 0.94 (s, 9H), 0.33 (s, 6H). **^{13}C NMR (101 MHz, CDCl$_3$) δ** 181.9, 157.0, 125.6, 26.4, 16.8, -6.8. **HR-MS** (ESI) $C_{10}H_{18}ClN_2Si^+$ (M+H)$^+$ 229.0922, found 229.0923.

7-(tert-butyldimethylsilyl)imidazo[1,2-b]pyridazine (36)[1]

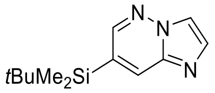

According to the general procedure, using methyl imidazo[1,2-b]pyridazine (23.8 mg, 0.2 mmol), tert-butyldimethylsilane (116.1 mg, 1.0 mmol), HCl (12 mol/L, 33 μL, 0.4 mmol) in MeCN (4 mL), and was purified by silica gel column chromatography (petroleum ether/ethyl acetate = 5:1) to obtain **36** as colorless oil (14.9 mg, 32% yield). **^{1}H NMR (500 MHz, $CDCl_3$) δ** 8.30 (s, 1H), 8.07 (s, 1H), 7.96 (s, 1H), 7.78 (s, 1H), 0.93 (s, 9H), 0.36 (s, 6H). **^{13}C NMR (126 MHz, $CDCl_3$) δ** 146.6, 133.8, 132.3, 130.1, 126.6, 116.4, 26.3, 17.0, -6.4.

8-(tert-butyldimethylsilyl)imidazo[1,2-b]pyridazine (36')[1]

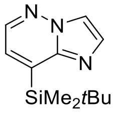

According to the general procedure, using methyl imidazo[1,2-b]pyridazine (23.8 mg, 0.2 mmol), tert-butyldimethylsilane (116.1 mg, 1.0 mmol), HCl (12 mol/L, 33 μL, 0.4 mmol) in MeCN (4 mL), and was purified by silica gel column chromatography (petroleum ether/ethyl acetate = 5:1) to obtain **36'** as colorless oil (14.0 mg, 30% yield). **^{1}H NMR (400 MHz, $CDCl_3$) δ** 8.21 (d, J = 4.2 Hz, 1H), 7.92 (s, 1H), 7.79 (s, 1H), 7.05 (d, J = 4.3 Hz, 1H), 0.96 (s, 9H), 0.49 (s, 6H). **^{13}C NMR (101 MHz, $CDCl_3$) δ** 142.2, 141.8, 138.6, 133.2, 123.3, 115.7, 26.9, 17.4, -5.4.

7-(tert-butyldimethylsilyl)-6-chloro-[1,2,4]triazolo[4,3-b]pyridazine (37)[1]

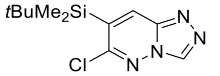

According to the general procedure, using methyl 6-chloro-[1,2,4]triazolo[4,3-b]pyridazine (30.8 mg, 0.2 mmol), tert-butyldimethylsilane (116.1 mg, 1.0 mmol), HCl (12 mol/L, 33 μL, 0.4 mmol) in MeCN (4 mL), and was purified by silica gel column chromatography (petroleum ether/ethyl acetate = 5:1) to obtain 37 as colorless solid (16.1 mg, 30% yield). **^{1}H NMR (400 MHz, $CDCl_3$) δ** 9.02 (s, 1H), 8.27 (s, 1H), 0.98 (s, 9H), 0.47 (s, 6H). **^{13}C NMR (101 MHz, $CDCl_3$)** δ 154.0, 137.9, 134.3, 134.2, 129.0, 27.0, 17.8, -4.1.

8-(tert-butyldimethylsilyl)-6-chloro-[1,2,4]triazolo[4,3-b]pyridazine (37')

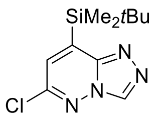

According to the general procedure, using methyl 6-chloro-[1,2,4]triazolo[4,3-b]pyridazine (30.8 mg, 0.2 mmol), tert-butyldimethylsilane (116.1 mg, 1.0 mmol), HCl (12 mol/L, 33 μL, 0.4 mmol) in MeCN (4 mL), and was purified by silica gel column chromatography (petroleum ether/ethyl acetate = 5:1) to obtain 37' as colorless solid (10.7 mg, 20% yield). **^{1}H NMR (400 MHz, $CDCl_3$) δ** 9.00 (s, 1H), 7.08 (s, 1H), 0.99 (s, 9H), 0.52 (s, 6H). **^{13}C NMR (101 MHz, $CDCl_3$) δ** 149.1, 145.5, 142.2, 137.9, 128.5, 26.8, 17.3, -5.7. **HR-MS** (ESI) $C_{11}H_{18}ClN_4Si^+$ $(M+H)^+$ 269.0984, found 269.0987.

1,4-bis(tert-butyldimethylsilyl)phthalazine (38)[8]

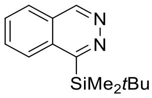

According to the general procedure, using phthalazine (26.0 mg, 0.2 mmol), tert-butyldimethylsilane (116.1 mg, 1.0 mmol), HCl (12 mol/L, 33 μL, 0.4 mmol) in MeCN (4 mL), and was purified by silica gel column chromatography (petroleum ether/ethyl acetate = 10:1) to obtain 38 as colorless oil (19.5 mg, 40% yield, 2:1). **^{1}H NMR (400 MHz, $CDCl_3$) δ** 9.55 (s, 1H), 9.43 (s, 1H), 8.20 – 8.14 (m, 1H), 8.00 – 7.94 (m, 1H), 7.93 – 7.89 (m, 1H), 7.88 – 7.84 (m, 2H), 0.99 (s, 9H), 0.60 (s, 6H). **^{13}C NMR (101 MHz, $CDCl_3$) δ** 151.1, 150.1, 132.7, 131.8, 131.4, 127.3, 127.3, 126.3, 27.0, 25.6, 17.8, -3.3, -3.6.

2-(tert-butyldimethylsilyl)-6-(thiophen-2-yl)pyridine (39)

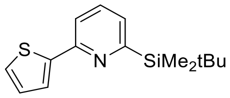

According to the general procedure, using 2-(thiophen-2-yl)pyridine (33.2 mg, 0.2 mmol), tert-butyldimethylsilane (116.1 mg, 1.0 mmol), HCl (12 mol/L, 33 μL, 0.4 mmol) in MeCN (4 mL), and was purified by silica gel column chromatography (petroleum ether/ethyl acetate = 50:1) to obtain **39** as colorless oil (11.0 mg, 20% yield). **^{1}H NMR (400 MHz, Chloroform-*d*)** δ 7.61 – 7.52 (m, 3H), 7.37 – 7.30 (m, 2H), 7.13 – 7.07 (m, 1H), 0.96 (s, 9H), 0.32 (s, 6H). **^{13}C NMR (101 MHz, $CDCl_3$)** δ 151.9, 148.1, 134.1, 127.8, 127.8, 127.4, 123.7, 122.2, 117.2, 26.6, 16.9, -6.3.

$C_{15}H_{22}NSSi^+$ (M+H)$^+$ 276.1237, found 276.1236.

6-(tert-butyldimethylsilyl)thieno[2,3-*b*]pyridine (40)

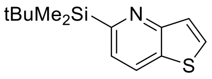

According to the general procedure, using thieno[2,3-*b*]pyridine (27.0 mg, 0.2 mmol), tert-butyldimethylsilane (116.1 mg, 1.0 mmol), HCl (12 mol/L, 33 μL, 0.4 mmol) in MeCN (4 mL), and was purified by silica gel column chromatography (petroleum ether/ethyl acetate = 30:1) to obtain **40** as yellow solid (30.9 mg, 62% yield, 1:2.3). **^{1}H NMR (400 MHz, Chloroform-*d*)** δ 8.08 (d, *J* = 8.1 Hz, 1H), 7.69 (d, *J* = 5.5 Hz, 1H), 7.62 (d, *J* = 5.5 Hz, 1H), 7.44 (d, *J* = 8.1 Hz, 1H), 0.95 (s, 9H), 0.38 (s, 6H). **^{13}C NMR (101 MHz, CDCl$_3$)** δ 164.0, 156.7, 131.1, 129.5, 127.8, 125.8, 124.3, 26.6, 17.1, -6.0. $C_{13}H_{20}NSSi^+$ (M+H)$^+$ 250.1080, found 250.1083.

6-(tert-butyldimethylsilyl)thieno[3,2-*c*]pyridine (41)

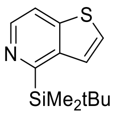

According to the general procedure, using thieno[3,2-*c*]pyridine (27.0 mg, 0.2 mmol), tert-butyldimethylsilane (116.1 mg, 1.0 mmol), HCl (12 mol/L, 33 μL, 0.4 mmol) in MeCN (4 mL), and was purified by silica gel column chromatography (petroleum ether/ethyl acetate = 30:1) to obtain **41** as colorless oil (31.4 mg, 63% yield). **^{1}H NMR (400 MHz, Chloroform-*d*)** δ 8.62 (d, *J* = 5.5 Hz, 1H), 7.75 (d, *J* = 5.5 Hz, 1H), 7.61 (d, *J* = 5.6 Hz, 1H), 7.46 (d, *J* = 5.6 Hz, 1H), 0.94 (s, 9H), 0.50 (s, 6H). **^{13}C NMR (101 MHz, CDCl$_3$)** δ 163.4, 142.2, 132.5, 129.9, 126.3, 124.3, 116.4, 26.8, 17.8, -4.3. $C_{13}H_{20}NSSi^+$ (M+H)$^+$ 250.1080, found 250.1082.

5-(tert-butyldimethylsilyl)-1-methyl-1*H*-pyrrolo[3,2-*b*]pyridine (42)

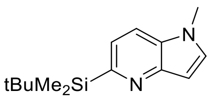

According to the general procedure, using 1-methyl-1*H*-pyrrolo[3,2-*b*]pyridine (26.4 mg, 0.2 mmol), tert-butyldimethylsilane (116.1 mg, 1.0 mmol), HCl (12 mol/L, 33 μL, 0.4 mmol) in MeCN (2 mL) and DMSO (2 mL), and was purified by silica gel column chromatography (petroleum ether/ethyl acetate = 5:1) to obtain **42** as yellow solid (22.1 mg, 45% yield). **^{1}H NMR (400 MHz, Chloroform-*d*)** δ 7.54 (d, *J* = 8.2 Hz, 1H), 7.36 (d, *J* = 8.2 Hz, 1H), 7.26 (s, 1H), 6.82 – 6.78 (m,

1H), 3.79 (s, 3H), 0.93 (s, 9H), 0.39 (s, 6H). **^{13}C NMR (101 MHz, $CDCl_3$)** δ 169.8, 142.1, 131.8, 122.6, 114.0, 104.5, 102.4, 32.8, 26.7, 17.2, -5.8. $C_{14}H_{23}N_2Si^+$ $(M+H)^+$ 247.1625, found 247.1625.

6-(tert-butyldimethylsilyl)-1-methyl-1*H*-pyrrolo[2,3-*b*]pyridine (43)

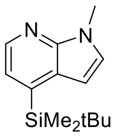

According to the general procedure, using 1-methyl-1*H*-pyrrolo[2,3-*b*]pyridine (26.4 mg, 0.2 mmol), tert-butyldimethylsilane (116.1 mg, 1.0 mmol), HCl (12 mol/L, 33 μL, 0.4 mmol) in MeCN (2 mL) and DMSO (2 mL), and was purified by silica gel column chromatography (petroleum ether/ethyl acetate = 5:1) to obtain **43** as colorless oil (20.2 mg, 41% yield). **^{1}H NMR (400 MHz, Chloroform-*d*)** δ 8.30 (d, *J* = 4.6 Hz, 1H), 7.19 (d, *J* = 3.5 Hz, 1H), 7.15 (d, *J* = 4.6 Hz, 1H), 6.53 (d, *J* = 3.5 Hz, 1H), 3.89 (s, 3H), 0.91 (s, 9H), 0.42 (s, 6H). **^{13}C NMR (101 MHz, $CDCl_3$)** δ 146.3, 141.4, 129.9, 128.6, 122.2, 115.4, 101.5, 31.3, 26.7, 17.5, -5.1. $C_{14}H_{23}N_2Si^+$ $(M+H)^+$ 247.1625, found 247.1623.

5-(tert-butyldimethylsilyl)-1*H*-pyrrolo[3,2-*b*]pyridine (44)

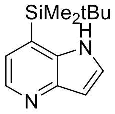

According to the general procedure, using 1H-pyrrolo[3,2-b]pyridine (23.6 mg, 0.2 mmol), tert-butyldimethylsilane (116.1 mg, 1.0 mmol), HCl (12 mol/L, 33 μL, 0.4 mmol) in MeCN (4 mL), and was purified by silica gel column chromatography (petroleum ether/ethyl acetate = 5:1) to obtain **44** as colorless oil (19.0 mg, 32% yield). **^{1}H NMR (400 MHz, Chloroform-*d*)** δ 8.45 (d, 1H), 8.18 (s, 1H), 7.47 – 7.41 (m, 1H), 7.16 (d, *J* = 4.6 Hz, 1H), 6.78 – 6.73 (m, 1H), 0.93 (s, 9H), 0.47 (s, 6H). **^{13}C NMR (101 MHz, $CDCl_3$)** δ 144.8, 142.6, 127.6, 127.4, 123.4, 123.2, 103.4, 26.4, 17.6, -5.2. $C_{13}H_{21}N_2Si^+$ $(M+H)^+$ 233.1469, found 233.1472.

6-(tert-butyldimethylsilyl)-1*H*-pyrrolo[2,3-*b*]pyridine (45)

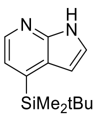

According to the general procedure, using 1*H*-pyrrolo[2,3-*b*]pyridine (23.6 mg, 0.2 mmol), tert-butyldimethylsilane (116.1 mg, 1.0 mmol), HCl (12 mol/L, 33 μL, 0.4 mmol) in MeCN (4 mL), and

was purified by silica gel column chromatography (petroleum ether/ethyl acetate = 5:1) to obtain **45** as colorless oil (17.6 mg, 38% yield). **^{1}H NMR (400 MHz, Chloroform-*d*)** δ 9.45 (s, 1H), 8.31 – 8.23 (m, 1H), 7.34 (d, *J* = 3.5 Hz, 1H), 7.18 (d, *J* = 4.6 Hz, 1H), 6.59 (d, *J* = 3.5 Hz, 1H), 0.92 (s, 9H), 0.43 (s, 6H). **^{13}C NMR (101 MHz, $CDCl_3$)** δ 146.9, 141.5, 140.4, 124.4, 122.7, 113.9, 103.2, 26.7, 17.5, -5.1. $C_{13}H_{21}N_2Si^+$ $(M+H)^+$ 233.1469, found 233.1470.

5-(tert-butyldimethylsilyl)-1*H*-pyrrolo[2,3-*c*]pyridine (46)[1]

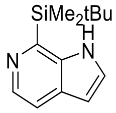

According to the general procedure, using 1*H*-pyrrolo[2,3-*c*]pyridine (23.6 mg, 0.2 mmol), tert-butyldimethylsilane (116.1 mg, 1.0 mmol), HCl (12 mol/L, 33 μL, 0.4 mmol) in MeCN (4 mL), and was purified by silica gel column chromatography (petroleum ether/ethyl acetate = 5:1) to obtain **46** as colorless solid (14.3 mg, 31% yield). **^{1}H NMR (400 MHz, Chloroform-*d*)** δ 8.52 – 8.42 (m, 2H), 7.53 (d, *J* = 5.5 Hz, 1H), 7.41 – 7.36 (m, 1H), 6.60 – 6.53 (m, 1H), 0.96 (s, 9H), 0.55 (s, 6H). ^{13}C NMR (101 MHz, $CDCl_3$) δ 138.8, 132.2, 130.9, 129.9, 128.8, 114.8, 101.9, 26.6, 17.9, -5.0.

4-(tert-butyldimethylsilyl)-9*H*-pyrido[2,3-*b*]indole (47)

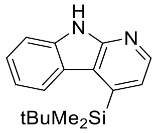

According to the general procedure, using 9*H*-pyrido[2,3-*b*]indole (33.6 mg, 0.2 mmol), tert-butyldimethylsilane (116.1 mg, 1.0 mmol), HCl (12 mol/L, 33 μL, 0.4 mmol) in MeCN (4 mL), and was purified by silica gel column chromatography (petroleum ether/ethyl acetate = 20:1) to obtain **47** as colorless oil (25.4 mg, 45% yield). **^{1}H NMR (400 MHz, Chloroform-*d*)** δ 9.57 – 9.30 (m, 1H), 8.45 – 8.36 (m, 1H), 8.28 (d, *J* = 8.1 Hz, 1H), 7.55 – 7.43 (m, 2H), 7.34 (d, *J* = 4.9 Hz, 1H), 7.26 – 7.23 (m, 1H), 1.02 (s, 9H), 0.59 (s, 6H). **^{13}C NMR (101 MHz, $CDCl_3$)** δ 151.4, 143.9, 142.4, 129.9, 126.4, 123.9, 122.9, 122.0, 119.4, 114.8, 111.0, 27.2, 18.5, -3.4. $C_{17}H_{23}N_2Si^+$ $(M+H)^+$ 283.1625, found 283.1627.

2-(tert-butyldimethylsilyl)terephthalonitrile (48)[1]

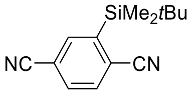

According to the general procedure, using terephthalonitrile (25.6 mg, 0.2 mmol), tert-butyldimethylsilane (116.1 mg, 1.0 mmol), HCl (12 mol/L, 33 μL, 0.4 mmol) in MeCN (4 mL), and was purified by silica gel column chromatography (petroleum ether/ethyl acetate = 30:1) to obtain **48** as colorless solid (20.3 mg, 42% yield). **^{1}H NMR (400 MHz, $CDCl_3$) δ** 7.86 – 7.84 (m, 1H), 7.81 – 7.78 (m, 1H), 7.75 – 7.70 (m, 1H), 0.94 (s, 9H), 0.49 (s, 6H). **^{13}C NMR (101 MHz, $CDCl_3$) δ** 144.6, 139.3, 134.2, 132.2, 26.5, 18.0, -5.1.

3-(tert-butyldimethylsilyl)-2,5-dichloroterephthalonitrile (49)[1]

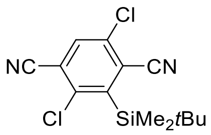

According to the general procedure, using 2,5-dichloroterephthalonitrile (39.0 mg, 0.2 mmol), tert-butyldimethylsilane (116.1 mg, 1.0 mmol), HCl (12 mol/L, 33 μL, 0.4 mmol) in MeCN (4 mL), and was purified by silica gel column chromatography (petroleum ether/ethyl acetate = 30:1) to obtain **49** as colorless solid (14.3 mg, 23% yield). **^{1}H NMR (400 MHz, $CDCl_3$) δ** 7.82 (s, 1H), 1.04 (s, 9H), 0.66 (s, 6H). **^{13}C NMR (101 MHz, $CDCl_3$) δ** 146.5, 142.0, 137.8, 134.6, 124.5, 119.2, 115.6, 114.3, 27.1, 19.9, -0.1.

2-(tert-butyldiphenylsilyl)-4-methylquinoline (50)[3]

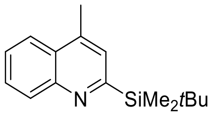

According to the general procedure, using 4-methylquinoline (28.6 mg, 0.2 mmol), tert-butyldiphenylsilane (240.1 mg, 1.0 mmol), HCl (12 mol/L, 33 μL, 0.4 mmol) in MeCN (4 mL), and was purified by silica gel column chromatography (petroleum ether/ethyl acetate = 100:1) to obtain **50** as colorless oil (48.8 mg, 64% yield). **^{1}H NMR (400 MHz, $CDCl_3$) δ** 8.32 – 8.17 (m, 1H), 8.02 – 7.93 (m, 1H), 7.81 – 7.67 (m, 5H), 7.61 – 7.55 (m, 1H), 7.45 – 7.35 (m, 6H), 7.23 – 7.15 (m, 1H), 2.57 (s, 3H), 1.29 – 1.27 (m, 9H). **^{13}C NMR (101 MHz, $CDCl_3$) δ** 166.3, 148.3, 140.4, 136.7, 136.6, 136.5, 134.5, 131.0, 129.2, 128.8, 128.5, 127.7, 127.6, 127.6, 127.2, 126.4, 123.7, 28.1, 19.2, 18.6.

4-methyl-2-(triisopropylsilyl)quinoline (51)[1]

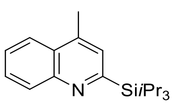

According to the general procedure, using 4-methylquinoline (28.6 mg, 0.2 mmol), triisopropylsilane (158.1 mg, 1.0 mmol), HCl (12 mol/L, 33 μL, 0.4 mmol) in MeCN (4 mL), and was purified by silica gel column chromatography (petroleum ether/ethyl acetate = 50:1) to obtain **51** as colorless oil (37.7 mg, 63% yield). **^{1}H NMR (400 MHz, CDCl$_3$) δ** 8.15 (d, J = 8.4 Hz, 1H), 7.98 (d, J = 8.3 Hz, 1H), 7.70 – 7.63 (m, 1H), 7.56 – 7.49 (m, 1H), 7.39 – 7.36 (m, 1H), 2.72 – 2.66 (m, 3H), 1.64 – 1.52 (m, 3H), 1.17 (d, J = 7.5 Hz, 18H). **^{13}C NMR (101 MHz, CDCl$_3$) δ** 167.3, 148.5, 139.7, 130.9, 128.2, 127.7, 127.2, 125.9, 123.6, 18.8, 18.7, 11.2.

Ethyl 4-(4-(tert-butyldimethylsilyl)-8-chloro-5,6-dihydro-11H-benzo[5,6]cyclohepta[1,2-b]pyridine-11-ylidene)piperidine-1-carboxylate (53)

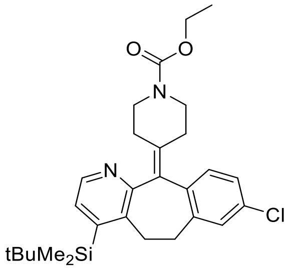

According to the general procedure, using ethyl 4-(8-chloro-5,6-dihydro-11*H*-benzo[5,6]cyclohepta[1,2-*b*]pyridin-11-ylidene)piperidine-1-carboxylate (76.4 mg, 0.2 mmol), tert-butyldimethylsilane (116.1 mg, 1.0 mmol), HCl (12 mol/L, 33 μL, 0.4 mmol) in DMSO (4 mL), and was purified by silica gel column chromatography (petroleum ether/ethyl acetate = 10:1) to obtain **53** as colorless solid (44.7 mg, 45% yield).^{1}H NMR (400 MHz, Chloroform-*d*) δ 8.34 (d, J = 4.9 Hz, 1H), 7.24 (d, J = 5.0 Hz, 1H), 7.15 – 7.08 (m, 2H), 7.06 (s, 1H), 4.13 (q, J = 7.1 Hz, 2H), 3.84 – 3.69 (m, 2H), 3.47 – 3.19 (m, 3H), 3.12 (s, 1H), 3.05 – 2.96 (m, 1H), 2.93 – 2.81 (m, 1H), 2.50 – 2.41 (m, 2H), 2.36 – 2.25 (m, 2H), 2.07 (s, 3H), 0.90 (s, 9H), 0.38 (d, J = 26.3 Hz, 6H). ^{13}C NMR (101 MHz, CDCl$_3$) δ 159.5, 155.5, 146.1, 145.1, 139.1, 138.3, 136.1, 134.8, 134.6, 132.9, 131.7, 130.2, 129.6, 125.8, 61.4, 44.8, 44.4, 32.9, 30.6, 30.3, 30.2, 26.8, 17.5, 14.7, -3.1, -3.8. $C_{28}H_{38}ClN_2O_2Si^+$ $(M+H)^+$ 497.2386, found 497.2392.

Heptan-2-yl 2-((2-(tert-butyldimethylsilyl)-5-chloroquinolin-8-yl)oxy)acetate (54)

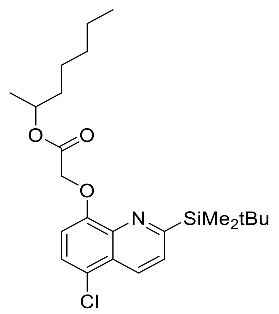

According to the general procedure, using heptan-2-yl 2-((5-chloroquinolin-8-yl)oxy)acetate (67.0 mg, 0.2 mmol), tert-butyldimethylsilane (116.1 mg, 1.0 mmol), HCl (12 mol/L, 33 μL, 0.4 mmol) in MeCN (2 mL) and DMSO (2 mL), and was purified by silica gel column chromatography (petroleum ether/ethyl acetate = 20:1) to obtain **54** as colorless solid (37.7 mg, 42% yield). ^{1}H NMR (400 MHz, Chloroform-*d*) δ 8.40 (d, *J* = 8.5 Hz, 1H), 7.71 (d, *J* = 8.5 Hz, 1H), 7.48 (d, *J* = 8.3 Hz, 1H), 7.19 (d, *J* = 8.3 Hz, 1H), 5.11 (s, 2H), 5.05 – 4.99 (m, 1H), 1.58 (s, 2H), 1.30 – 1.21 (m, 9H), 0.96 (s, 9H), 0.90 – 0.82 (m, 3H), 0.42 (s, 6H).^{13}C NMR (101 MHz, $CDCl_3$) δ 169.1, 156.9, 153.3, 141.9, 134.5, 129.8, 127.1, 126.1, 120.8, 115.4, 72.2, 68.9, 35.8, 31.6, 26.6, 25.0, 22.5, 19.9, 17.1, 14.0, -6.2. $C_{24}H_{37}ClNO_3Si^+$ $(M+H)^+$ 450.2226, found 450.2233.

(3*S*,8*R*,9*S*,10*R*,13*S*,14*S*)-17-(6-(tert-butyldimethylsilyl)pyridin-3-yl)-10,13-dimethyl-2,3,4,7,8,9,10,11,12,13,14,15-dodecahydro-1*H*-cyclopenta[*a*]phenanthren-3-yl acetate (55)

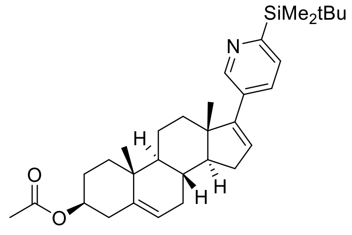

According to the general procedure, using (3*S*,8*R*,9*S*,10*R*,13*S*,14*S*)-10,13-dimethyl-17-(pyridin-3-yl)-2,3,4,7,8,9,10,11,12,13,14,15-dodecahydro-1*H*-cyclopenta[*a*]phenanthren-3-yl acetate (78.2 mg, 0.2 mmol), tert-butyldimethylsilane (116.1 mg, 1.0 mmol), HCl (12 mol/L, 33 μL, 0.4 mmol) in MeCN (2 mL) and DMSO (2 mL), and was purified by silica gel column chromatography (petroleum ether/ethyl acetate = 5:1) to obtain 13 as colorless solid (36.3 mg, 36% yield).^{1}H NMR (400 MHz, Chloroform-*d*) δ 8.87 – 8.79 (m, 1H), 7.60 – 7.51 (m, 1H), 7.43 (d, *J* = 7.9 Hz, 1H), 6.06 – 5.98 (m, 1H), 5.47 – 5.28 (m, 2H), 4.70 – 4.53 (m, 1H), 2.04 (s, 3H), 1.58 (s, 8H), 1.26 (s, 8H), 1.07 (d, *J* = 9.8 Hz, 6H), 0.93 (s, 9H), 0.32 (s, 6H). ^{13}C NMR (101 MHz, CDCl3) δ 170.5, 165.0,

151.9, 147.8, 140.0, 131.1, 129.5, 129.2, 122.3, 73.9, 57.4, 50.2, 47.2, 38.1, 36.9, 36.8, 35.2, 31.8, 31.5, 30.4, 29.3, 27.7, 27.2, 26.6, 21.4, 20.8, 19.2, 16.6, -6.3. $C_{32}H_{48}NO_2Si^+$ $(M+H)^+$ 506.3449, found 506.3456.

6. Mechanism study

6.1 Radical trapping experiments

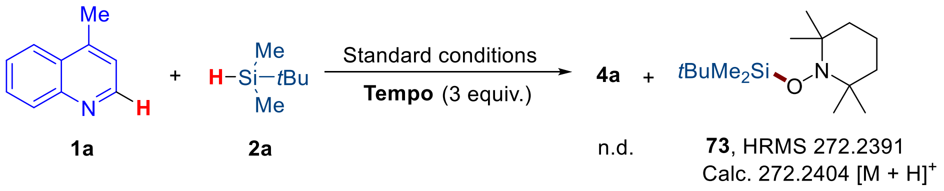

A 10 mL tube equipped with a magnetic stir bar was charged with 4-methylquinoline **1a** (0.2 mmol, 1 equiv.), trialkylsilanes **2a** (1.0 mmol, 5.0 equiv.), HCl (0.4 mmol, 2 equiv.), TEMPO (0.6 mmol, 3 equiv) and 4 mL of CH_3CN. The mixture was irradiated by blue LEDs (20W, λ = 365 nm) in the air at room temperature. After being stirred at 25 °C for 12 h, the reaction mixture was analyzed by HR-MS.

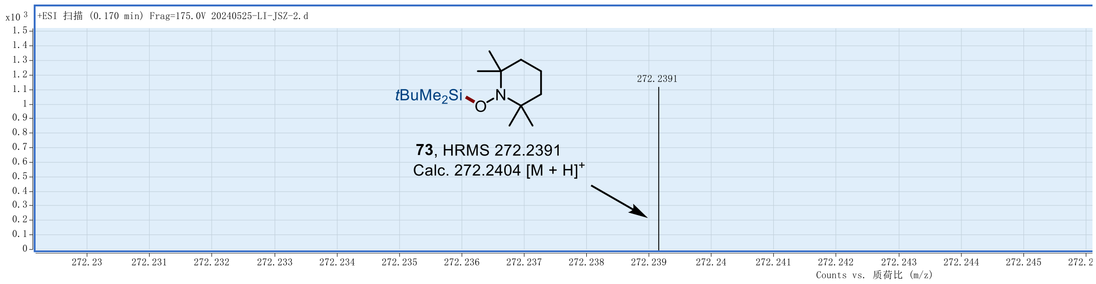

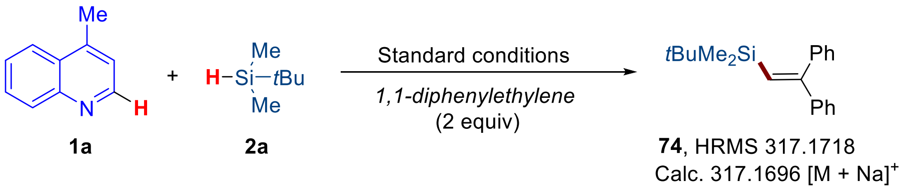

A 10 mL tube equipped with a magnetic stir bar was charged with 4-methylquinoline **1a** (0.2 mmol, 1 equiv.), trialkylsilanes **2a** (1.0 mmol, 5.0 equiv.), HCl (0.4 mmol, 2 equiv.), 1,1-diphenylethylene (0.4 mmol, 2 equiv.) and 4 mL of CH_3CN. The mixture was irradiated by blue LEDs (20W, λ = 365 nm) in the air at room temperature. After being stirred at 25 °C for 12 h, the reaction mixture was analyzed by HR-MS.

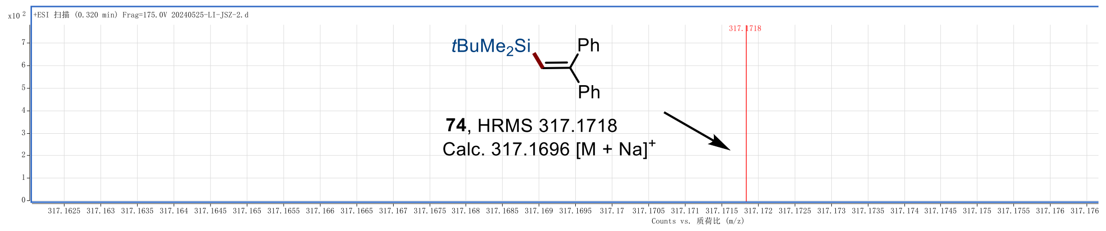

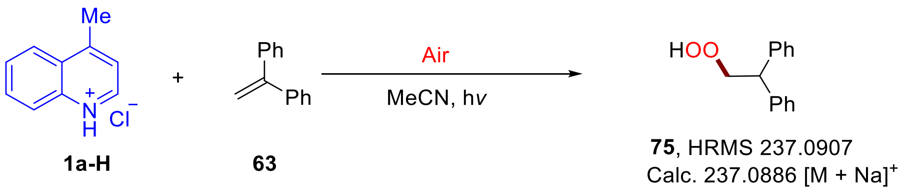

A 10 mL tube equipped with a magnetic stir bar was charged with 4-methylquinoline hydrochlorides **1a-H** (0.2 mmol, 1 equiv.), 1,1-diphenylethylene (0.4 mmol, 2 equiv.) and 4 mL of CH_3CN. The mixture was irradiated by blue LEDs (20W, $\lambda = 365$ nm) in the air at room temperature. After being stirred at 25 °C for 12 h, the reaction mixture was analyzed by HR-MS.

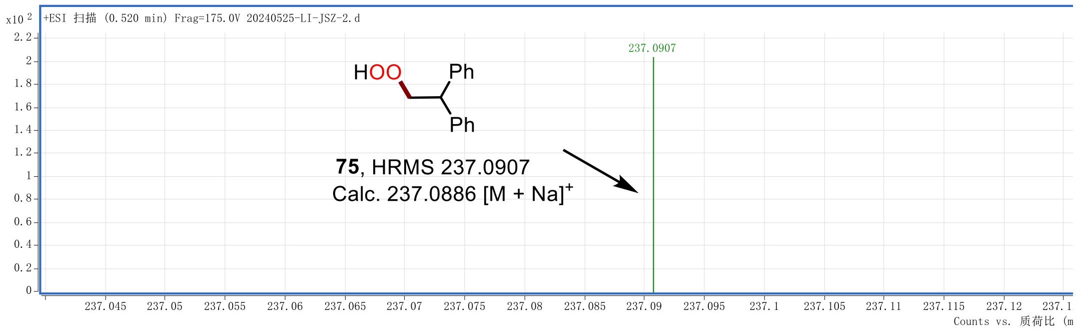

6.2 UV-vis absorption experiments.

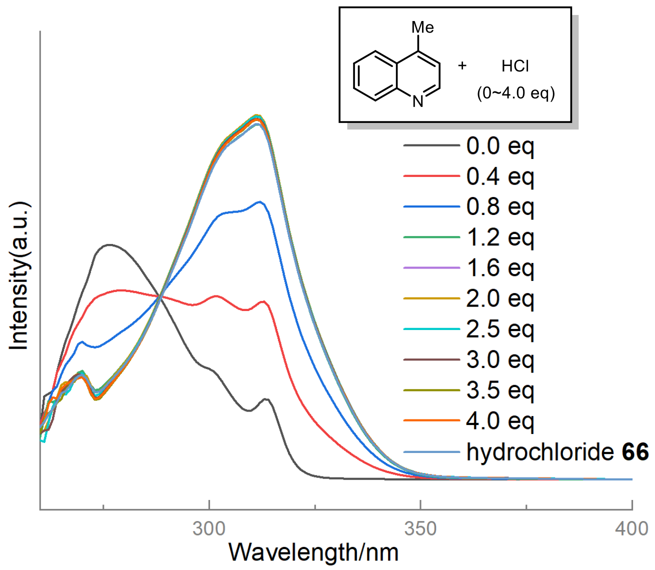

Figure S1. UV-vis absorption spectrum of 4-methylquinolines **1a** by HCl in MeCN.

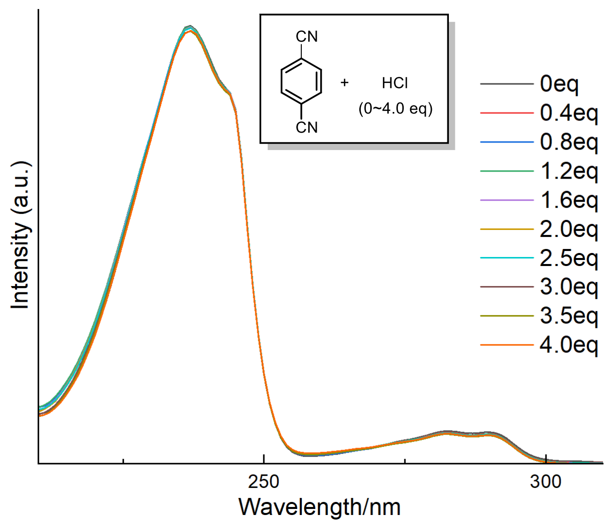

Figure S2. UV-vis absorption spectrum of *p*-phthalonitriles **44a** by HCl in MeCN.

6.2 Stern-Volmer quenching experiments.

Stern-Volmer quenching experiments were carried by Edinburgh Fluorescence Spectrometer, using a 0.1 M solution of 4-methylquinolines **1a** and different equivalent of HCl or trialkylsilane **2a** in MeCN. The samples were prepared in 4 mL quartz cuvettes. Stern-Volmer quenching experiments for each component are given in the Supplementary Figures below. This result showed that excited **1a-H** fluorescence was quenched more effectively by HCl than by trialkylsilane **2a** (Fig. S3 and S4).

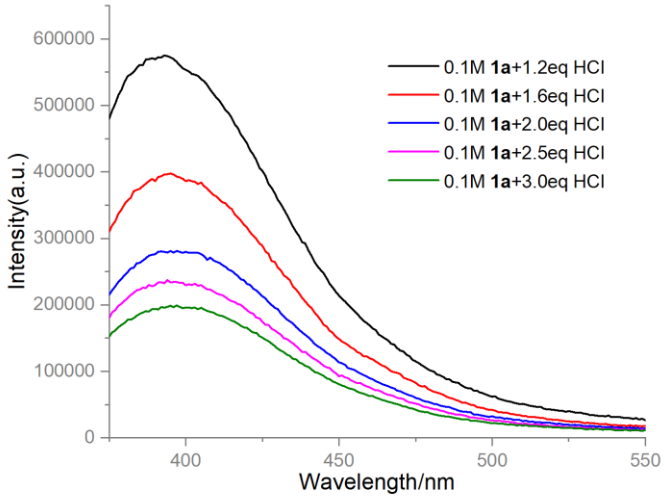

Figure S3. UV-vis absorption spectrum of 4-methylquinolines **1a** by HCl in MeCN.

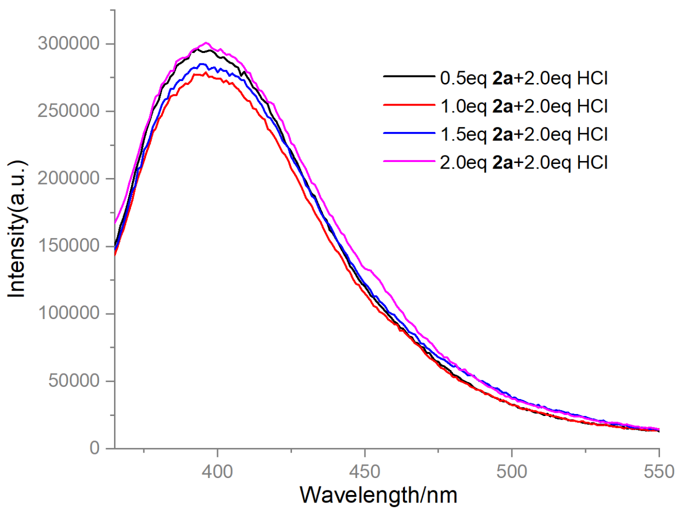

Figure S4. UV-vis absorption spectrum of 4-methylquinolines **1a** by trialkylsilane **2a** in MeCN.

Stern-Volmer quenching experiments were carried by Edinburgh Fluorescence Spectrometer, using a 0.1 M solution of *p*-phthalonitriles **44a** and different equivalent of HCl in MeCN. The samples were prepared in 4 mL quartz cuvettes. Stern-Volmer quenching experiments for each component are given in the Supplementary Figures below. This result showed that excited **44a** fluorescence was quenched by HCl (Fig. S5).

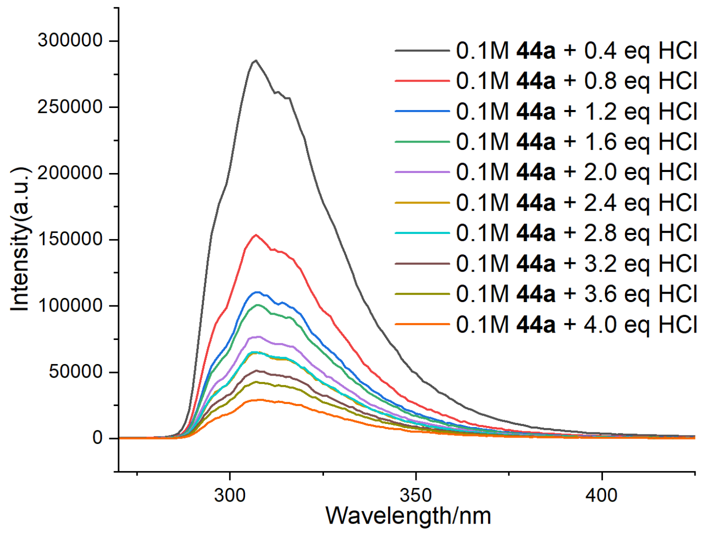

Figure S5. UV-vis absorption spectrum of *p*-phthalonitriles **44a** by HCl in MeCN.

Several mechanistic studies on the benzonitrile substrate, including UV-vis spectroscopy (Figure S2) and Stern-Volmer quenching experiments (Figure S5). The results revealed no significant shift in the UV-vis absorption spectrum of benzenedicarbonitrile upon addition of HCl. But stern-Volmer

quenching studies reveal a pronounced quenching effect of HCl on the excited state of p-phthalonitrile. These findings indicate that the cyano nitrogen atom possesses very weak basicity and is not protonated by HCl, but instead forms a weaker hydrogen-bonded complex. This weak, hydrogen-bonded EDA complex is less effective in promoting the reaction than the protonated quinoline species, rationalizing the higher efficiency of quinoline silylation under standard conditions.

6.3 Electron paramagnetic resonance (EPR) spectroscopy experiments

The electron paramagnetic resonance (EPR) was measured on Bruker EMXplus Spectrometer. According to the general procedure, 4-methylquinoline **1a** (28.6 mg, 0.2 mmol, 1.0 equiv.), *tert*-Butyldimethylsilane **2a** (116 mg, 1.0 mmol, 5.0 equiv.), HCl (12 mol/L, 33 μL, 0.4 mmol, 2.0 equiv.), 5,5-dimethyl-1-pyrroline *N*-oxide (22.6 mg, 0.2 mmol, 1.0 equiv.) and CH_3CN. The tube was sealed with a teflon-lined screw cap was irradiated by blue LEDs (λ = 365 nm) at 25 °C for 2 h in the air at room temperature. The resulting mixture was analyzed by EPR. When irradiating a mixture of quinoline **1a**, HCl, and DMPO with blue LEDs (λ = 365 nm), the production of $^1O_2$ was observed . When silane **2a** was added to the mixture of **1a**, HCl, and DMPO, a silyl radical and $^1O_2$ simultaneously appeared.

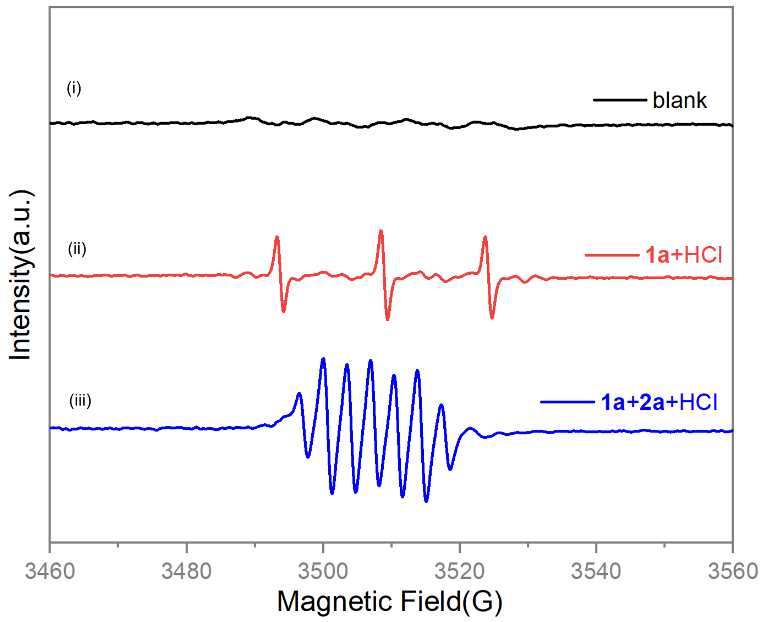

Figure S6. Electron paramagnetic resonance (EPR) spectroscopy experiments: i: the MeCN solution of DMPO in air atmosphere with blue light irradiation; ii: the MeCN solution of DMPO,

1a, and HCl in air atmosphere with blue light irradiation; iii: the MeCN solution of DMPO, **1a**, **2a**, and HCl in air atmosphere with blue light irradiation.

6.4 Mechanism study of the trifluoromethylation, alkylation, and acylation reactions

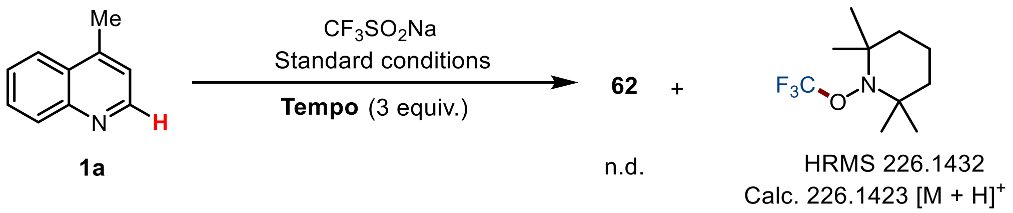

An oven-dried Schlenk tube (10 mL) was equipped with a magnetic stir bar, 4-methylquinoline (0.2 mmol, 1.0 equiv.), CF_3SO_2Na (0.6 mmol, 3.0 equiv.), HCl (0.4 mmol, 2.0 equiv.), TEMPO (0.6 mmol, 3.0 equiv), and acetonitrile (4.0 mL) were added. The reaction mixture was then stirred at room temperature under the irradiation of blue LEDs (λ = 390 nm). After the reaction, the mixture was analyzed by HR-MS.

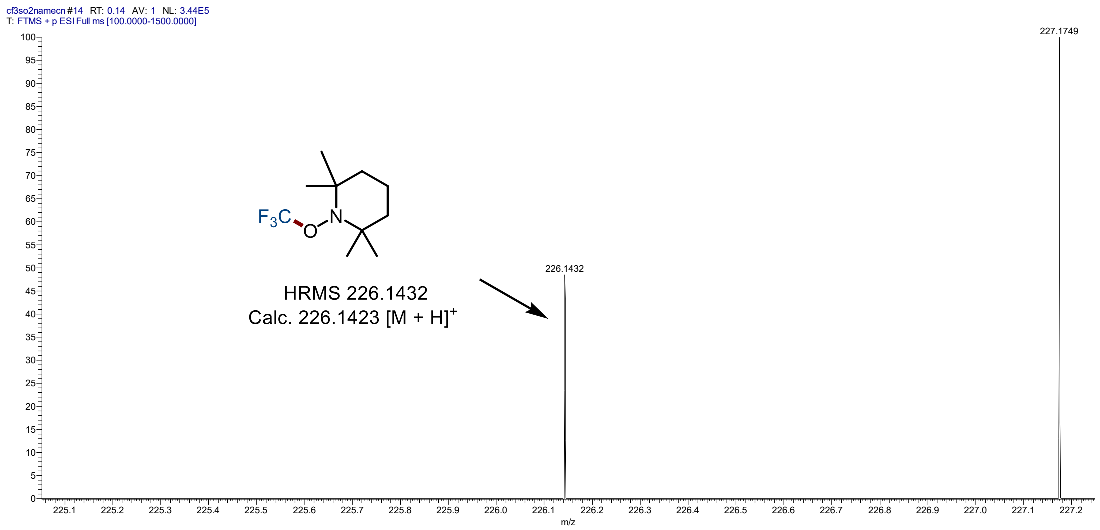

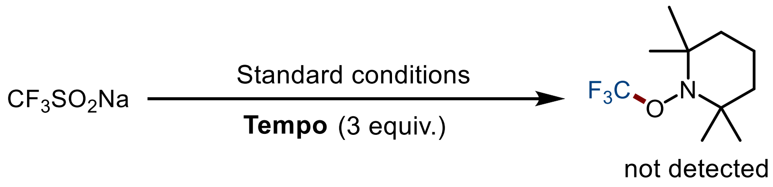

An oven-dried Schlenk tube (10 mL) was equipped with a magnetic stir bar, CF_3SO_2Na (0.2 mmol, 1.0 equiv.), HCl (0.4 mmol, 2.0 equiv.), TEMPO (0.6 mmol, 3.0 equiv), and acetonitrile (4.0 mL) were added. The reaction mixture was then stirred at room temperature under the irradiation of blue LEDs (λ = 390 nm). After the reaction, the mixture was analyzed by HR-MS.

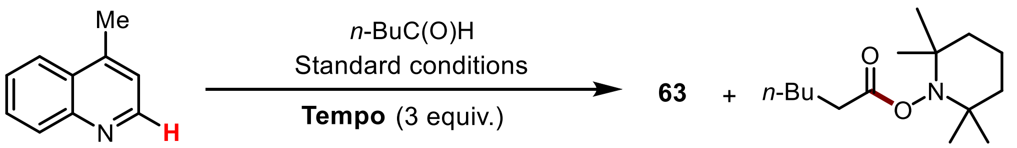

1a n.d. HRMS 242.2111
Calc. 242.2115 [M + H]$^+$

An oven-dried Schlenk tube (10 mL) was equipped with a magnetic stir bar, 4-methylquinoline (0.2 mmol, 1.0 equiv.), valeraldehyde (0.6 mmol, 3.0 equiv.), HCl (0.4 mmol, 2.0 equiv.), TEMPO (0.6 mmol, 3.0 equiv), and acetonitrile (4.0 mL) were added. The reaction mixture was then stirred at room temperature under the irradiation of blue LEDs (λ = 390 nm). After the reaction, the mixture was analyzed by HR-MS.

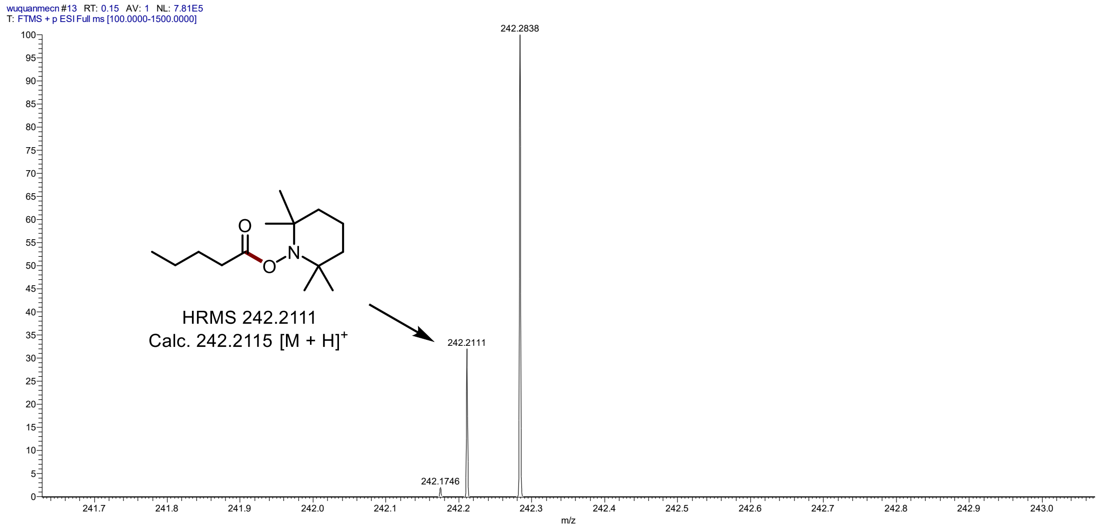

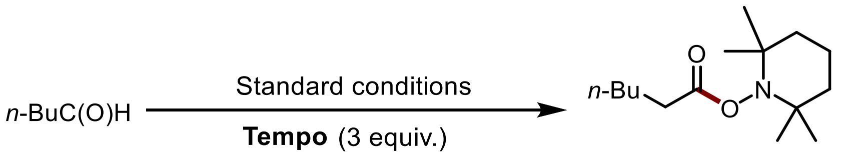

not detected

An oven-dried Schlenk tube (10 mL) was equipped with a magnetic stir bar, valeraldehyde (0.2 mmol, 1.0 equiv.), HCl (0.4 mmol, 2.0 equiv.), TEMPO (0.6 mmol, 3.0 equiv), and acetonitrile (4.0 mL) were added. The reaction mixture was then stirred at room temperature under the irradiation of blue LEDs (λ = 390 nm). After the reaction, the mixture was analyzed by HR-MS.

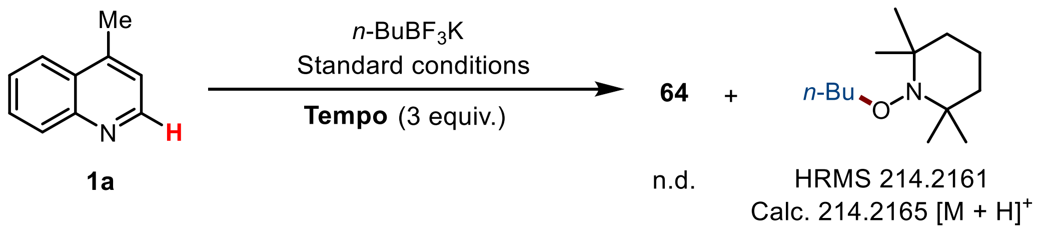

An oven-dried Schlenk tube (10 mL) was equipped with a magnetic stir bar, 4-methylquinoline (0.2 mmol, 1.0 equiv.), *n*-Butyl trifluoroborate (0.6 mmol, 3.0 equiv.), HCl (0.4 mmol, 2.0 equiv.), TEMPO (0.6 mmol, 3.0 equiv), and acetonitrile (4.0 mL) were added. The reaction mixture was then stirred at room temperature under the irradiation of blue LEDs (λ = 390 nm). After the reaction, the mixture was analyzed by HR-MS.

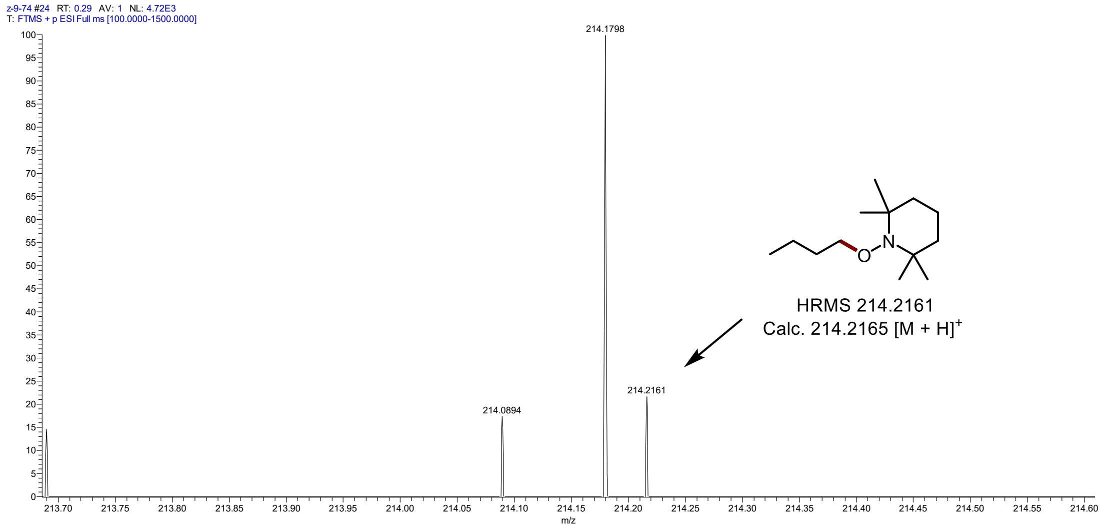

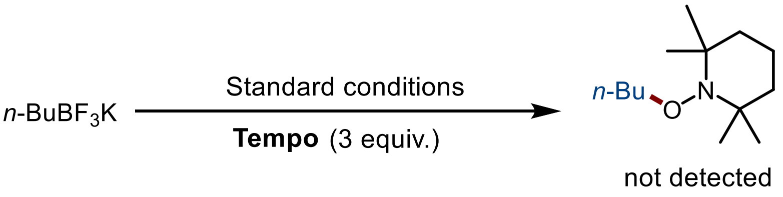

An oven-dried Schlenk tube (10 mL) was equipped with a magnetic stir bar, *n*-Butyl trifluoroborate (0.2 mmol, 1.0 equiv.), HCl (0.4 mmol, 2.0 equiv.), TEMPO (0.6 mmol, 3.0 equiv), and acetonitrile (4.0 mL) were added. The reaction mixture was then stirred at room temperature under the irradiation of blue LEDs (λ = 390 nm). After the reaction, the mixture was analyzed by HR-MS.

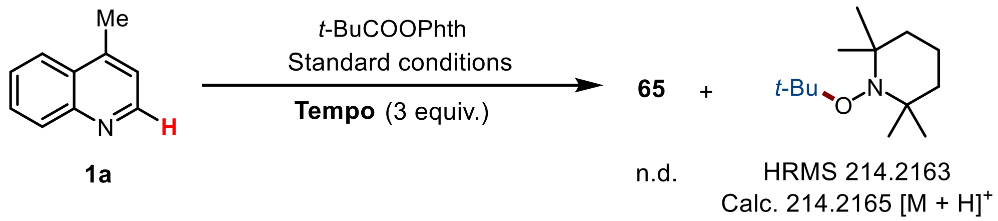

An oven-dried Schlenk tube (10 mL) was equipped with a magnetic stir bar, 4-methylquinoline (0.2 mmol, 1.0 equiv.), *t*-BuCOOPhth (0.6 mmol, 3.0 equiv.), HCl (0.4 mmol, 2.0 equiv.), TEMPO (0.6 mmol, 3.0 equiv), and acetonitrile (4.0 mL) were added. The reaction mixture was then stirred at room temperature under the irradiation of blue LEDs (λ = 390 nm). After the reaction, the mixture was analyzed by HR-MS.

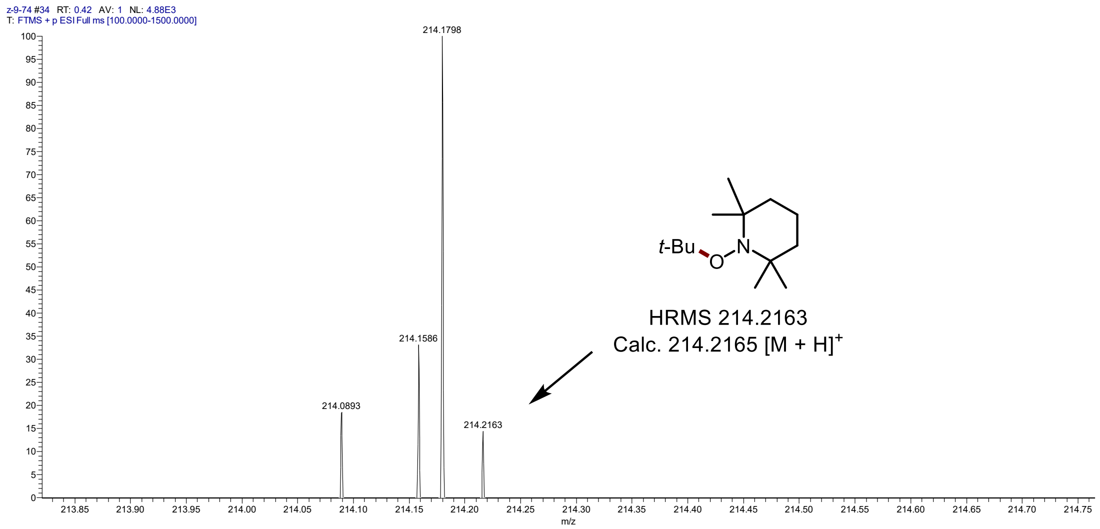

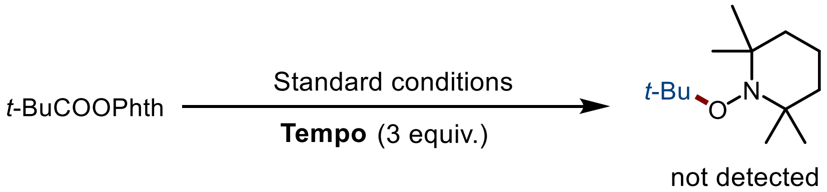

An oven-dried Schlenk tube (10 mL) was equipped with a magnetic stir bar, *t*-BuCOOPhth (0.2 mmol, 1.0 equiv.), HCl (0.4 mmol, 2.0 equiv.), TEMPO (0.6 mmol, 3.0 equiv), and acetonitrile (4.0 mL) were added. The reaction mixture was then stirred at room temperature under the irradiation of blue LEDs (λ = 390 nm). After the reaction, the mixture was analyzed by HR-MS.

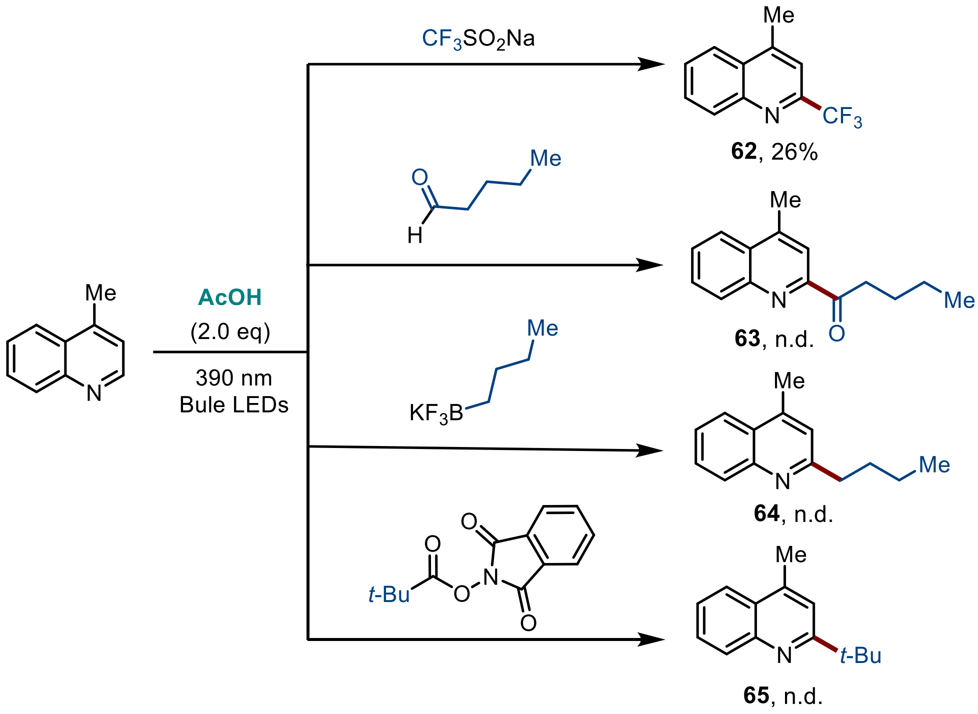

An oven-dried Schlenk tube (10 mL) was equipped with a magnetic stir bar, 4-methylquinoline (0.2 mmol, 1.0 equiv.), CF_3SO_2Na/*n*-BuC(O)H/*n*-$BuBF_3K$/*t*-BuCOOPhth (0.6 mmol, 3.0 equiv.), AcOH (0.4 mmol, 2.0 equiv.), and acetonitrile (4.0 mL) were added. The reaction mixture was then stirred at room temperature under the irradiation of blue LEDs (λ = 390 nm). After the reaction, the mixture was analyzed by GC-MS.

Introduction of TEMPO into the reaction mixtures led to the HRMS detection of trapped trifluoromethyl and alkyl radicals. Crucially, these radical species were only observed when quinoline hydrochloride was present, underscoring its role in the radical initiation step. Furthermore, replacing hydrochloric acid with acetic acid led to diminished efficiency in the trifluoromethylation of quinoline and completely inhibited the alkylation and acylation reactions. Experimental evidence confirmed that chlorine radicals are essential for generating the corresponding radical species from sodium trifluoromethanesulfonate, pentanal, boronic esters, and alkyl esters. We hypothesize that the subsequent functionalization steps follow a mechanism analogous to that proposed for quinoline silylation.

7. Synthetic Transformations of Products

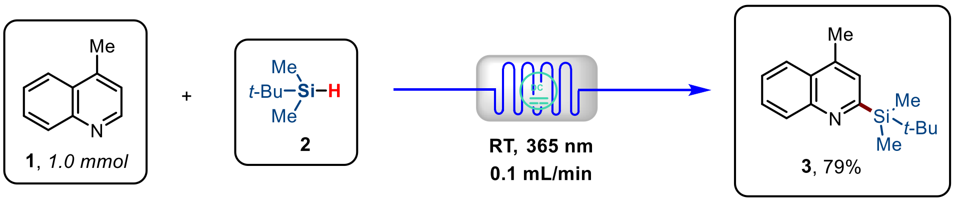

A 10 mL tube equipped with a magnetic stir bar was charged with (hetero)arenes **1a** (1.0 mmol, 1 equiv.), trialkylsilanes **2a** (5.0 mmol, 5.0 equiv.), and 2 mL of CH_3CN. HCl (12 mol/L, 2.5 mmol, 2.5 equiv.) in 2 mL of CH_3CN, was pumped to the flow reactor by a peristaltic pump with a flow speed of 0.1 mL/min. The mixture was irradiated by blue LEDs (20W, λ = 365 nm) in the air at room temperature for 8h. After reaction, the mixture was diluted with 1.0 mL of aqueous 1 M NaOH solution and extracted with ethyl acetate. Then the organic phase was combined and washed with brine and dried over anhydrous sodium sulfate. Upon removal of solvent under vacuum, the residue was purified by chromatography on silica gel (petroleum ether/ethyl acetate = 100:1–5:1) to give the desired product **3**.

Scaled-up reaction (10.0 mmol)

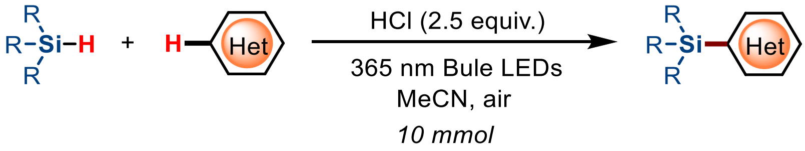

In a 500 mL oven-dried Schlenk tube, (hetero)arenes **1** (10.0 mmol, 1.0 equiv.), trialkylsilanes **2** (50.0 mmol, 5.0 equiv.) and HCl (12 mol/L, 25 mmol, 2.5 equiv.) were dissolved in MeCN (200 mL). The tube was positioned approximately 5 cm away from four 20 W blue LEDs lamps (λmax = 365 nm). The reaction mixture was stirred for 24 h. The solvent was removed under reduced pressure, and the residue was purified by column chromatography on silica gel affording the desired products.

4-methylquinoline-2-d (56)[1]

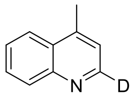

The title compound was prepared starting from 3a (26 mg, 0.10 mmol, 1.0 equiv.) and D_2O (8 mg, 0.40 mmol, 4.0 equiv.) were placed into a Schlenk flask under nitrogen in anhydrous

tetrahydrofuran (2.0 mL). Then anhydrous AgF (52 mg, 0.40 mmol, 4.0 equiv.) was added and the reaction mixture was stirred at 80 °C for 4 h. The solvent was removed in vacuo and the crude was directly poured into a flash column chromatography (petroleum ether : ethyl acetate 4:1) to give the title compound as a colorless oil (11.2 mg, 78% yield). **^{1}H NMR (400 MHz, $CDCl_3$) δ** 8.12 (d, *J* = 8.2 Hz, 1H), 8.03 – 7.98 (m, 1H), 7.75 – 7.68 (m, 1H), 7.61 – 7.54 (m, 1H), 7.24 (s, 1H), 2.72 (s, 3H). **^{13}C NMR**

(101 MHz, $CDCl_3$) δ 150.0, 147.8, 144.5, 129.9, 129.2, 128.3, 126.3, 123.8, 121.7, 18.6.

2-iodo-4-methylquinoline (57)[1]

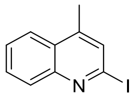

3a (26 mg, 0.10 mmol, 1.0 equiv.) and NIS (90 mg, 0.40 mmol, 4.0 equiv.) were placed into a Schlenk flask under nitrogen in anhydrous tetrahydrofuran (2.0 mL). Then anhydrous AgF (52 mg, 0.40 mmol, 4.0 equiv.) was added and the reaction mixture was stirred at 80 °C for 4 h. The solvent was removed in vacuo and the crude was directly poured into a flash column chromatography (petroleum ether : ethyl acetate 4:1) to give the title compound as a white solid (16.9 mg, 63% yield). **^{1}H NMR (400 MHz, $CDCl_3$) δ** 8.03 (d, *J* = 8.9 Hz, 1H), 7.93 (d, *J* = 9.2 Hz, 1H), 7.72 – 7.65 (m, 1H), 7.61 – 7.53 (m, 2H), 2.61 (s, 3H). **^{13}C NMR (101 MHz, $CDCl_3$) δ** 149.1, 145.8, 132.2, 129.9, 129.4, 127.3, 126.8, 124.0, 119.5, 18.0.

4-methyl-2-((trifluoromethyl)thio)quinoline (58)[1]

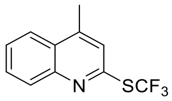

The title compound was prepared starting from 3a (26 mg, 0.10 mmol, 1.0 equiv.) and 1-((trifluoromethyl)thio)pyrrolidine-2,5-dione (80 mg, 0.40 mmol, 1.0 equiv.) and was obtained by eluting with petroleum ether : ethyl acetate 5:1 as a colorless oil (14.6 mg, 60% yield). ^{1}H NMR **(400 MHz, $CDCl_3$) δ** 8.09 (d, *J* = 8.3 Hz, 1H), 8.00 (d, *J* = 8.4 Hz, 1H), 7.75 (t, *J* = 7.7 Hz, 1H), 7.62 (t, *J* = 7.6 Hz, 1H), 7.45 (s, 1H), 2.73 (s, 3H). **^{13}C NMR (101 MHz, $CDCl_3$) δ** 149.5, 148.2,

146.5, 134.1, 131.0, 130.2, 129.9, 128.0, 127.5, 127.3, 124.8, 124.0, 124.0, 124.0, 124.0, 123.8, 123.4, 29.7, 27.8, 18.8. **^{19}F NMR (376 MHz, $CDCl_3$) δ** -39.32.

4-methylquinolin-2-ol (59)[1]

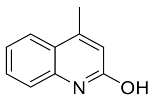

3a (26 mg, 0.10 mmol, 1.0 equiv.) and 30% H_2O_2 (0.25 mL) were placed into a flask in tetrahydrofuran (1.0 mL). The reaction mixture was stirred at room temperature for 4 h. The reaction was quenched with saturated $NaHSO_3$ aqueous solution (10 mL) and extracted with ethyl acetate (3×10 mL). Organic layers were combined, dried over anhydrous Na_2SO_4 and concentrated to obtain the compound as brown oil, which was purified by column chromatography (petroleum ether : ethyl acetate 4:1) to yield title compound as white solid (14.6 mg, 92% yield). **^{1}H NMR (400 MHz, $CDCl_3$) δ** 12.20 (s, 1H), 7.73 – 7.68 (m, 1H), 7.56 – 7.50 (m, 1H), 7.47 – 7.43 (m, 1H), 7.29 – 7.25 (m, 1H), 6.77 – 6.45 (m, 1H), 2.53 (s, 3H). **^{13}C NMR (101 MHz, $CDCl_3$) δ** 164.2, 149.3, 138.2, 130.5, 124.4, 122.5, 120.6, 120.5, 116.5, 19.1.

4-methyl-2-phenylquinoline (60)[1]

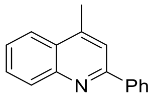

To a 10 mL vial was added **3a** (26 mg, 0.10 mmol, 1.0 equiv.), 4-iodobenzene (22 μL, 0.20 mmol, 2.0 equiv.), $Pd(Ph_3P)_4$ (6 mg, 0.005 mmol, 0.05 equiv.) and Ag_2O (23 mg, 0.10 mmol, 1.0 equiv.). The vial was sealed and placed under an atmosphere of nitrogen, then anhydrous DMF (2.0 mL) and TBAF (0.12 mL, 0.25 equiv., 1.0 mol/L in THF) were added. The reaction was heated at 90 ^{o} C for 2 hours. The reaction mixture was diluted with H_2O, extracted with ethyl acetate (3×10 mL), the combined organic extracts were washed with brine (10 mL), dried over anhydrous Na_2SO_4 and concentrated in vacuo. Purification of the crude product by flash chromatography on silica gel (petroleum ether : ethyl acetate 5:1) to afford the desired product as a white solid (11 mg, 50% yield).**^{1}H NMR (400 MHz, $CDCl_3$) δ** 8.22 (d, J = 8.4 Hz, 1H), 8.19 – 8.13 (m, 2H), 8.01 (d, J = 8.3 Hz, 1H), 7.76 – 7.70 (m, 2H), 7.59 – 7.50 (m, 3H), 7.50 – 7.43 (m, 1H), 2.78 (s, 3H). **^{13}C NMR (101 MHz, $CDCl_3$) δ** 157.1, 147.9, 145.1, 139.6, 130.1, 129.4, 129.3, 128.8, 127.6, 127.3, 126.1,

123.6, 119.8, 19.0.

4,4'-di-tert-butyl-2,2'-bipyridine (61)[1]

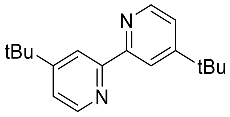

Add 4-(tert-butyl)-2-(tert-butyldimethylsilyl) pyridine (0.2 mmol, 1.0 equiv.), 2-bromo-4-(tertbutyl) pyridine (0.4 mmol, 2 equiv.), Pd $(Ph_3P)_4$ (0.1 mmol, 0.05 equiv.) and Ag_2O (0.2 mmol, 1 equiv.) to a 10 mL vial equipped with a Teflon septum and magnetic stir bar. Seal the vial and place under an atmosphere of nitrogen. Add then anhydrous DMF (2 mL, 0.1 M) and TBAF (0.1 mL, 0.5 equiv., 1 mmol/L in THF). Heat the reaction at 90 °C for 4 hours. Dilute the reaction mixture with H_2O and extract with ethyl acetate (3 × 10 mL). Wash the combined organic extracts with brine (15 mL). Dry over anhydrous Na_2SO_4 and concentrate in vacuo. Purify the crude product by flash chromatography on silica gel using the indicated solvent system (50% ethyl acetate/hexane) to give the title compound as a colorless solid (28.4 mg, 53% yield). **^{1}H NMR (400 MHz, $CDCl_3$) δ** 8.59 (d, J = 5.2 Hz, 2H), 8.45 – 8.34 (m, 2H), 7.31 – 7.28 (m, 2H), 1.39 (s, 18H). **^{13}C NMR (101 MHz, $CDCl_3$) δ** 161.0, 156.6, 149.0, 120.7, 118.3, 35.0, 30.6.

4-methyl-2-(trifluoromethyl)quinoline (62)[9]

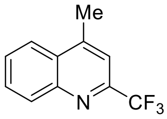

An oven-dried Schlenk tube (10 mL) was equipped with a magnetic stir bar, 4-methylquinoline (0.2 mmol, 1 equiv.), sodium trifluoromethanesulfinate (0.6 mmol, 3 equiv.), HCl (0.4 mmol, 2 equiv.) and acetonitrile (4.0 mL) were added. The reaction mixture was then stirred at room temperature under the irradiation of blue LEDs (λ = 390 nm). After reaction, the mixture was diluted with 1.0 mL of aqueous 1 M NaOH solution and extracted with ethyl acetate. Purification of the crude product by flash column chromatography afforded the product **62** (petroleum ether/ethyl acetate as eluent (50:1). white solid, 65%, 29.5 mg. **^{1}H NMR (400 MHz, $CDCl_3$) δ** 8.93 (d, J = 4.3 Hz, 1H), 8.22 (dd, J = 8.5, 1.4 Hz, 1H), 8.08 (d, J = 7.2 Hz, 1H), 7.61 (t, J = 7.9 Hz, 1H), 7.34 (dd, J = 4.3, 1.1 Hz, 1H), 2.74 (s, 3H).

1-(4-methylquinolin-2-yl)pentan-1-one (63)[10]

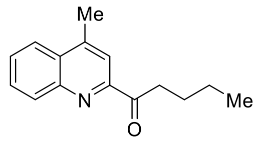

An oven-dried Schlenk tube (10 mL) was equipped with a magnetic stir bar, 4-methylquinoline (0.2 mmol, 1 equiv.), valeraldehyde (0.6 mmol, 3 equiv.), HCl (0.4 mmol, 2 equiv.) and acetonitrile (4.0 mL) were added. The reaction mixture was then stirred at room temperature under the irradiation of blue LEDs (λ = 390 nm). After reaction, the mixture was diluted with 1.0 mL of aqueous 1 M NaOH solution and extracted with ethyl acetate. Purification of the crude product by flash column chromatography afforded the product **63** (petroleum ether/ethyl acetate as eluent (50:1). white solid, 72%, 32.7 mg. **^{1}H NMR (400 MHz, $CDCl_3$) δ** 8.19 (d, J = 8.5 Hz, 1H), 8.02 (d, J = 8.4 Hz, 1H), 7.95 (s, 1H), 7.81 – 7.70 (m, 1H), 7.70 – 7.59 (m, 1H), 3.43 – 3.32 (m, 2H), 2.75 (s, 3H), 1.84 – 1.70 (m, 2H), 1.54 – 1.40 (m, 2H), 0.98 (t, J = 7.3 Hz, 3H). **^{13}C NMR (101 MHz, $CDCl_3$) δ** 203.2, 152.8, 147.0, 145.2, 131.1, 129.5, 128.1, 123.7, 118.7, 37.1, 26.4, 22.5, 18.8, 14.0.

2-butyl-4-methylquinoline (64)[11]

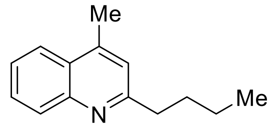

An oven-dried Schlenk tube (10 mL) was equipped with a magnetic stir bar, 4-methylquinoline (0.2 mmol, 1 equiv.), *n*-Butyl trifluoroborate (0.6 mmol, 3 equiv.), HCl (0.4 mmol, 2 equiv.) and acetonitrile (4.0 mL) were added. The reaction mixture was then stirred at room temperature under the irradiation of blue LEDs (λ = 390 nm). After reaction, the mixture was diluted with 1.0 mL of aqueous 1 M NaOH solution and extracted with ethyl acetate. Purification of the crude product by flash column chromatography afforded the product **64** (petroleum ether/ethyl acetate as eluent (50:1). white solid, 42%, 18.0 mg. **^{1}H NMR (400 MHz, $CDCl_3$) δ** 8.07 (d, J = 8.4 Hz, 1H), 7.95 (d, J = 8.2 Hz, 1H), 7.68 (t, J = 7.6 Hz, 1H), 7.52 (s, 1H), 7.16 (s, 1H), 2.97 – 2.92 (m, 2H), 2.69 (s, 3H), 1.78 (p, J = 7.8 Hz, 2H), 1.47 – 1.41 (m, 2H), 0.96 (t, J = 7.4 Hz, 3H).

2-(*tert*-butyl)-4-methylquinoline (65)[12]

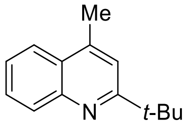

An oven-dried Schlenk tube (10 mL) was equipped with a magnetic stir bar, 4-methylquinoline (0.2 mmol, 1 equiv.), *t*-BuCOOPhth (0.6 mmol, 3 equiv.), HCl (0.4 mmol, 2 equiv.) and acetonitrile (2.0 mL) were added. The reaction mixture was then stirred at room temperature under the irradiation of blue LEDs (λ = 390 nm). After reaction, the mixture was diluted with 1.0 mL of aqueous 1 M NaOH solution and extracted with ethyl acetate. Purification of the crude product by flash column chromatography afforded the product **65** (petroleum ether/ethyl acetate as eluent (50:1). white solid, 63%, 25.2 mg. **^{1}H NMR (400 MHz, CDCl$_3$) δ** 8.07 (d, *J* = 8.3 Hz, 1H), 7.94 (d, *J* = 8.3 Hz, 1H), 7.69 – 7.64 (m, 1H), 7.53 – 7.47 (m, 1H), 7.36 (s, 1H), 2.70 (s, 3H), 1.47 (s, 9H).

4-((tert-butyldimethylsilyl)methyl)-2,4-dimethylisoquinoline-1,3(2H,4H)-dione (68)[6]

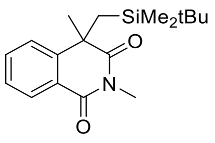

An oven-dried Schlenk tube (10 mL) was equipped with a magnetic stir bar, **66** (10 mol%), *N*-methacryloyl-*N*-methylbenzamide **67** (0.2 mmol, 1 equiv.), *t*BuMe$_2$SiH **2a** (1.0 mmol, 5 equiv., 116 mg), and acetonitrile (2.0 mL) were added. The reaction mixture was then stirred at room temperature under the irradiation of blue LEDs (λ = 365 nm). After the reaction, the mixture was diluted with 1.0 mL of aqueous 1 M NaOH solution and extracted with ethyl acetate. Purification of the crude product by flash column chromatography afforded the product **68** (petroleum ether/ethyl acetate as eluent (15:1). white solid, 51%, 32.3 mg. **^{1}H NMR (400 MHz, CDCl$_3$) δ** 8.29 – 8.20 (m, 1H), 7.66 – 7.58 (m, 1H), 7.49 – 7.37 (m, 2H), 3.37 (s, 3H), 1.84 (d, *J* = 14.6 Hz, 1H), 1.67 (s, 3H), 1.35 (d, *J* = 14.5 Hz, 1H), 0.78 (s, 9H), -0.37 (s, 3H), -0.78 (s, 3H). **^{13}C NMR (101 MHz, CDCl$_3$) δ** 177.0, 164.5, 144.9, 133.7, 128.8, 127.3, 126.2, 123.7, 45.2, 35.1, 27.2, 26.6, 26.1, 16.4, -5.7, -6.3.

tert-butyldimethyl(phenylethynyl)silane (70)

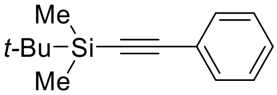

An oven-dried Schlenk tube (10 mL) was equipped with a magnetic stir bar, **66** (10 mol%), **69** (0.2 mmol, 1 equiv.), *t*BuMe$_2$SiH (1.0 mmol, 3 equiv.), and acetonitrile (2.0 mL) were added. The reaction mixture was then stirred at room temperature under the irradiation of blue LEDs (λ = 390 nm). After the reaction, the mixture was diluted with 1.0 mL of aqueous 1 M NaOH solution and extracted with ethyl acetate. Purification of the crude product by flash column chromatography afforded the product **70** (petroleum ether/ethyl acetate as eluent (50:1). white solid, 67%, 28.9 mg. **^{1}H NMR (400 MHz, CDCl$_3$) δ** 7.37 (s, 1H), 7.34 (s, 1H), 7.11 (s, 1H), 7.09 (s, 1H), 2.34 (s, 3H), 0.99 (s, 9H), 0.17 (s, 6H). **^{13}C NMR (101 MHz, CDCl$_3$) δ** 132.0, 128.4, 128.2, 123.3, 105.7, 92.4, 26.1, 16.7, -4.6.

1-(tert-butyl)-2-phenethyldisulfane (73)

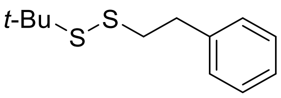

An oven-dried Schlenk tube (10 mL) was equipped with a magnetic stir bar, **66** (10 mol%), trifluoroborate **70** (0.2 mmol, 1 equiv.), disulfide **71** (0.2 mmol, 1 equiv., 40.6 mg), and acetonitrile (2.0 mL) were added. The reaction mixture was then stirred at room temperature under the irradiation of blue LEDs (λ = 390 nm). After the reaction, the mixture was diluted with 1.0 mL of aqueous 1 M NaOH solution and extracted with ethyl acetate. Purification of the crude product by flash column chromatography afforded the product **73** (petroleum ether/ethyl acetate as eluent (50:1). white solid, 39%, 17.6 mg. **^{1}H NMR (400 MHz, CDCl$_3$) δ** 7.35 – 7.14 (m, 5H), 2.99 – 2.88 (m, 4H), 1.33 (s, 12H). **^{13}C NMR (101 MHz, CDCl$_3$) δ** 140.2, 128.6, 128.5, 126.3, 47.9, 41.9, 35.8, 30.0.

8. DFT calculations

Computational Studies

Calculations were performed using the Gaussian 16, Revision A.03 package.[15] All structures were optimized at the B3LYP[16] level of theory in combination with Grimme's D3 dispersion corrections with the Becke-Johnson[17] damping scheme in combination with a def2-TZVP[18] basis set. Analytical frequency calculations were carried out at the same level of theory to identify the stationary points either as intermediates (no imaginary frequencies) or transition states (only one imaginary frequency), as well as to provide thermal and non-thermal corrections to the

free energy at 298.15 K and 1 atm. The electronic energy was then refined through B3LYP[16] single-point calculations on the optimized geometries in combination with a standalone version of Grimme's D4[19] dispersion corrections with a def2-QZVPP[18] basis set. Solvent effects were included in both optimization and single-point calculations through the use of an implicit solvation model SMD[20]. In the latter parameters for MeCN, the experimental solvent of choice, were included as implemented in Gaussian 16. Unless otherwise stated, the energies herein provided are based on solution Gibbs free energies with def2-TZVP basis set for which the electronic energies were improved at the B3LYP-D4/def2-QZVPP level of theory. Open-shell systems were calculated under unrestricted formalism.

To elucidate the regioselectivity of the direct C–H silylation process under the developed conditions, density functional theory (DFT) calculations were performed at the B3LYP-D4/def2-QZVPP+SMD(MeCN)//B3LYP-D3(BJ)/def2-TZVP+SMD (MeCN) level of theory using substrates **42a** and **2a** (Scheme 5). Initially, HCl associates with the nitrogen atom of the pyridine in **42a** to form the protonated complex **I-1**. This complex then undergoes intermolecular radical addition with silyl radical at the 5-position to give rise to intermediate **I-3**, with an energy barrier of 6.7 kcal mol^{-1}, 0.5 kcal mol^{-1} lower than that observed for the 7-position pathway. Subsequently, the desired silylated product **42** was obtained *via* deprotonation facilitated by $O_2^{-\bullet}$. The radical attack at the 5-position was proven to be kinetically and thermodynamically favored over the 7-position pathway.

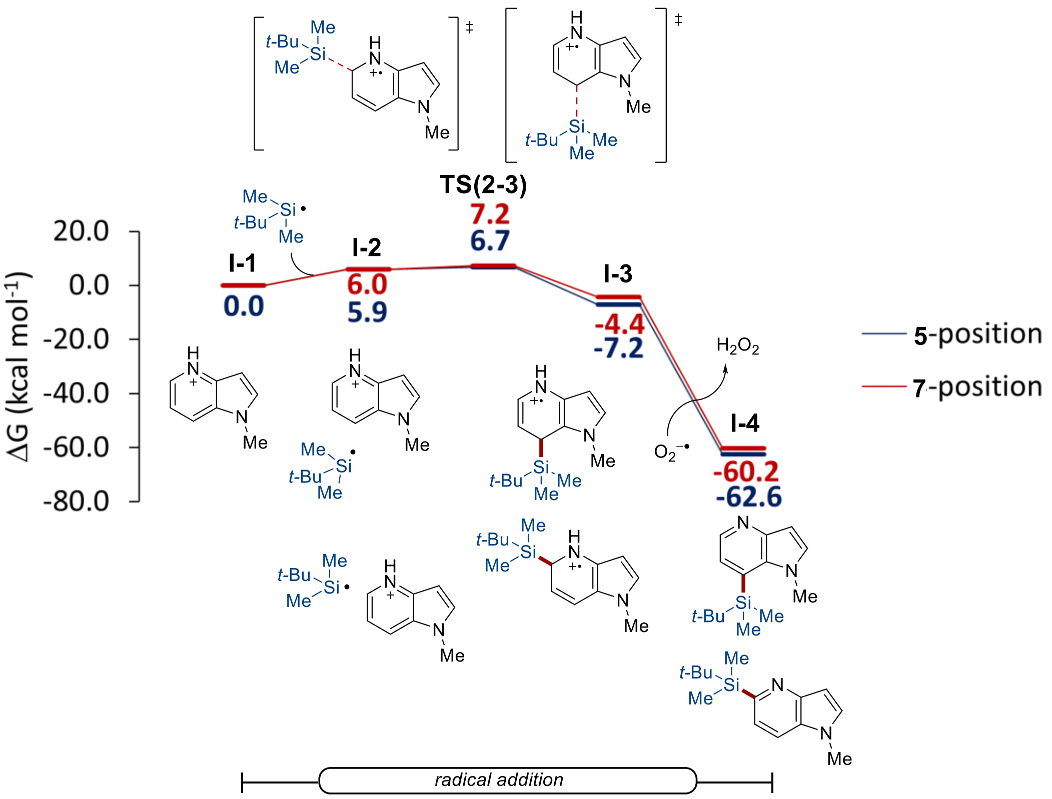

Figure S2. Computational studies for the regioselective silylation of substrate 42

Table S4. Calculated electronic energies at the B3LYP-D4/def2-QZVPP+SMD(MeCN) level of theory and Gibbs free energies with dispersion corrections for all structures in the present study (all in Hartree).[a]

Structure	Electronic energy	Total Gibbs Free Energy
**I-1**	-419.866286	-419.738896
**I-2**^{5}	-947.209800	-946.909872
**I-2**^{7}	-947.211932	-946.909797
**TS(2-3)**^{5}	-947.209763	-946.908626
**TS(2-3)**^{7}	-947.211928	-946.907794
**I-3**^{5}	-947.237780	-946.930751
**I-3**^{7}	-947.235029	-946.926271
**I-4**^{5}	-946.199953	-945.915530
**I-4**^{7}	-946.198807	-945.911710
**$^{\bullet}$Si(*t*-Bu)Me_2**	-527.336812	-527.180410
**$O_2^{-\bullet}$**	-150.518469	-150.535543
**H_2O_2**	-151.642865	-151.639072

[a] Superscripts correspond to the respective regioselectivity pathway.

Cartesian coordinates of the optimized structures

I-1

Lowest frequency = 90.9376 cm^{-1}

Charge = 1, Multiplicity = 1

19

C	1.390240	-0.911951	0.013150
C	-0.033551	-0.909634	-0.007163
C	0.003614	1.439422	-0.147891
C	1.385896	1.456098	-0.129303
C	2.108644	0.265601	-0.047476
C	0.663755	-3.007059	0.128835
C	-0.482763	-2.240572	0.066498
H	-1.684069	0.298524	-0.103206
H	-0.596944	2.333579	-0.210162
H	1.895139	2.406583	-0.179238
H	3.190082	0.271718	-0.032521
H	0.761175	-4.078461	0.194638
H	-1.498305	-2.596742	0.073538
N	-0.669269	0.278712	-0.087631
C	3.157170	-2.689351	0.145048
H	3.687861	-2.385511	-0.756919
H	3.662720	-2.278745	1.018728
H	3.150613	-3.773902	0.210167
N	1.781039	-2.223146	0.097531

I-2[5]

Lowest frequency = 16.3139 cm^{-1}

Charge = 1, Multiplicity = 2

41

C	1.045038	-1.085251	0.095199
C	-0.275344	-1.216555	0.605381
C	0.331787	0.133156	2.437708
C	1.627017	0.258143	1.960970
C	2.006789	-0.354852	0.770713
C	-0.151784	-2.318531	-1.312817
C	-1.025188	-1.998733	-0.291657
H	-1.514121	-0.677857	2.146725

H	2.330092	0.852036	2.524456
H	3.013954	-0.254069	0.390088
H	-0.328368	-2.902955	-2.201115
H	-2.058870	-2.287991	-0.212882
N	-0.574630	-0.601927	1.772077
C	2.244875	-1.908165	-1.949592
H	2.583342	-0.924467	-2.274352
H	3.052645	-2.411405	-1.418422
H	1.964528	-2.496549	-2.818847
N	1.081891	-1.777558	-1.087697
Si	-0.252197	3.183725	1.034952
C	-1.103536	2.155627	-0.296123
H	-2.005355	1.662611	0.070557
H	-1.389440	2.795289	-1.138780
H	-0.427541	1.388281	-0.677696
H	-0.002428	0.582378	3.358664
C	1.476438	3.659942	0.456061
H	2.005758	4.259823	1.198986
H	2.072447	2.767613	0.252057
H	1.420055	4.240989	-0.471218
C	-1.295350	4.728868	1.501829
C	-2.717882	4.298213	1.884277
C	-0.649113	5.464907	2.683155
C	-1.360066	5.675216	0.290446
H	-3.225734	3.796761	1.057192
H	-2.717578	3.617795	2.739952
H	-3.316855	5.174485	2.156730
H	0.366140	5.794391	2.449851
H	-1.234228	6.355618	2.938688
H	-0.600434	4.832831	3.573473
H	-1.968596	6.553638	0.536656
H	-0.369306	6.032083	0.000760
H	-1.814351	5.194662	-0.579197

I-2[7]

Lowest frequency = 18.1937 cm^{-1}

Charge = 1, Multiplicity = 2

41

C	0.511374	-1.063862	-0.786052
C	-0.269439	-1.073435	0.402139

C	-0.953339	1.129052	-0.069296
C	-0.194009	1.158296	-1.223664
C	0.565413	0.049857	-1.602048
C	0.788945	-3.013652	0.240960
C	-0.086573	-2.311724	1.045441
H	-1.535605	0.048484	1.557910
H	-0.193111	2.056295	-1.822437
H	1.188060	-4.006006	0.373247
H	-0.523542	-2.647964	1.969684
N	-0.977637	0.035199	0.710497
C	2.030239	-2.700080	-1.924494
H	2.837886	-1.978617	-2.045568
H	2.448669	-3.668909	-1.665845
H	1.474998	-2.783229	-2.859038
N	1.147080	-2.275836	-0.851707
Si	3.666875	0.545610	0.084907
C	4.258562	2.258863	-0.435667
H	3.410426	2.882658	-0.729376
H	4.766432	2.759285	0.396507
H	4.951971	2.214029	-1.277776
C	2.504357	0.705586	1.558956
H	2.109090	-0.259915	1.876710
H	3.036766	1.148805	2.408091
H	1.662715	1.358762	1.319877
C	5.139157	-0.621747	0.493612
C	4.614402	-1.983134	0.969755
C	6.016161	-0.821240	-0.749597
C	5.983565	0.014020	1.611942
H	3.992774	-1.890650	1.862991
H	4.021043	-2.478525	0.198887
H	5.453424	-2.642932	1.218246
H	6.421401	0.124544	-1.116967
H	6.864483	-1.473452	-0.512764
H	5.457597	-1.286330	-1.565676
H	6.824137	-0.643863	1.863408
H	6.399288	0.977796	1.309624
H	5.402496	0.167644	2.524199
H	-1.551253	1.962884	0.264395
H	1.162129	0.064115	-2.502968

TS(2-3)[5]

Lowest frequency = -32.1660 cm^{-1}

Charge = 1, Multiplicity = 2

41

C	1.004047	-1.042100	0.115303
C	-0.320417	-1.092097	0.621892
C	0.315514	0.381094	2.350285
C	1.624124	0.415470	1.879948
C	1.990921	-0.299559	0.748003
C	-0.223311	-2.337574	-1.209621
C	-1.090600	-1.914290	-0.220837
H	-1.548730	-0.411686	2.118512
H	2.342910	1.025487	2.405341
H	3.005010	-0.268224	0.374110
H	-0.413010	-2.979976	-2.053933
H	-2.133753	-2.163154	-0.128702
N	-0.607010	-0.388367	1.743882
C	2.189704	-2.054231	-1.850920
H	2.562938	-1.110177	-2.247470
H	2.977267	-2.540743	-1.275553
H	1.896547	-2.698830	-2.675014
N	1.025878	-1.820731	-1.012856
Si	-0.158276	3.154851	0.913800
C	-0.934368	2.225548	-0.527184
H	-1.862960	1.727028	-0.244816
H	-1.159956	2.919656	-1.344695
H	-0.246150	1.470735	-0.912275
H	0.001403	0.884602	3.249369
C	1.563560	3.754111	0.449944
H	2.062711	4.249580	1.284809
H	2.186149	2.915638	0.129776
H	1.508989	4.464799	-0.382193
C	-1.284154	4.578036	1.541218
C	-2.673538	4.025699	1.888519
C	-0.666456	5.228404	2.786536
C	-1.421031	5.635784	0.431708
H	-3.159793	3.575561	1.020080
H	-2.621664	3.268114	2.674843
H	-3.320588	4.833766	2.247554
H	0.320866	5.647136	2.578345
H	-1.304221	6.046605	3.139364
H	-0.561563	4.511997	3.605299

H	-2.072269	6.449223	0.773215
H	-0.456474	6.075164	0.167920
H	-1.863746	5.219867	-0.476295

TS(2-3)[7]

Lowest frequency = -12.9544 cm^{-1}

Charge = 1, Multiplicity = 2

41

C	0.579017	-1.094539	-0.786136
C	-0.286332	-1.103323	0.339982
C	-0.813144	1.151351	-0.088428
C	0.031168	1.181207	-1.180566
C	0.763459	0.047715	-1.545119
C	0.672189	-3.095421	0.174611
C	-0.221235	-2.374334	0.941741
H	-1.573265	0.046130	1.447599
H	0.125370	2.099416	-1.739918
H	1.003895	-4.114111	0.291913
H	-0.744854	-2.719308	1.816531
N	-0.954580	0.032819	0.644021
C	2.086598	-2.772498	-1.879959
H	2.906862	-2.060019	-1.958835
H	2.481856	-3.743949	-1.596396
H	1.582167	-2.852657	-2.843353
N	1.152616	-2.336790	-0.855631
Si	3.600905	0.589286	0.148820
C	4.148294	2.322518	-0.348456
H	3.286097	2.927315	-0.640695
H	4.638190	2.826847	0.491921
H	4.847519	2.303567	-1.186890
C	2.485100	0.679795	1.662734
H	2.055729	-0.290536	1.915121
H	3.057178	1.030626	2.529141
H	1.667450	1.384301	1.498095
C	5.096864	-0.578437	0.456081
C	4.611299	-1.899552	1.067678
C	5.826888	-0.864885	-0.863119
C	6.068989	0.104729	1.434277
H	4.128921	-1.744846	2.035058
H	3.898633	-2.410165	0.416746

H	5.460039	-2.574474	1.225241
H	6.179456	0.052508	-1.340883
H	6.702143	-1.498587	-0.680223
H	5.181063	-1.386404	-1.573602
H	6.913514	-0.562278	1.645140
H	6.475667	1.030736	1.022259
H	5.590806	0.340306	2.388166
H	-1.390141	2.004587	0.232952
H	1.415015	0.058038	-2.406572

I-3[5]

Lowest frequency = 31.4523 cm^{-1}

Charge = 1, Multiplicity = 2

41

C	1.189970	-0.340933	0.136739
C	-0.166822	-0.502419	-0.264292
C	-0.818843	1.267919	1.277054
C	0.562083	1.246672	1.773763
C	1.550969	0.510692	1.203463
C	1.106944	-1.851270	-1.494101
C	-0.201743	-1.467445	-1.296315
H	-2.092906	0.067721	0.078053
H	0.778911	1.893823	2.610950
H	2.568300	0.555321	1.566577
H	1.518878	-2.559625	-2.193687
H	-1.066072	-1.827958	-1.827297
N	-1.129574	0.196653	0.355374
C	3.382658	-1.330460	-0.554886
H	3.876493	-0.389832	-0.798085
H	3.673675	-1.642877	0.447674
H	3.685332	-2.091287	-1.269097
N	1.939268	-1.176045	-0.632627
Si	-1.184382	2.978148	0.327301
C	-2.981667	2.844666	-0.175384
H	-3.641405	2.820784	0.694861
H	-3.263503	3.703022	-0.789531
H	-3.166370	1.945344	-0.767500
H	-1.545442	1.270267	2.094160
C	-0.067286	3.002646	-1.167896
H	0.986031	2.928594	-0.893666

H	-0.304296	2.179321	-1.845110
H	-0.206452	3.936072	-1.719926
C	-0.897551	4.456470	1.489572
C	-1.447640	4.159803	2.891758
C	0.591722	4.817792	1.580244
C	-1.656265	5.659938	0.899656
H	-2.500289	3.867547	2.864187
H	-0.889741	3.364300	3.389275
H	-1.371836	5.055319	3.517825
H	0.999260	5.094102	0.605765
H	0.721197	5.676745	2.247551
H	1.197300	4.001774	1.976904
H	-1.458415	6.549268	1.507409
H	-1.340417	5.884598	-0.122371
H	-2.735309	5.497695	0.891837

I-3[7]

Lowest frequency = 28.9102 cm^{-1}

Charge = 1, Multiplicity = 2

41

C	1.418520	-0.999675	-0.080379
C	0.299638	-0.860187	0.774729
C	0.166953	1.444229	0.205797
C	1.249777	1.352957	-0.596718
C	2.118872	0.156987	-0.625235
C	0.752811	-3.022390	0.598763
C	-0.095812	-2.142123	1.217922
H	-1.074101	0.465261	1.538389
H	1.532952	2.214354	-1.180399
H	0.772036	-4.098505	0.628688
H	-0.905971	-2.392369	1.880877
N	-0.256068	0.370070	0.955717
C	2.520240	-2.983889	-1.166946
H	3.329752	-2.321750	-1.450454
H	2.946645	-3.886328	-0.734784
H	1.941997	-3.245512	-2.053789
N	1.665880	-2.332315	-0.183042
Si	3.659071	0.754440	0.602654
C	4.048176	2.498269	0.048833
H	3.231388	3.185617	0.266936

H	4.927301	2.859174	0.584768
H	4.269785	2.557104	-1.018510
C	2.964476	0.714394	2.334531
H	2.674035	-0.290488	2.641579
H	3.722904	1.067931	3.036217
H	2.100359	1.371170	2.436948
C	5.186714	-0.363418	0.422652
C	4.978373	-1.724359	1.103559
C	5.588959	-0.535739	-1.048209
C	6.338347	0.360109	1.155820
H	4.818498	-1.611989	2.176613
H	4.131221	-2.277462	0.704164
H	5.868729	-2.344986	0.970881
H	5.778461	0.424654	-1.531144
H	6.509468	-1.121296	-1.119331
H	4.833594	-1.055723	-1.638989
H	7.220998	-0.285274	1.157817
H	6.618990	1.292546	0.666842
H	6.097319	0.577880	2.198199
H	-0.425834	2.341727	0.291736
H	2.614324	-0.001725	-1.583459

I-4[5]

Lowest frequency = 2.8806 cm^{-1}

Charge = 0, Multiplicity = 1

39

C	1.369239	-0.975656	0.045686
C	0.003349	-0.777362	0.403455
C	0.078435	1.418666	-0.282975
C	1.431005	1.273987	-0.652870
C	2.102510	0.069047	-0.494172
C	0.586317	-2.894248	0.864750
C	-0.470992	-2.022415	0.924664
H	1.963650	2.115406	-1.069959
H	3.141214	-0.042247	-0.778175
H	0.645223	-3.929720	1.159578
H	-1.460047	-2.240714	1.293063
N	-0.622857	0.394871	0.240786
C	2.993759	-2.884929	0.125751
H	3.262163	-2.856789	-0.931537

H	3.761200	-2.363693	0.700240
H	2.949638	-3.921110	0.452363
N	1.696520	-2.274294	0.337957
Si	-0.973677	2.993222	-0.436914
C	-2.445736	2.559521	-1.520870
H	-2.974659	1.700070	-1.102176
H	-3.152759	3.390022	-1.585420
H	-2.135770	2.298839	-2.535538
C	-1.585938	3.408826	1.290057
H	-0.760358	3.657340	1.961058
H	-2.277445	4.254638	1.275963
H	-2.113526	2.550825	1.713384
C	-0.042601	4.489198	-1.173708
C	1.148444	4.890463	-0.290894
C	0.442107	4.192520	-2.600595
C	-1.019059	5.677126	-1.234063
H	0.829278	5.146249	0.722020
H	1.895866	4.099541	-0.212900
H	1.646087	5.772433	-0.710553
H	-0.389447	3.941957	-3.263185
H	0.940204	5.074995	-3.018649
H	1.154686	3.367165	-2.636645
H	-0.512173	6.553440	-1.653844
H	-1.883356	5.464064	-1.866774
H	-1.387117	5.954419	-0.243926

I-4[7]

Lowest frequency = 37.3568 cm^{-1}

Charge = 0, Multiplicity = 1

39

C	1.789866	-0.950082	0.299087
C	0.497549	-0.759387	0.898052
C	0.195368	1.190426	-0.202947
C	1.437711	1.072252	-0.831472
C	2.298319	-0.015557	-0.620448
C	1.353905	-2.665547	1.682320
C	0.249378	-1.864489	1.762625
H	1.718457	1.859278	-1.515658
H	1.574550	-3.592831	2.185904
H	-0.630975	-2.035067	2.360268

N	-0.292052	0.294431	0.647269
C	3.591719	-2.721599	0.629126
H	4.363190	-2.078259	1.049426
H	3.610856	-3.677393	1.147660
H	3.798183	-2.898425	-0.421744
N	2.278140	-2.137677	0.811245
Si	3.836496	-0.010676	-1.755006
C	4.162887	1.780043	-2.230117
H	4.285909	2.401245	-1.338974
H	5.096312	1.830262	-2.795878
H	3.380014	2.224042	-2.845598
C	5.443359	-0.609144	-0.985744
H	5.567837	-1.690096	-0.993153
H	6.266342	-0.176903	-1.561033
H	5.545629	-0.258529	0.043569
C	3.412002	-1.012832	-3.327541
C	3.148839	-2.486683	-2.995907
C	2.163995	-0.428161	-4.003413
C	4.599414	-0.923564	-4.299211
H	4.030748	-2.971293	-2.571926
H	2.322337	-2.605049	-2.291442
H	2.884483	-3.033607	-3.908093
H	2.296425	0.622505	-4.270250
H	1.948150	-0.977833	-4.926527
H	1.283868	-0.502953	-3.361714
H	4.382220	-1.494246	-5.209201
H	4.804043	0.106564	-4.599045
H	5.512872	-1.335333	-3.863725
H	-0.427511	2.054391	-0.411392

•Si(*t*-Bu)Me₂

Lowest frequency = 20.5288 cm^{-1}

Charge = 0, Multiplicity = 2

22

Si	-0.082186	-0.206900	0.016006
C	0.577935	0.663744	-1.563744
C	2.112295	0.657997	-1.564328
C	0.064650	-0.059878	-2.815625
C	0.076950	2.118211	-1.578349

H	2.520518	1.182510	-0.697377
H	2.512015	-0.359338	-1.559692
H	2.491068	1.159942	-2.461947
H	-1.026644	-0.061067	-2.865024
H	0.435116	0.439146	-3.718326
H	0.403026	-1.098720	-2.848465
H	0.445791	2.629883	-2.475458
H	-1.013942	2.173125	-1.594325
H	0.431444	2.679853	-0.711047
C	0.547765	0.639556	1.579142
H	0.190612	0.117123	2.470128
H	1.638284	0.668515	1.619275
H	0.178689	1.670324	1.627902
C	-1.967603	-0.242169	0.042138
H	-2.381102	-0.740454	-0.836731
H	-2.330300	-0.766512	0.929758
H	-2.366141	0.778272	0.072862

$O_2^{-\bullet}$

Lowest frequency = 1192.4573 cm^{-1}

Charge = -1, Multiplicity = 2

2

O	1.101202	-0.277068	0.000000
O	2.445417	-0.277068	0.000000

H_2O_2

Lowest frequency = 373.6827 cm^{-1}

Charge = 0, Multiplicity = 1

4

O	1.050095	-0.245457	-0.295485
H	0.890952	0.510040	0.295496
O	2.496523	-0.308738	-0.295483
H	2.655668	-1.064118	0.29564

9. NMR Spectra

^{1}H NMR (400 MHz, $CDCl_3$) of 3

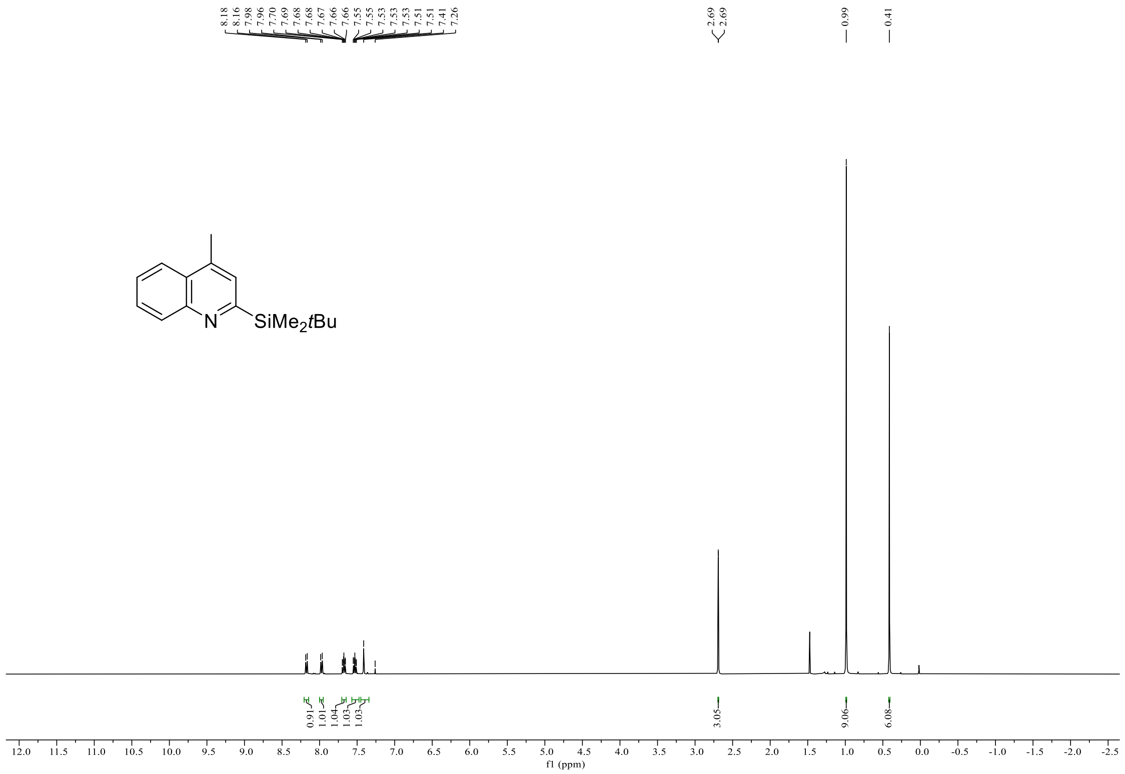

^{13}C NMR (101 MHz, $CDCl_3$) of 3

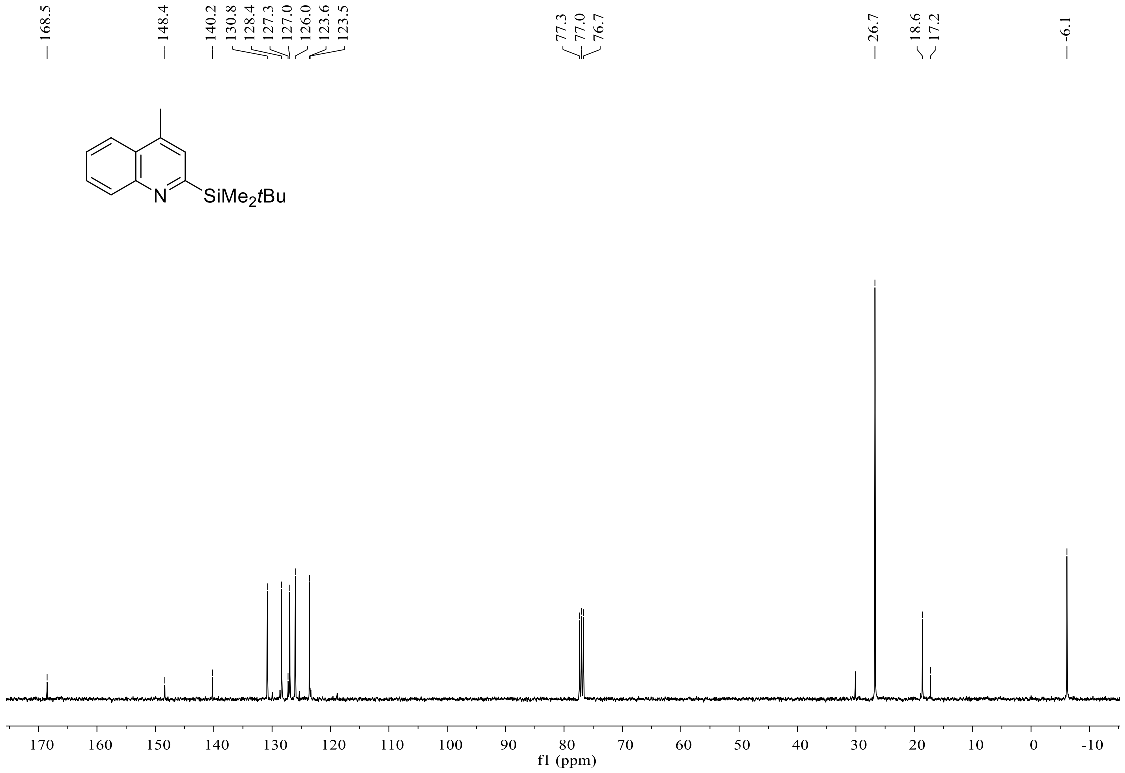

^{1}H NMR (400 MHz, $CDCl_3$) of 4

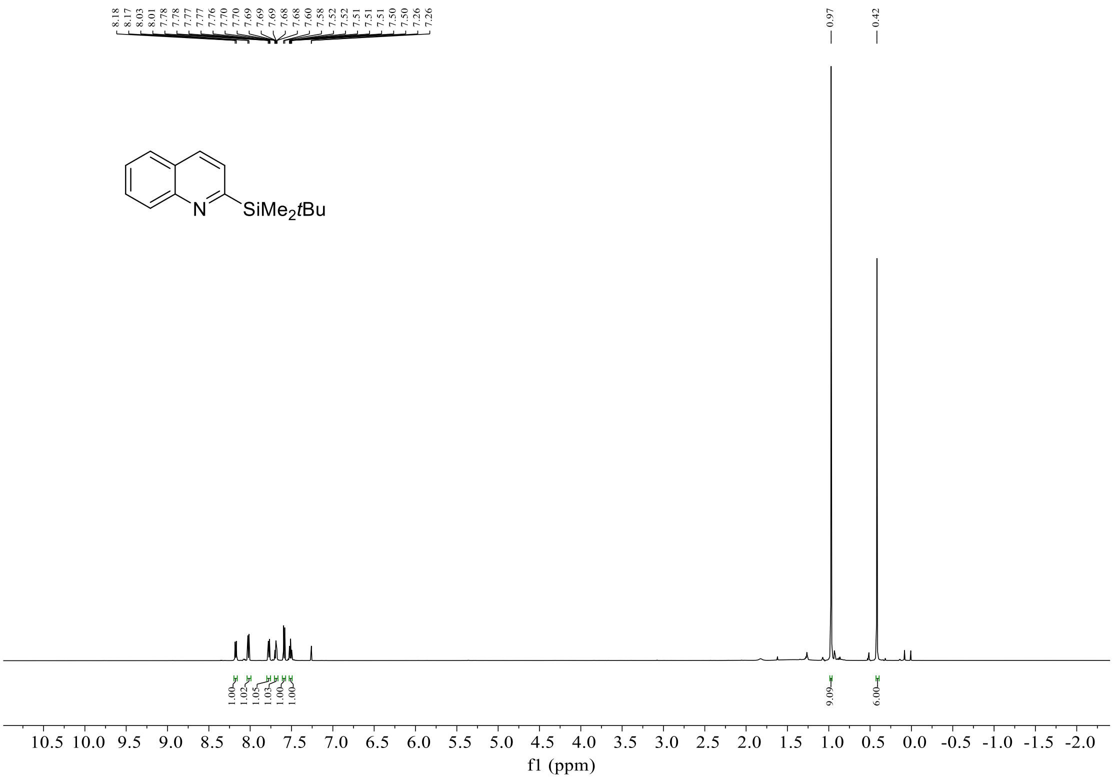

^{13}C NMR (101 MHz, $CDCl_3$) of 4

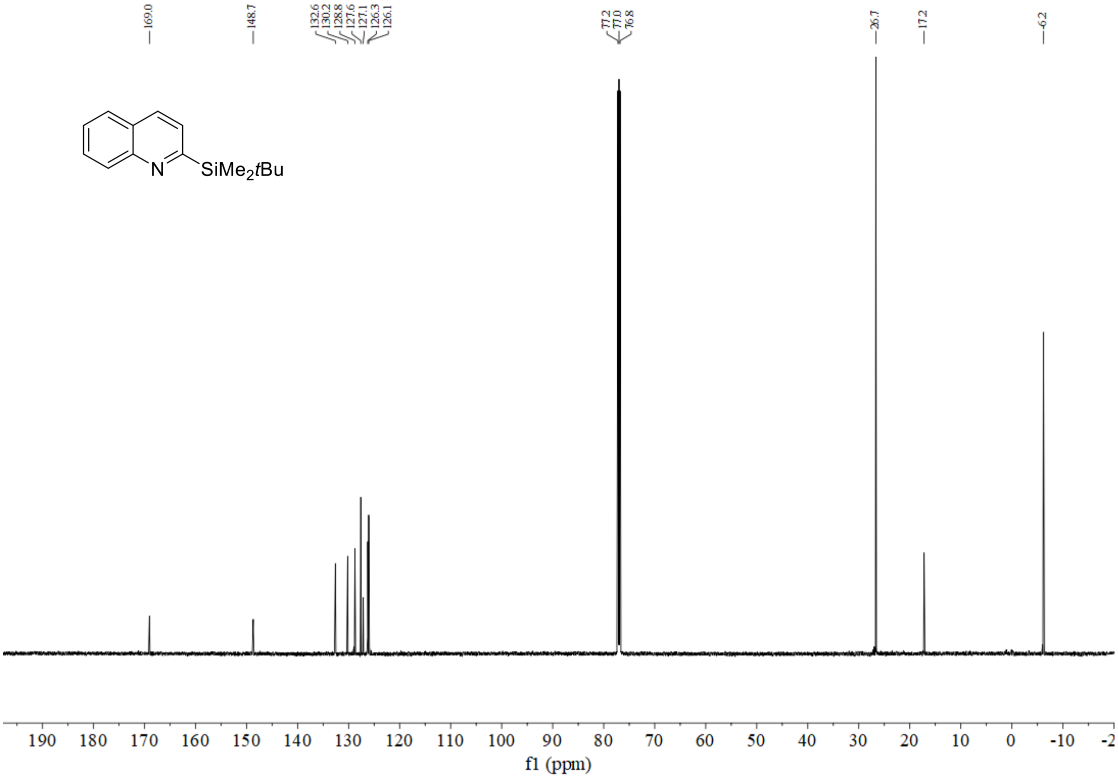

^{1}H NMR (400 MHz, $CDCl_3$) of 5

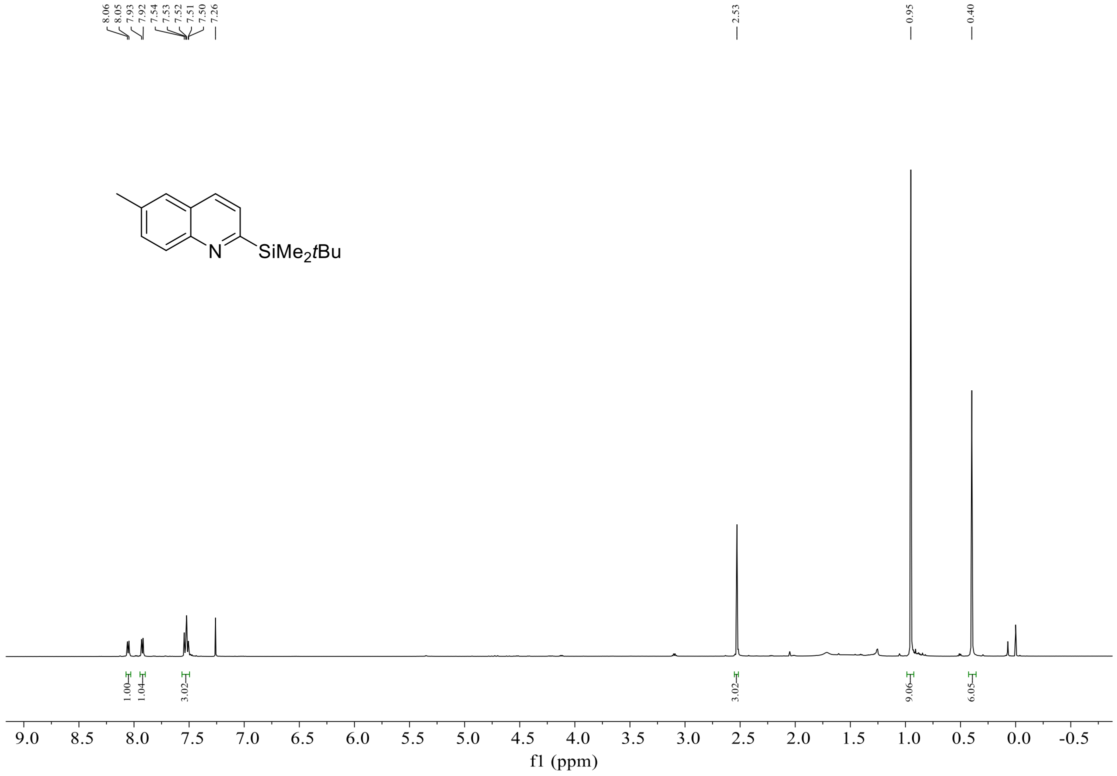

^{13}C NMR (101 MHz, $CDCl_3$) of 5

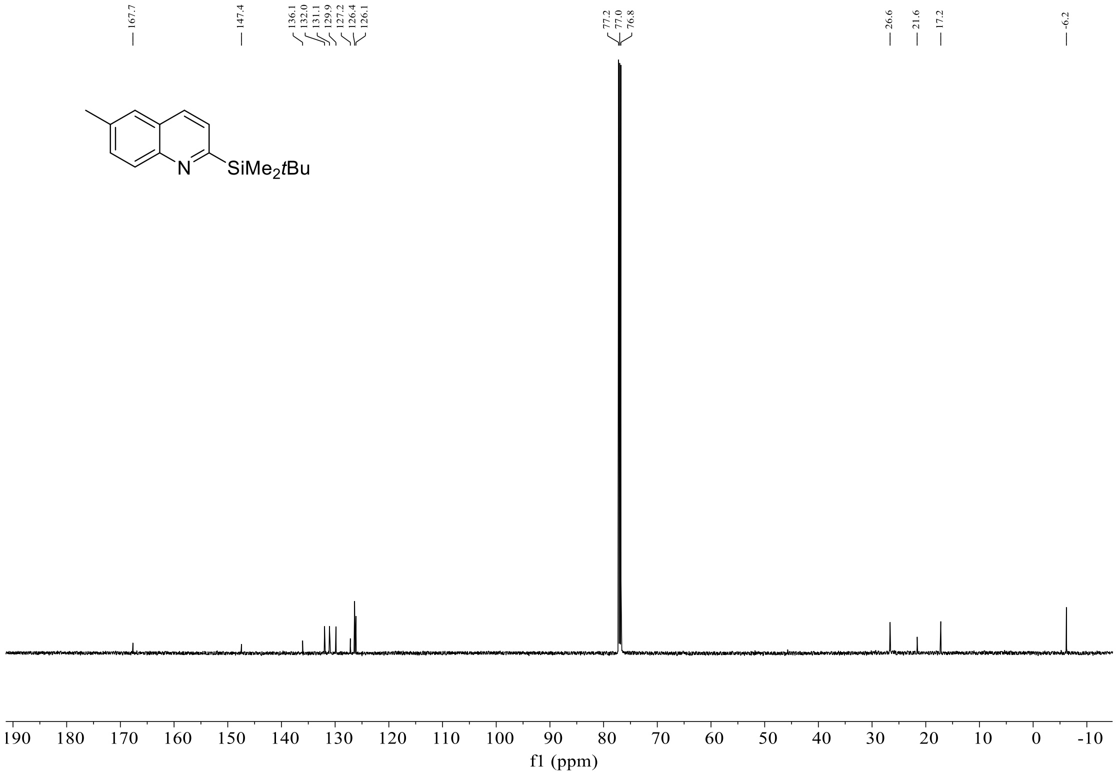

^{1}H NMR (400 MHz, $CDCl_3$) of 6

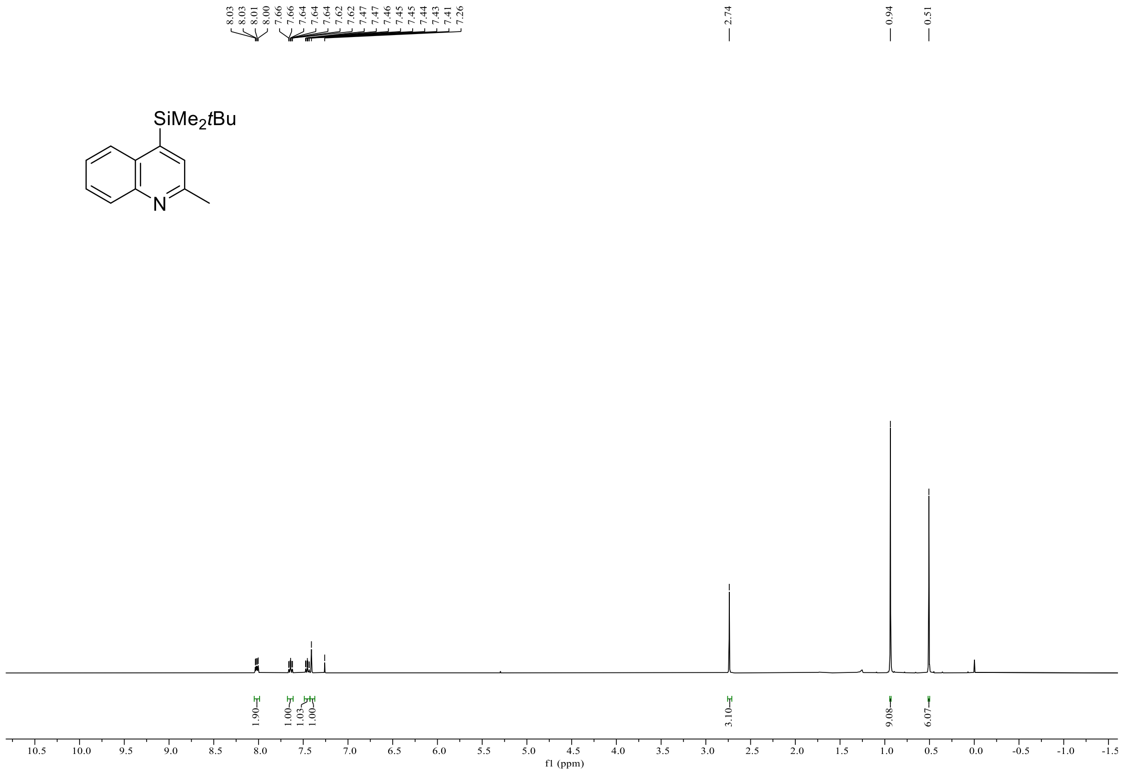

^{13}C NMR (101 MHz, $CDCl_3$) of 6

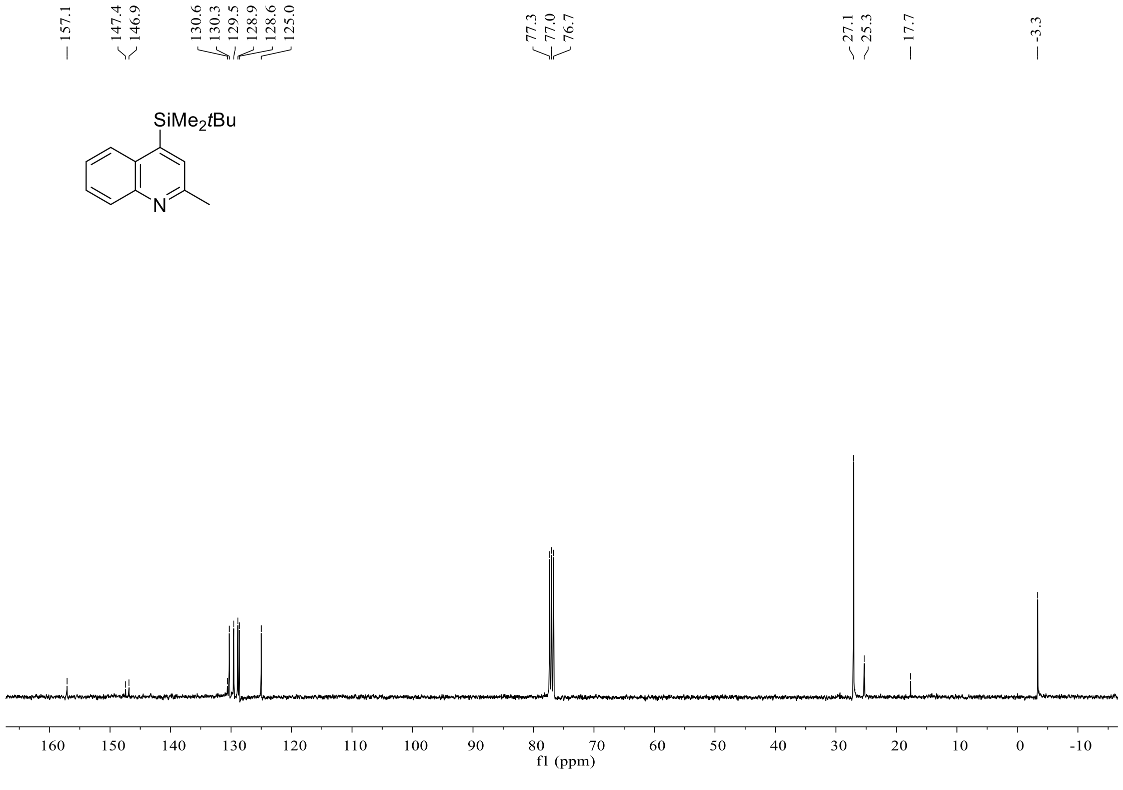

^{1}H NMR (400 MHz, $CDCl_3$) of 7

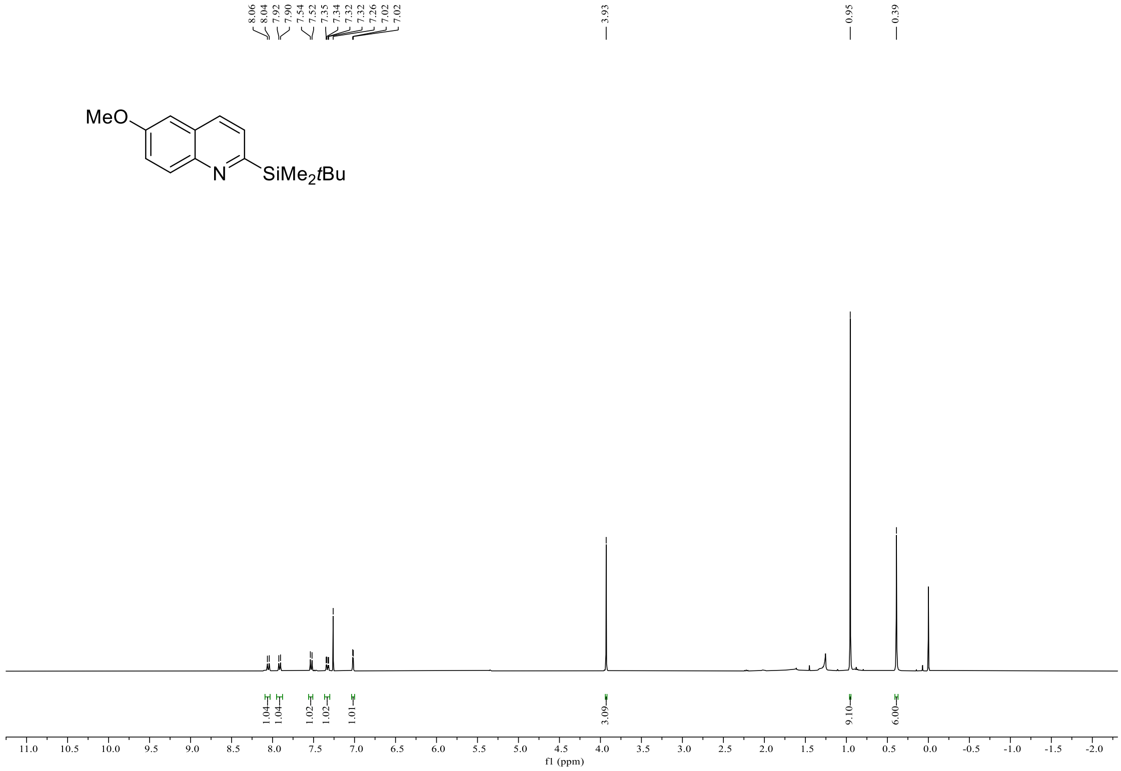

^{13}C NMR (101 MHz, $CDCl_3$) of 7

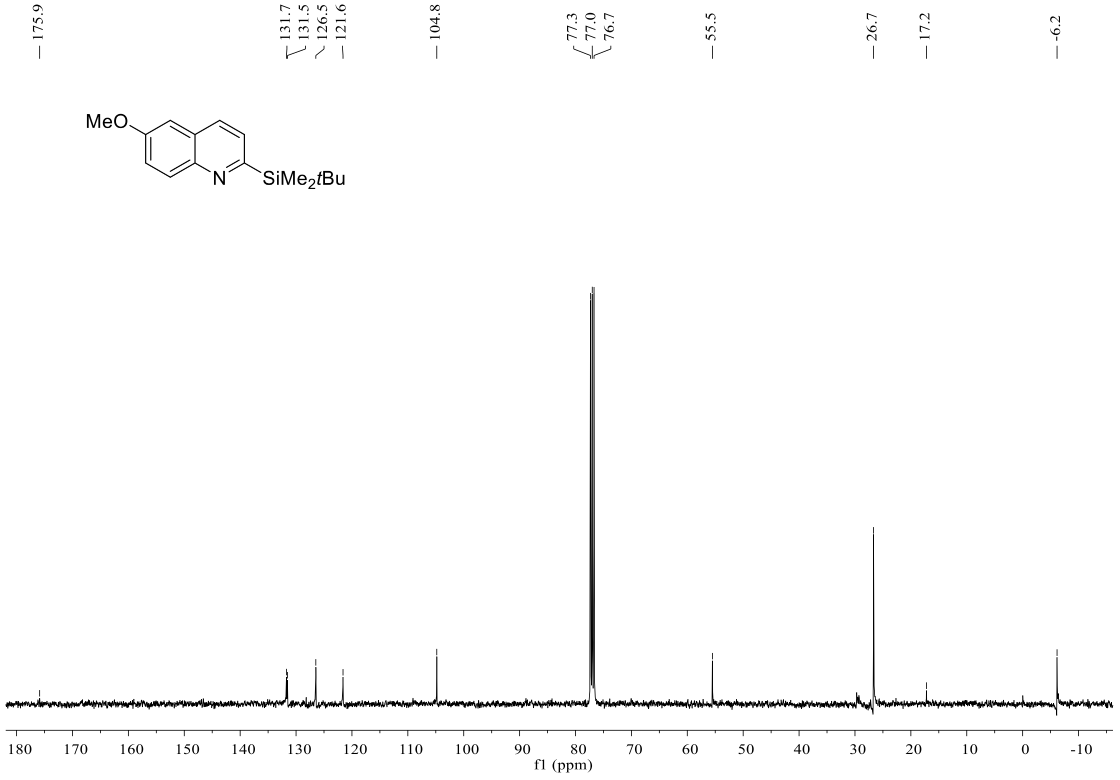

^{1}H NMR (400 MHz, $CDCl_3$) of 8

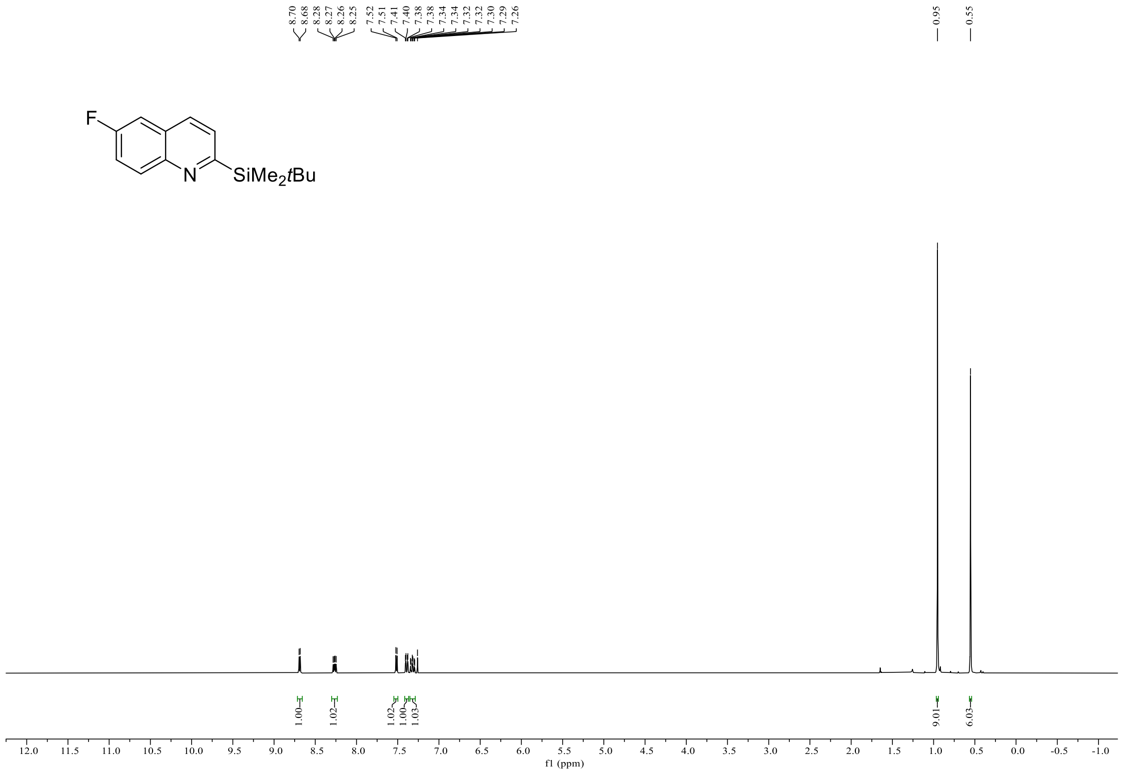

^{13}C NMR (101 MHz, $CDCl_3$) of 8

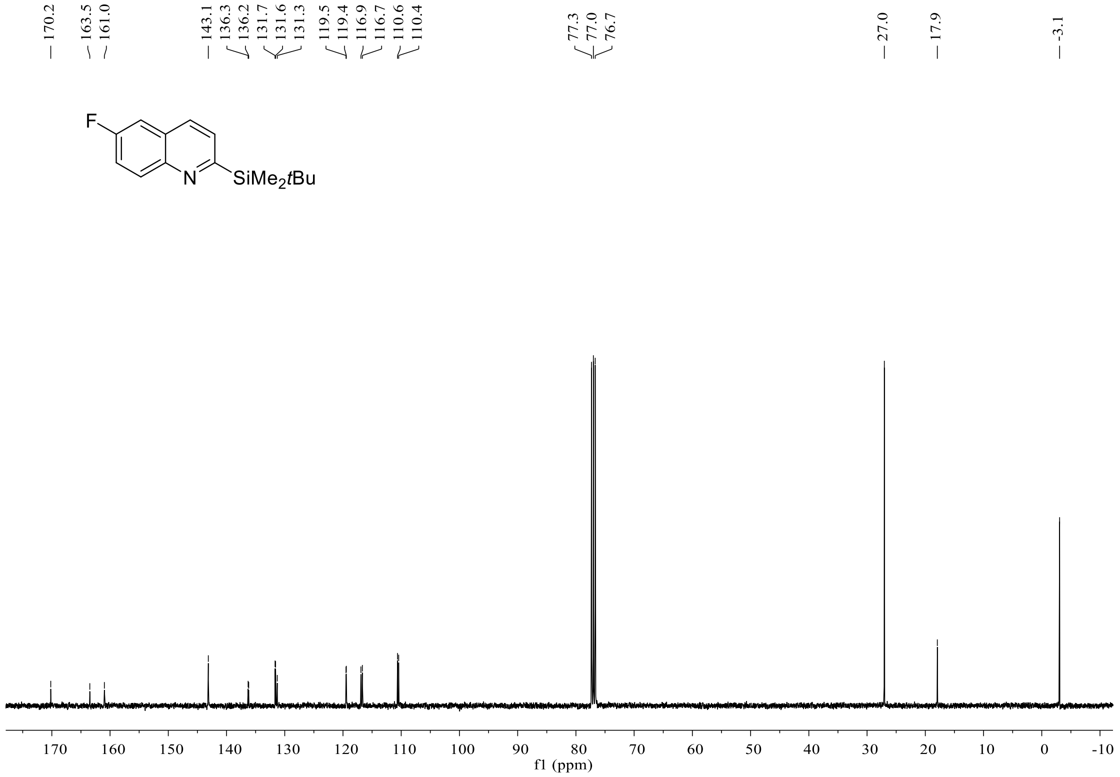

^{19}F NMR (376 MHz, $CDCl_3$) of 8

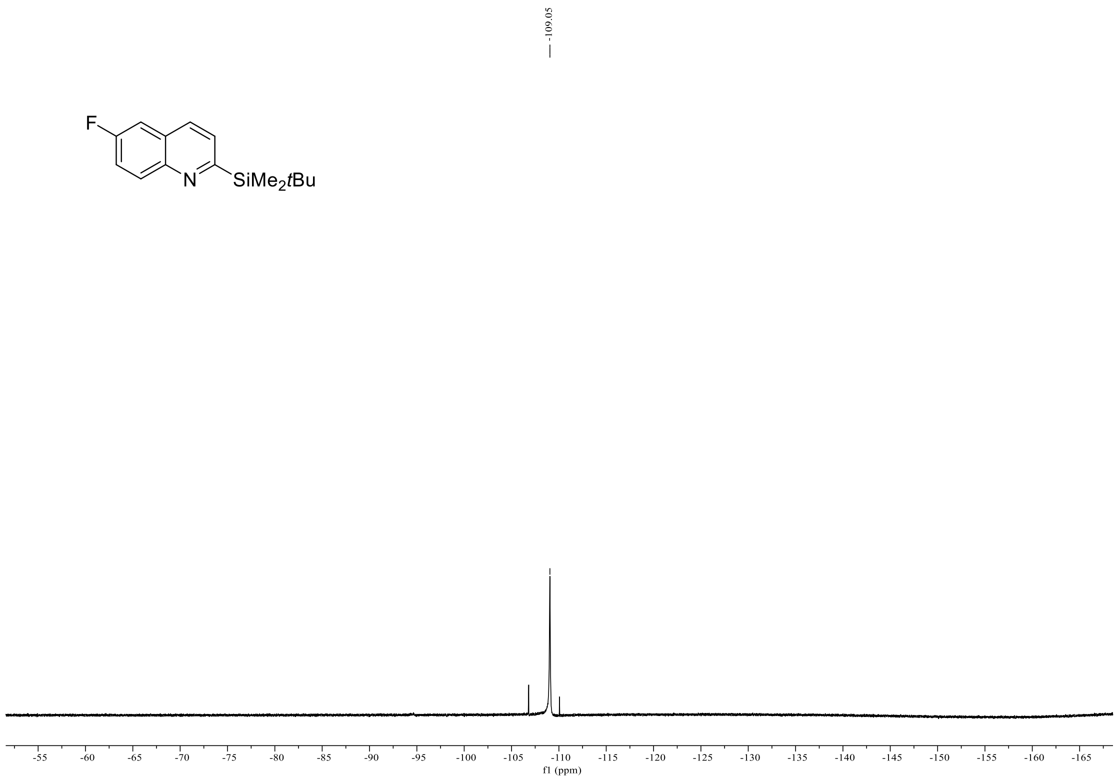

^{1}H NMR (400 MHz, $CDCl_3$) of 9

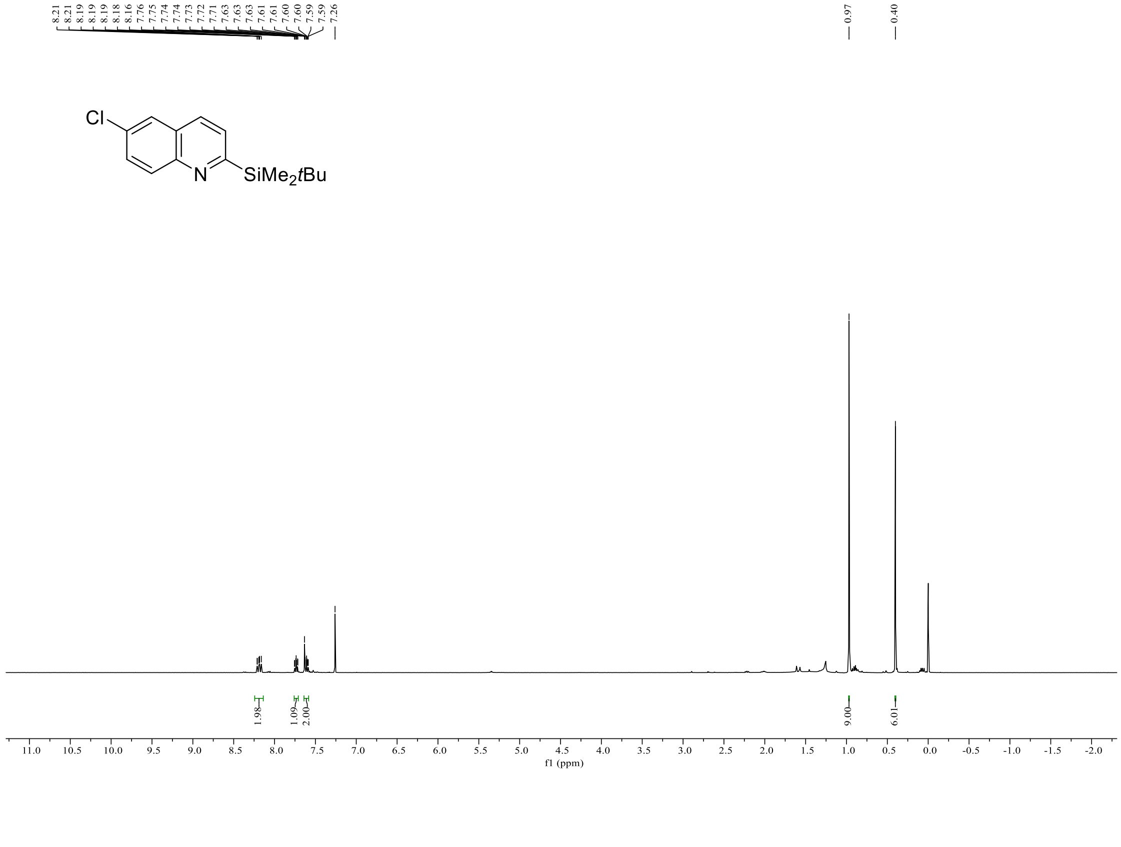

^{13}C NMR (101 MHz, $CDCl_3$) of 9

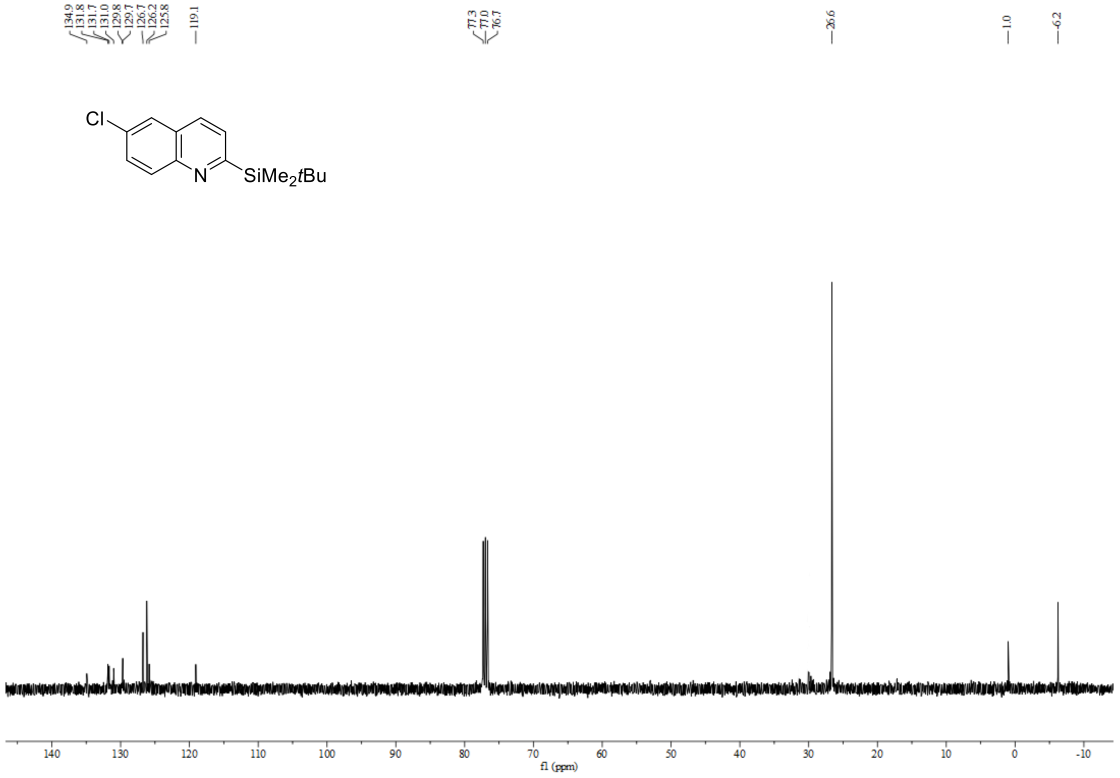

^{1}H NMR (400 MHz, $CDCl_3$) of 10

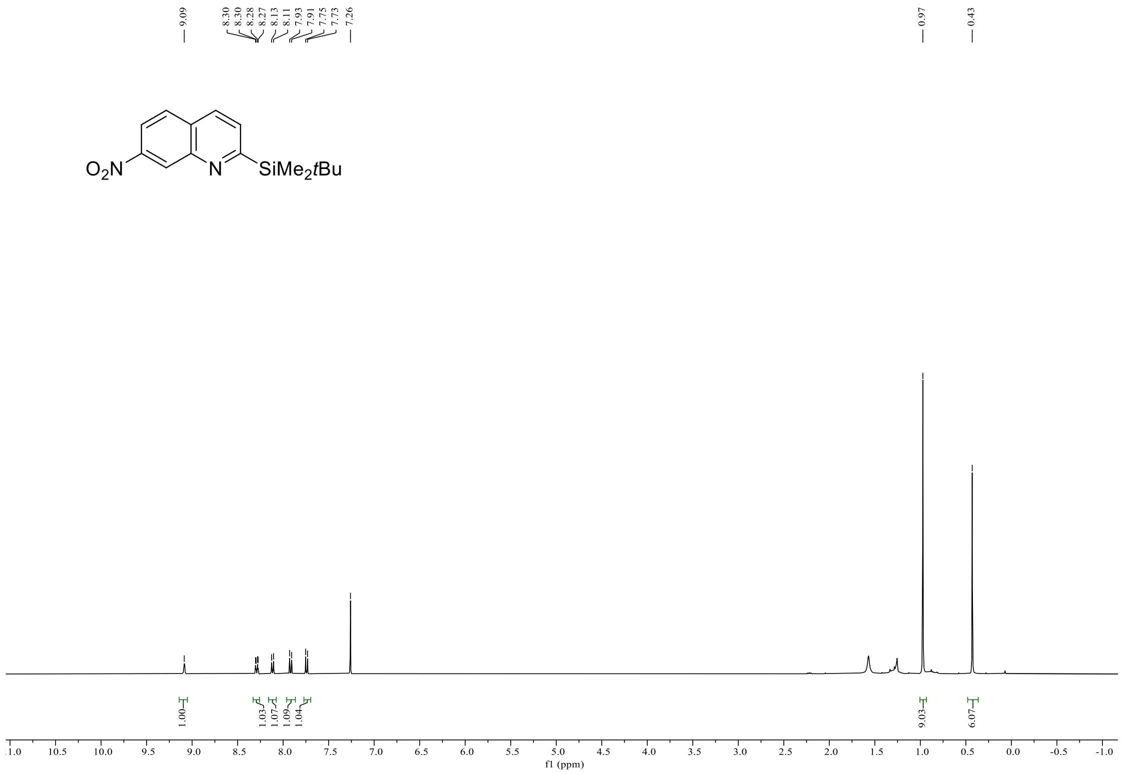

^{13}C NMR (101 MHz, $CDCl_3$) of 10

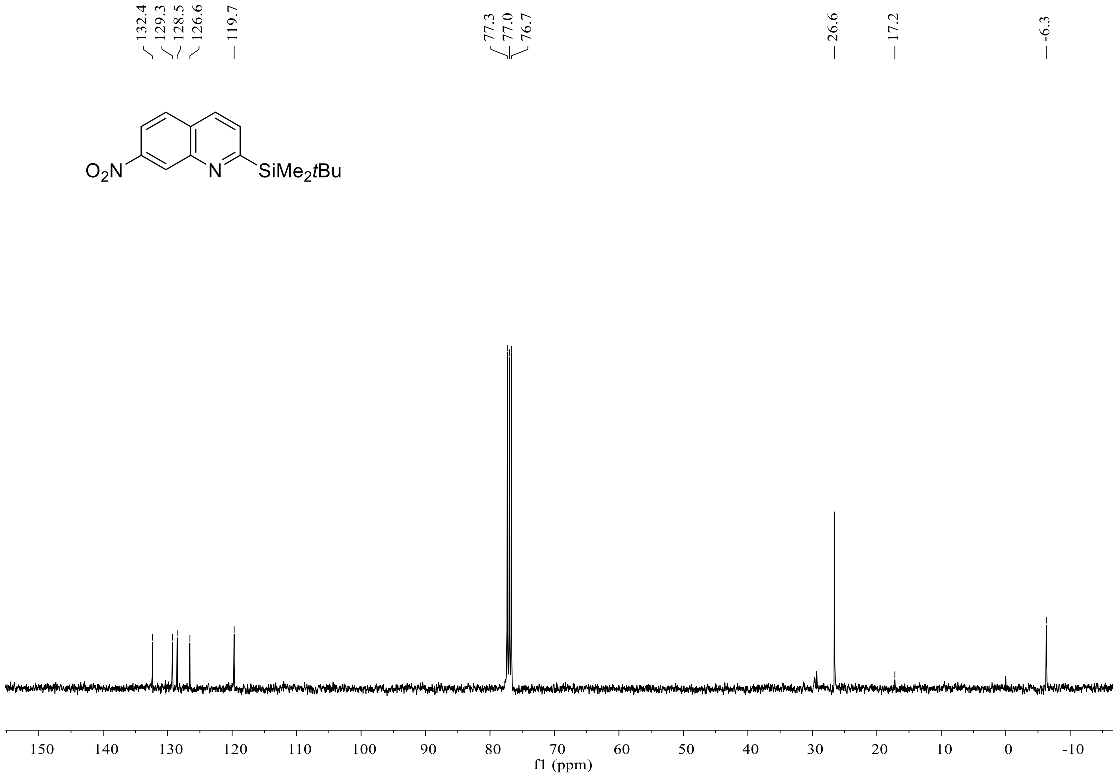

^{1}H NMR (400 MHz, $CDCl_3$) of 11

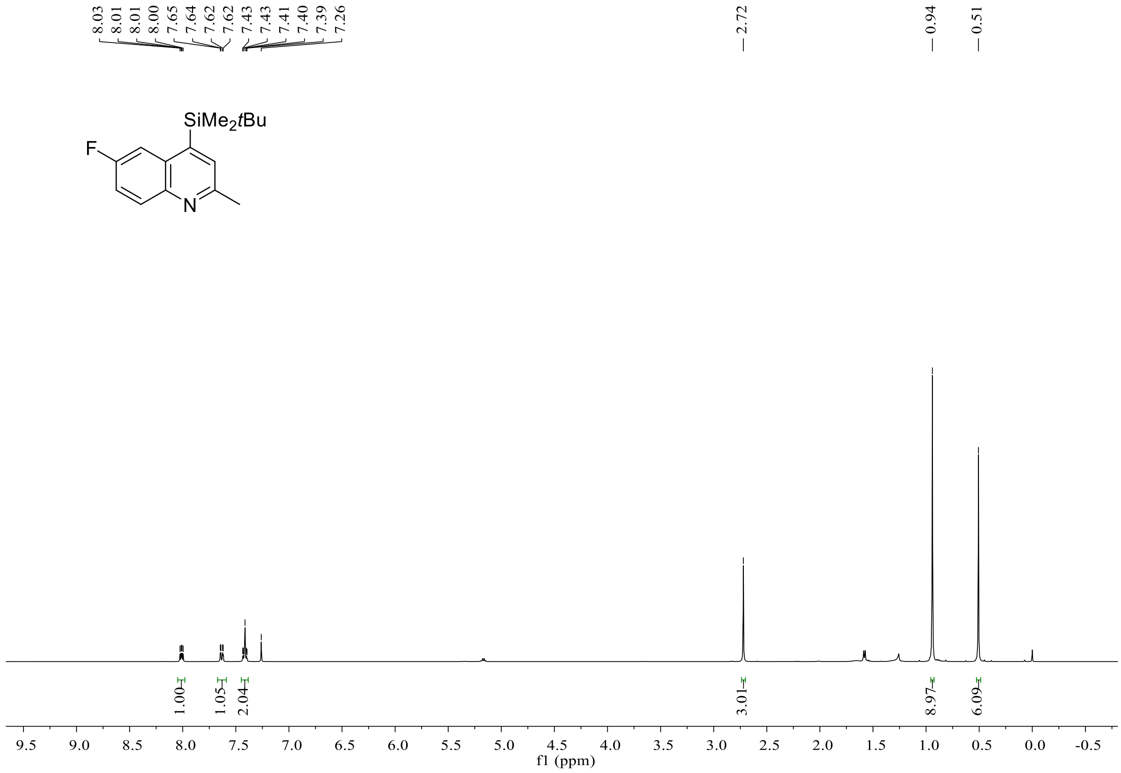

^{13}C NMR (101 MHz, $CDCl_3$) of 11

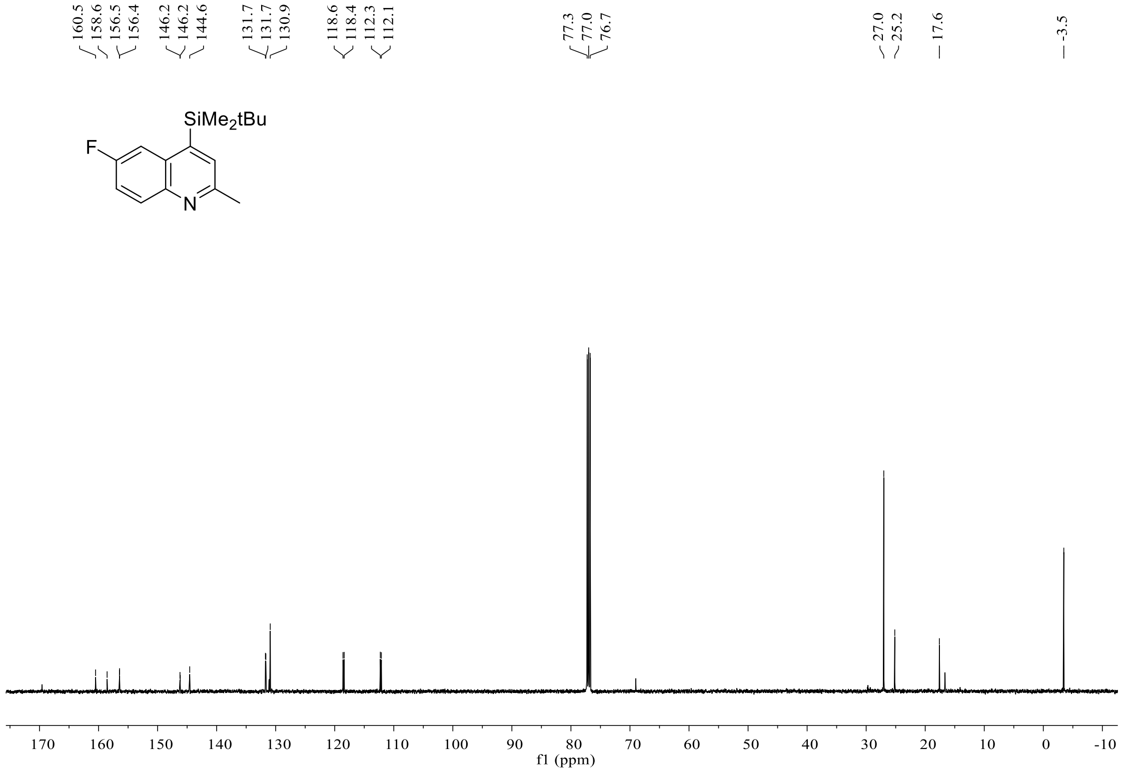

^{19}F NMR (376 MHz, $CDCl_3$) of 11

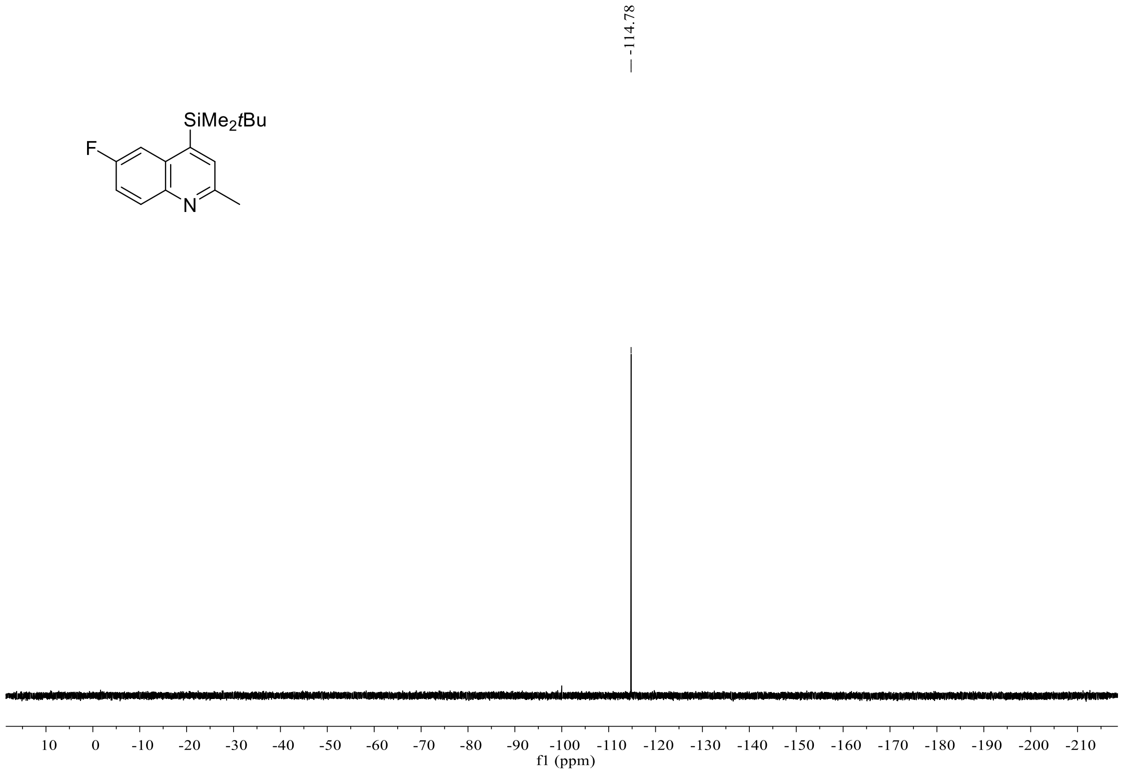

^{1}H NMR (400 MHz, $CDCl_3$) of 12

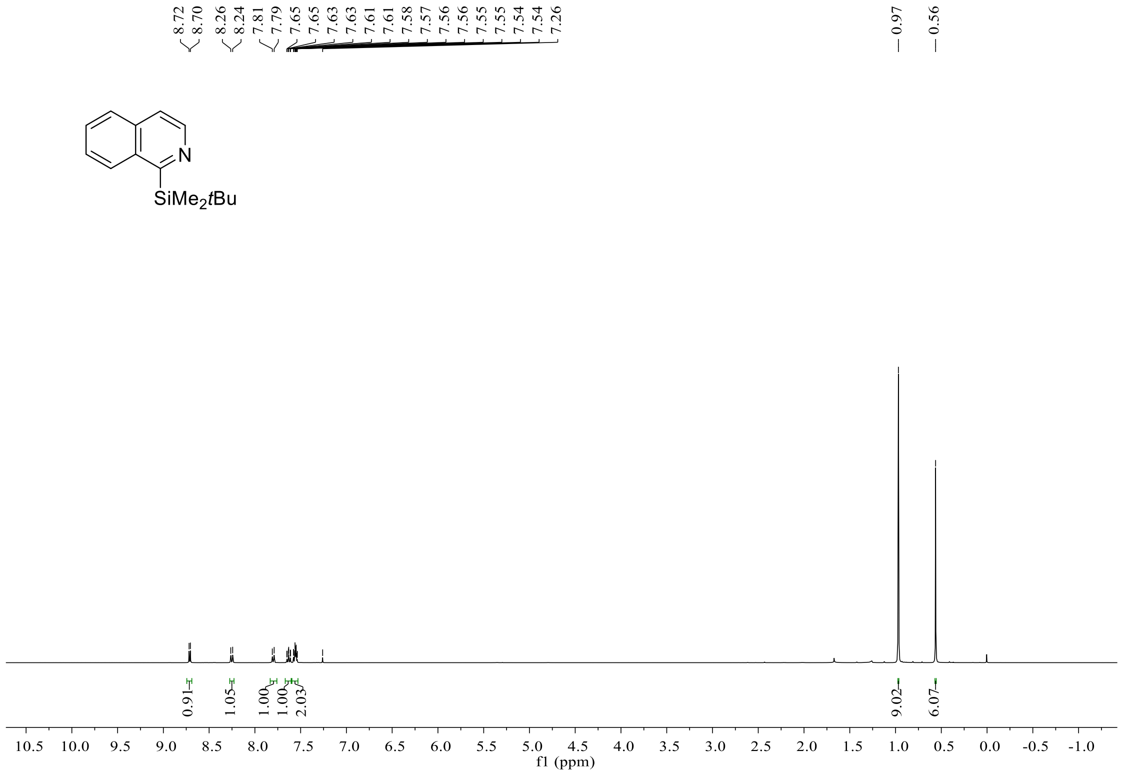

^{13}C NMR (101 MHz, $CDCl_3$) of 12

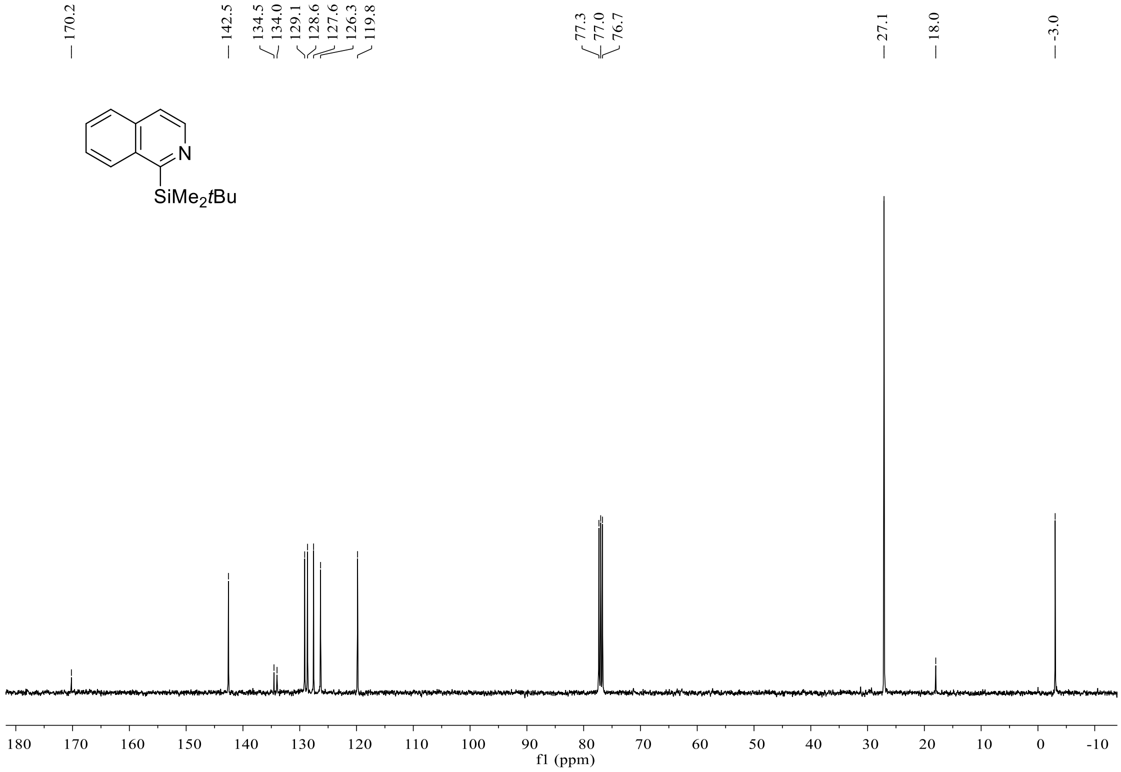

^{1}H NMR (400 MHz, $CDCl_3$) of 13

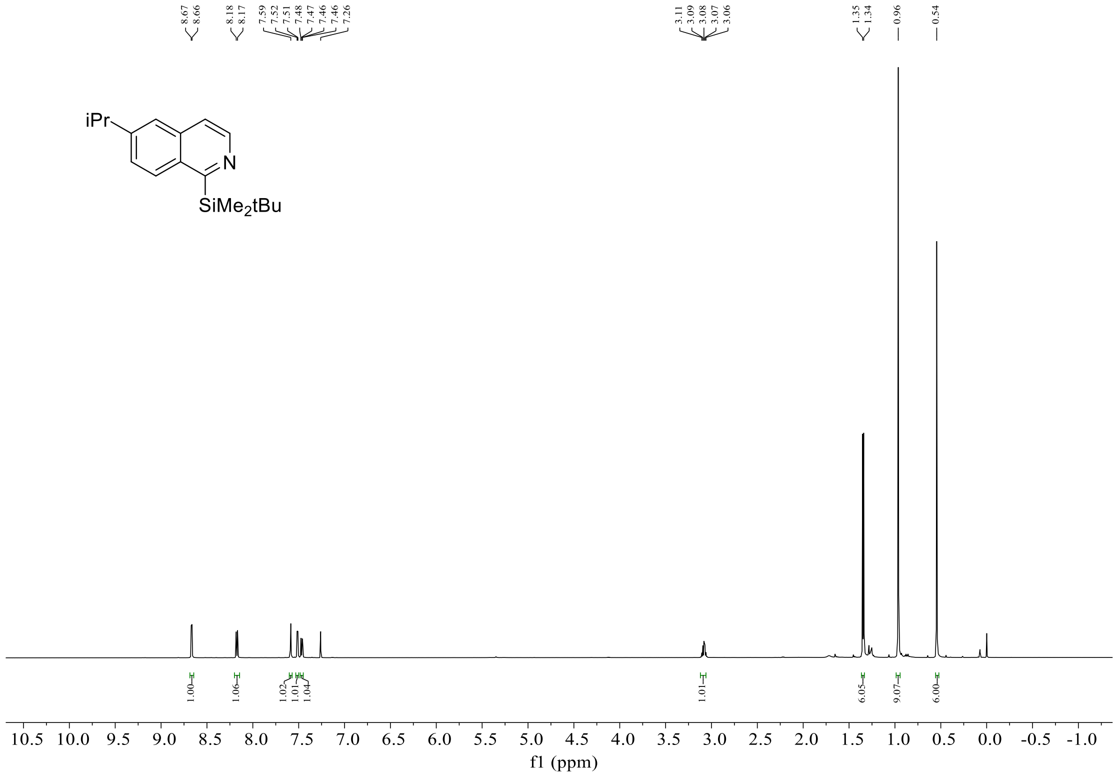

^{13}C NMR (101 MHz, $CDCl_3$) of 13

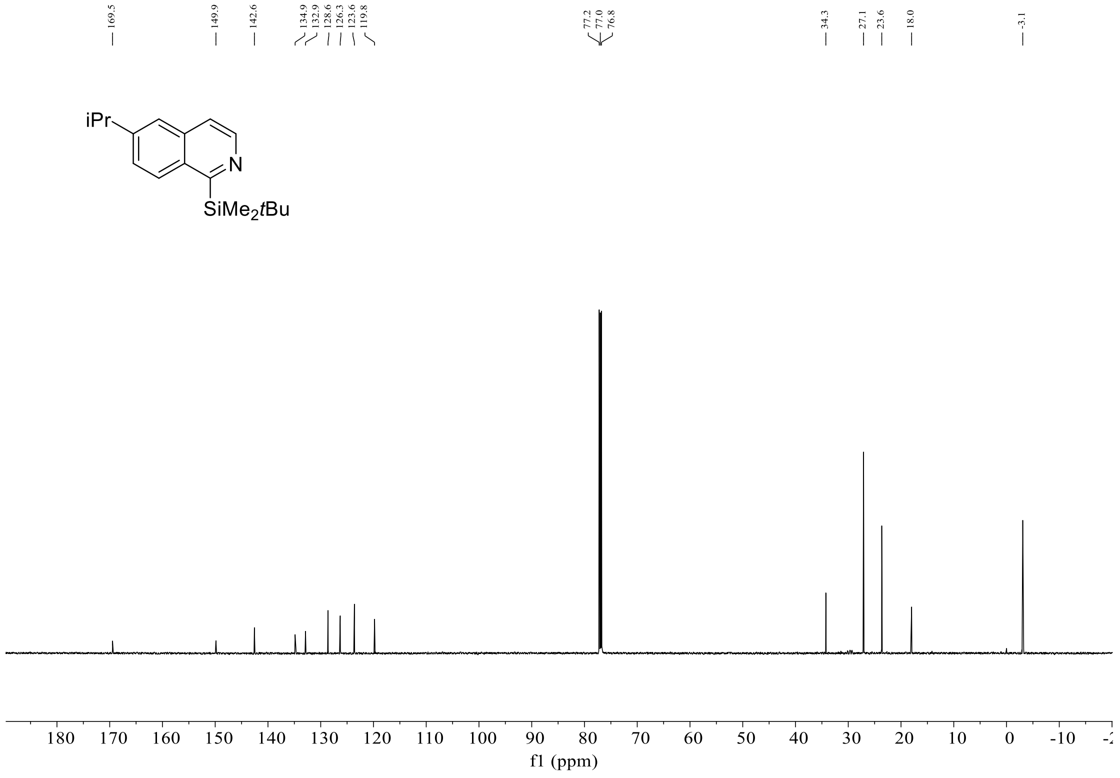

^{1}H NMR (400 MHz, $CDCl_3$) of 14

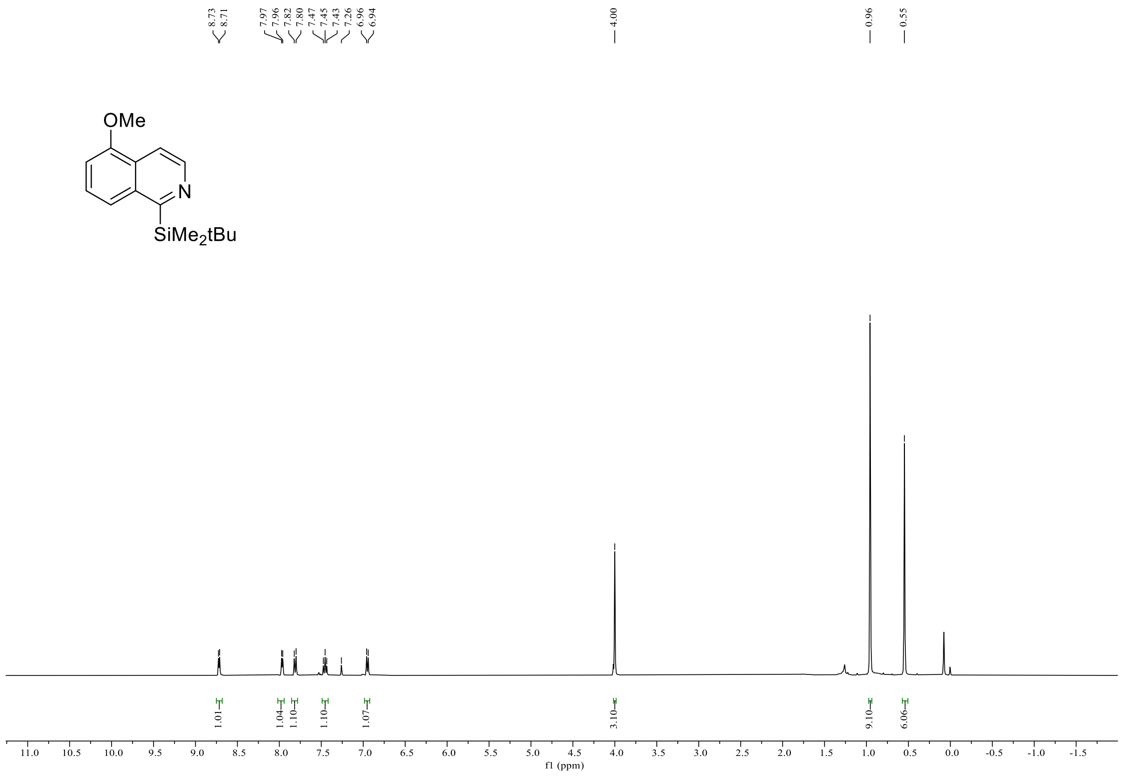

^{13}C NMR (101 MHz, $CDCl_3$) of 14

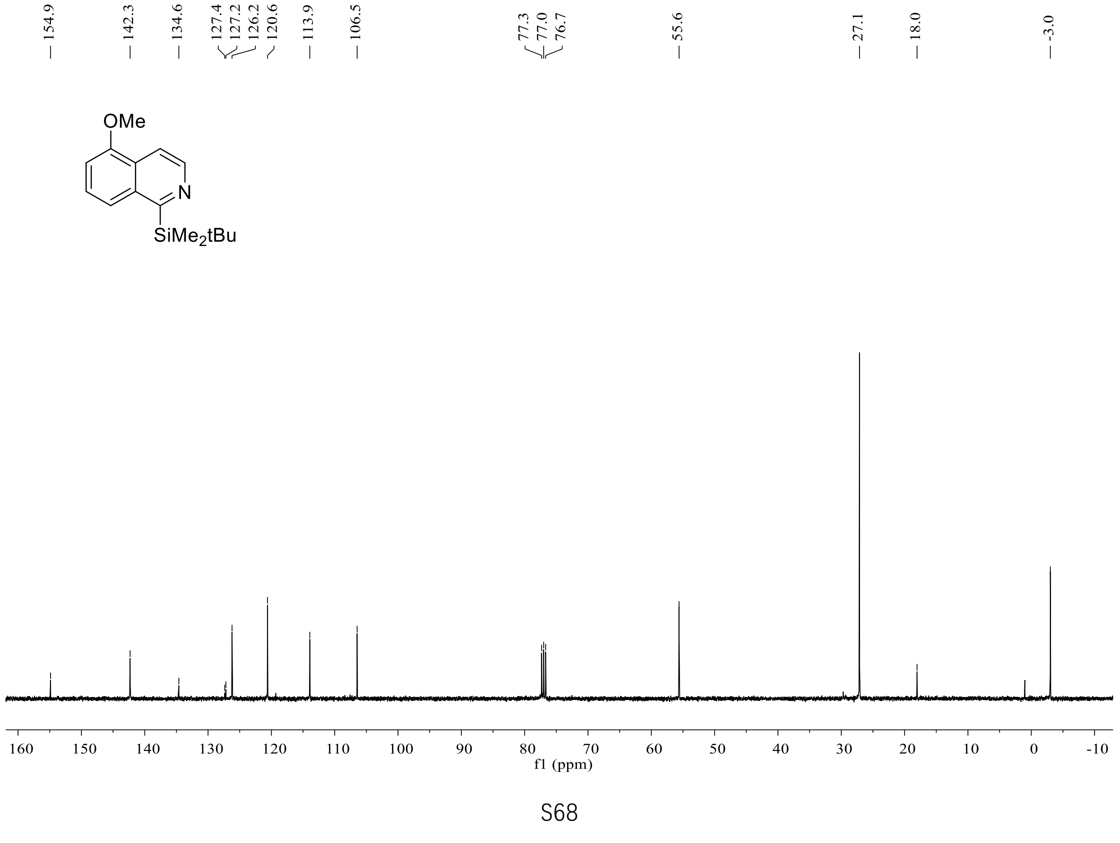

^{1}H NMR (400 MHz, CDCl$_3$) of 15

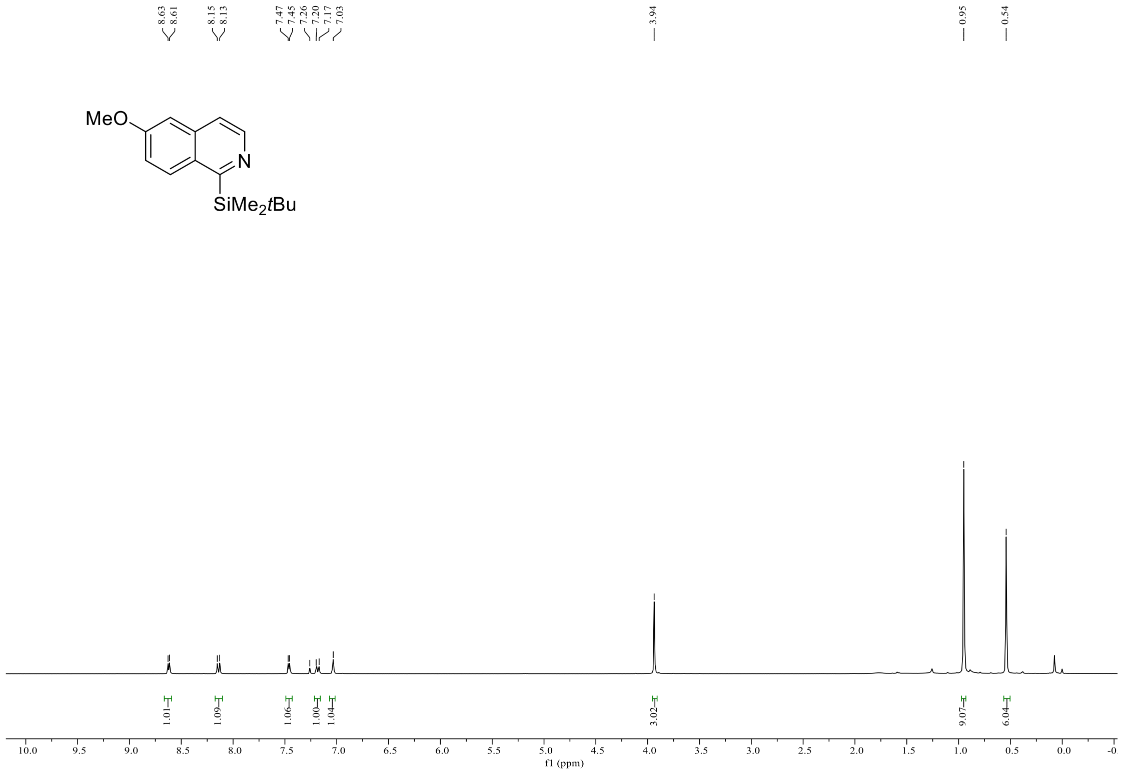

^{13}C NMR (101 MHz, CDCl$_3$) of 15

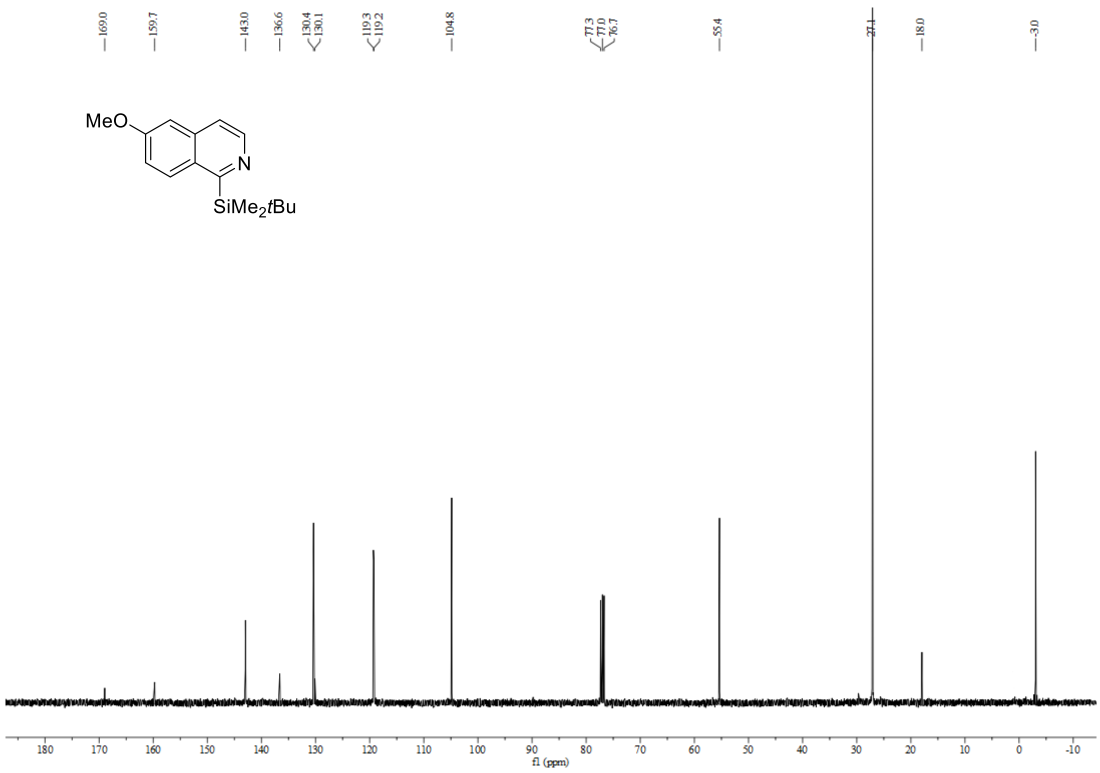

^{1}H NMR (400 MHz, $CDCl_3$) of 16

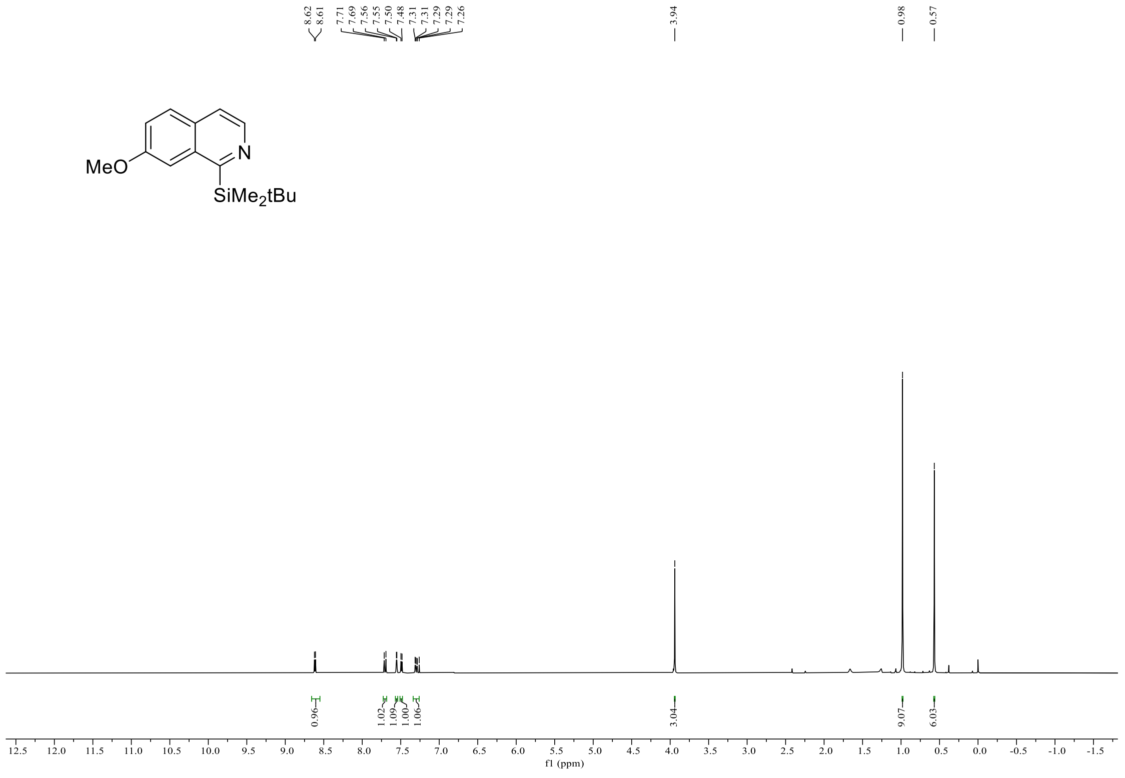

^{13}C NMR (101 MHz, $CDCl_3$) of 16

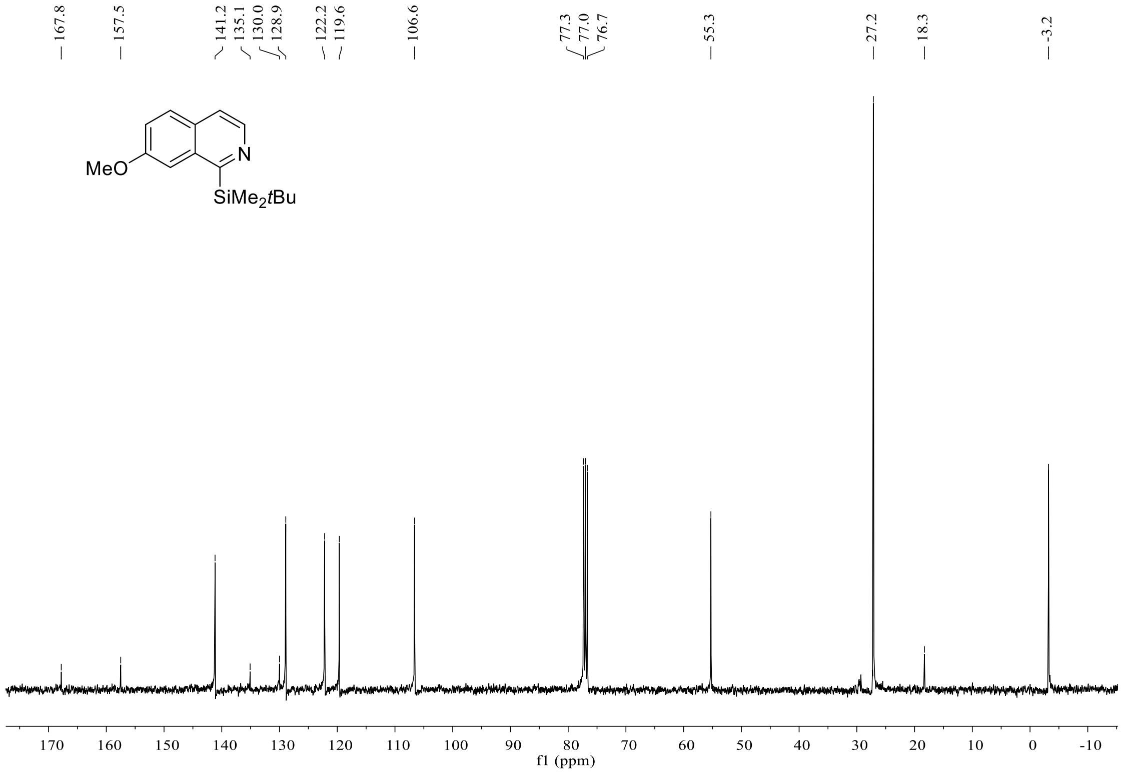

^{1}H NMR (400 MHz, $CDCl_3$) of 17

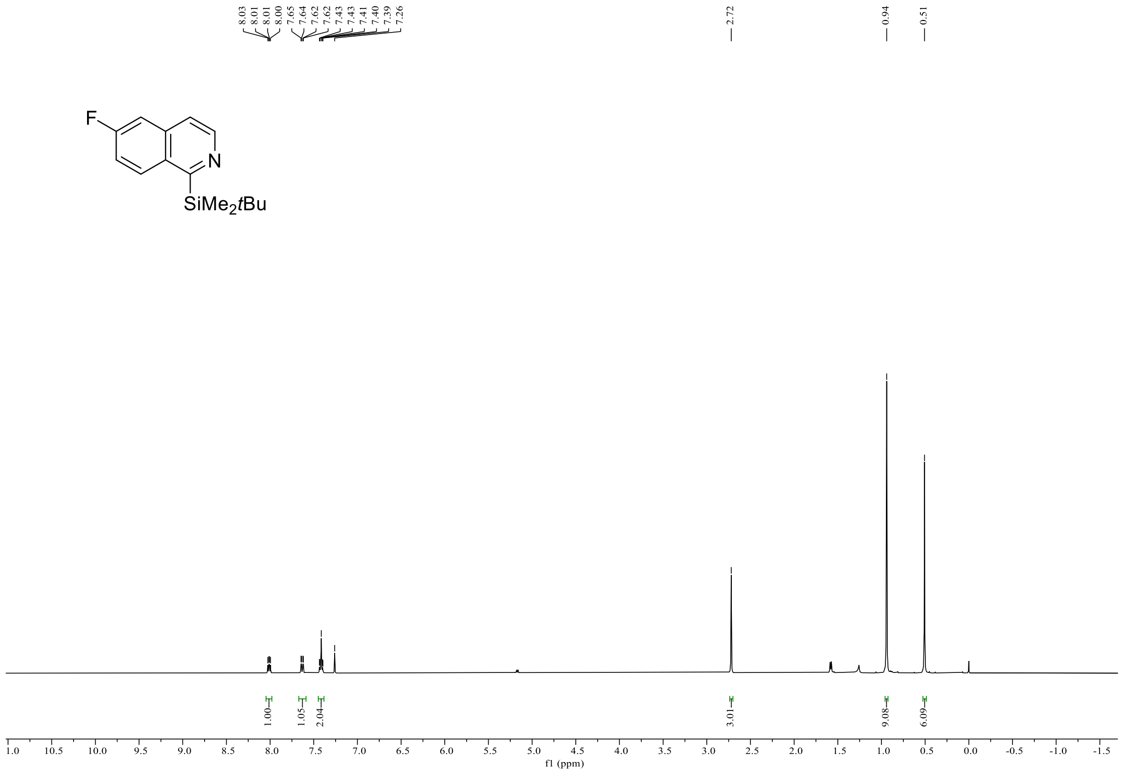

^{13}C NMR (101 MHz, $CDCl_3$) of 17

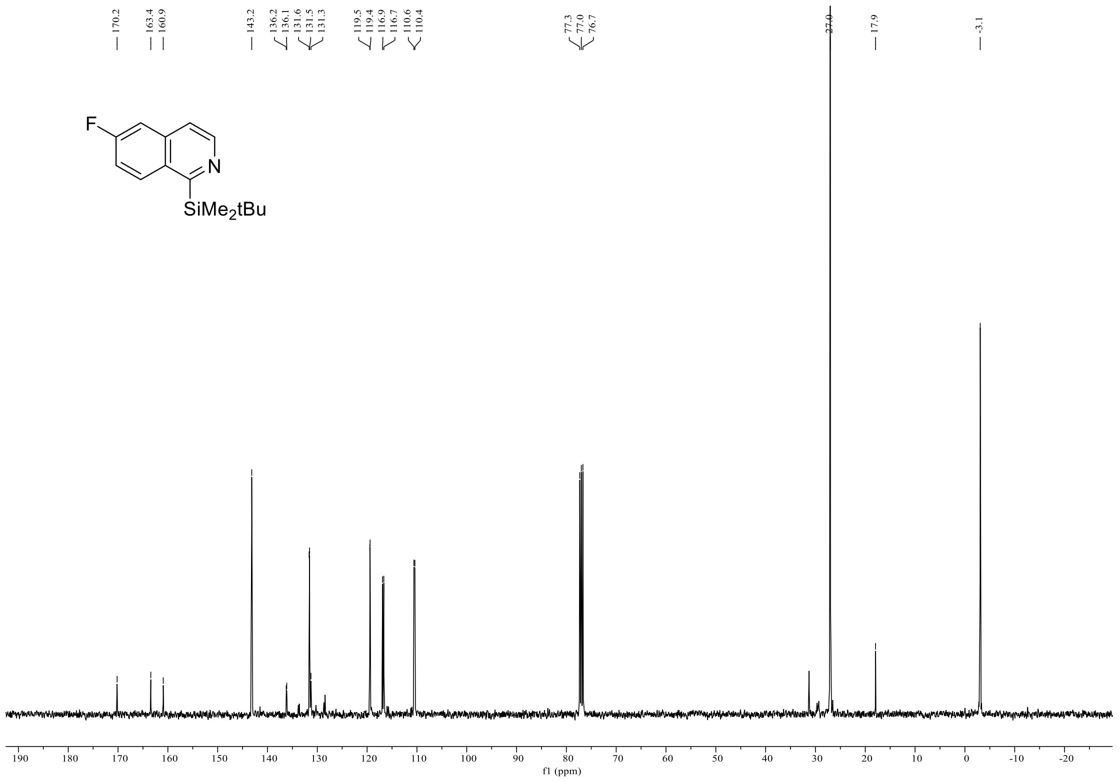

^{19}F NMR (376 MHz, CDCl$_3$) of 17

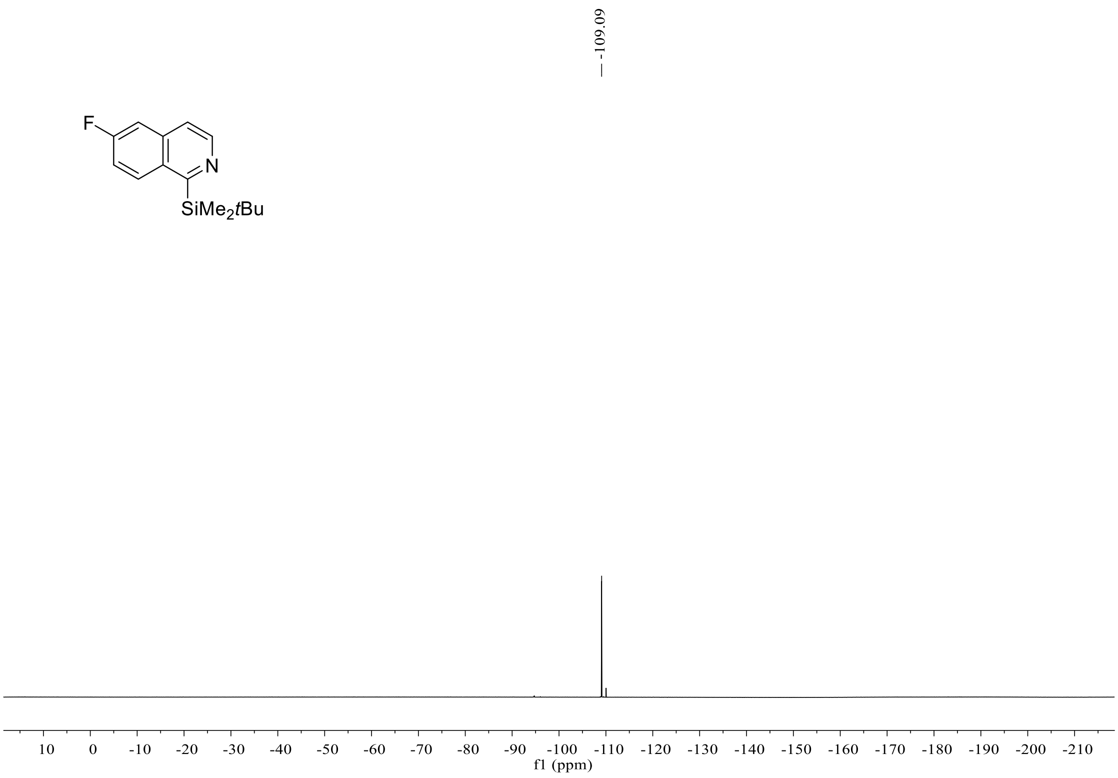

^{1}H NMR (400 MHz, CDCl$_3$) of 18

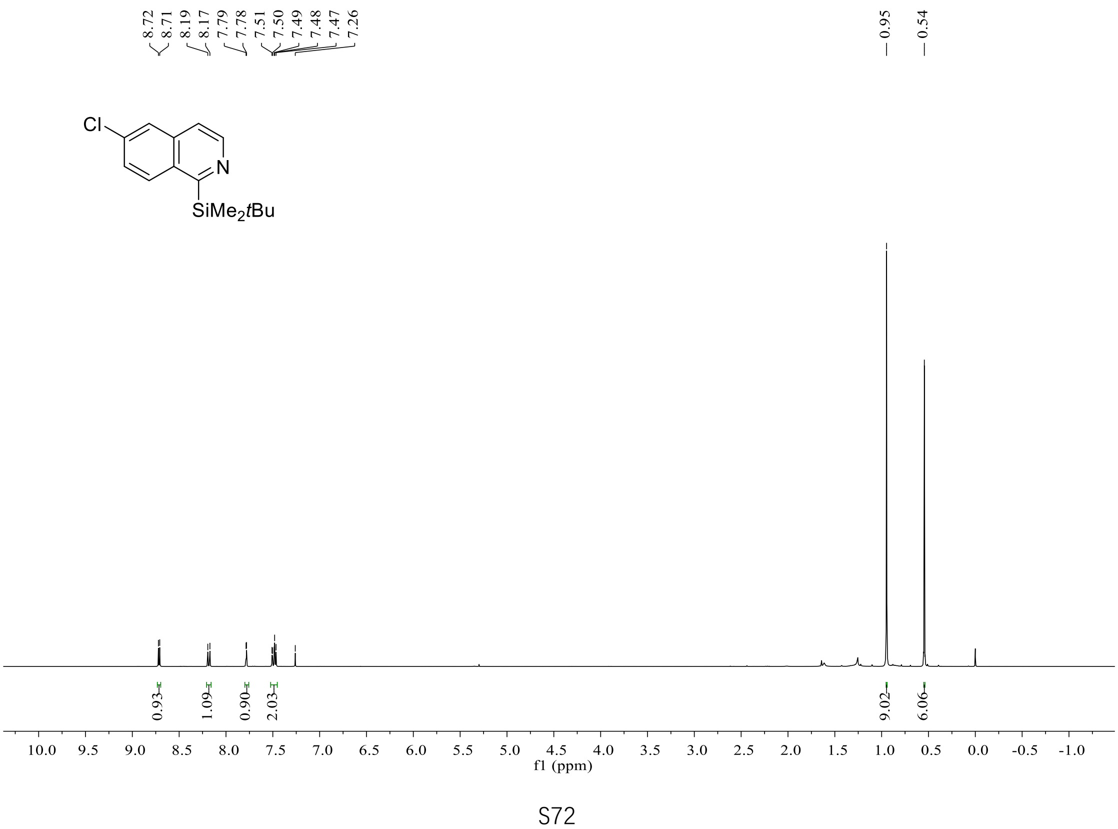

^{13}C NMR (101 MHz, $CDCl_3$) of 18

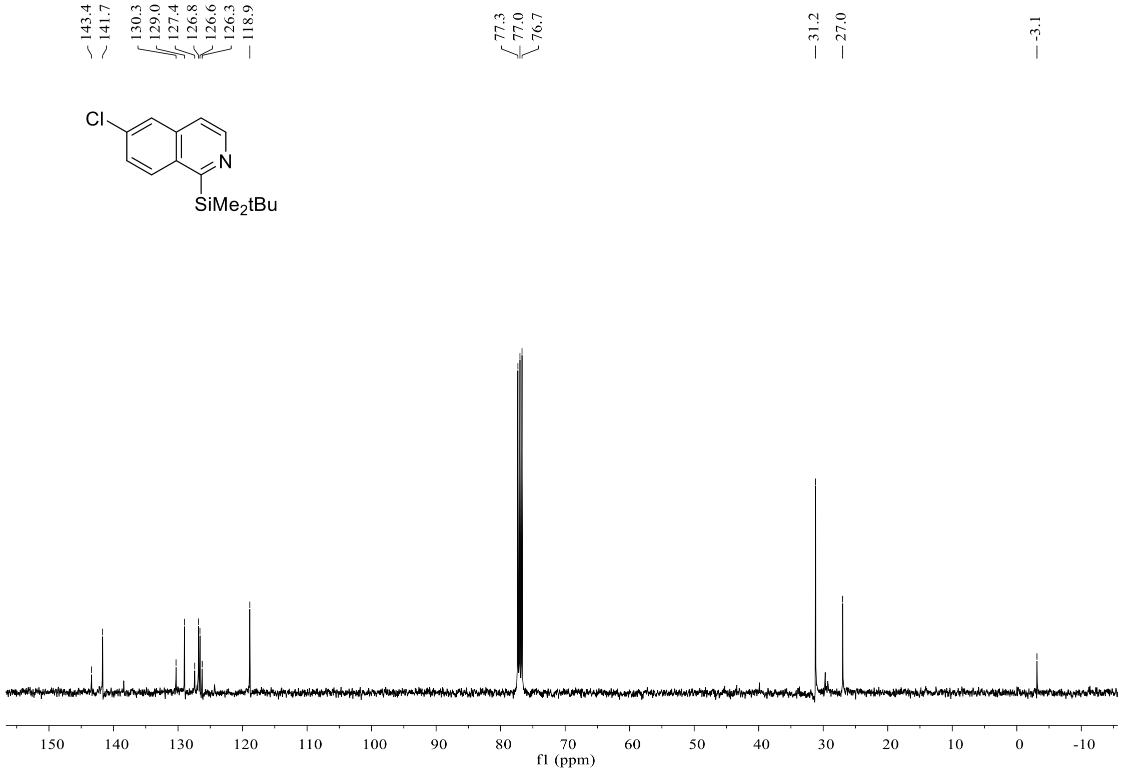

^{1}H NMR (400 MHz, $CDCl_3$) of 20

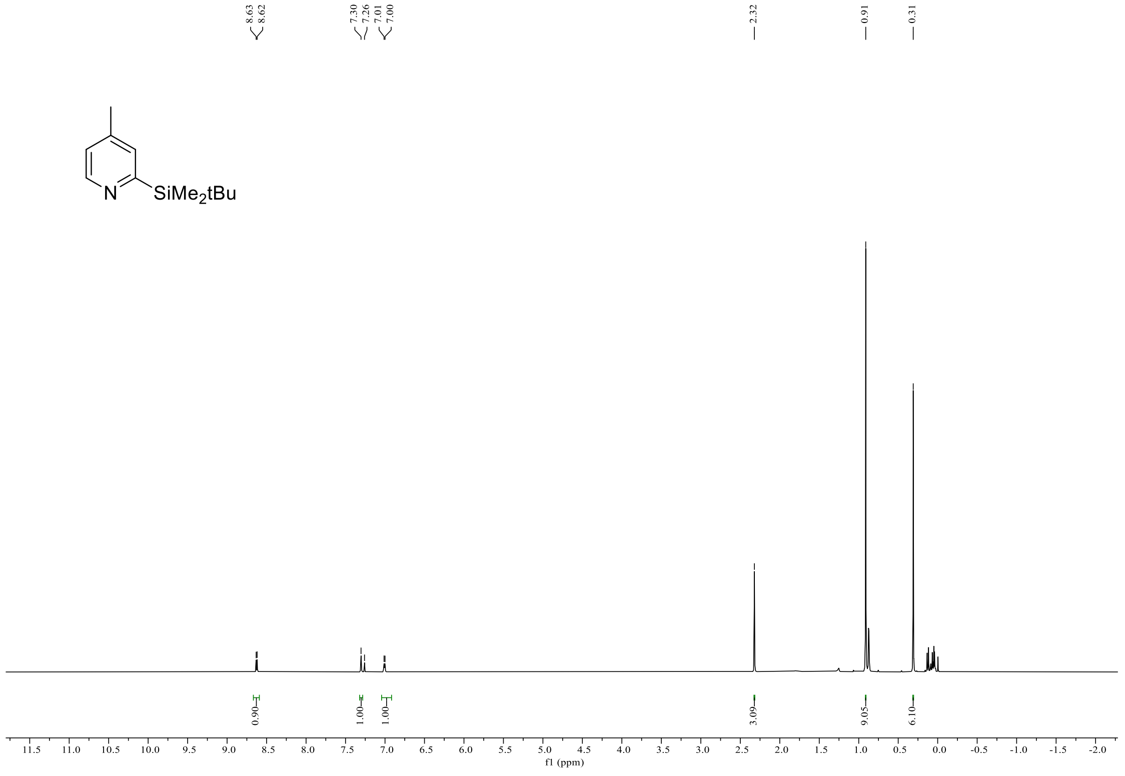

^{13}C NMR (101 MHz, $CDCl_3$) of 20

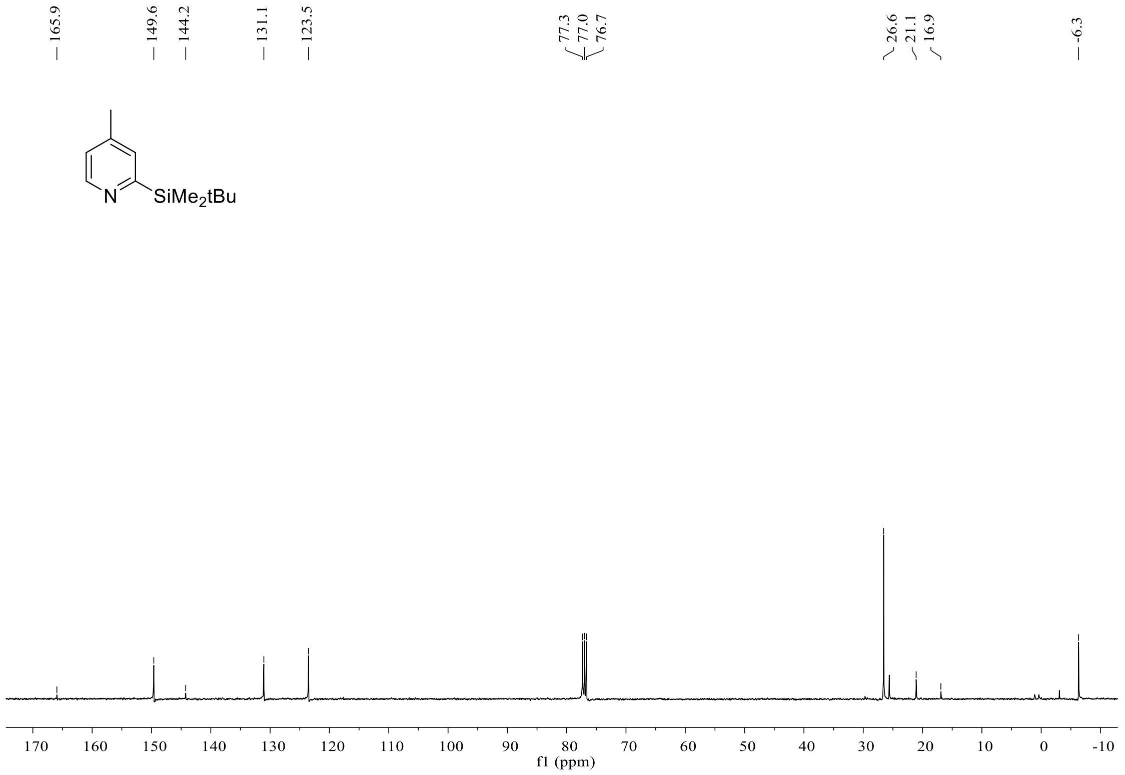

^{1}H NMR (400 MHz, $CDCl_3$) of 21

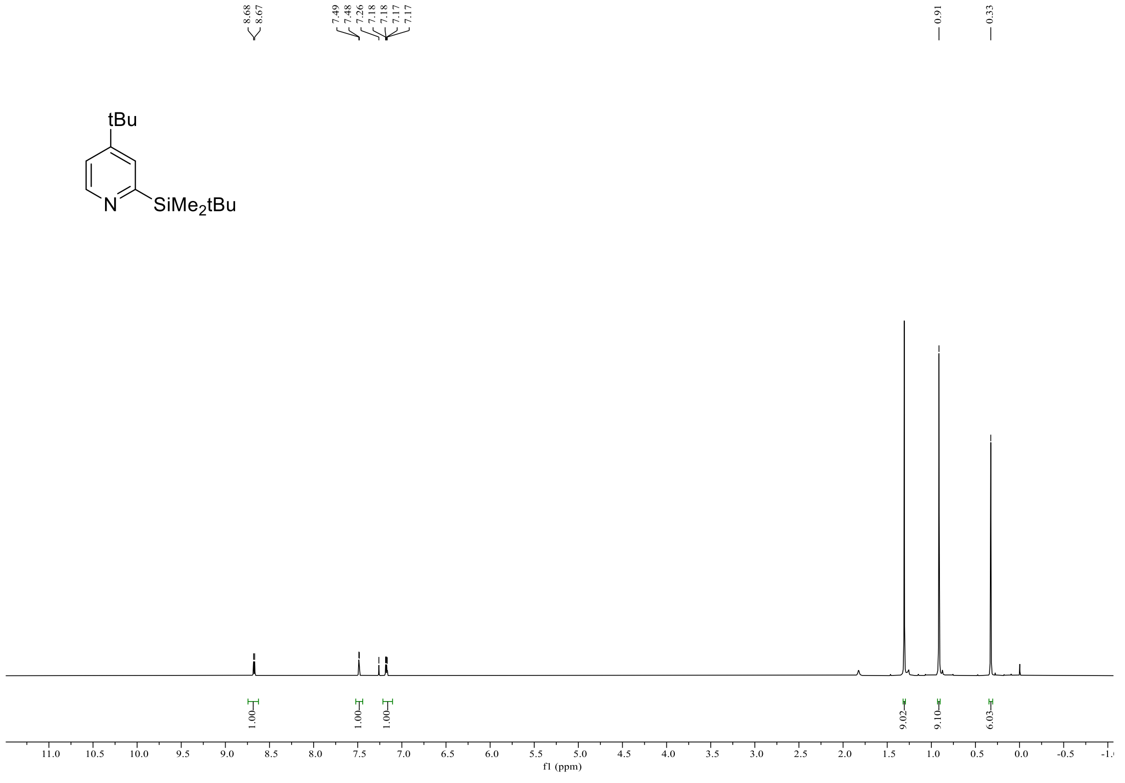

^{13}C NMR (101 MHz, $CDCl_3$) of 21

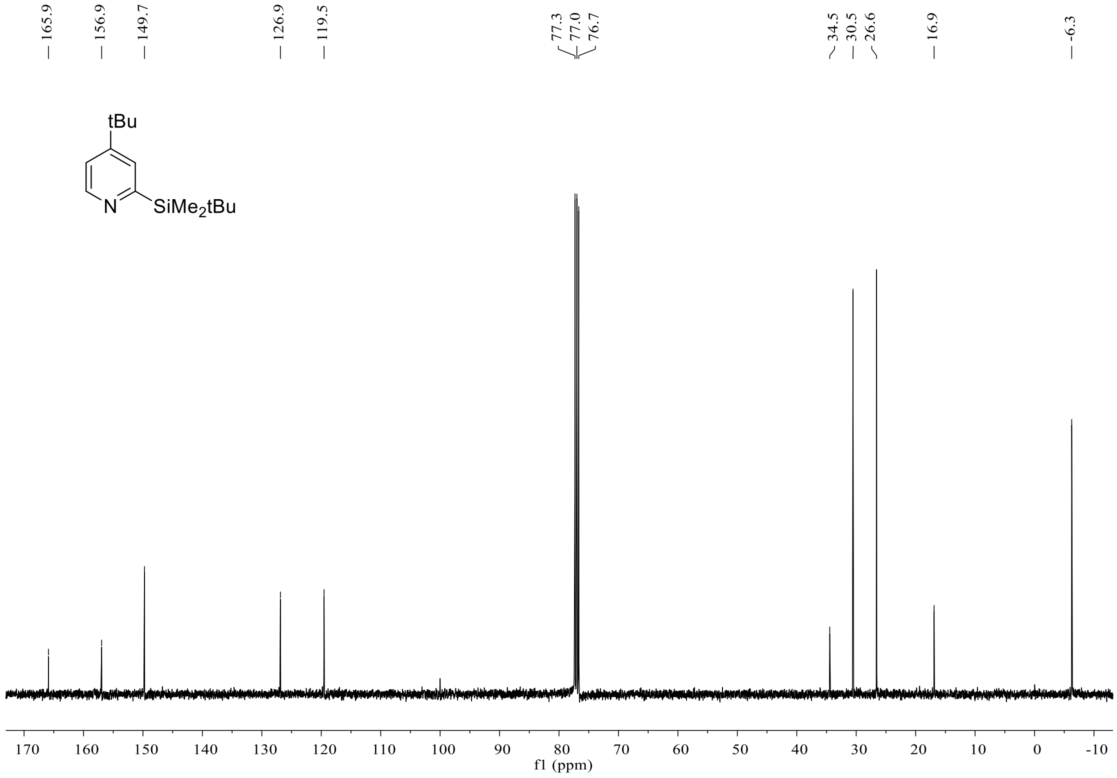

^{1}H NMR (400 MHz, $CDCl_3$) of 22

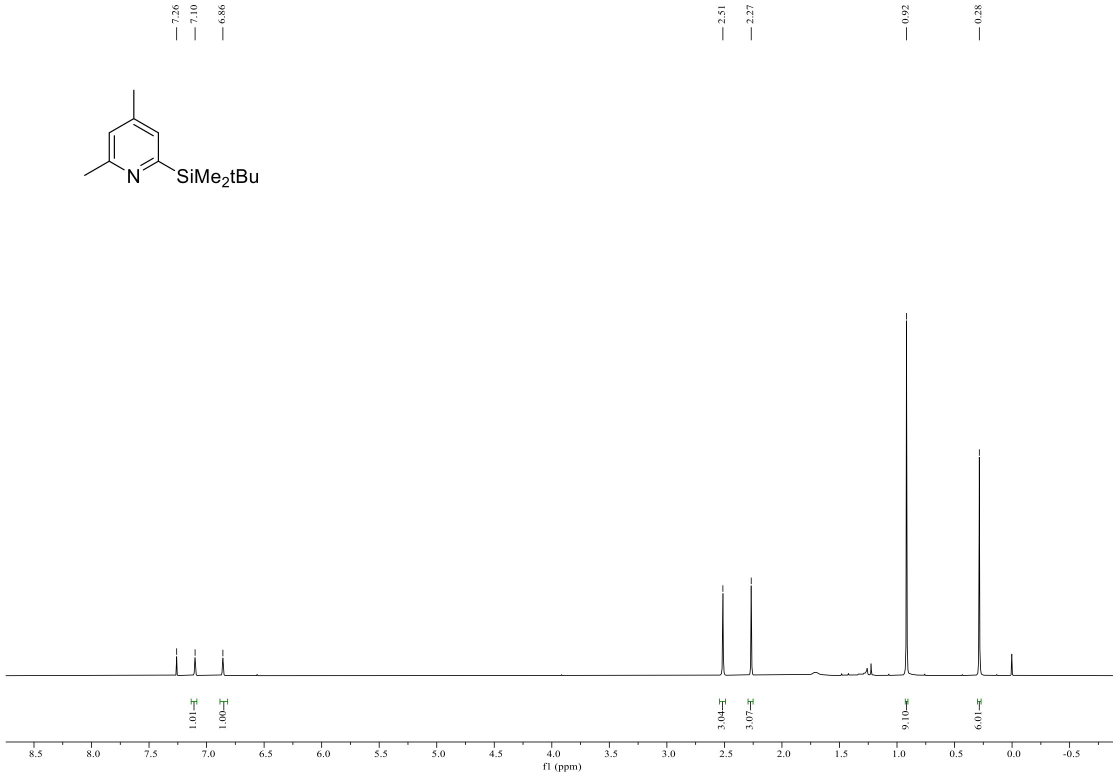

^{13}C NMR (101 MHz, $CDCl_3$) of 22

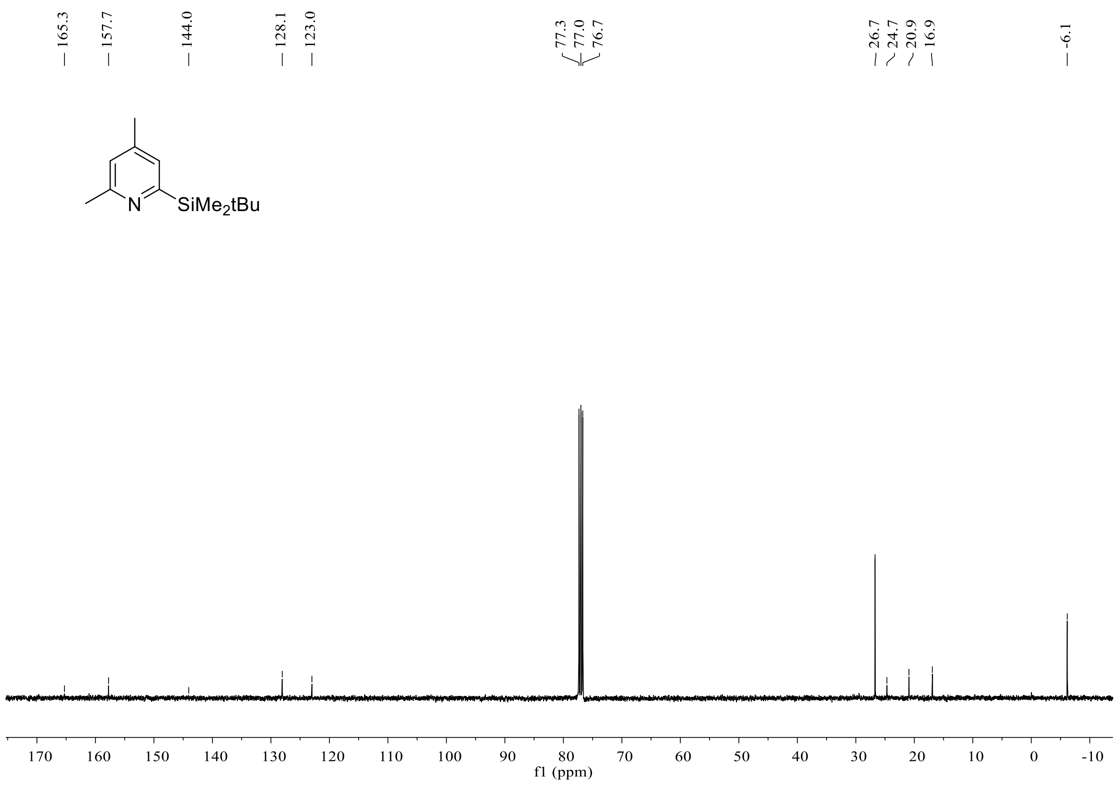

^{1}H NMR (400 MHz, $CDCl_3$) of 23

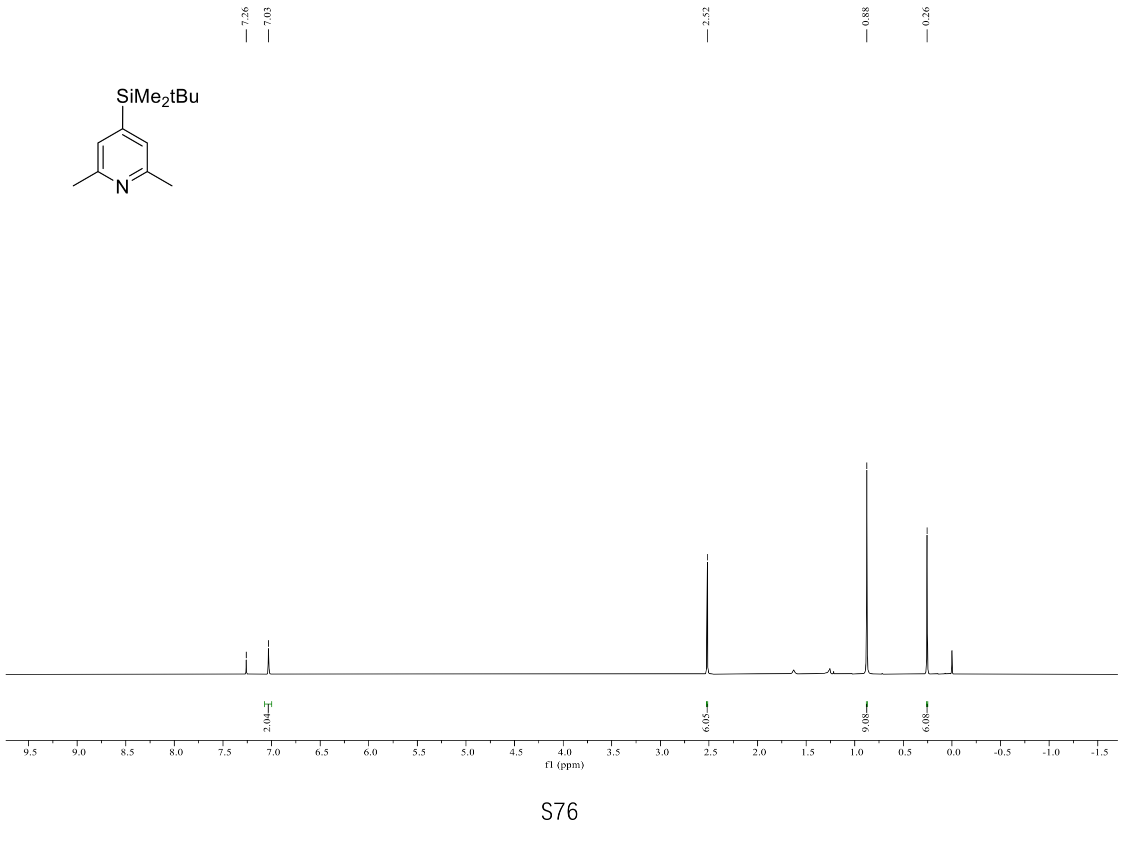

^{13}C NMR (101 MHz, $CDCl_3$) of 23

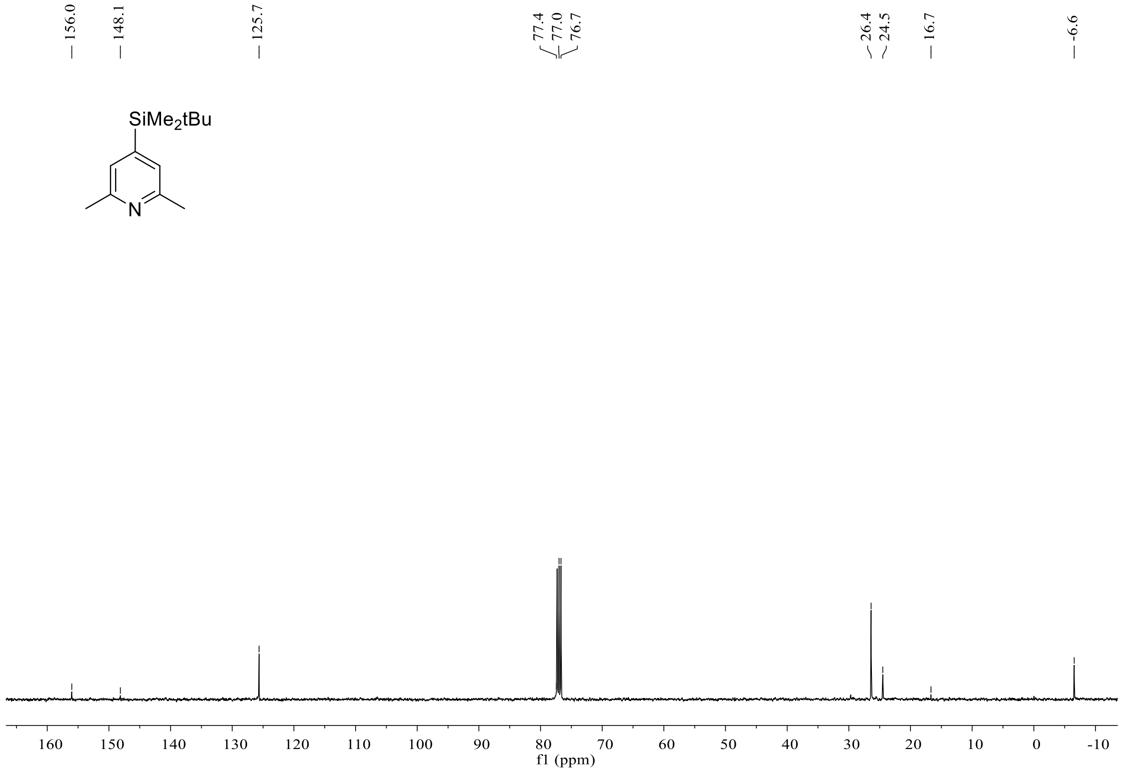

^{1}H NMR (400 MHz, $CDCl_3$) of 24

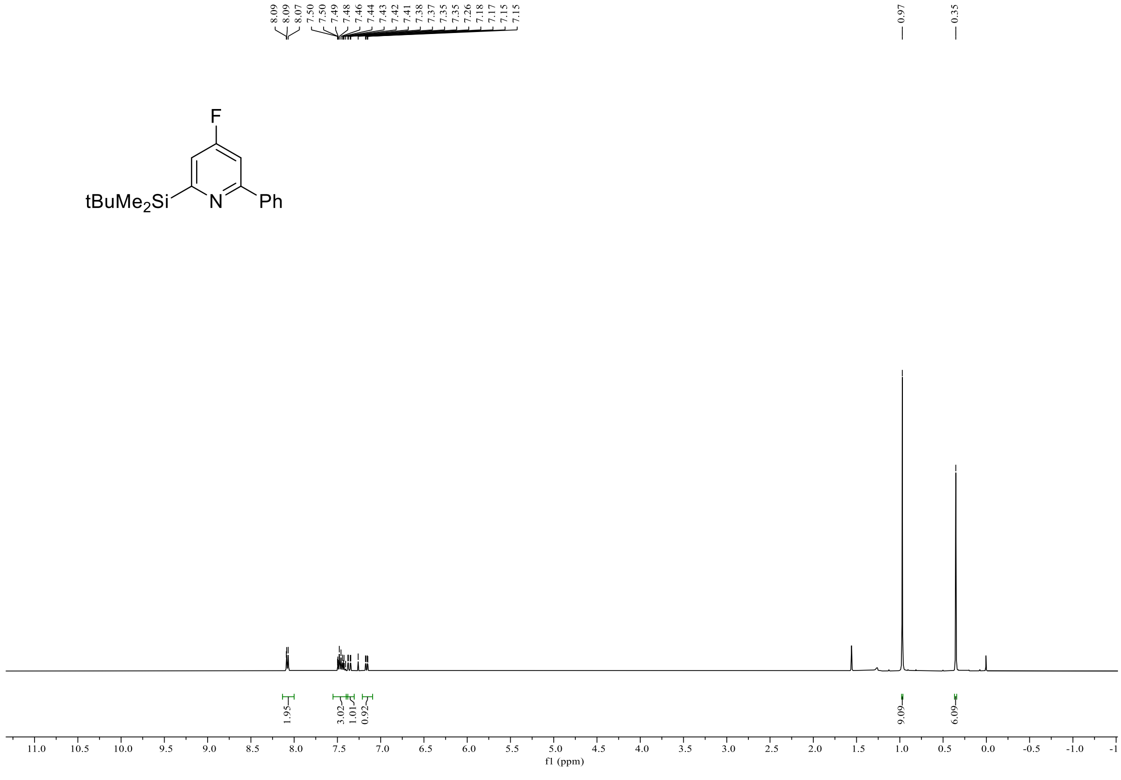

^{13}C NMR (101 MHz, CDCl$_3$) of 24

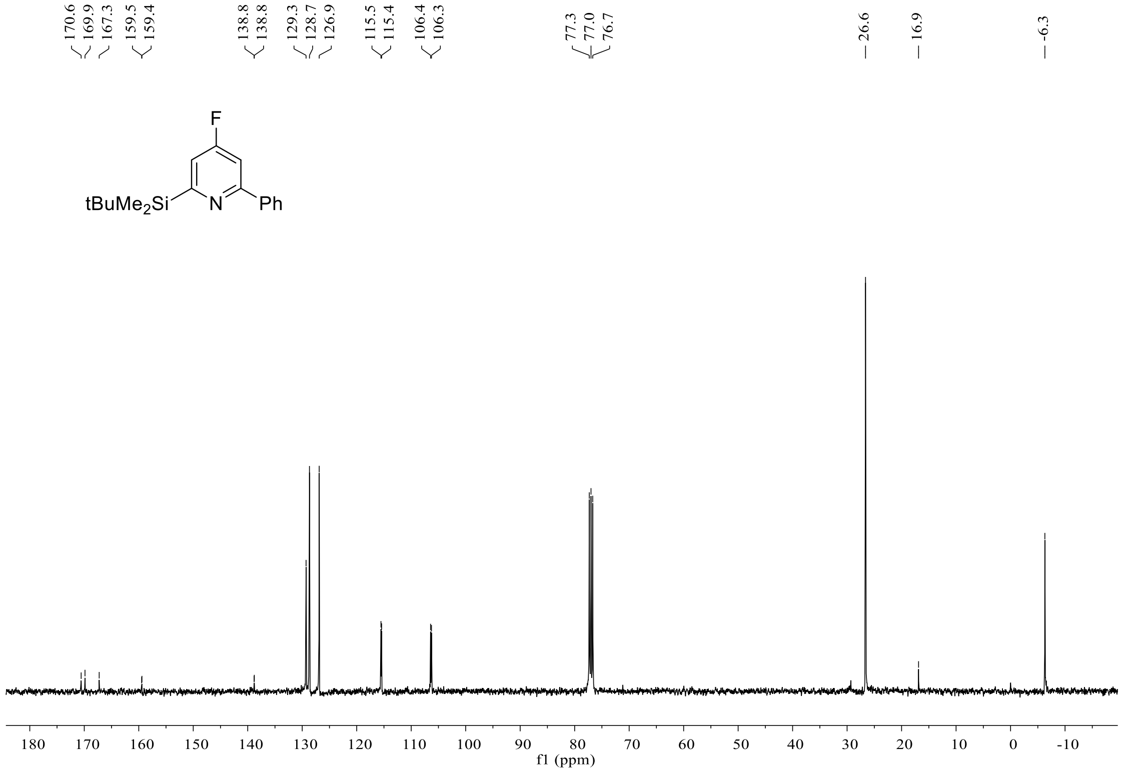

^{19}F NMR (376 MHz, CDCl$_3$) of 24

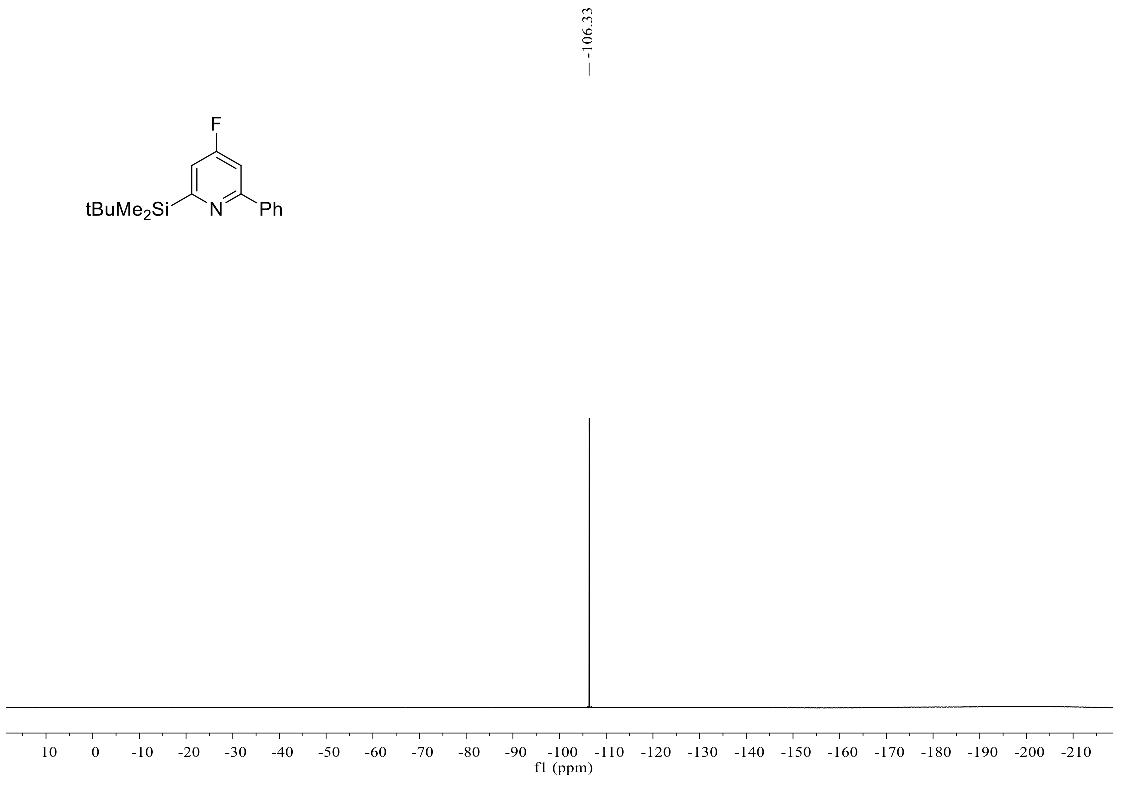

^{1}H NMR (400 MHz, $CDCl_3$) of 25

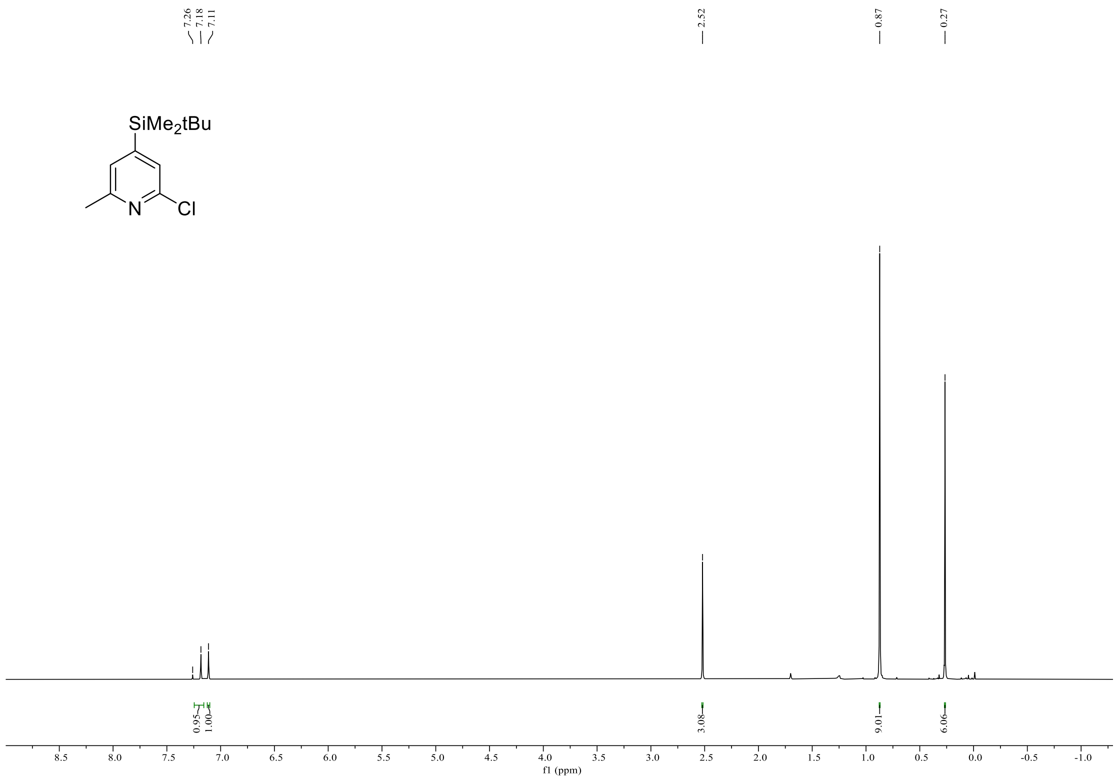

^{13}C NMR (101 MHz, $CDCl_3$) of 25

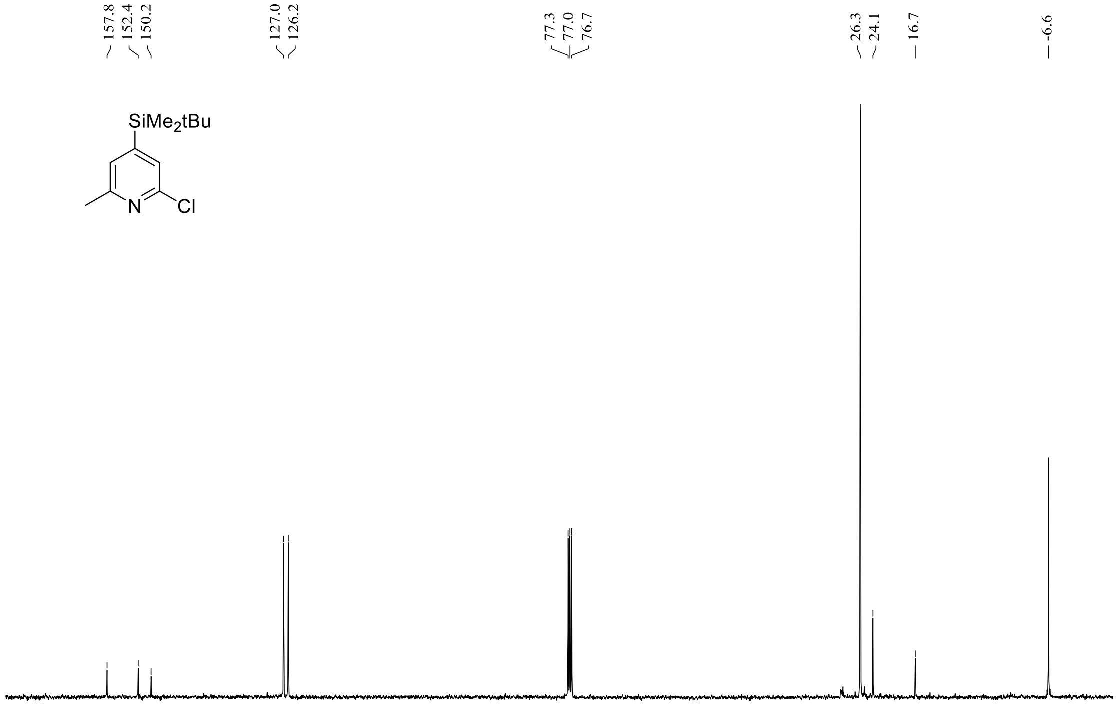

^1H NMR (400 MHz, $CDCl_3$) of 26

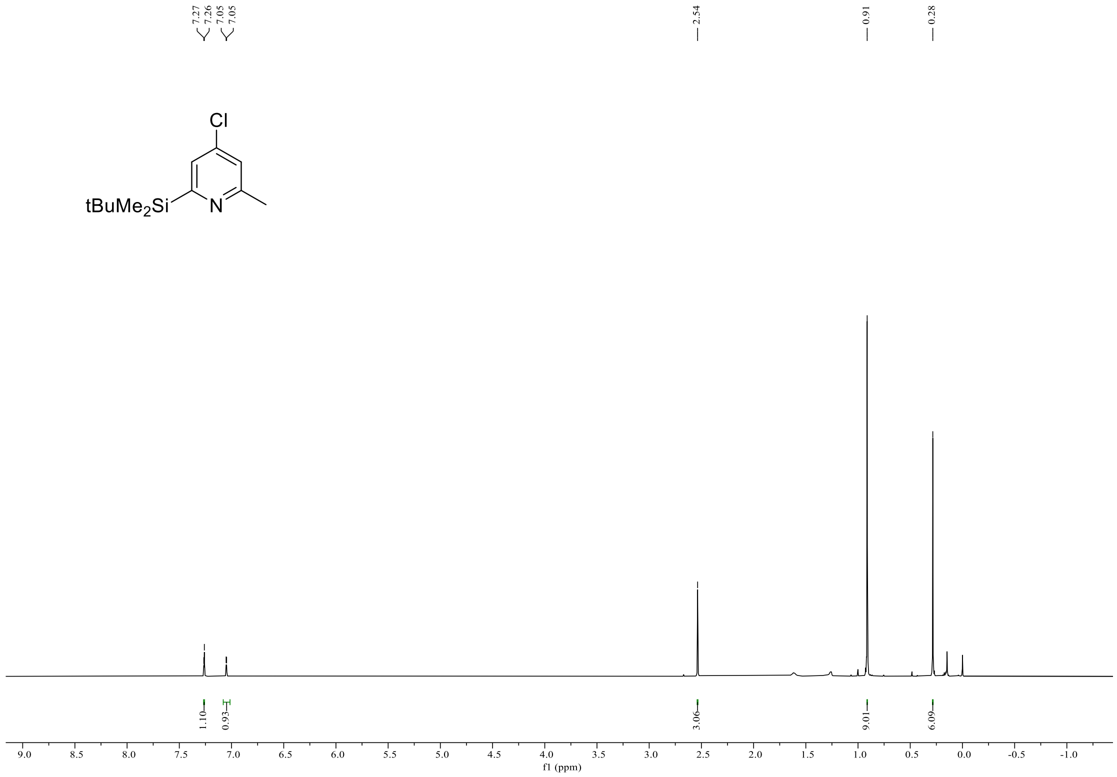

^{13}C NMR (101 MHz, $CDCl_3$) of 26

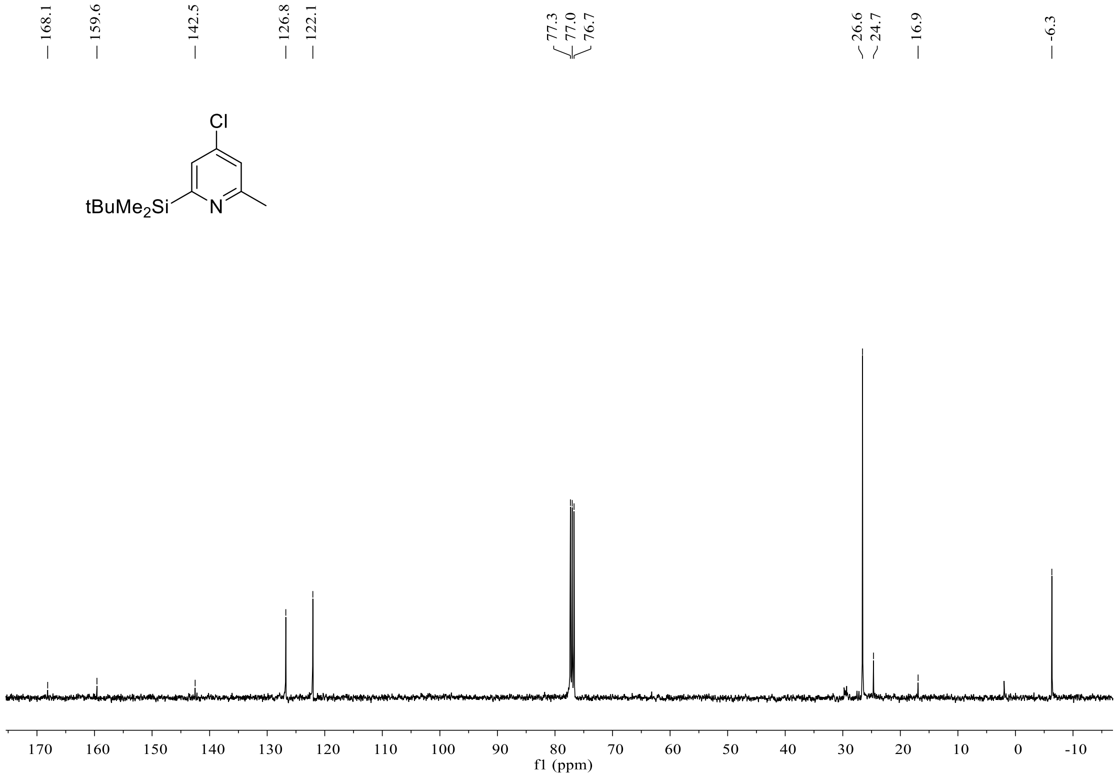

^{1}H NMR (400 MHz, CDCl$_3$) of 27

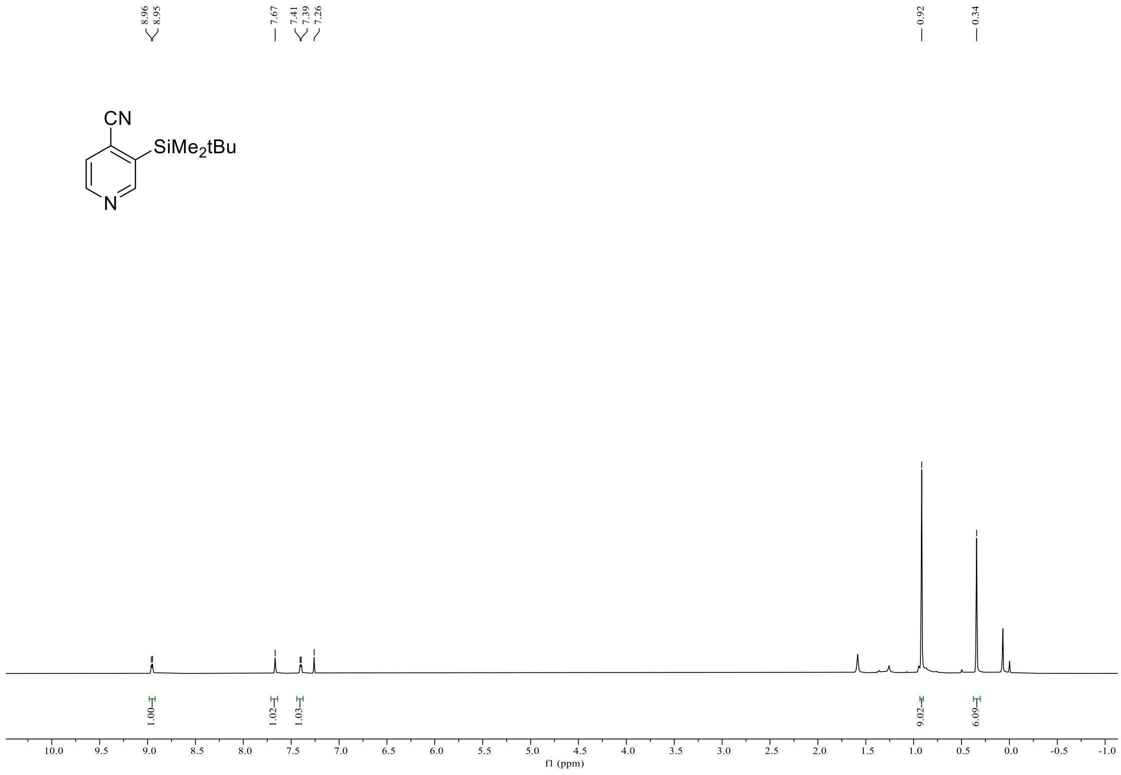

^{13}C NMR (101 MHz, CDCl$_3$) of 27

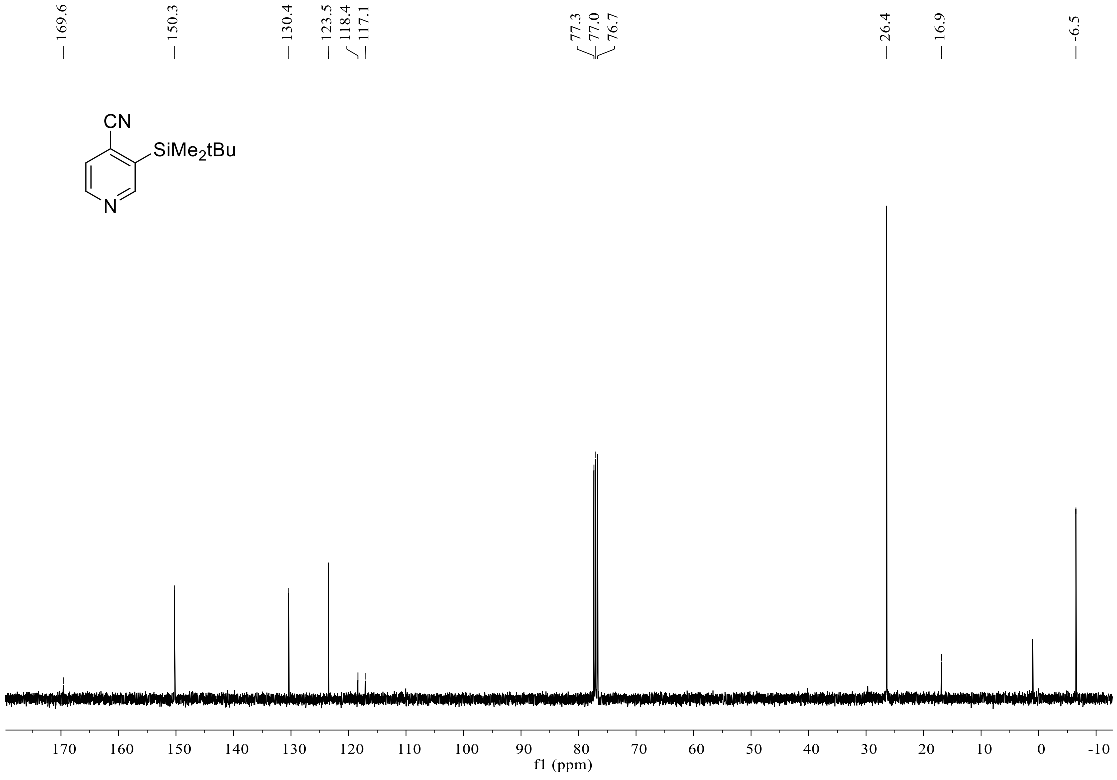

^{1}H NMR (400 MHz, $CDCl_3$) of 28

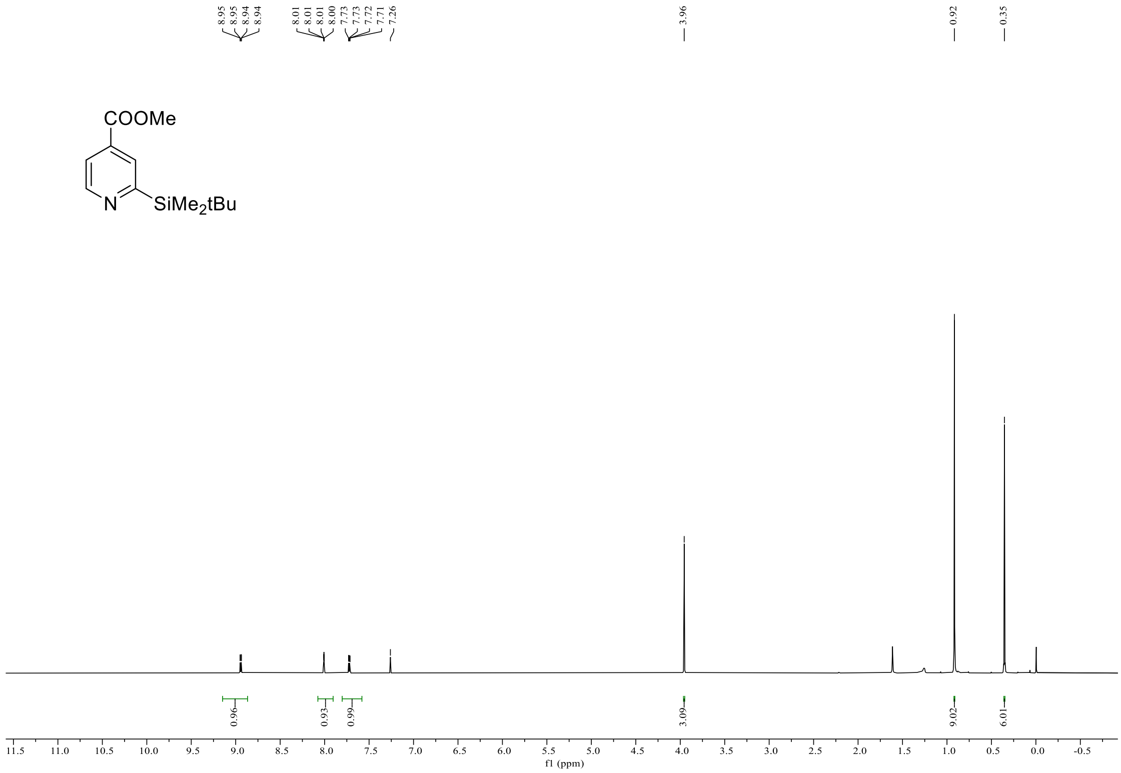

^{13}C NMR (101 MHz, $CDCl_3$) of 28

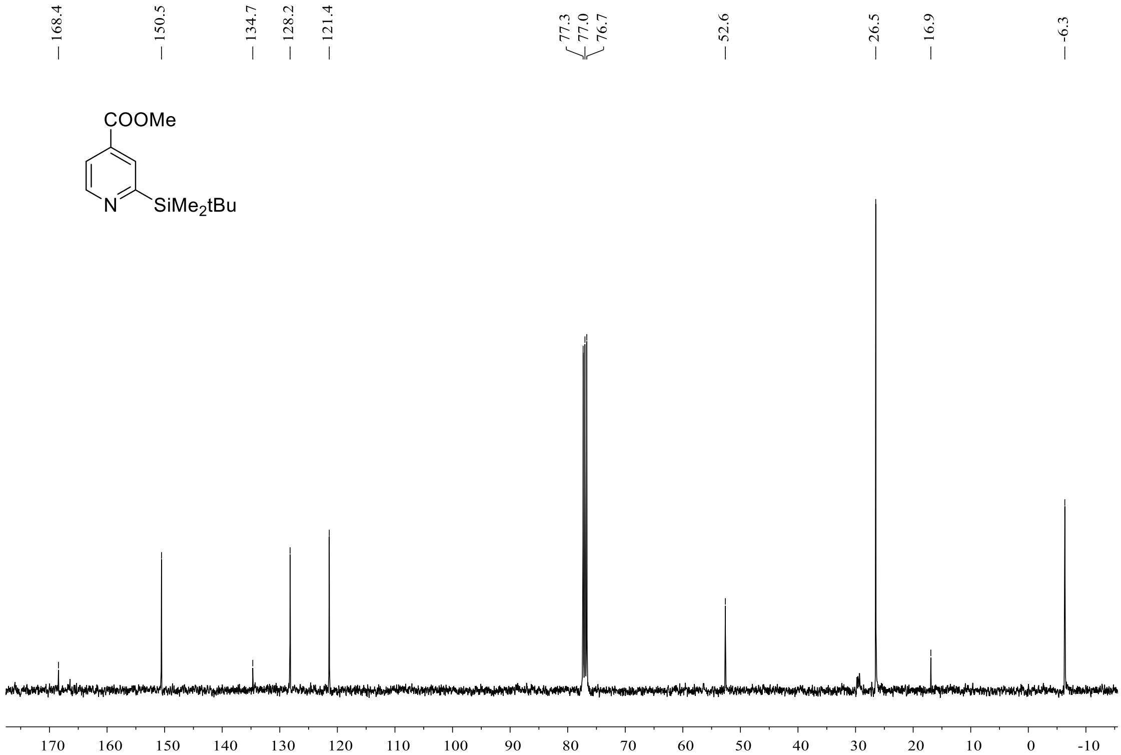

^{1}H NMR (400 MHz, $CDCl_3$) of 29

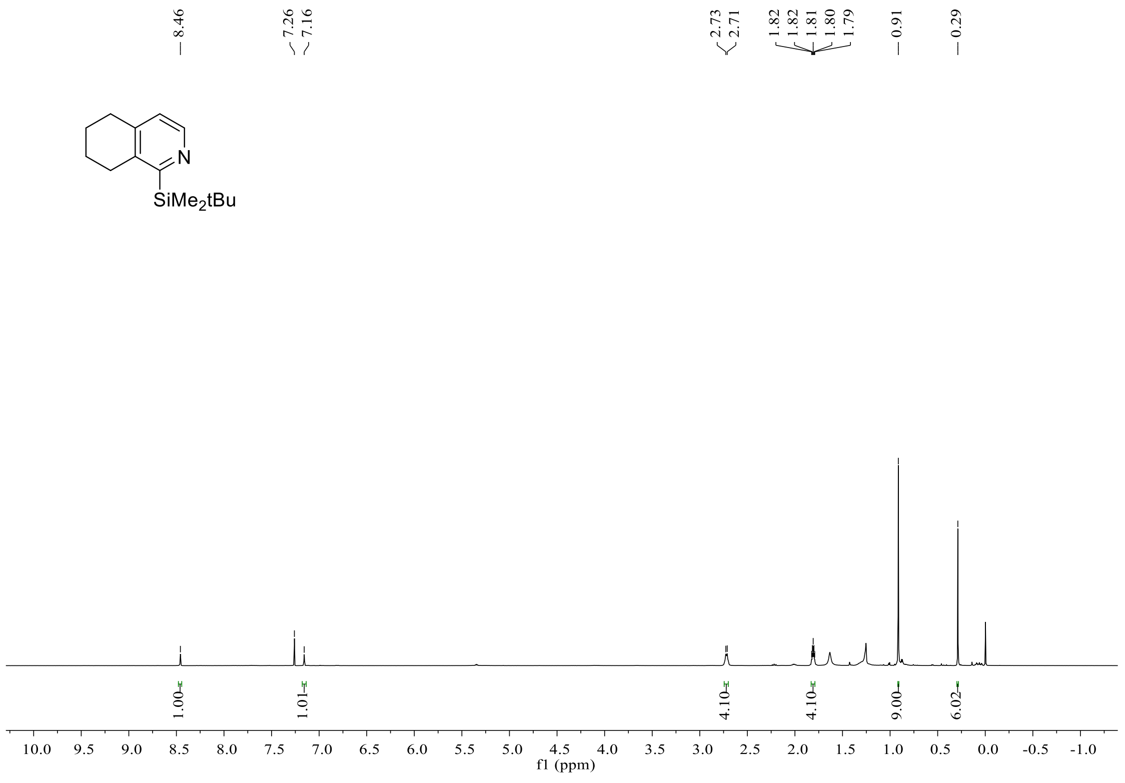

^{13}C NMR (101 MHz, $CDCl_3$) of 29

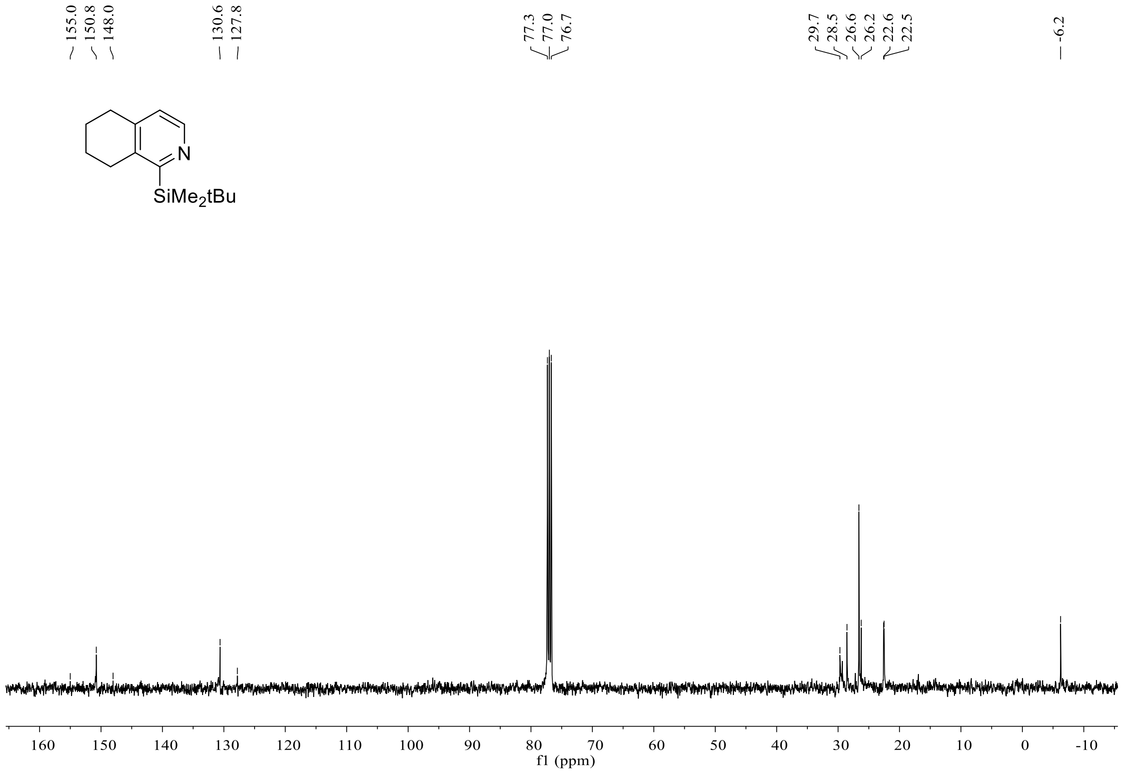

^{1}H NMR (400 MHz, CDCl$_3$) of 31

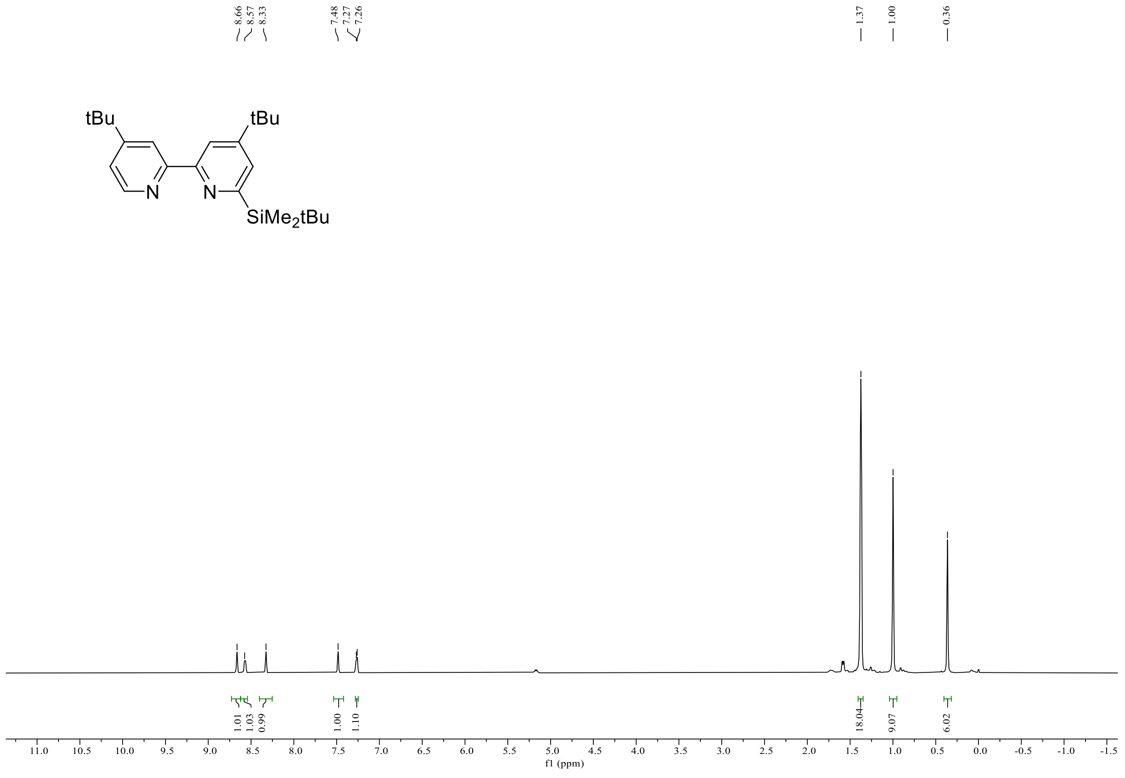

^{13}C NMR (101 MHz, CDCl$_3$) of 31

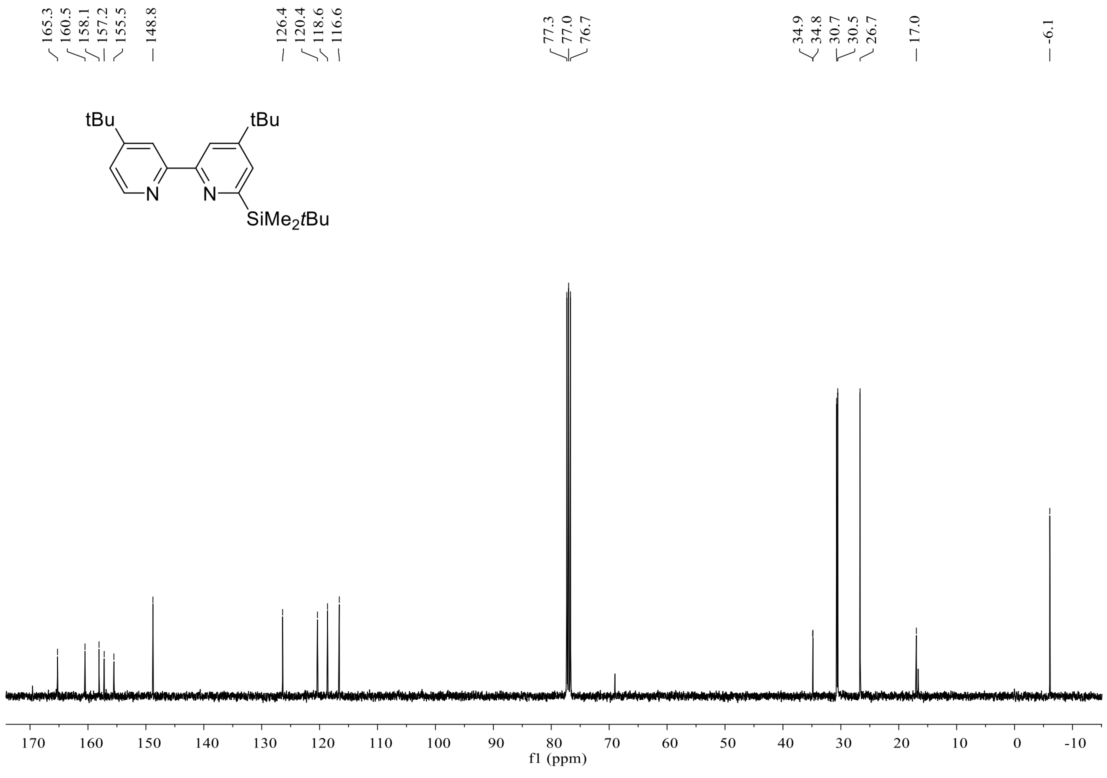

^{1}H NMR (400 MHz, $CDCl_3$) of 32

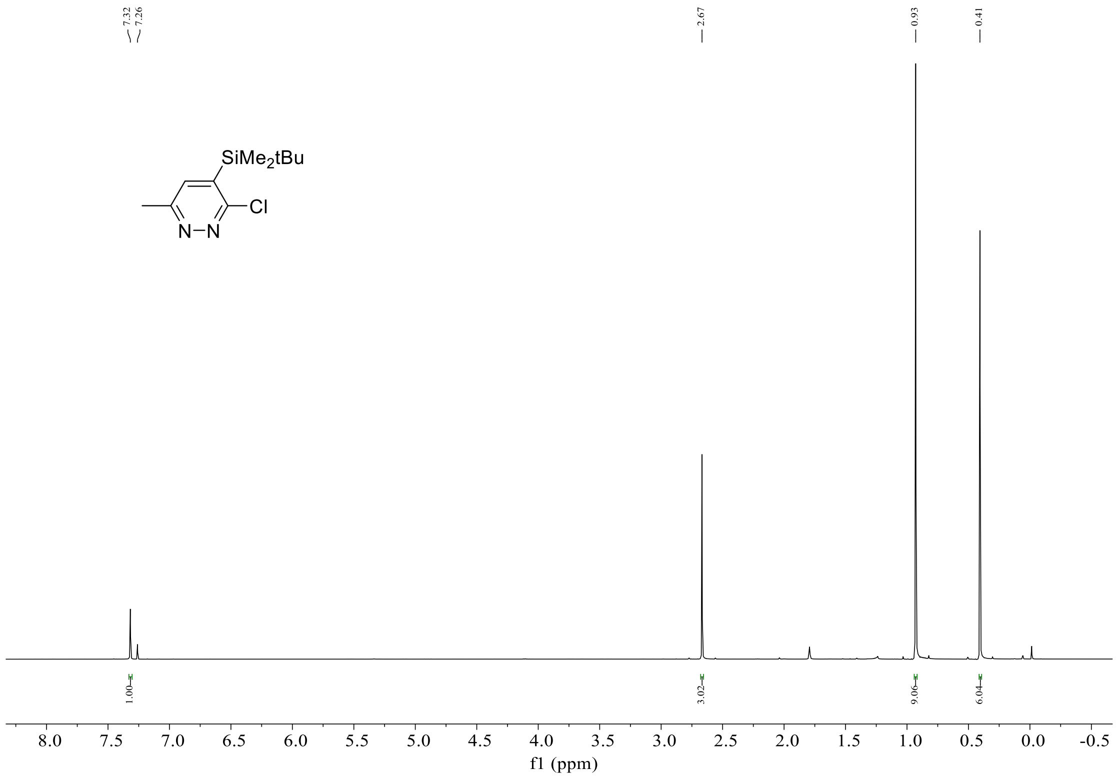

^{13}C NMR (101 MHz, $CDCl_3$) of 32

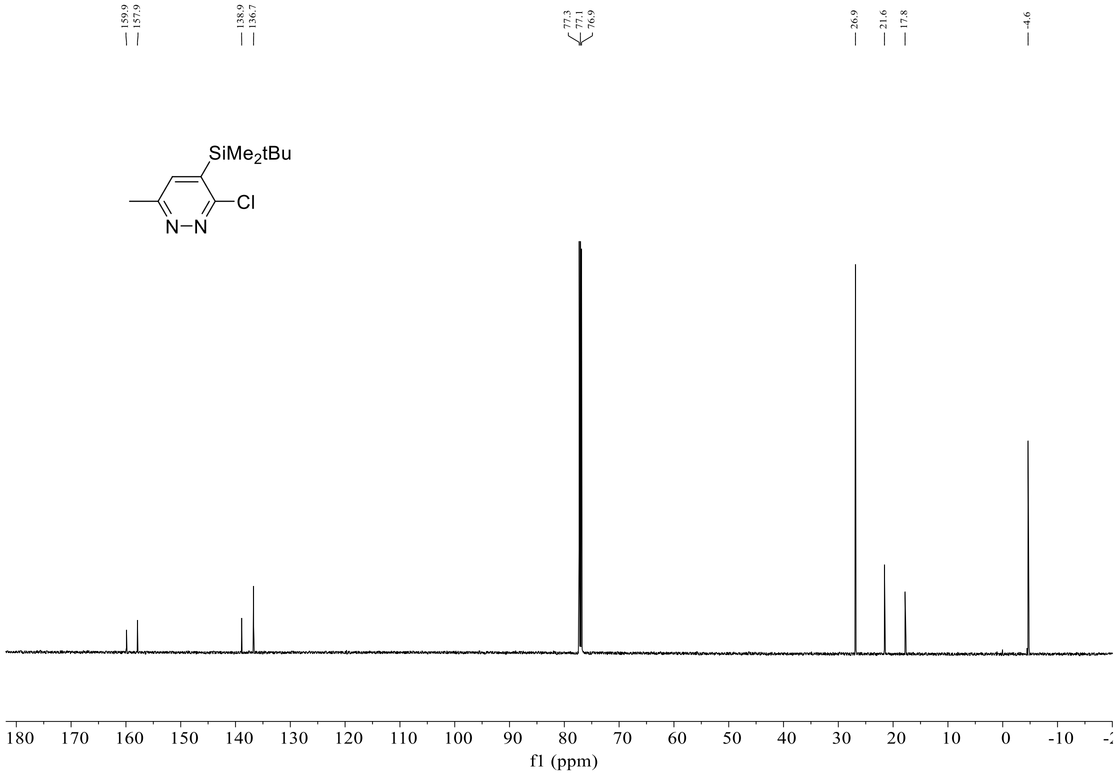

^{1}H NMR (400 MHz, $CDCl_3$) of 33

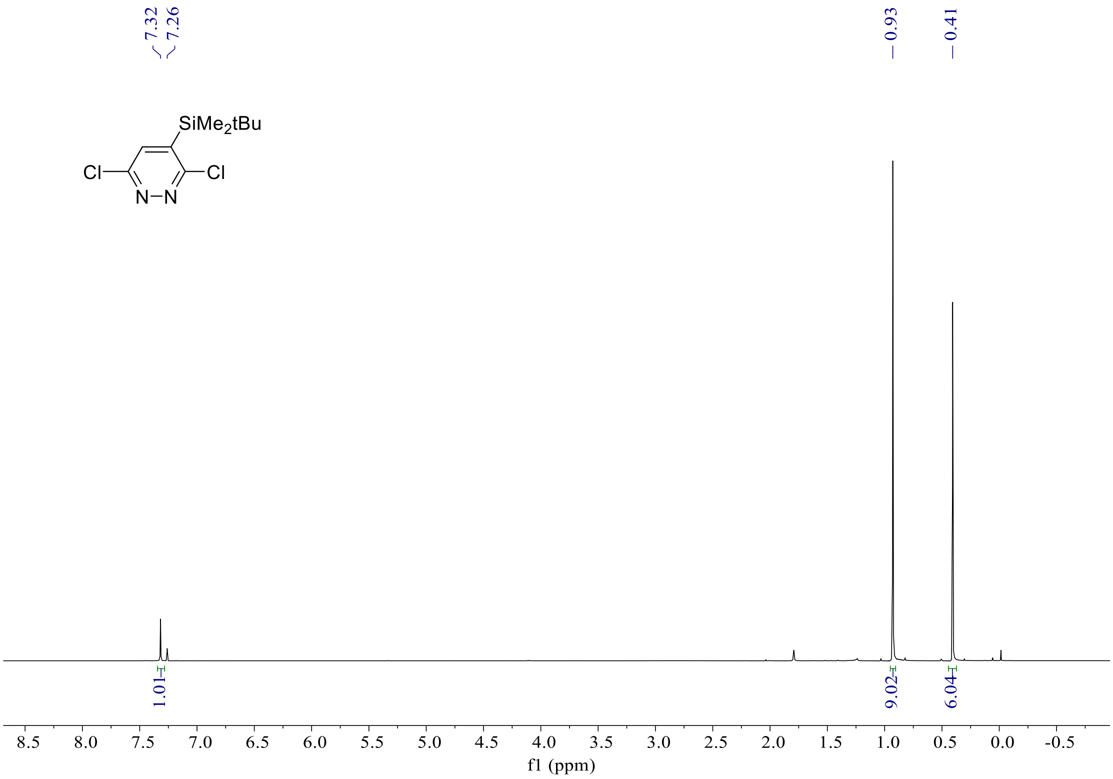

^{13}C NMR (101 MHz, $CDCl_3$) of 33

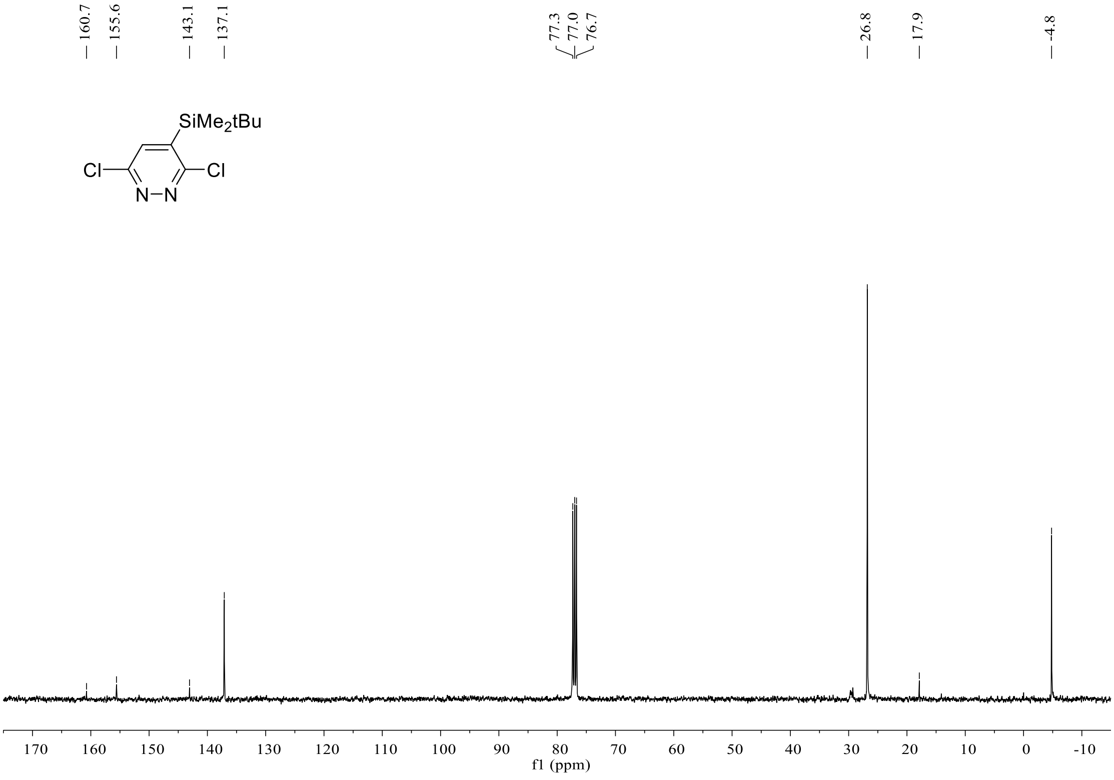

^{1}H NMR (400 MHz, $CDCl_3$) of 34

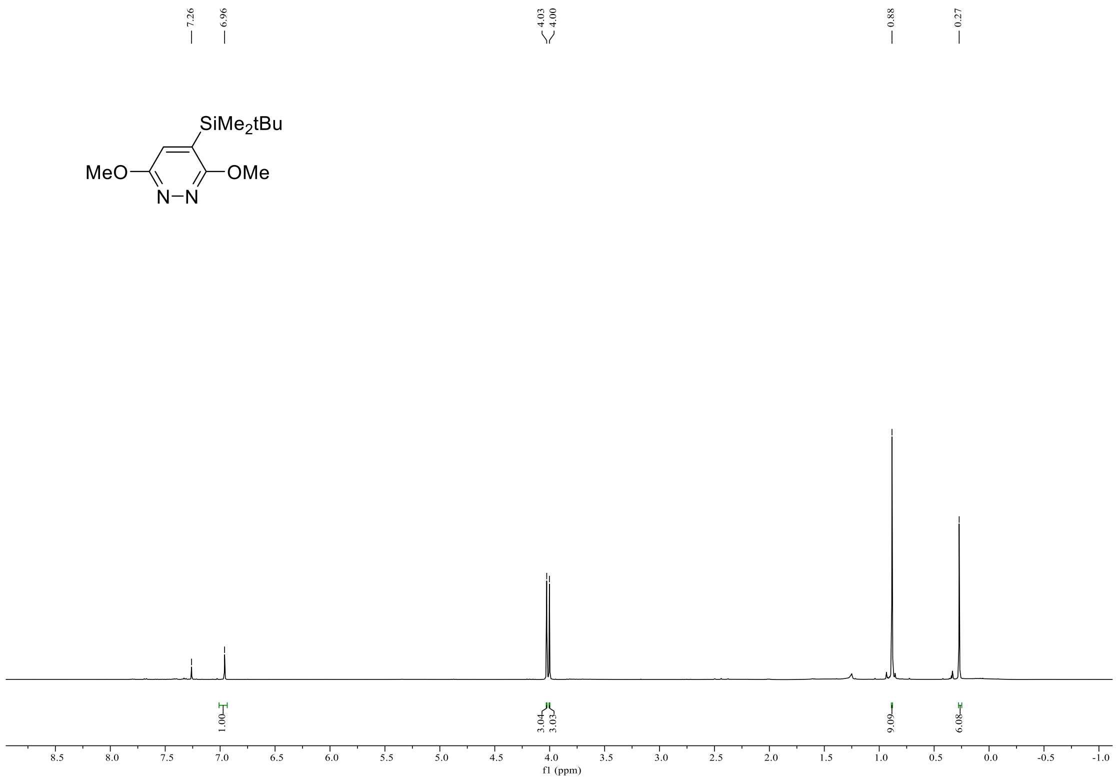

^{13}C NMR (101 MHz, $CDCl_3$) of 34

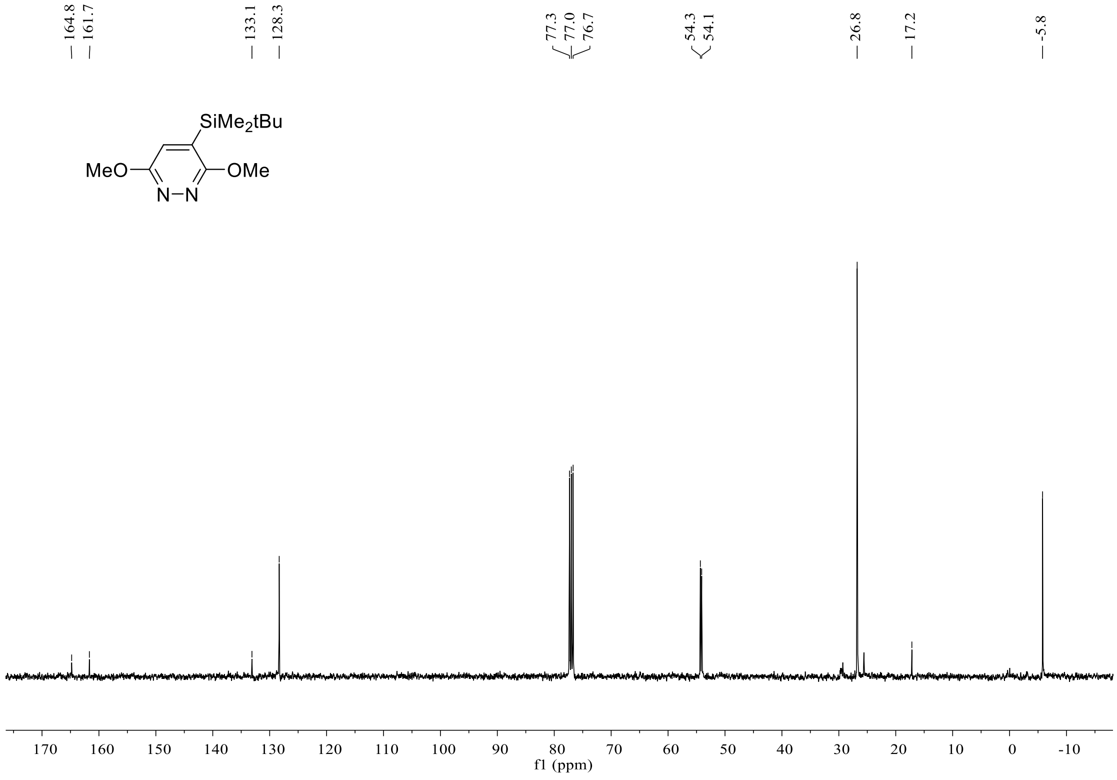

^{1}H NMR (400 MHz, CDCl$_3$) of 35

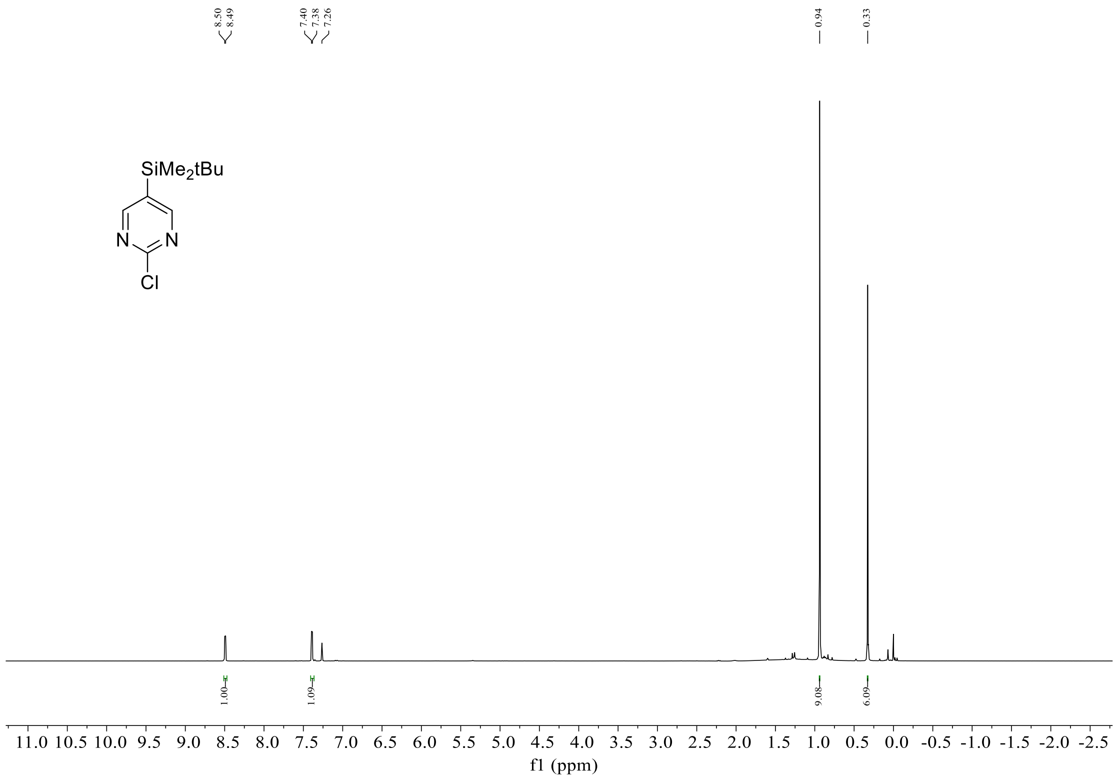

^{13}C NMR (101 MHz, CDCl$_3$) of 35

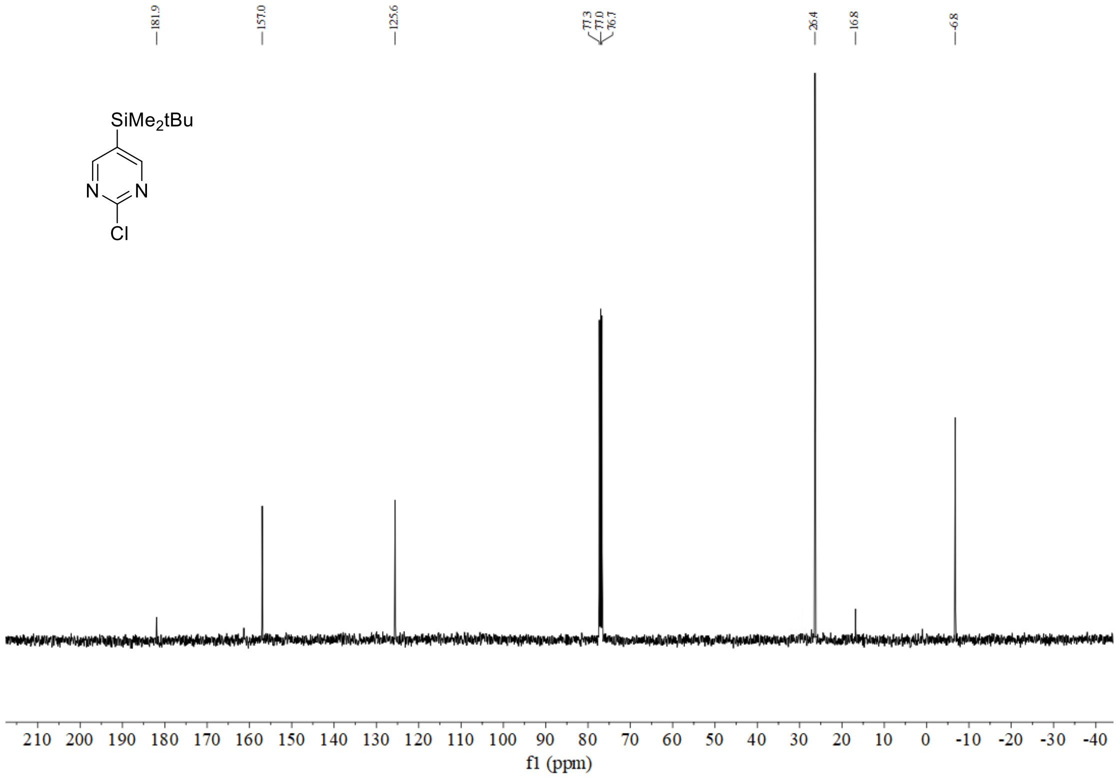

^1H NMR (400 MHz, $CDCl_3$) of 36

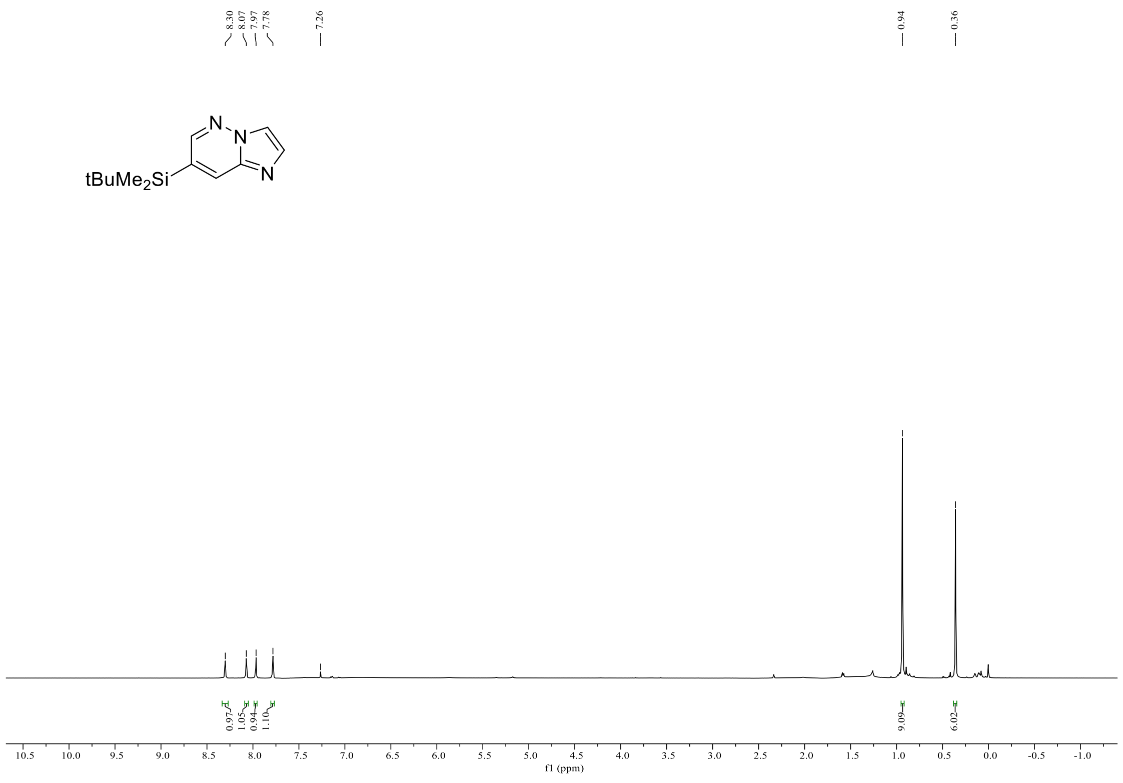

^{13}C NMR (101 MHz, $CDCl_3$) of 36

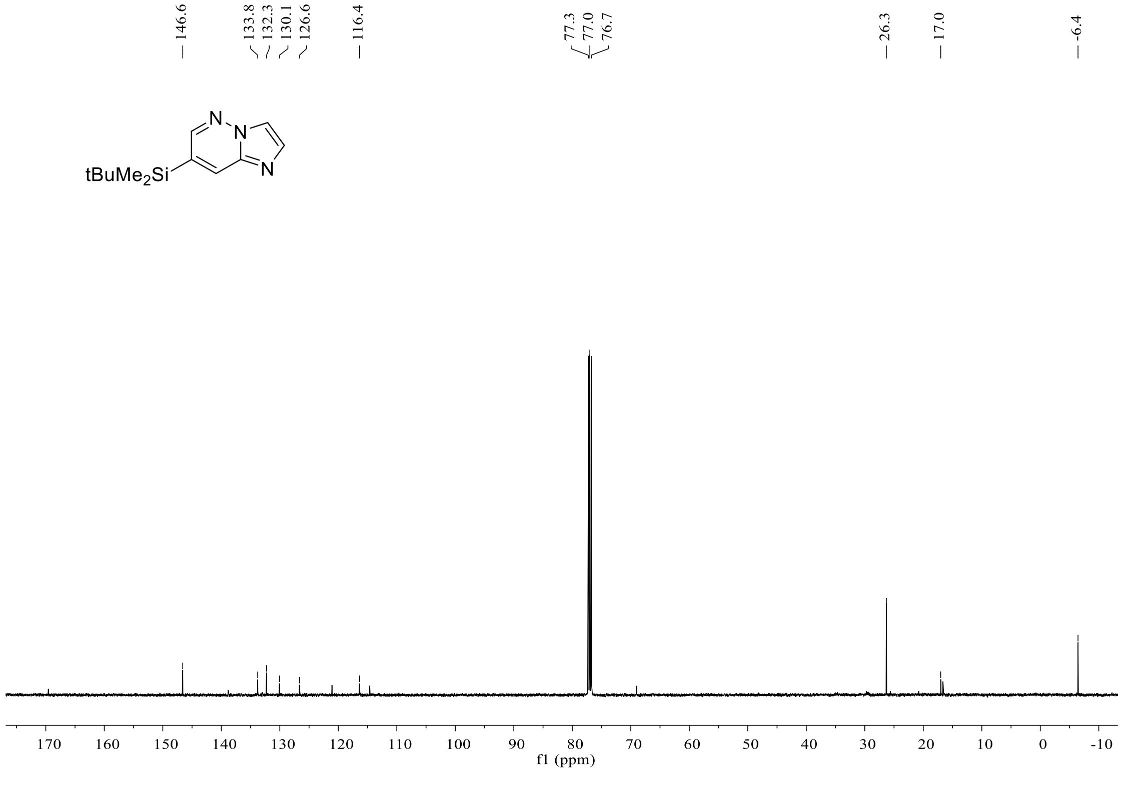

^{1}H NMR (400 MHz, $CDCl_3$) of 36'

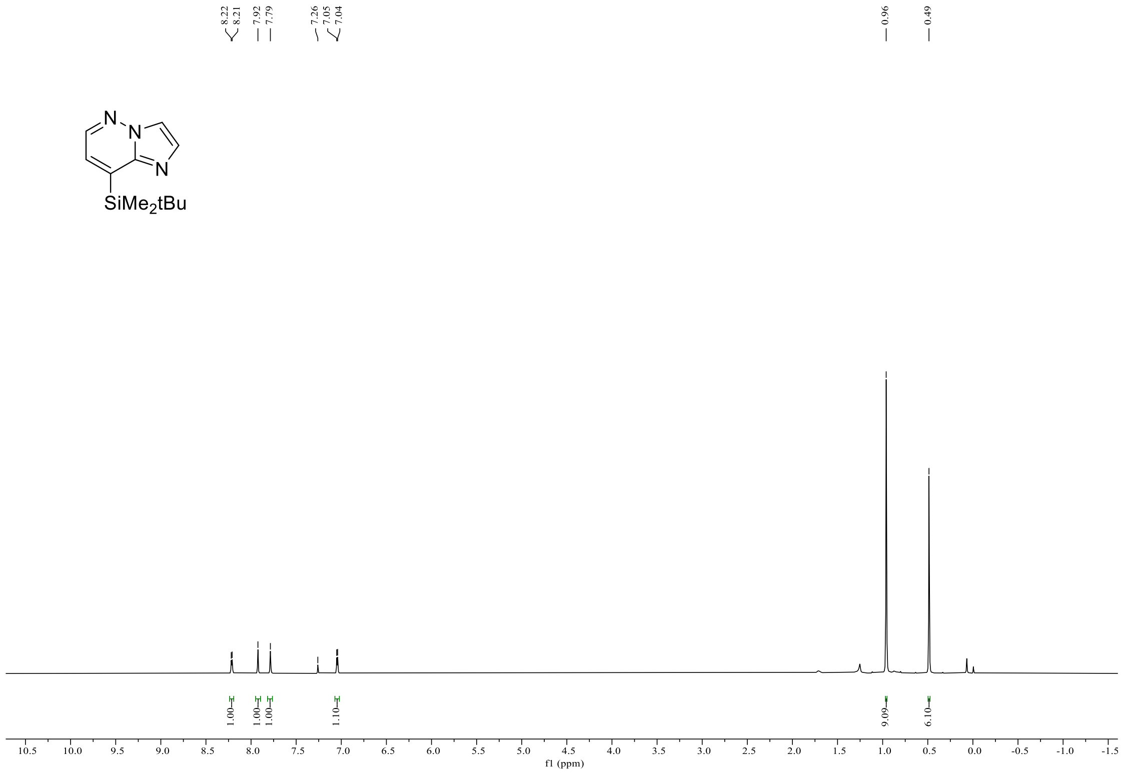

^{13}C NMR (101 MHz, $CDCl_3$) of 36'

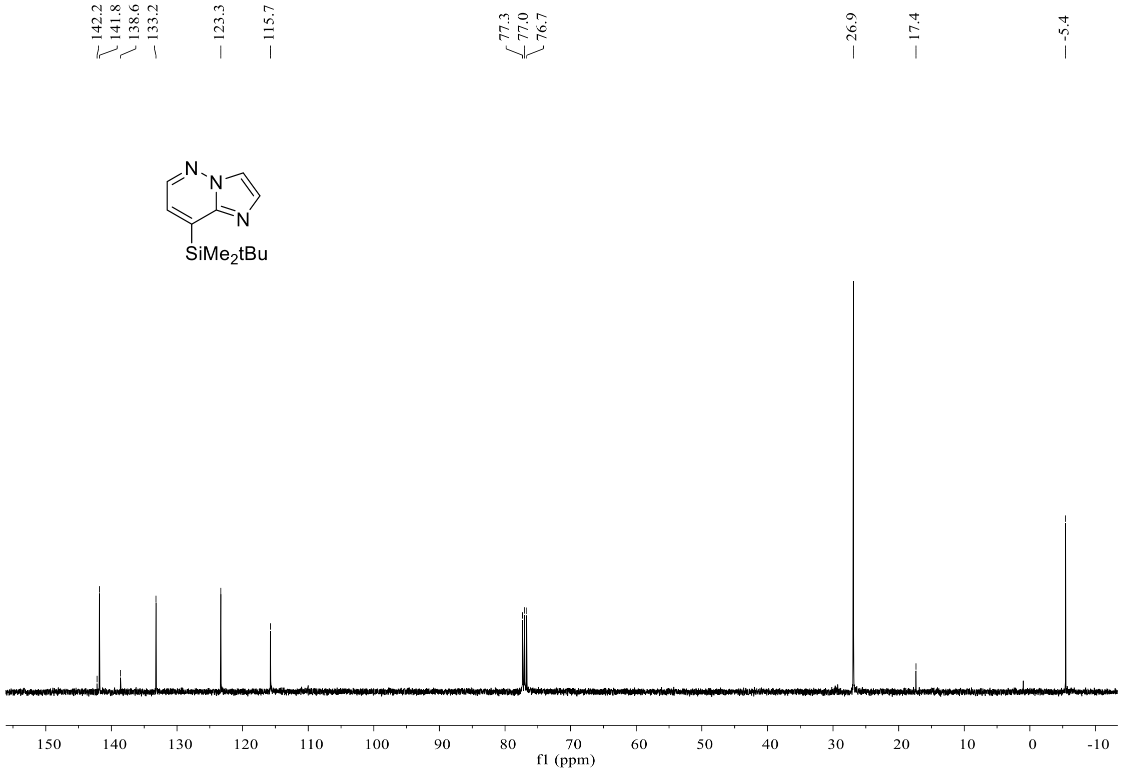

^1H NMR (400 MHz, $CDCl_3$) of 37

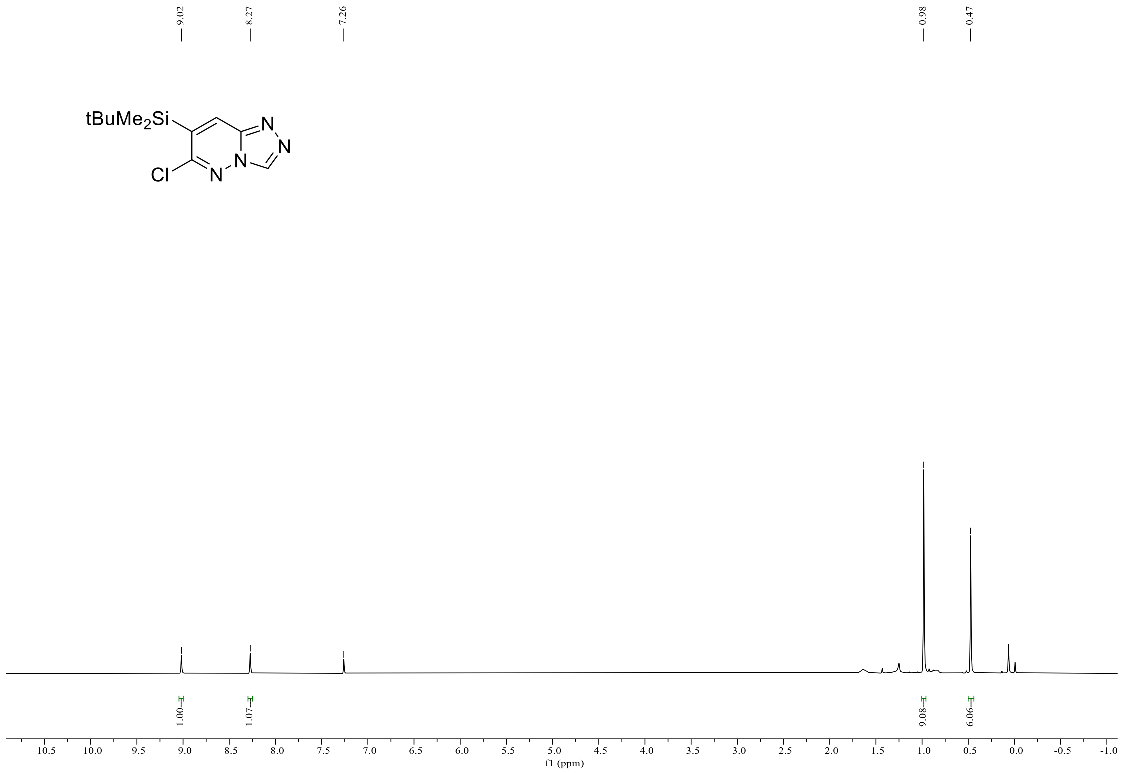

^{13}C NMR (101 MHz, $CDCl_3$) of 37

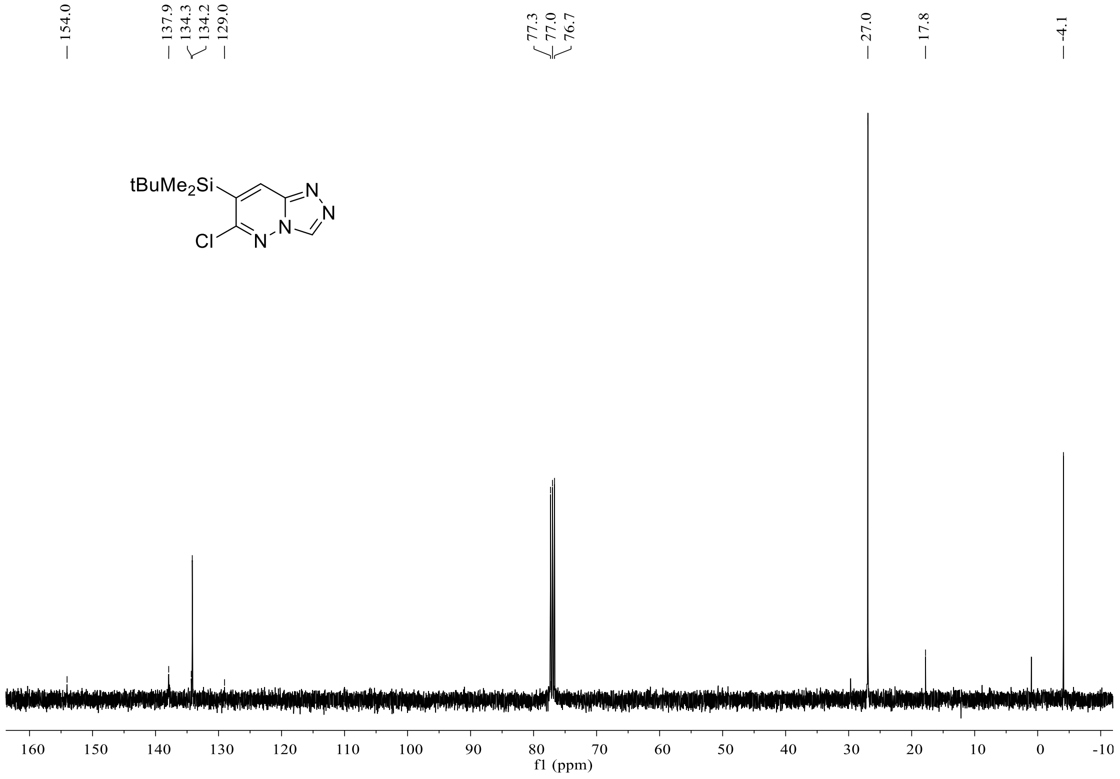

^{1}H NMR (400 MHz, $CDCl_3$) of 37'

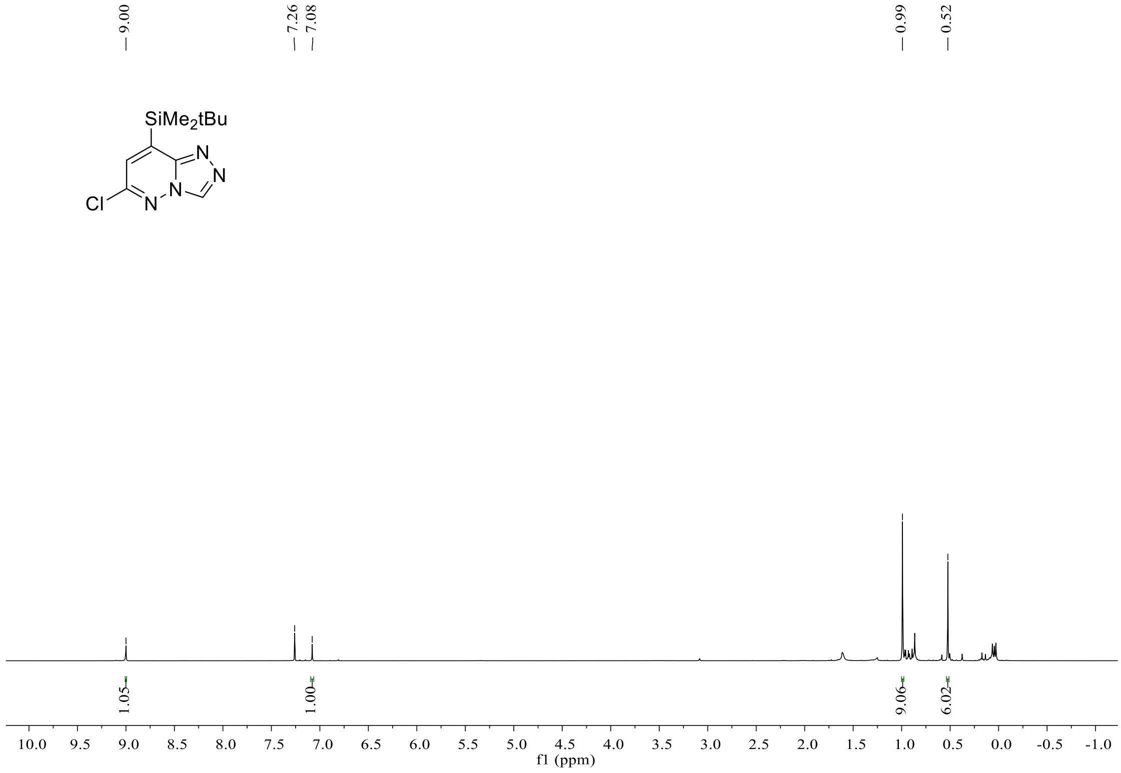

^{13}C NMR (101 MHz, $CDCl_3$) of 37'

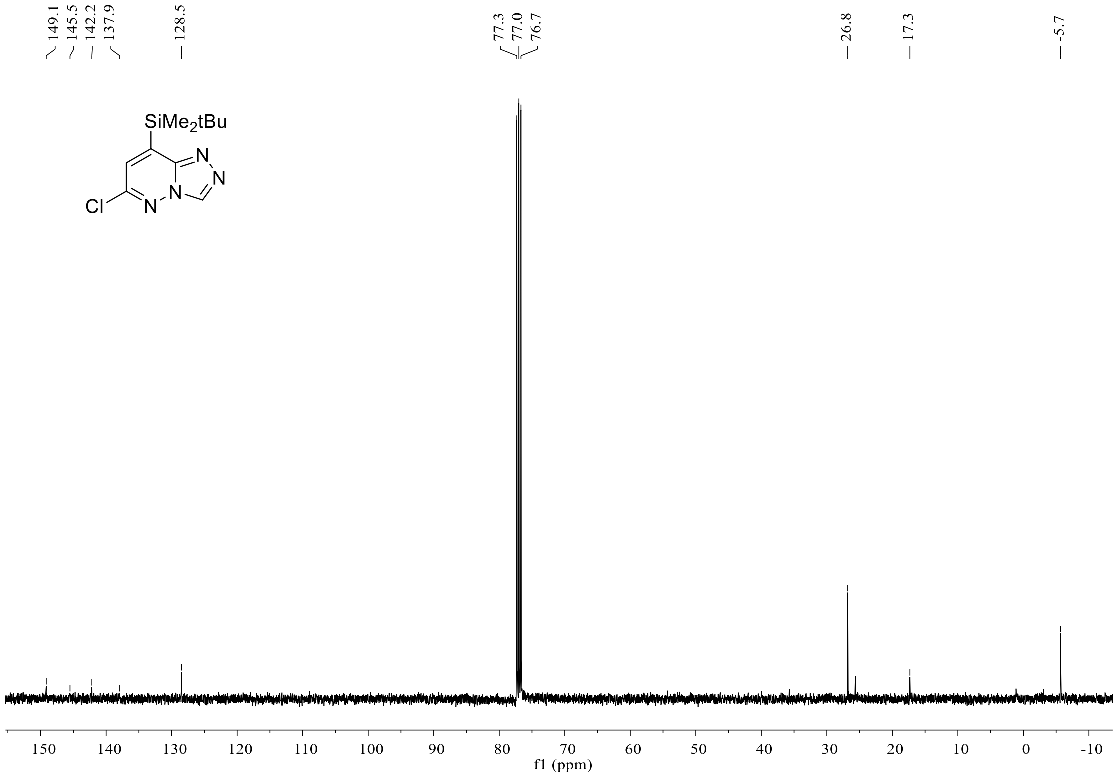

^{1}H NMR (400 MHz, $CDCl_3$) of 38

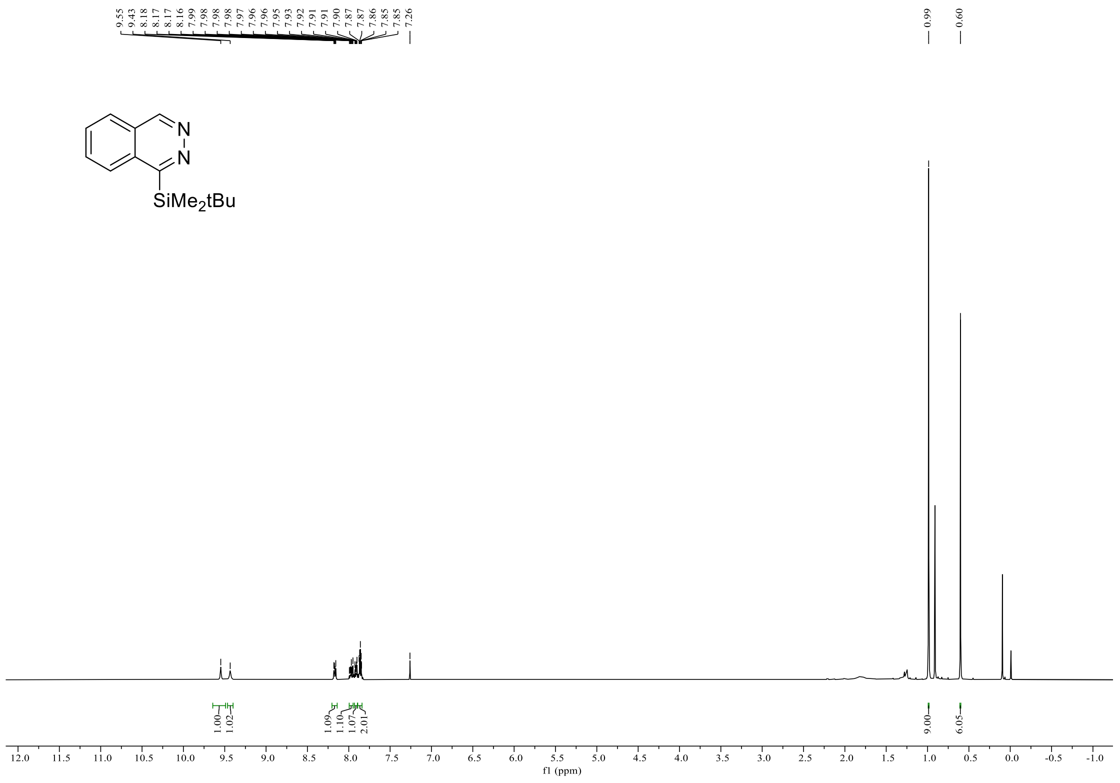

^{13}C NMR (101 MHz, $CDCl_3$) of 38

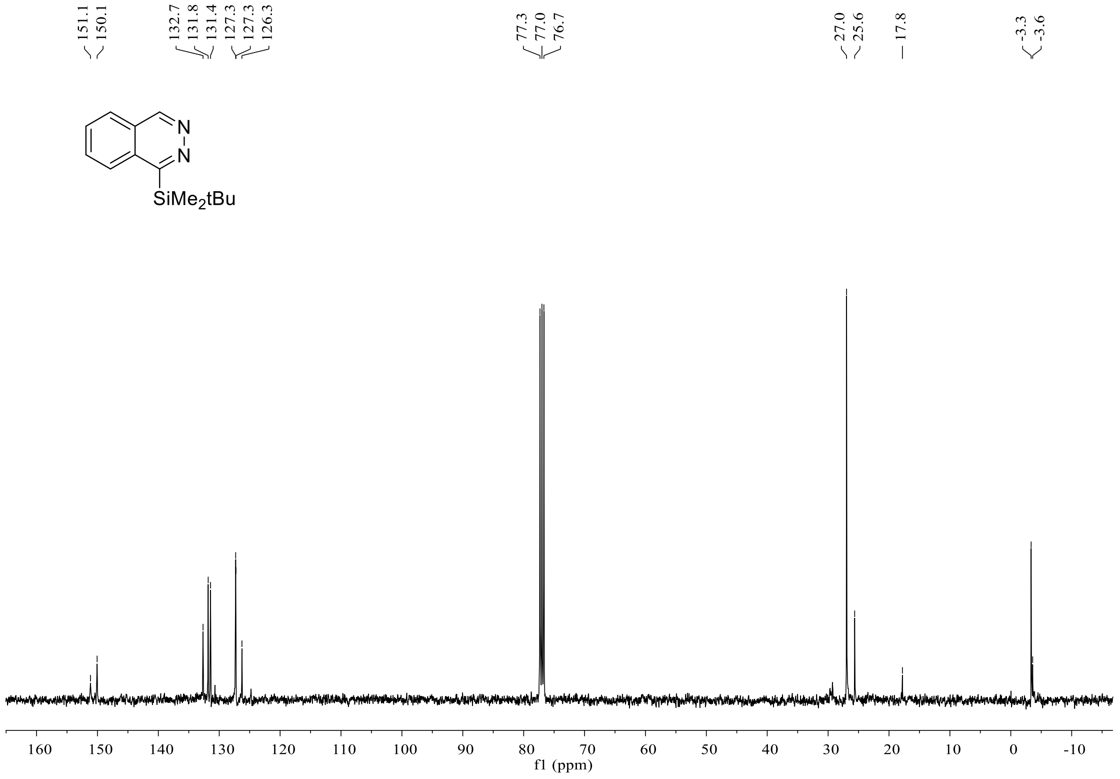

^{1}H NMR (400 MHz, $CDCl_3$) of 39

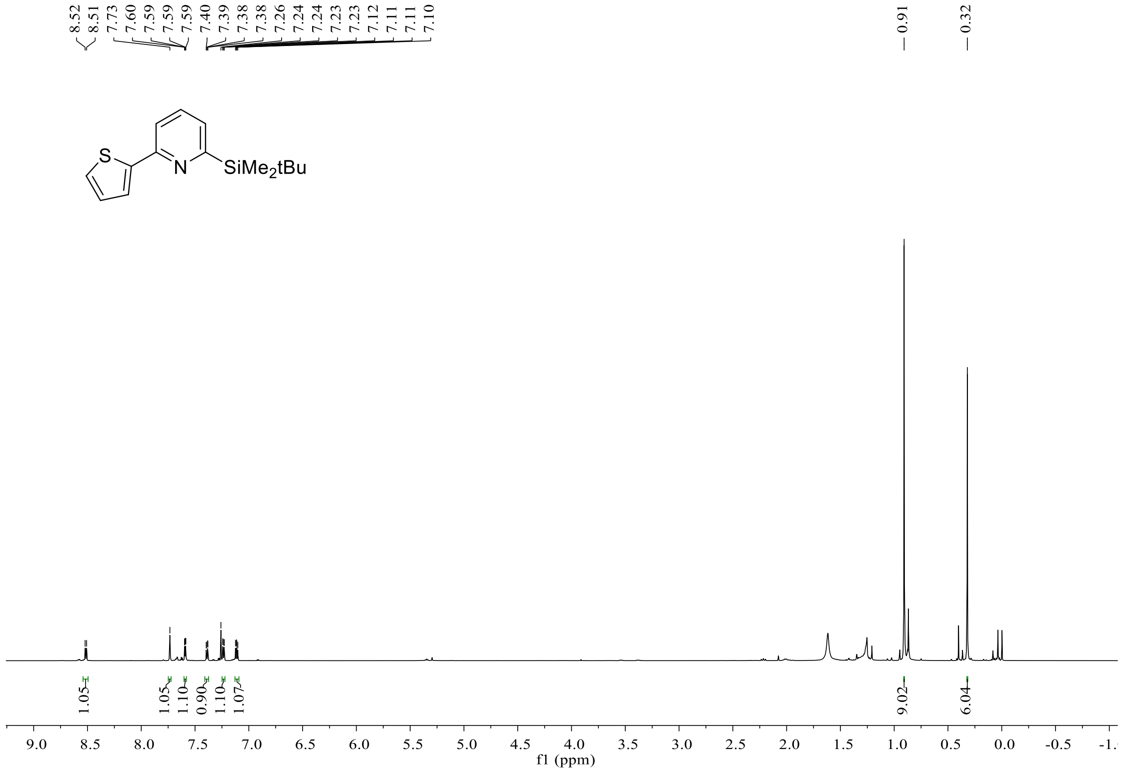

^{13}C NMR (101 MHz, $CDCl_3$) of 39

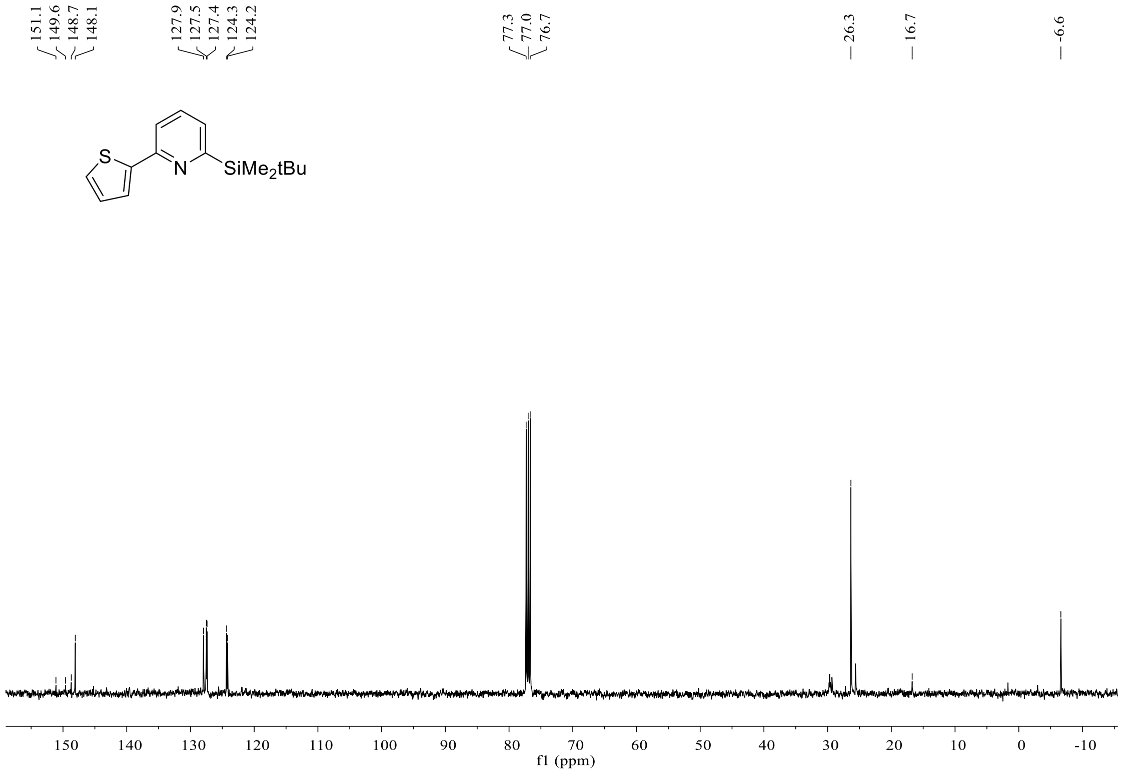

^{1}H NMR (400 MHz, $CDCl_3$) of 40

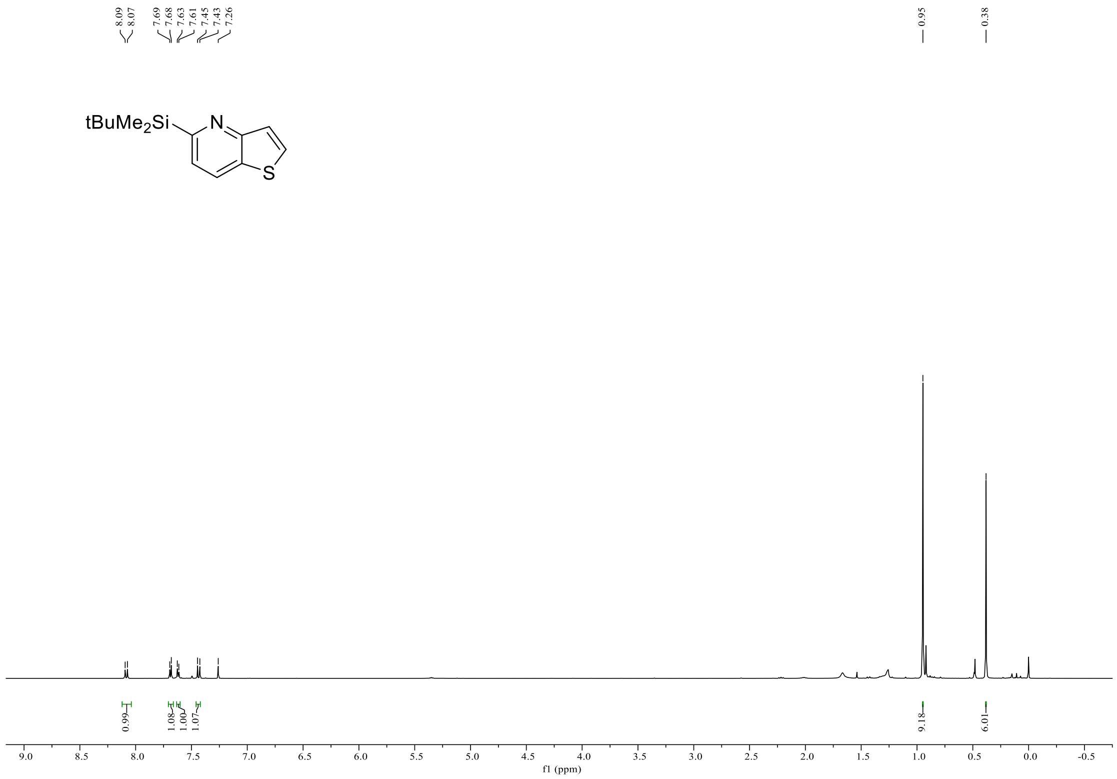

^{13}C NMR (101 MHz, $CDCl_3$) of 40

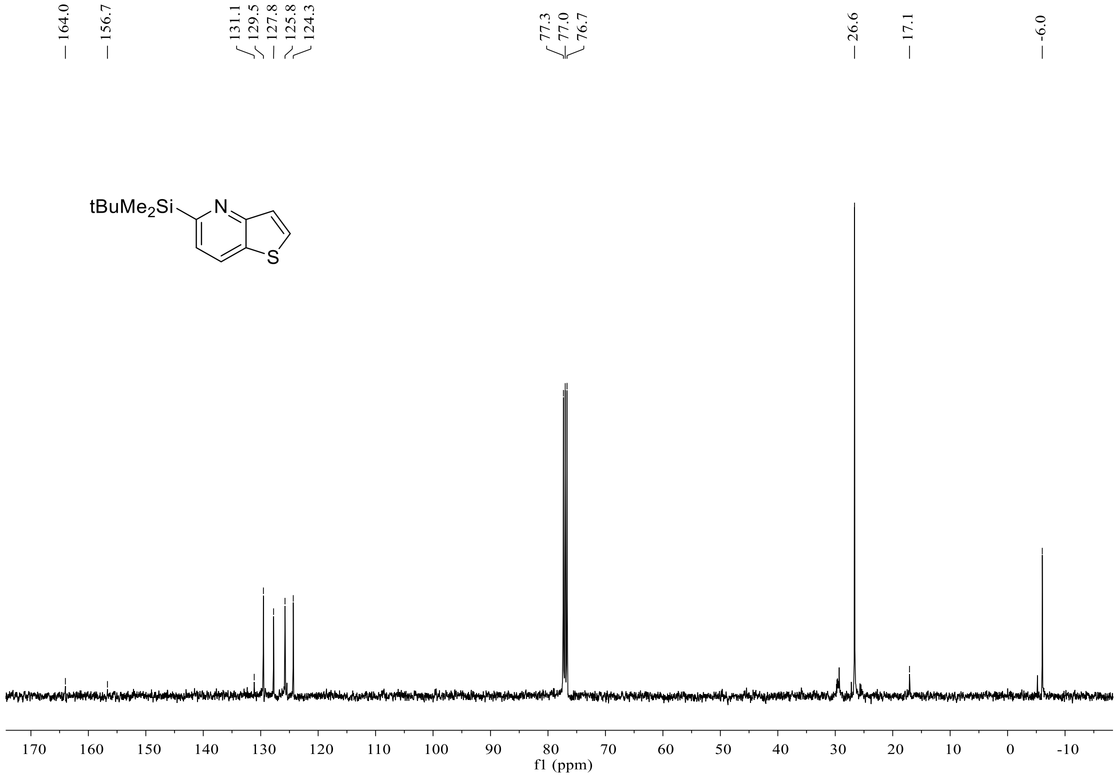

^{1}H NMR (400 MHz, $CDCl_3$) of 41

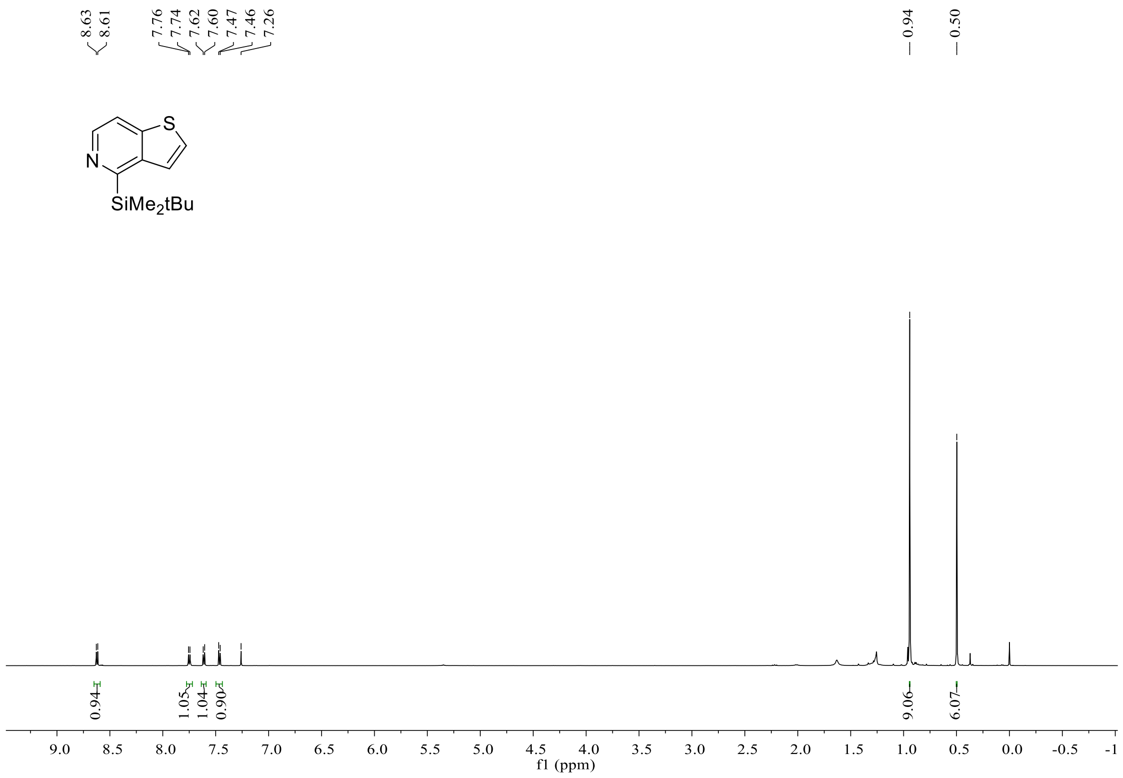

^{13}C NMR (101 MHz, $CDCl_3$) of 41

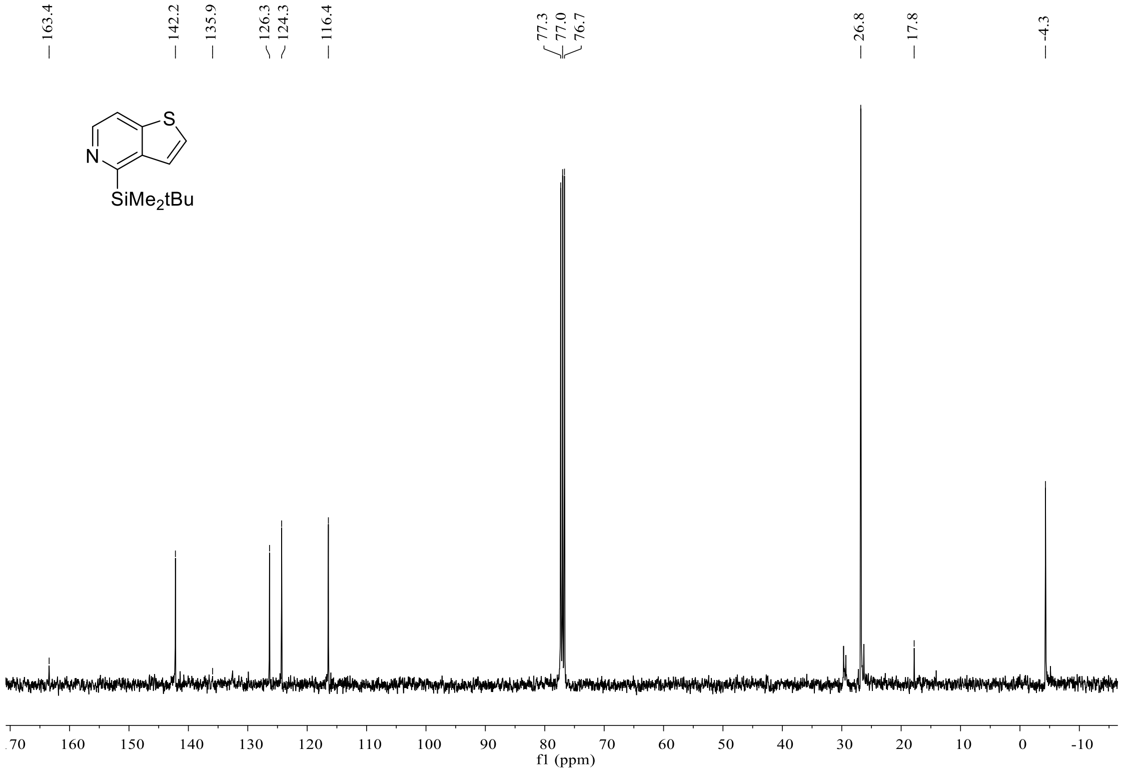

^{1}H NMR (400 MHz, $CDCl_3$) of 42

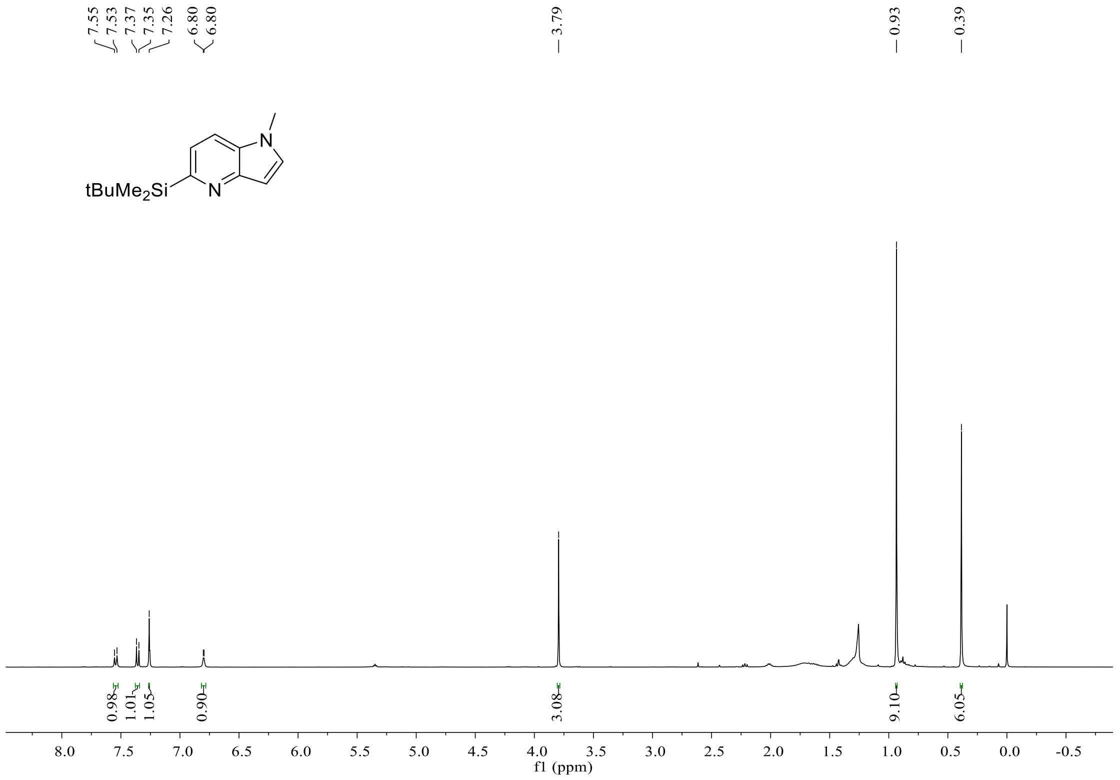

^{13}C NMR (101 MHz, $CDCl_3$) of 42

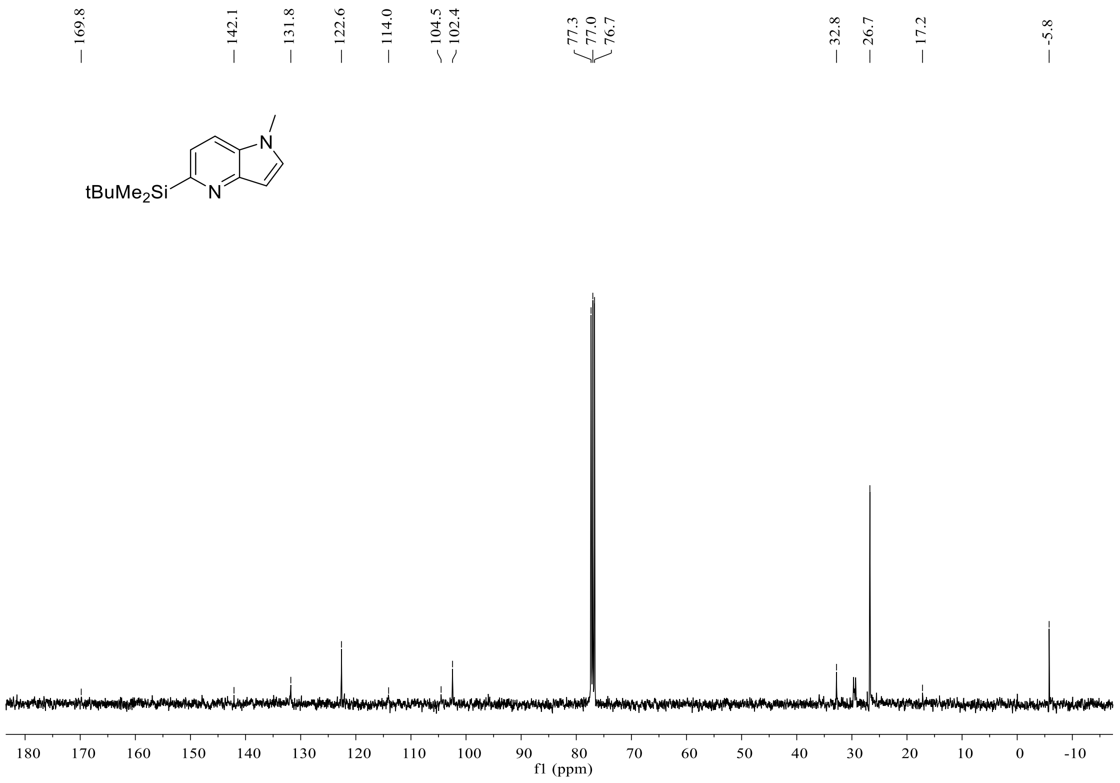

^{1}H NMR (400 MHz, $CDCl_3$) of 43

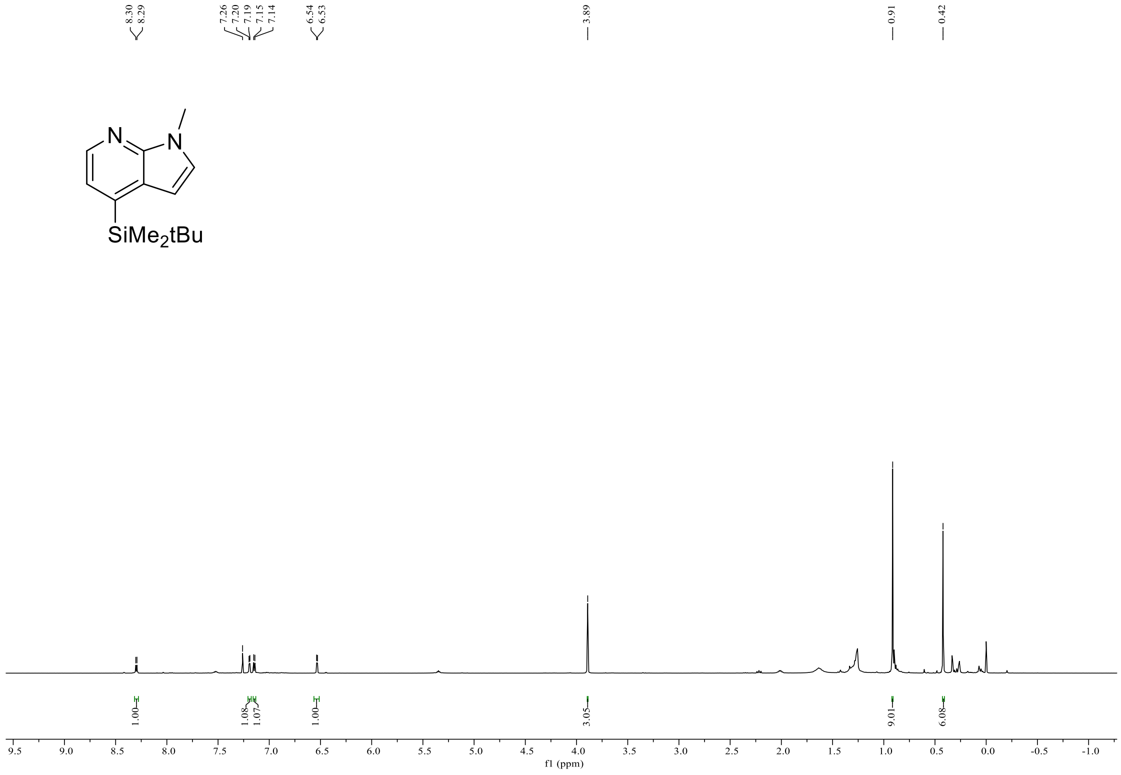

^{13}C NMR (101 MHz, $CDCl_3$) of 43

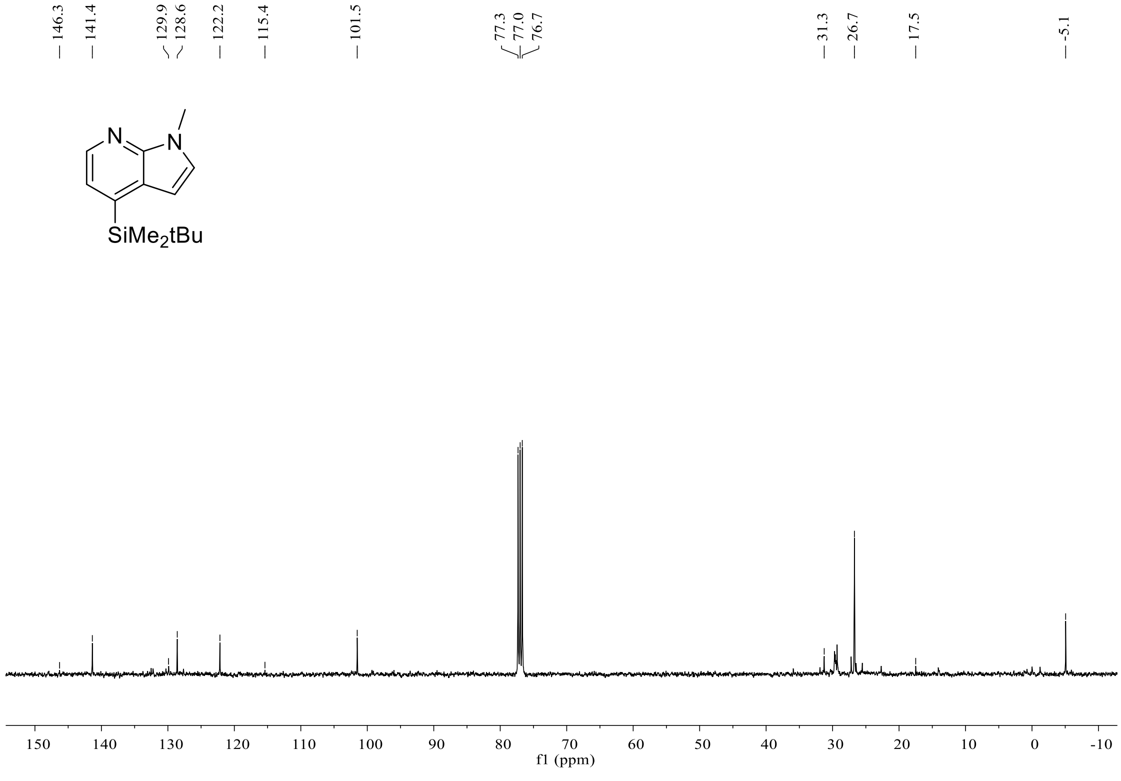

^{1}H NMR (400 MHz, $CDCl_3$) of 44

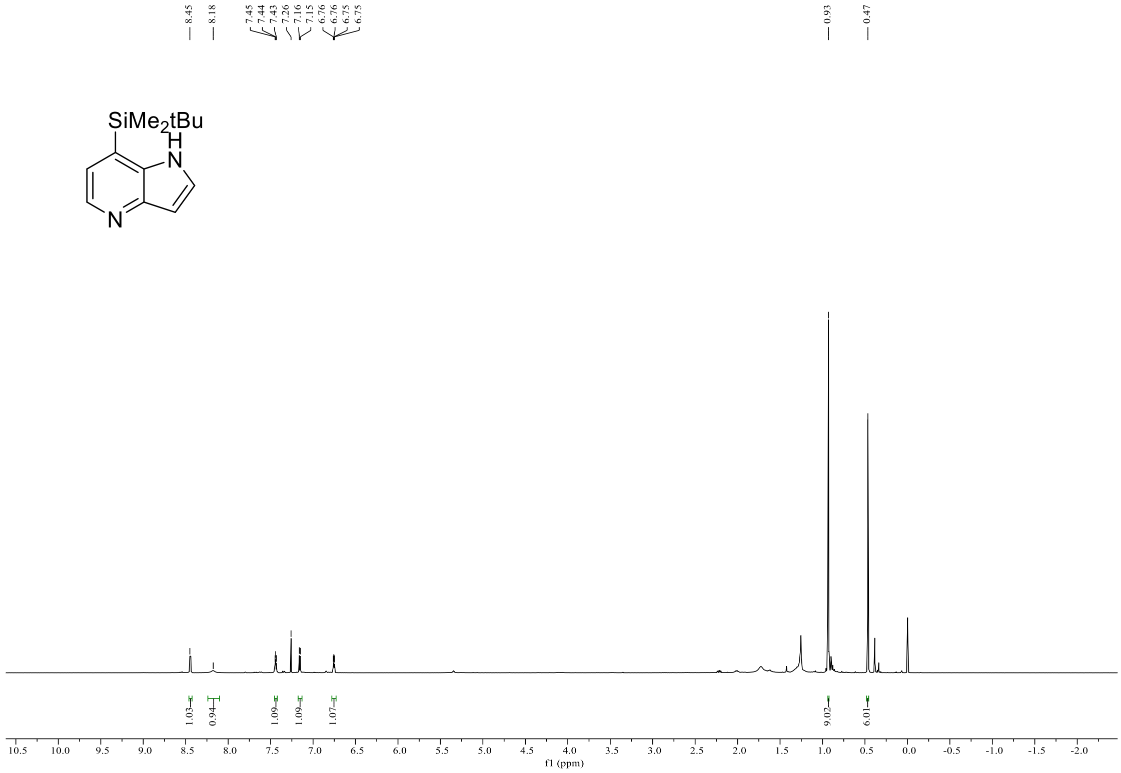

^{13}C NMR (101 MHz, $CDCl_3$) of 44

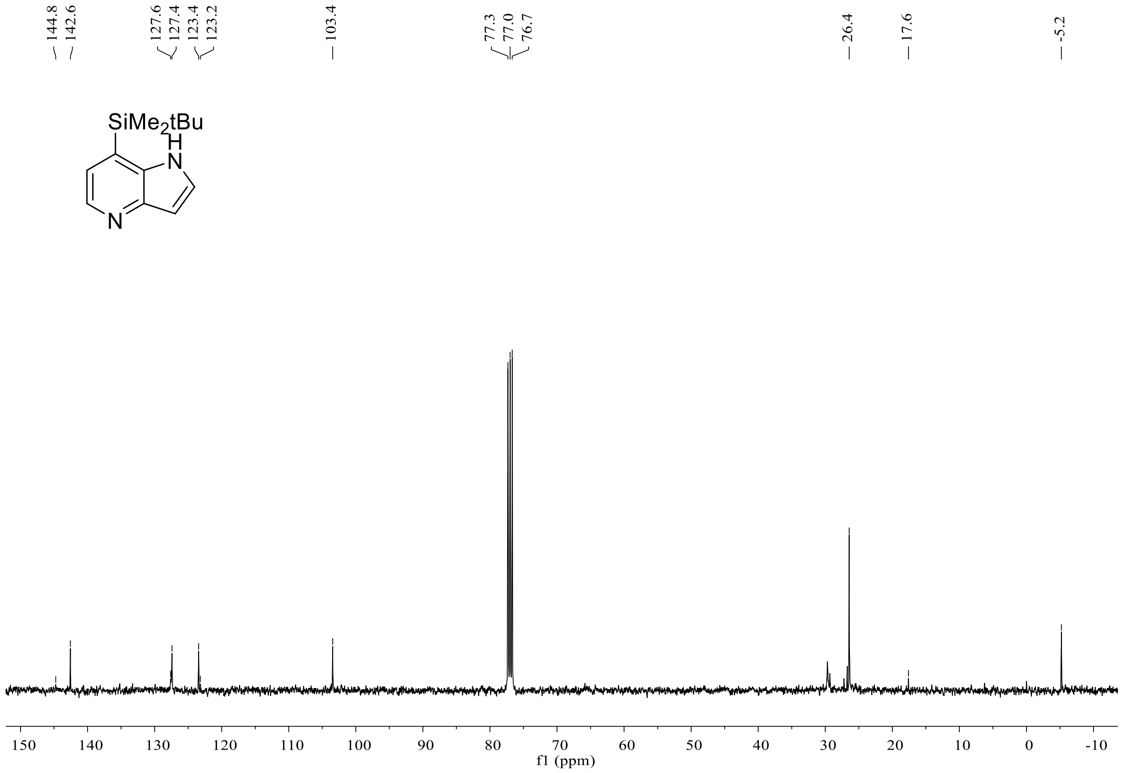

^{1}H NMR (400 MHz, $CDCl_3$) of 45

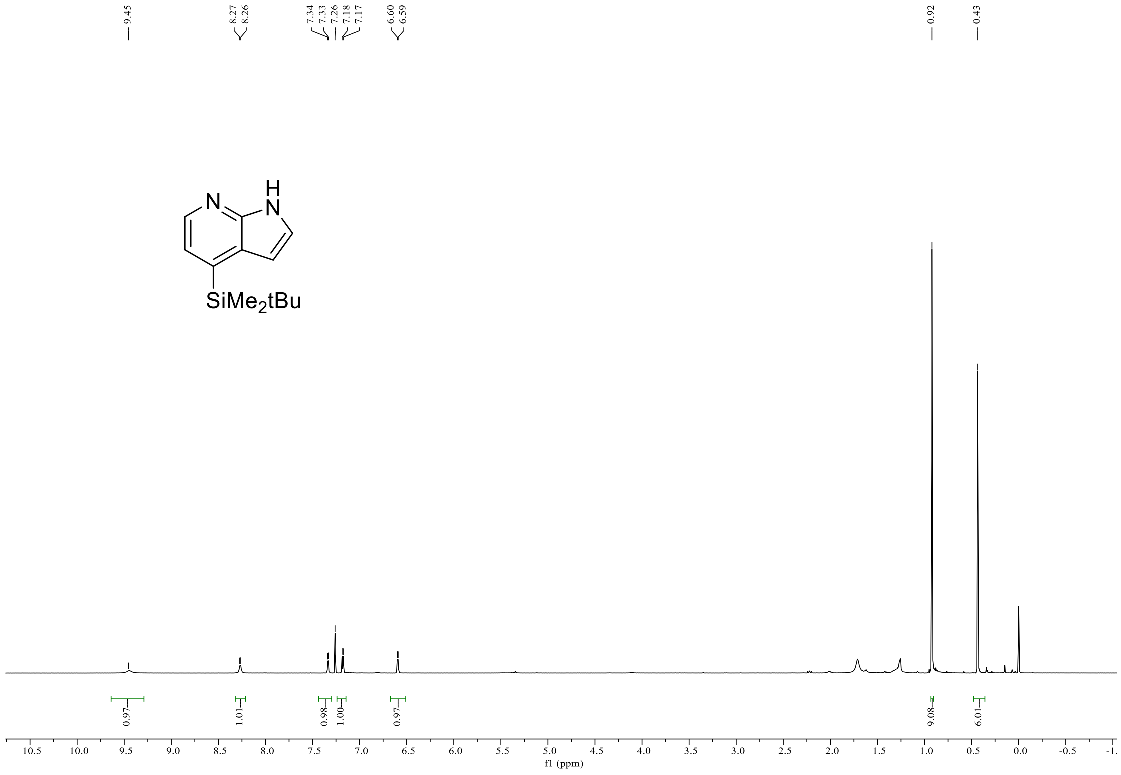

^{13}C NMR (101 MHz, $CDCl_3$) of 45

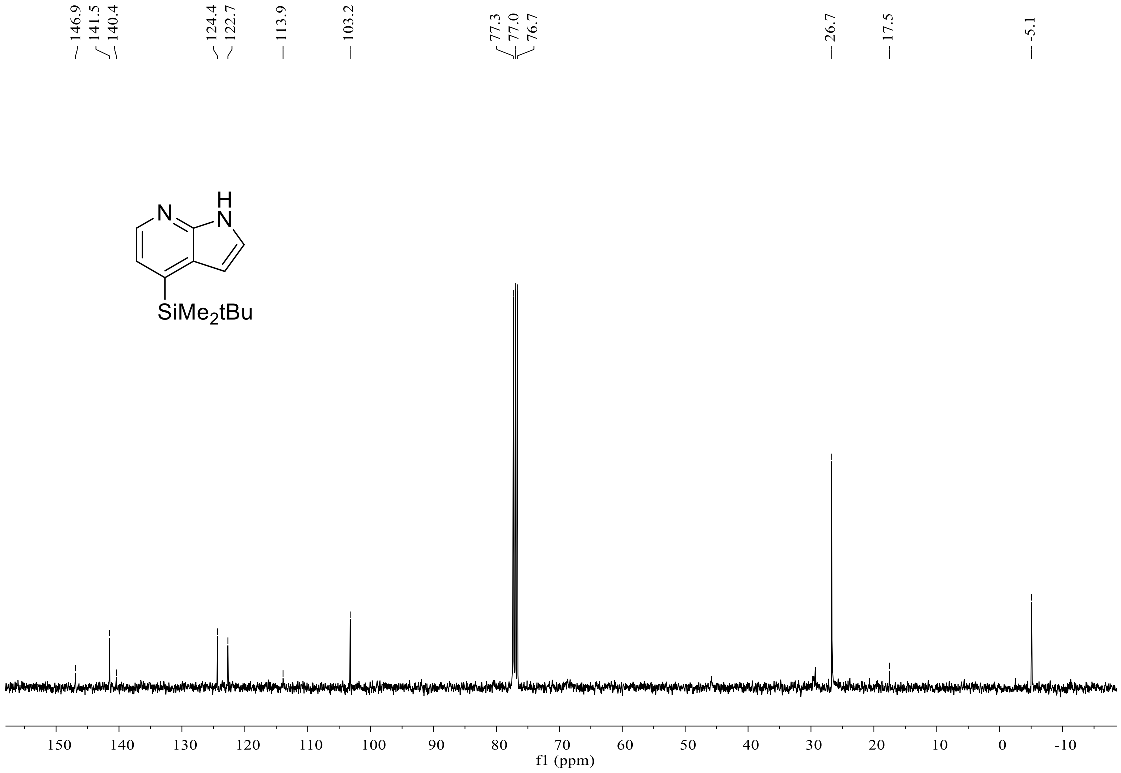

^{1}H NMR (400 MHz, $CDCl_3$) of 46

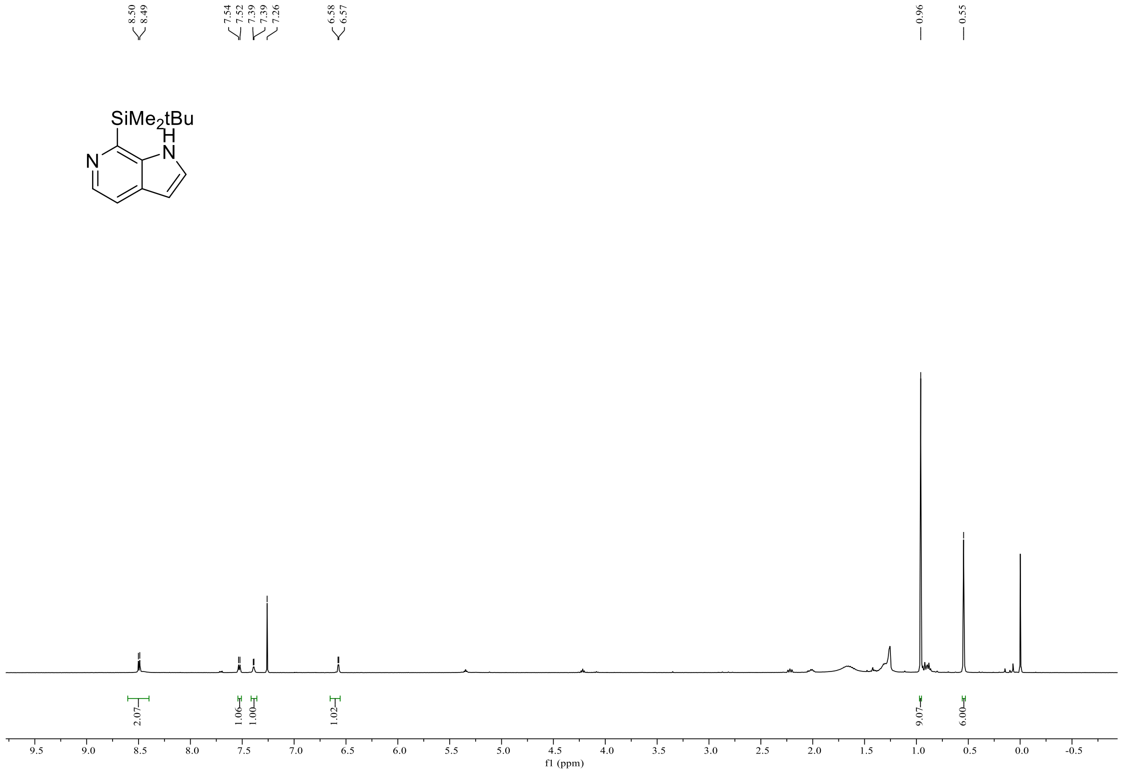

^{13}C NMR (101 MHz, $CDCl_3$) of 46

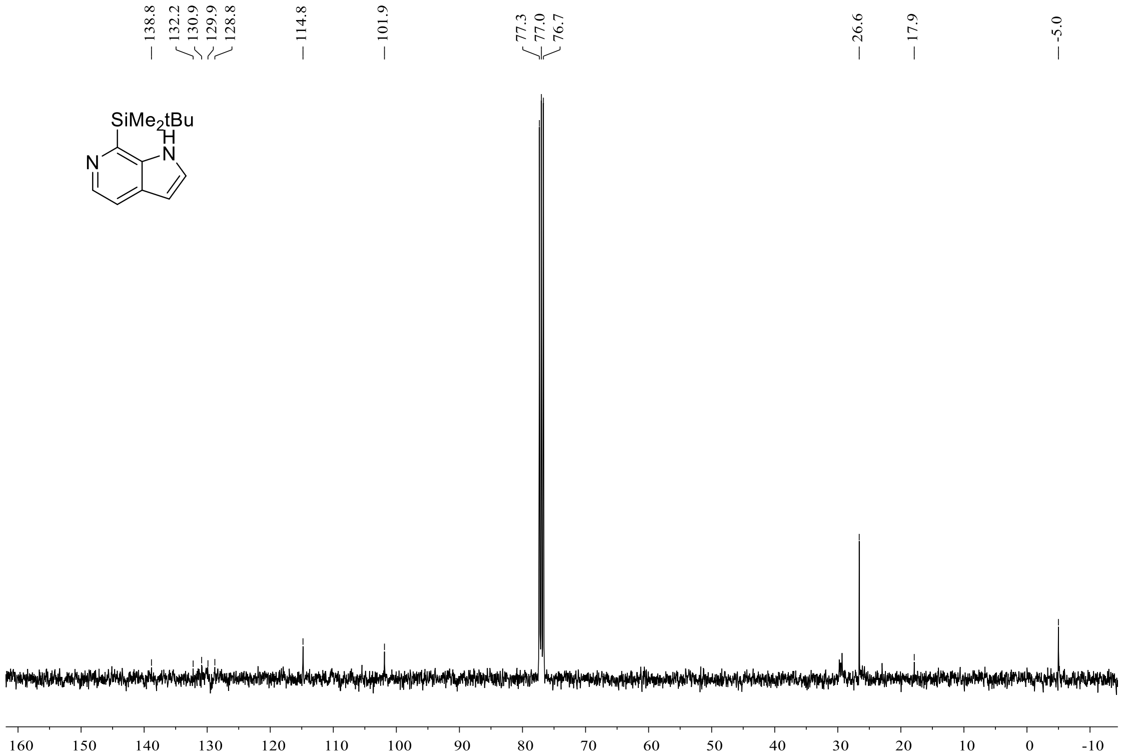

^{1}H NMR (400 MHz, CDCl$_3$) of 47

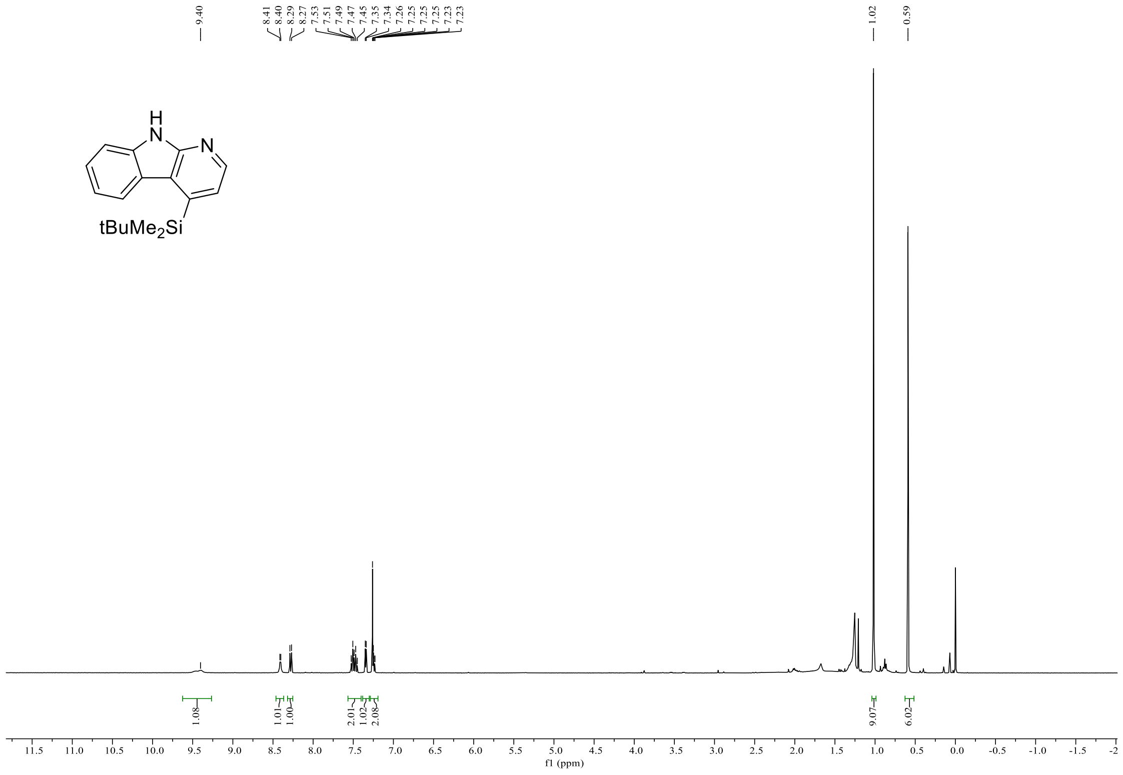

^{13}C NMR (101 MHz, CDCl$_3$) of 47

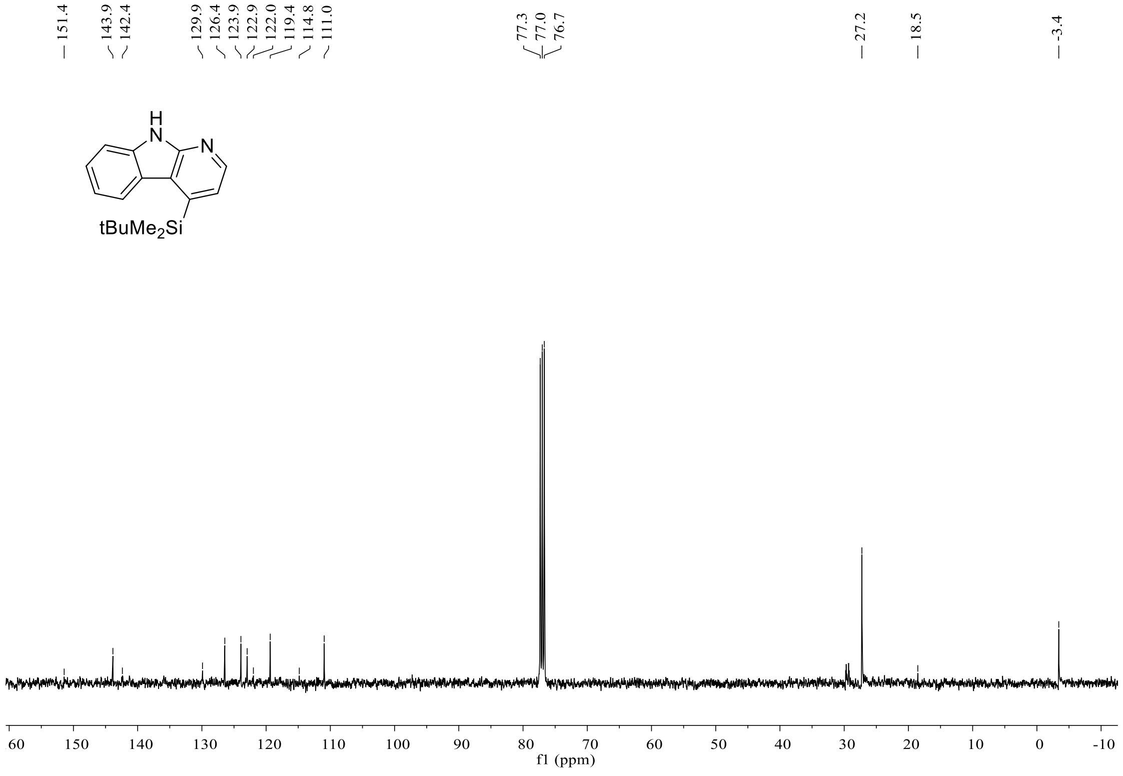

^{1}H NMR (400 MHz, $CDCl_3$) of 48

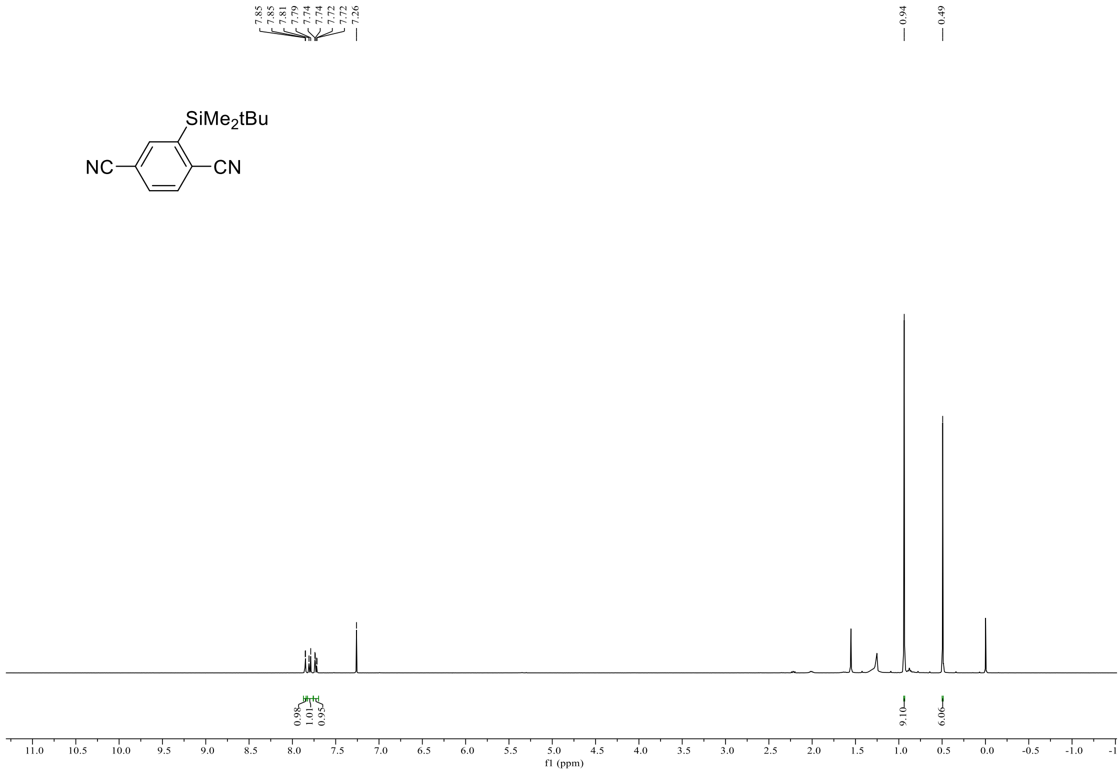

^{13}C NMR (101 MHz, $CDCl_3$) of 48

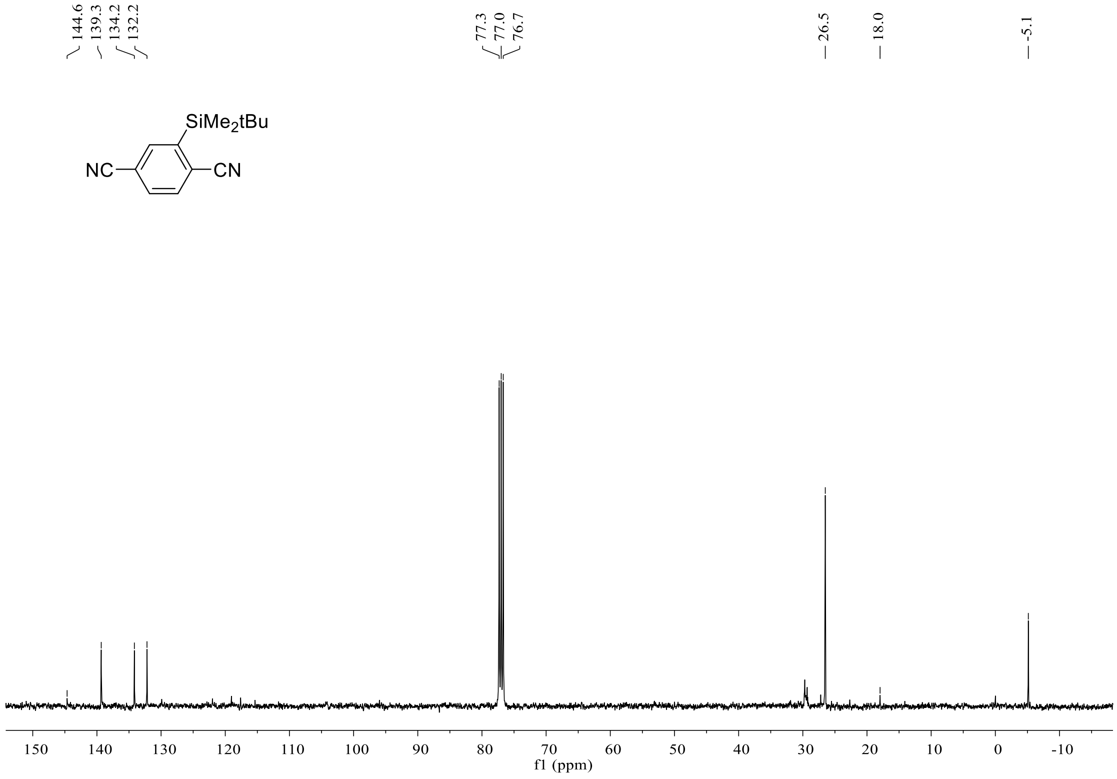

^{1}H NMR (400 MHz, $CDCl_3$) of 49

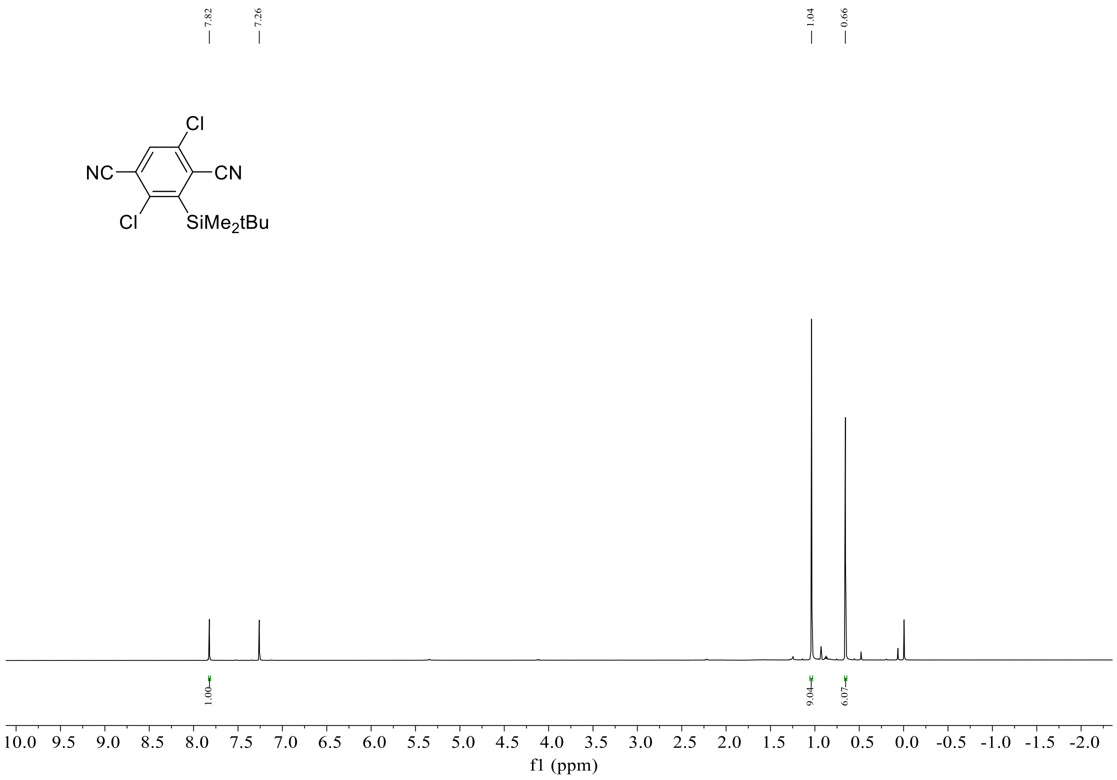

^{13}C NMR (101 MHz, $CDCl_3$) of 49

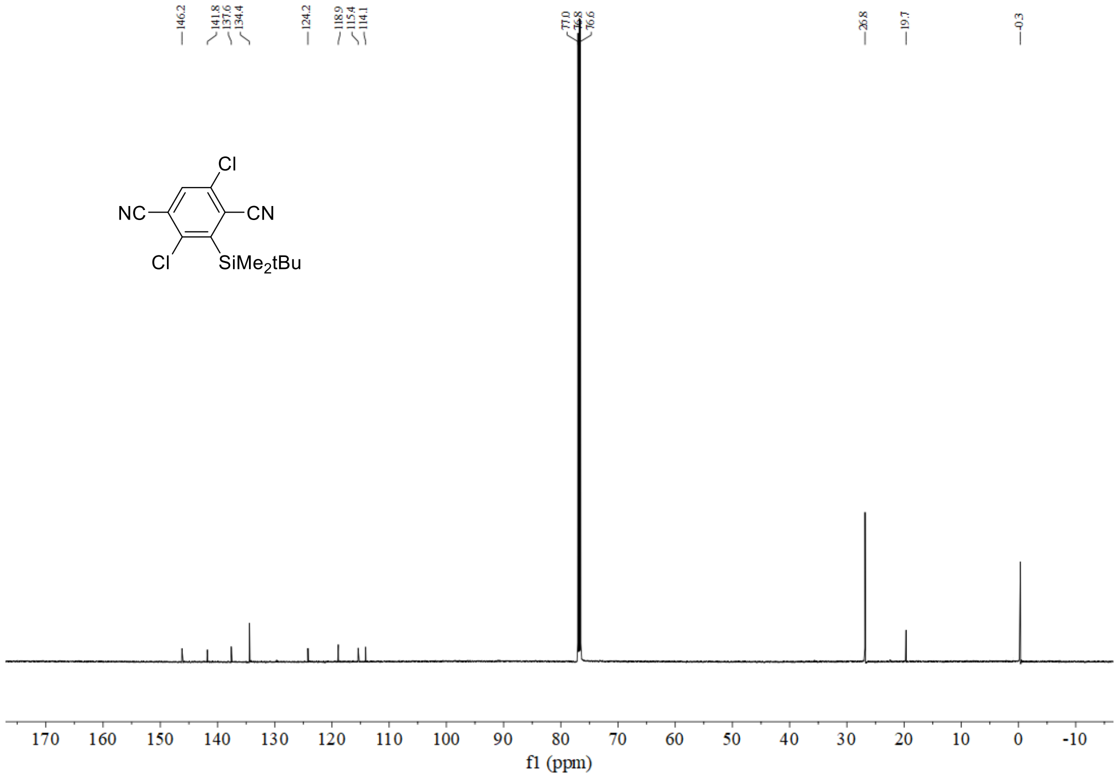

^{1}H NMR (400 MHz, CDCl$_3$) of 50

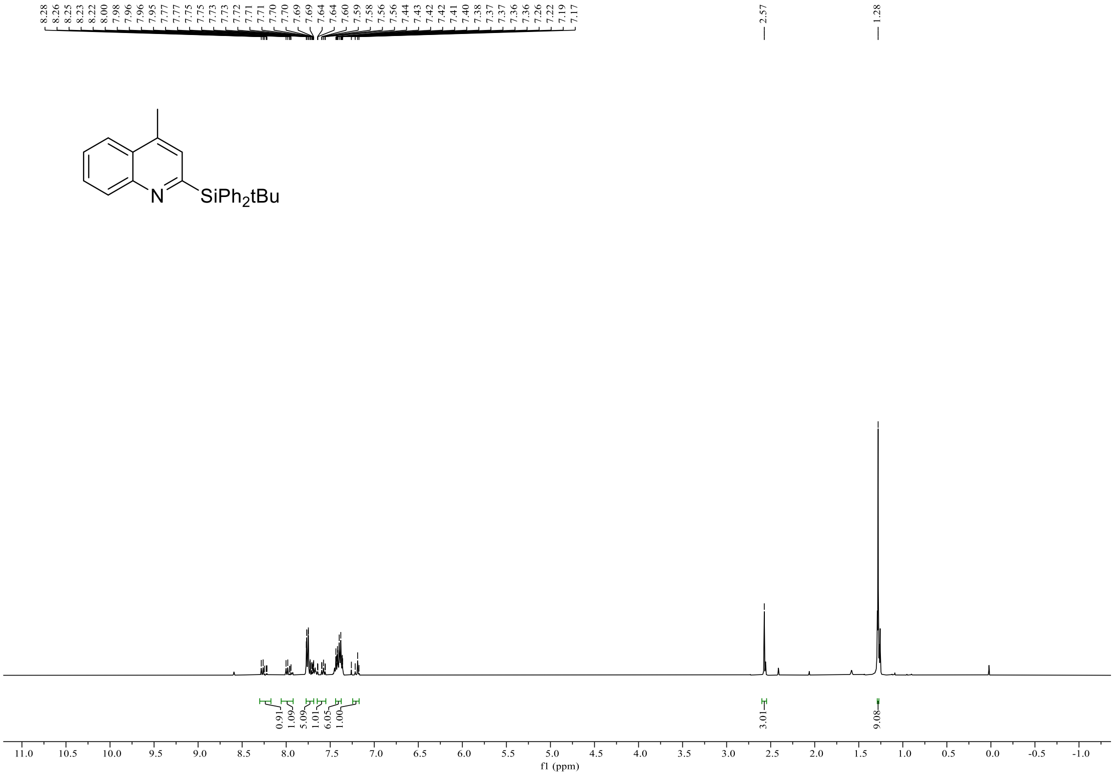

^{13}C NMR (101 MHz, CDCl$_3$) of 50

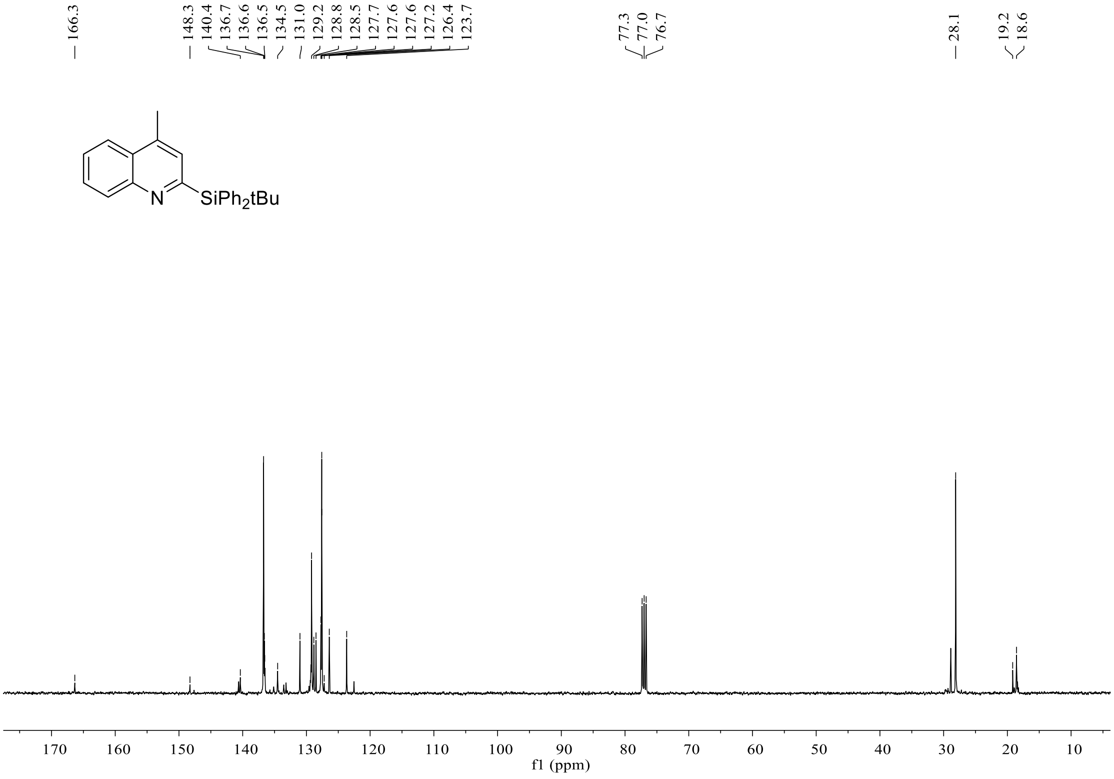

^{1}H NMR (400 MHz, $CDCl_3$) of 51

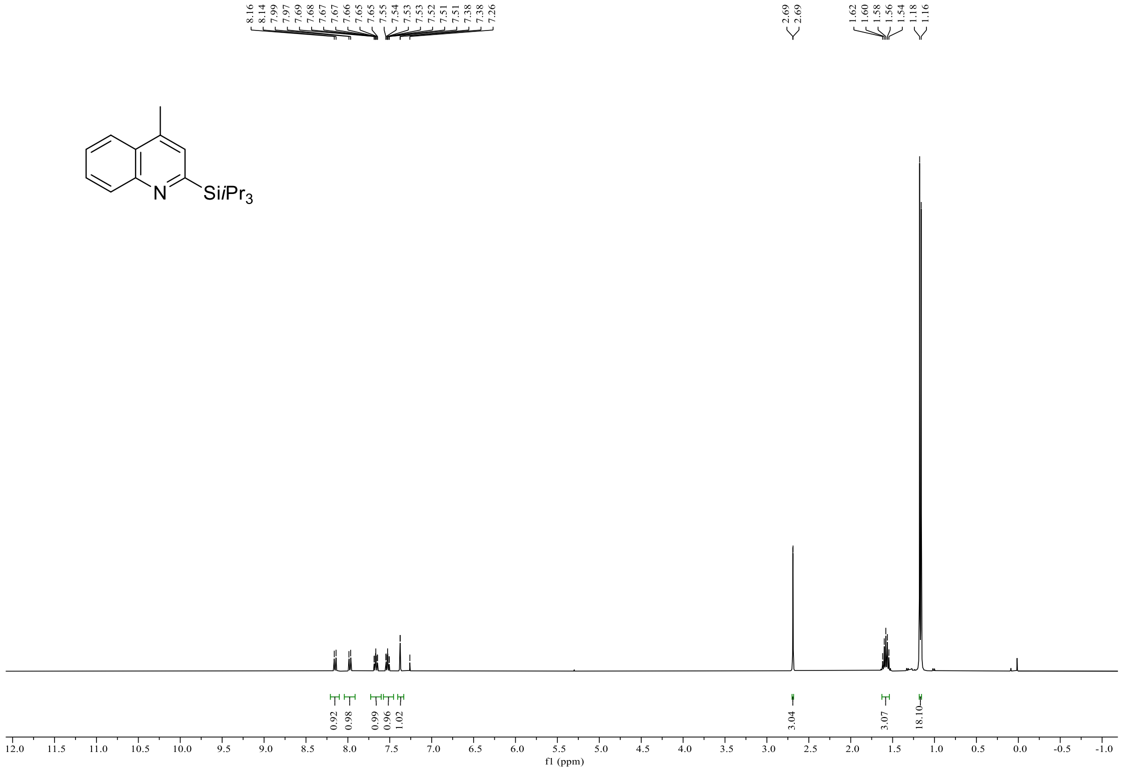

^{13}C NMR (101 MHz, $CDCl_3$) of 51

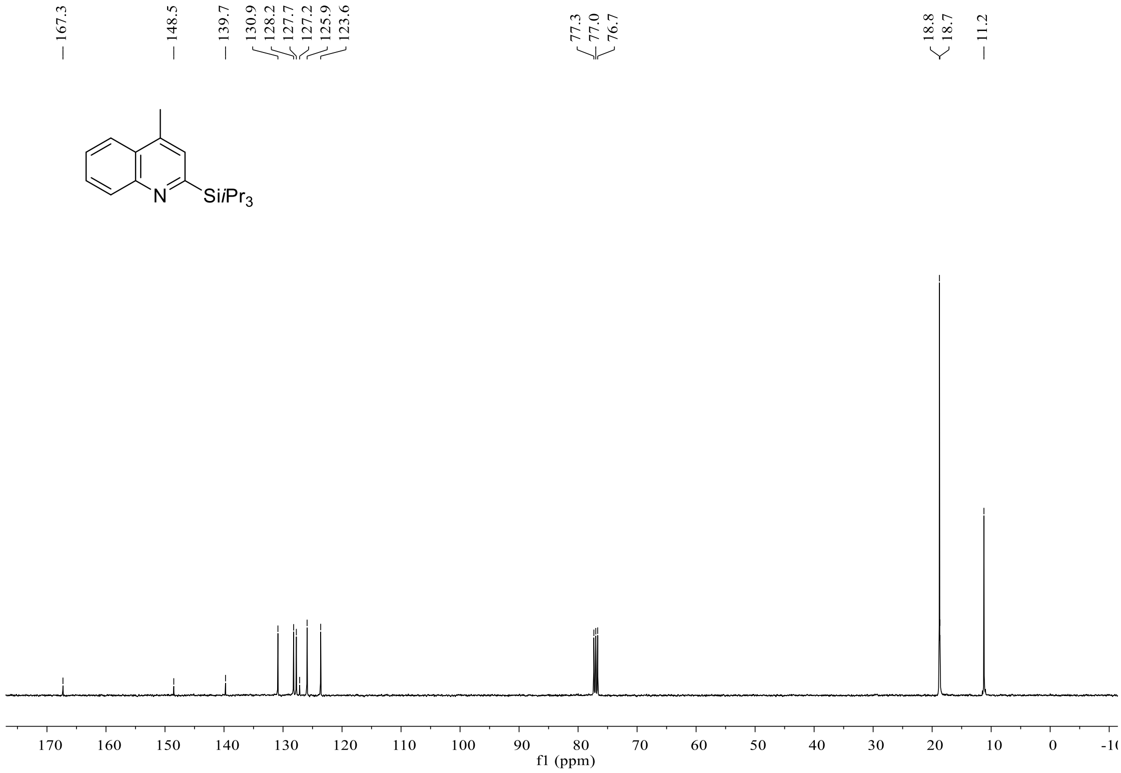

^{1}H NMR (400 MHz, $CDCl_3$) of 53

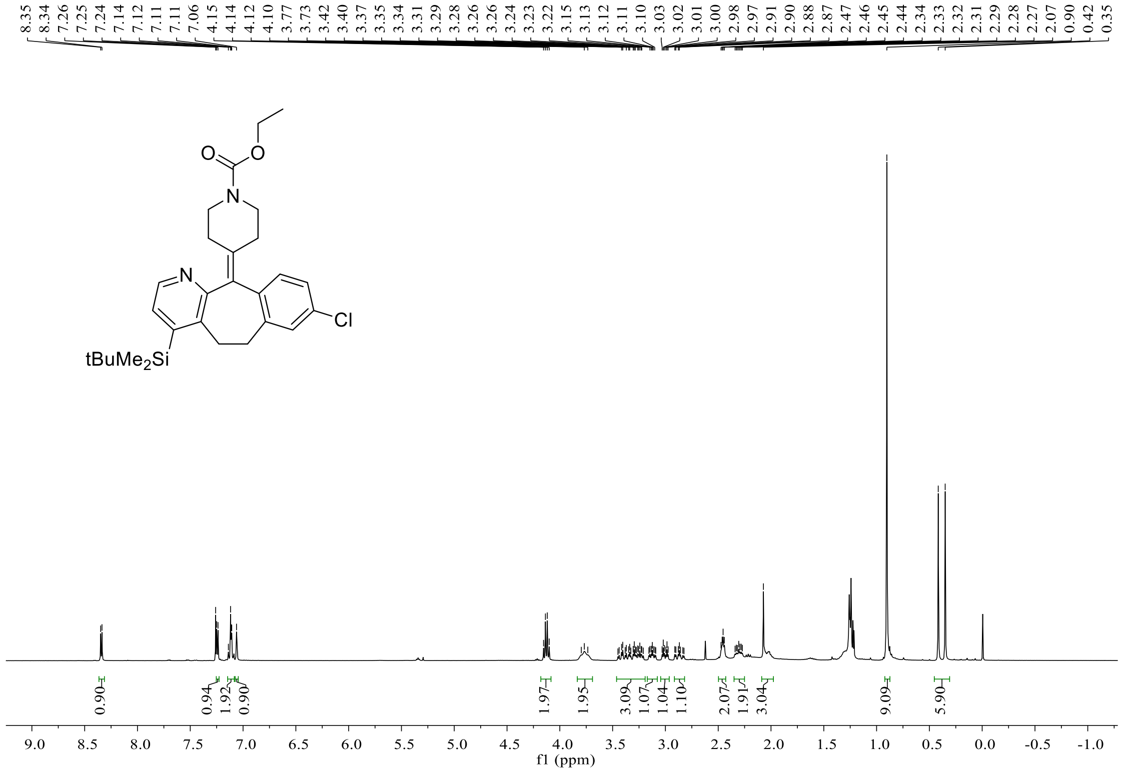

^{13}C NMR (101 MHz, $CDCl_3$) of 53

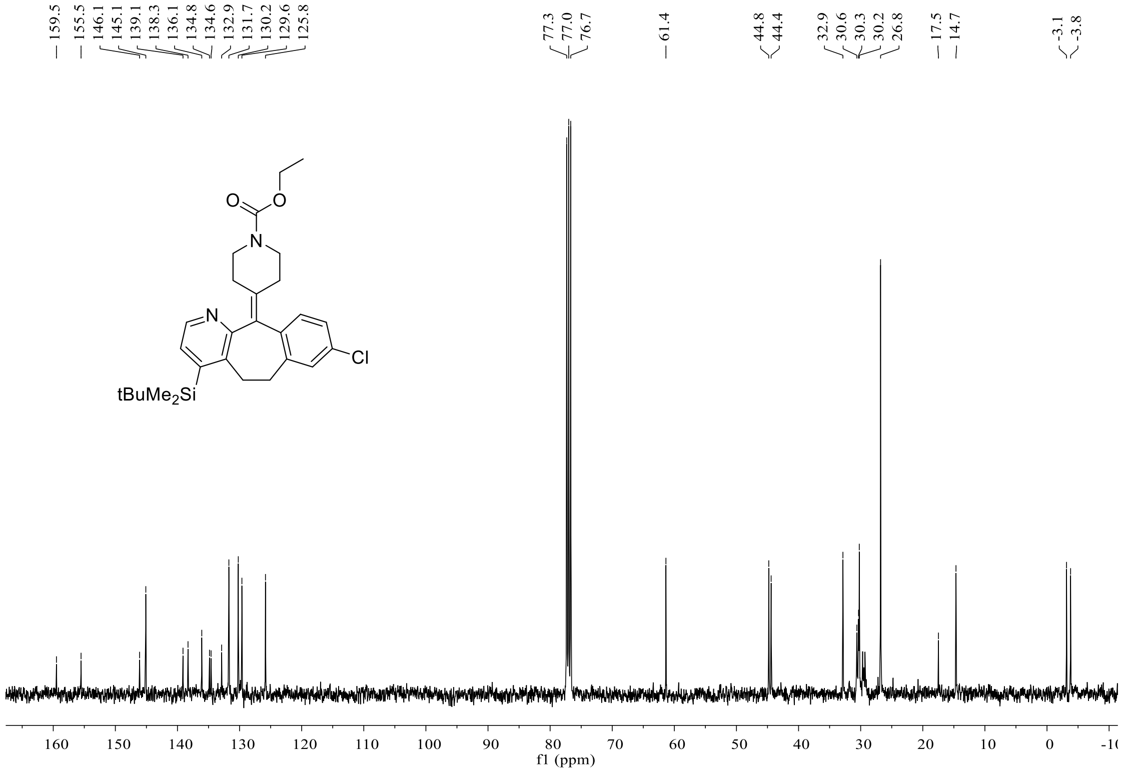

^{1}H NMR (400 MHz, CDCl$_3$) of 54

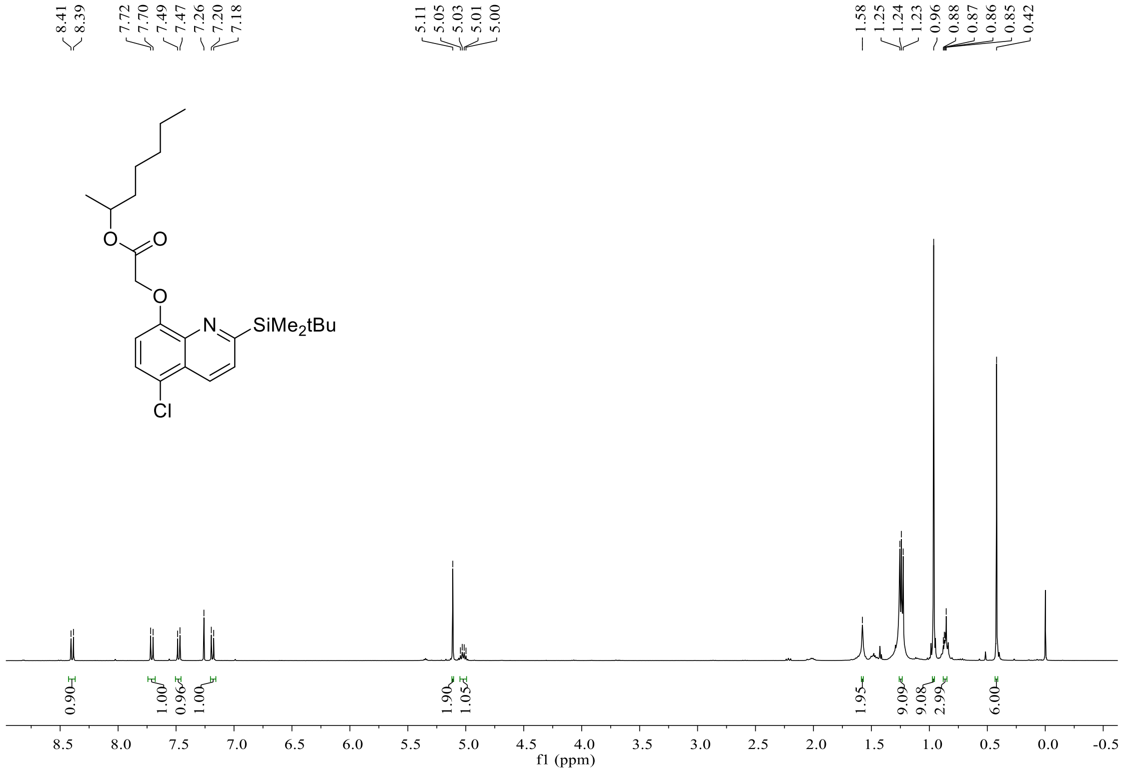

^{13}C NMR (101 MHz, CDCl$_3$) of 54

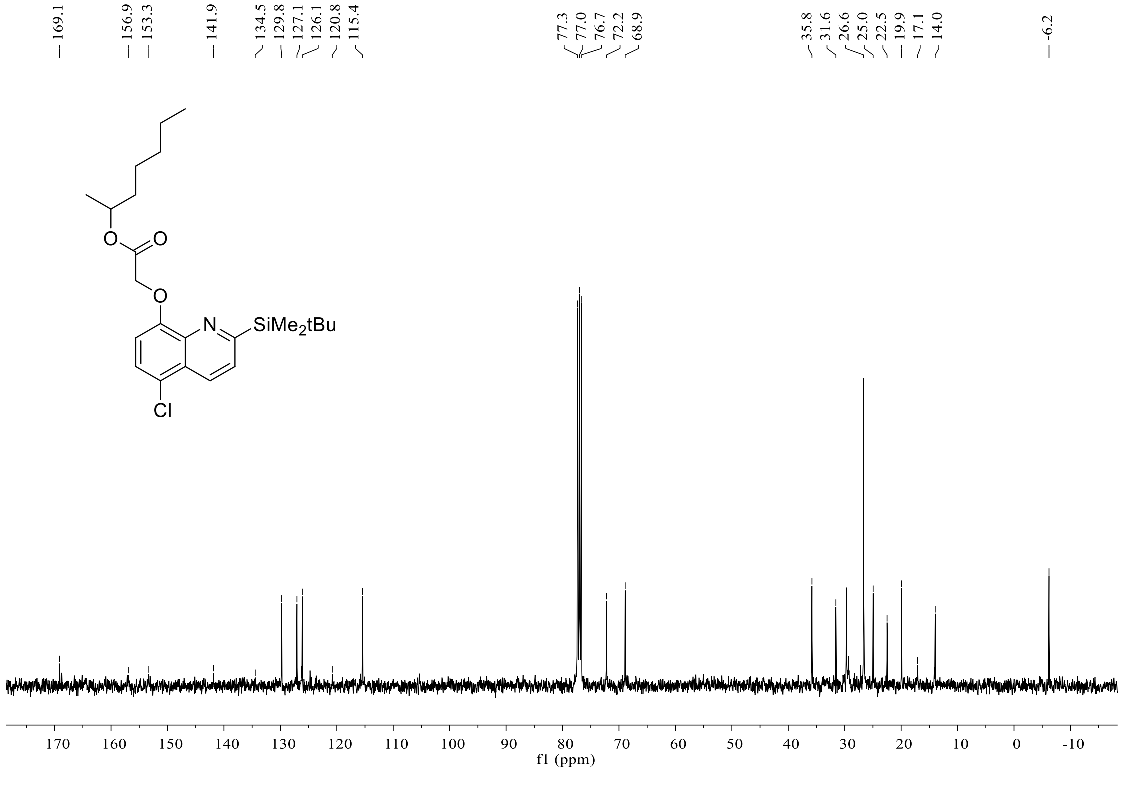

^{1}H NMR (400 MHz, $CDCl_3$) of 55

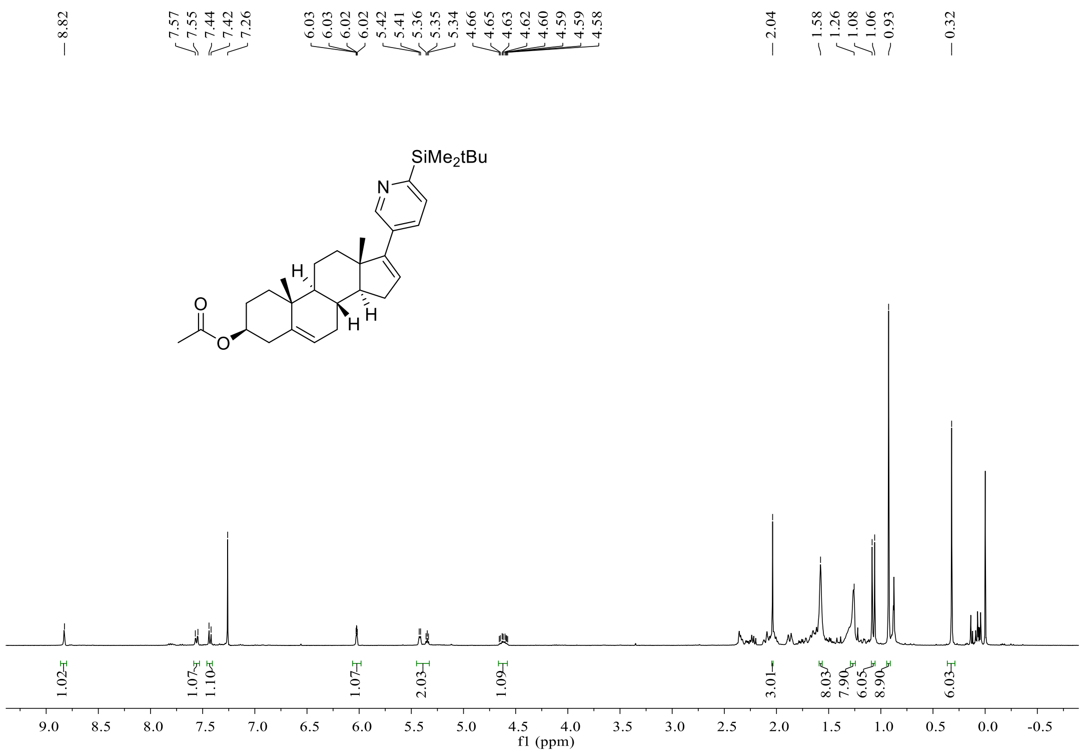

^{13}C NMR (101 MHz, $CDCl_3$) of 55

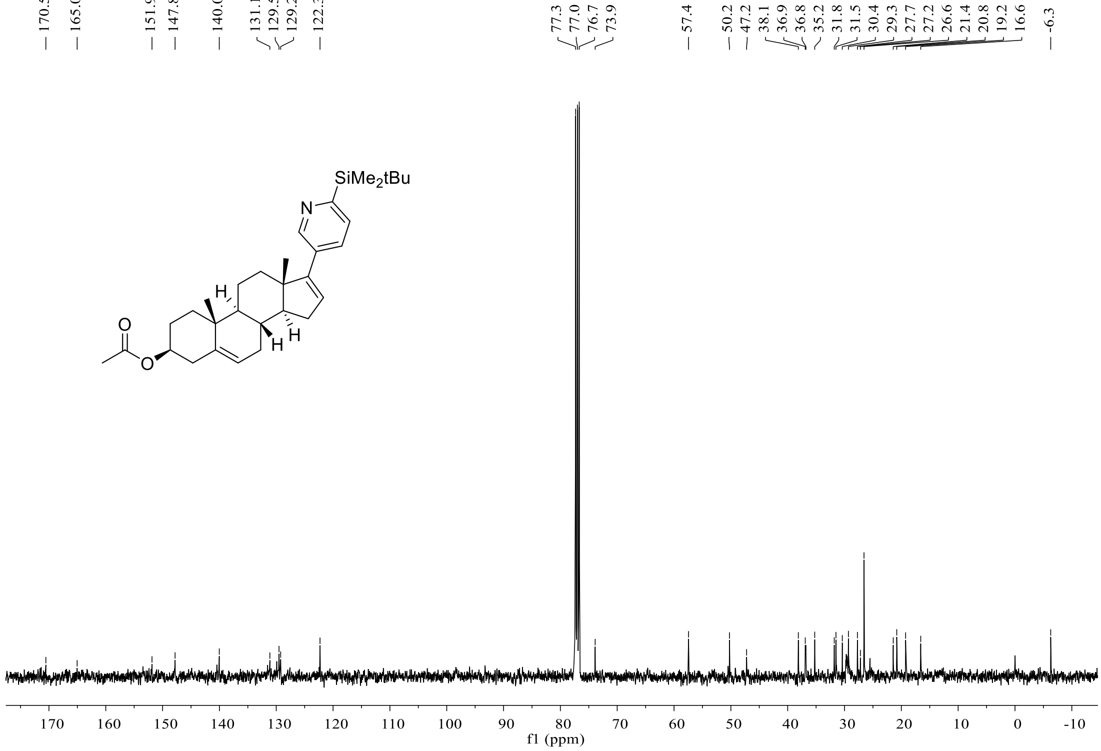

^{1}H NMR (400 MHz, $CDCl_3$) of 56

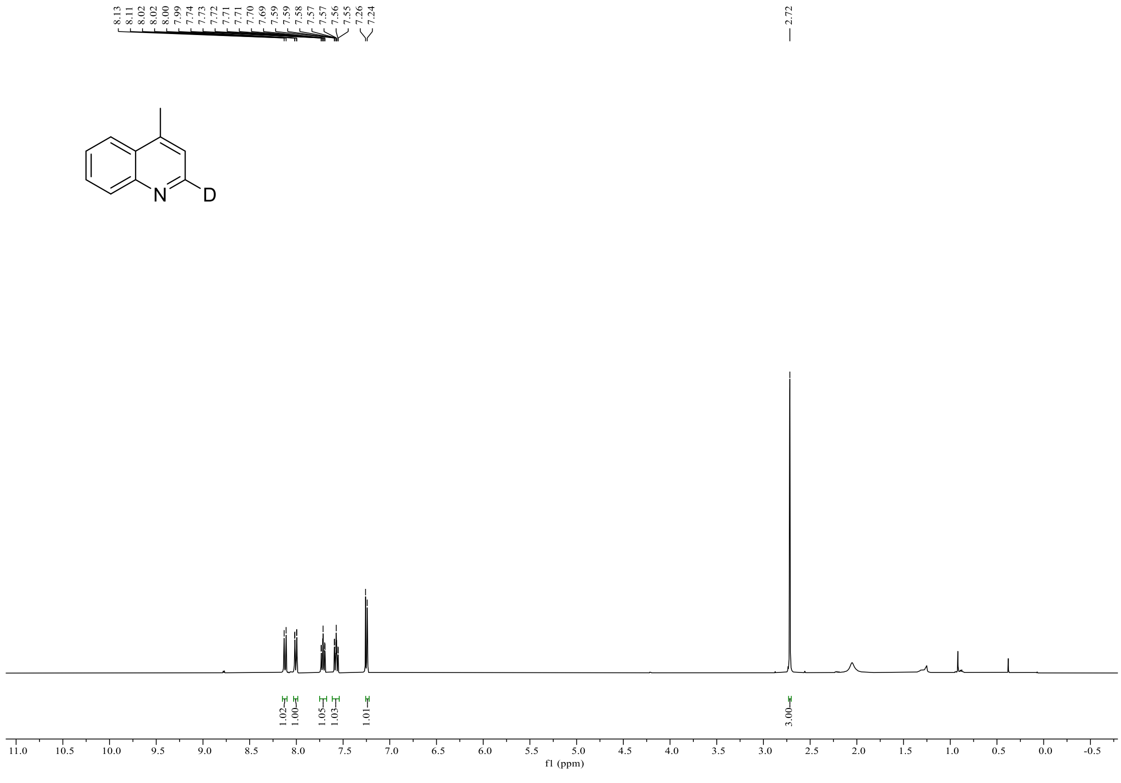

^{13}C NMR (101 MHz, $CDCl_3$) of 56

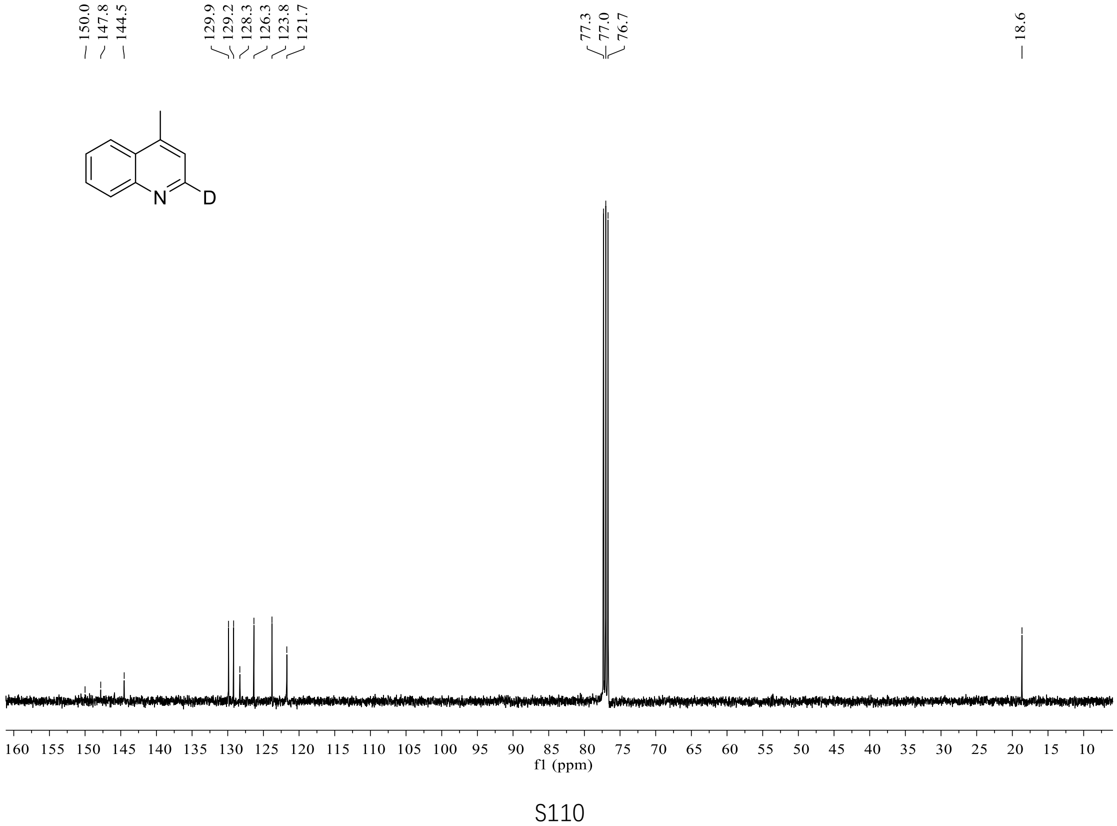

^{1}H NMR (400 MHz, $CDCl_3$) of 57

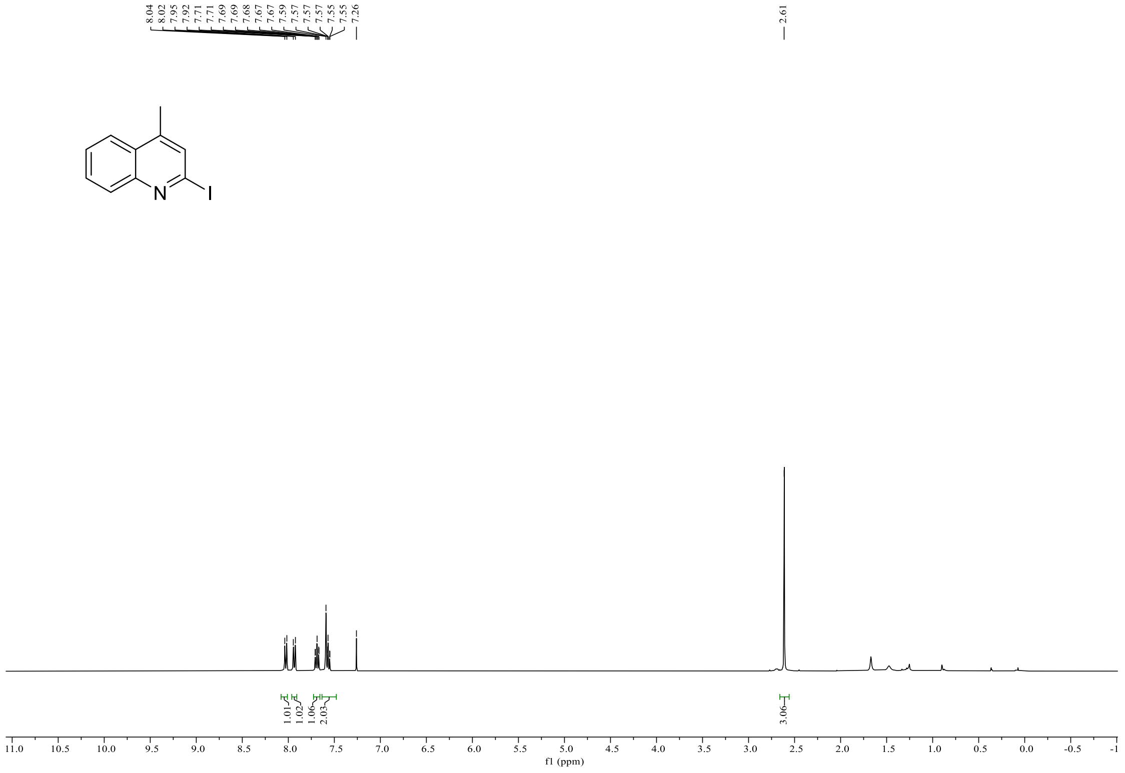

^{13}C NMR (101 MHz, $CDCl_3$) of 57

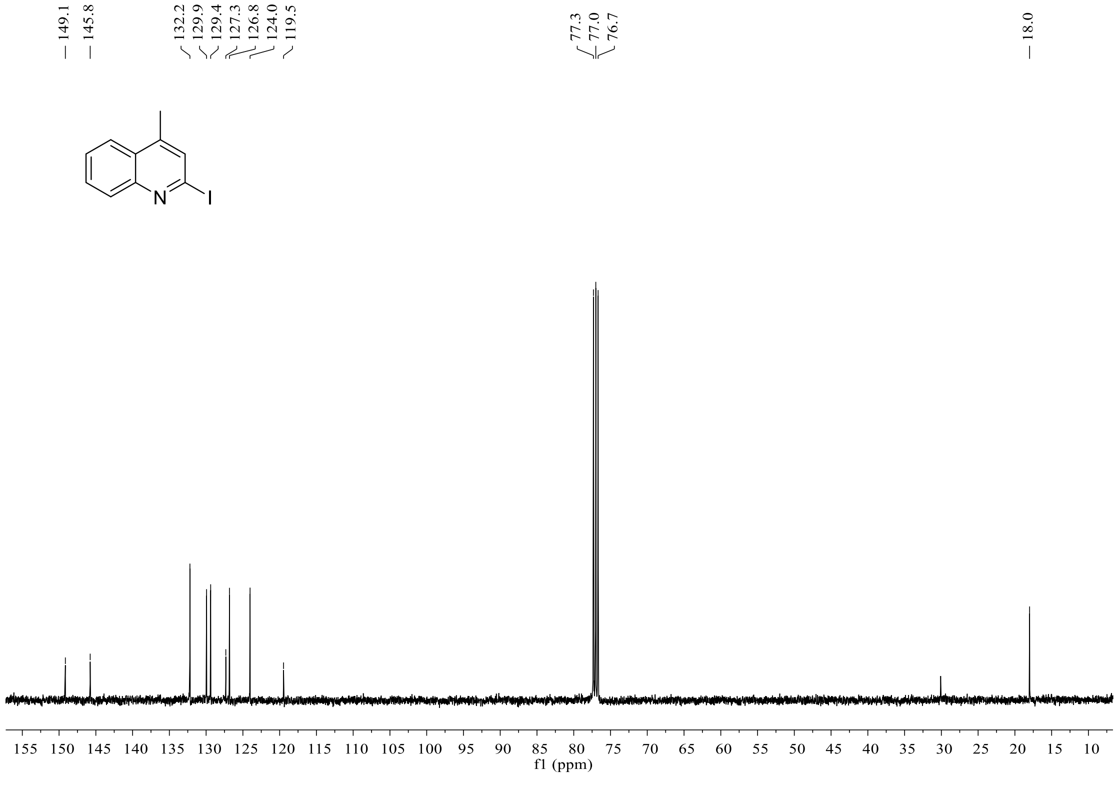

^{1}H NMR (400 MHz, CDCl$_3$) of 58

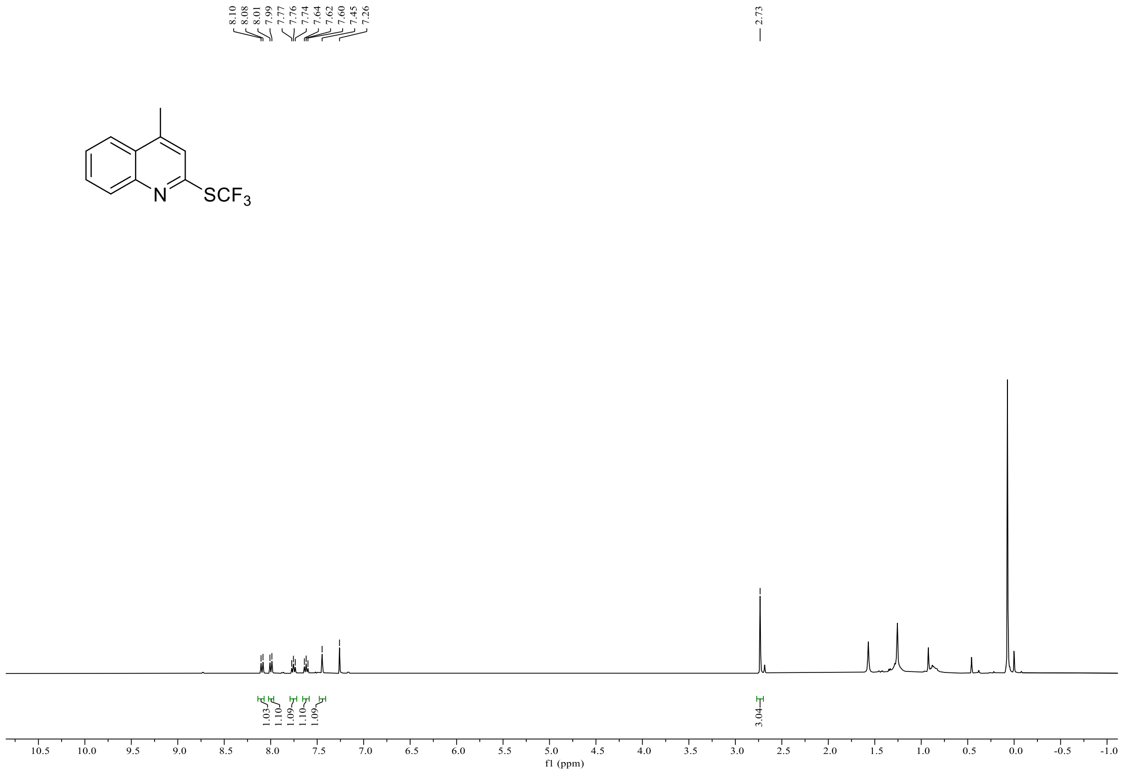

^{13}C NMR (101 MHz, CDCl$_3$) of 58

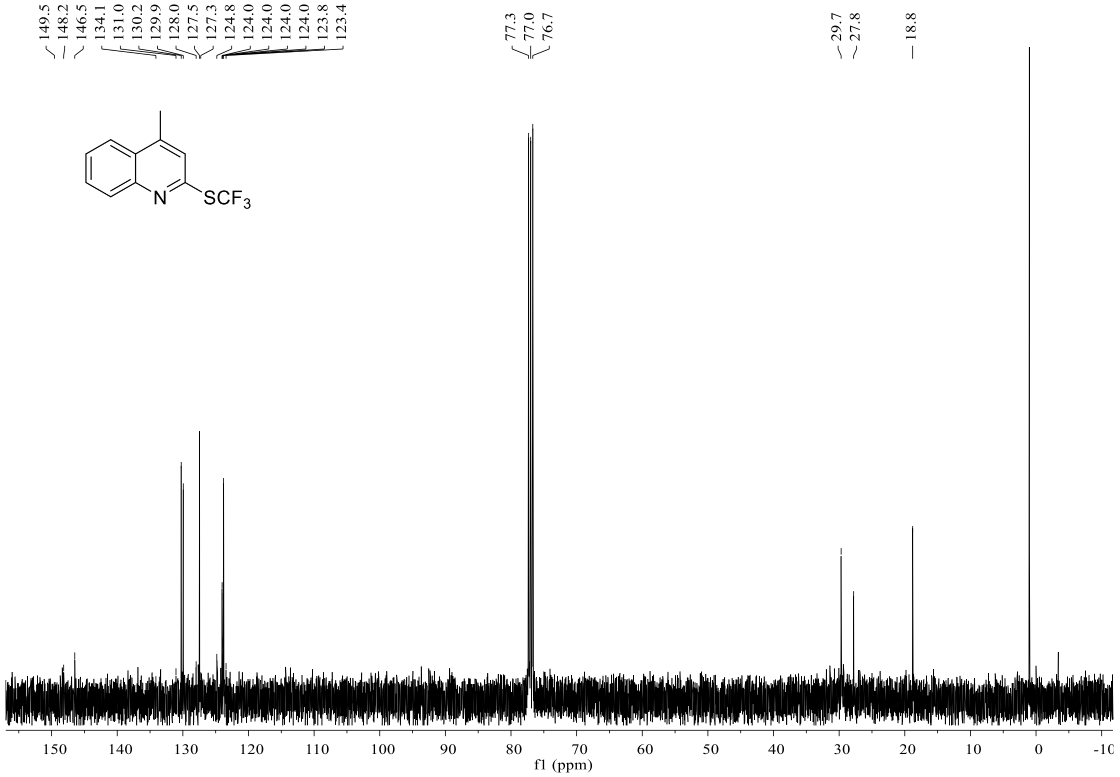

^{19}F NMR (376 MHz, $CDCl_3$) of 58

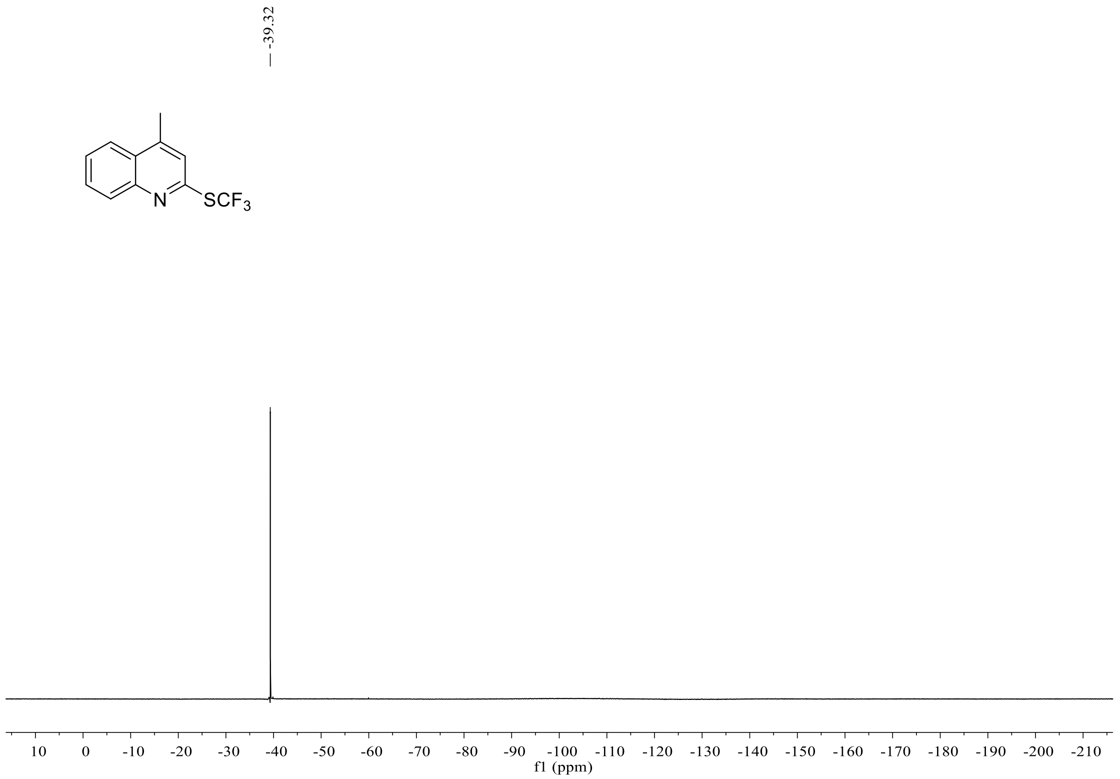

^{1}H NMR (400 MHz, $CDCl_3$) of 59

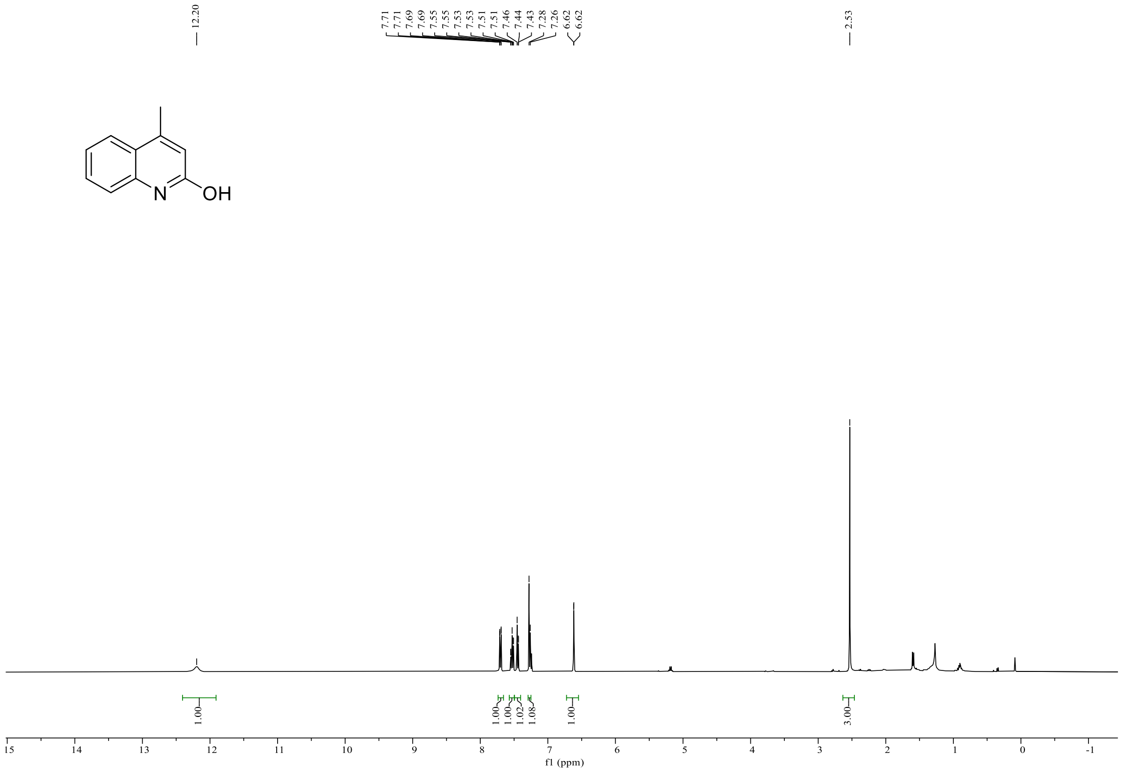

^{13}C NMR (101 MHz, $CDCl_3$) of 59

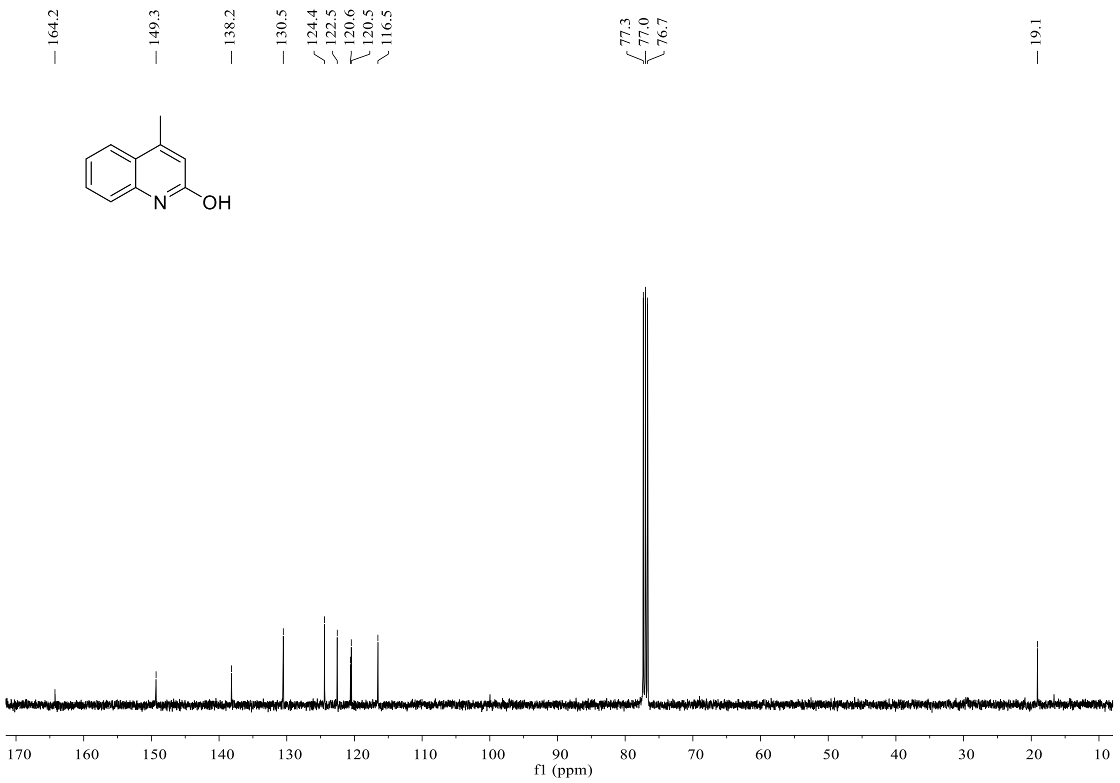

^{1}H NMR (400 MHz, $CDCl_3$) of 60

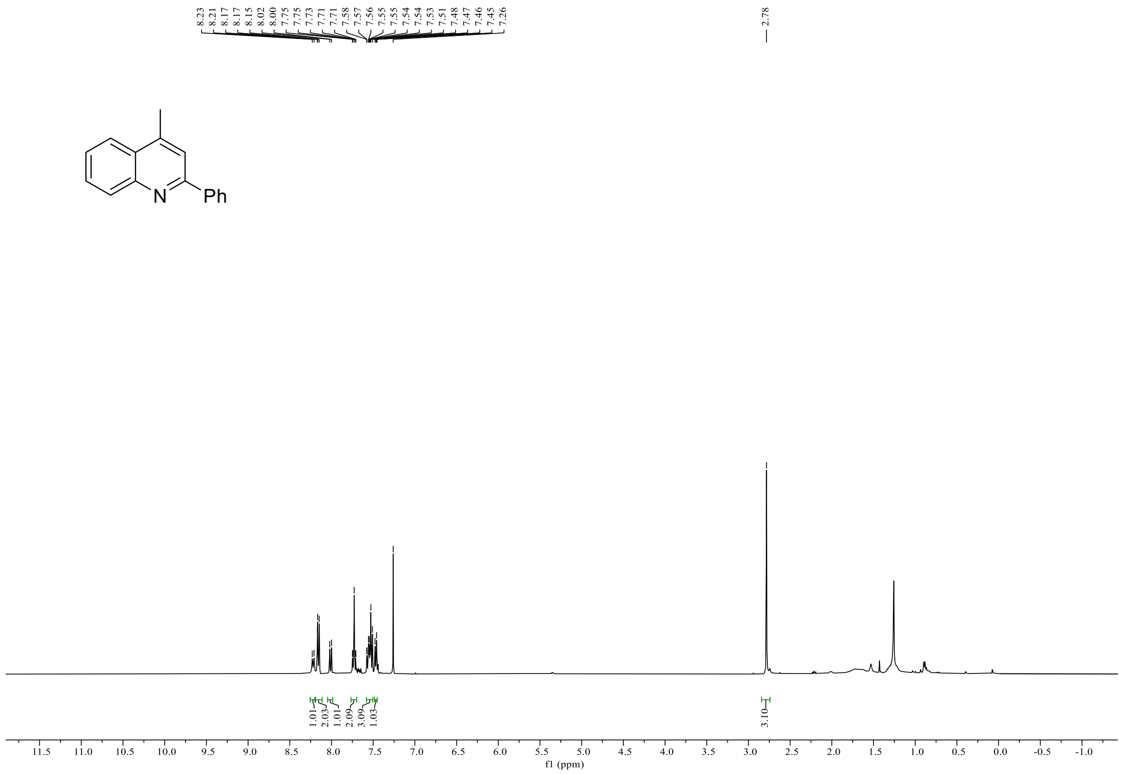

^{13}C NMR (101 MHz, $CDCl_3$) of 60

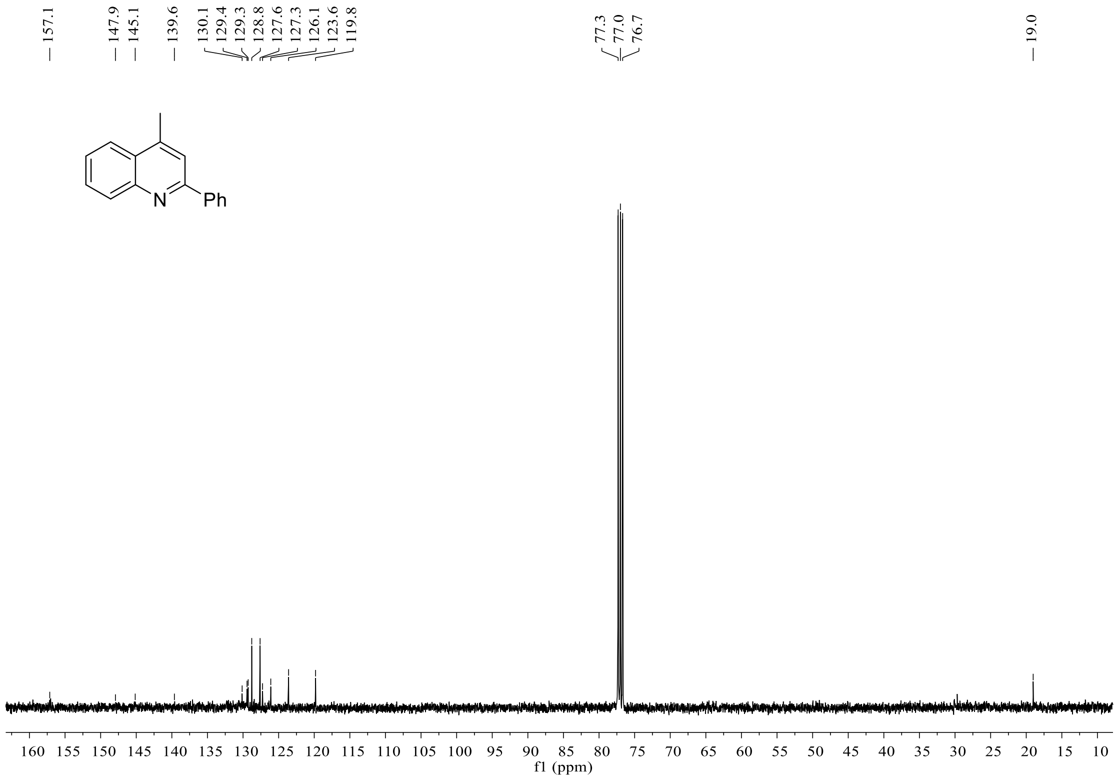

^{1}H NMR (400 MHz, $CDCl_3$) of 61

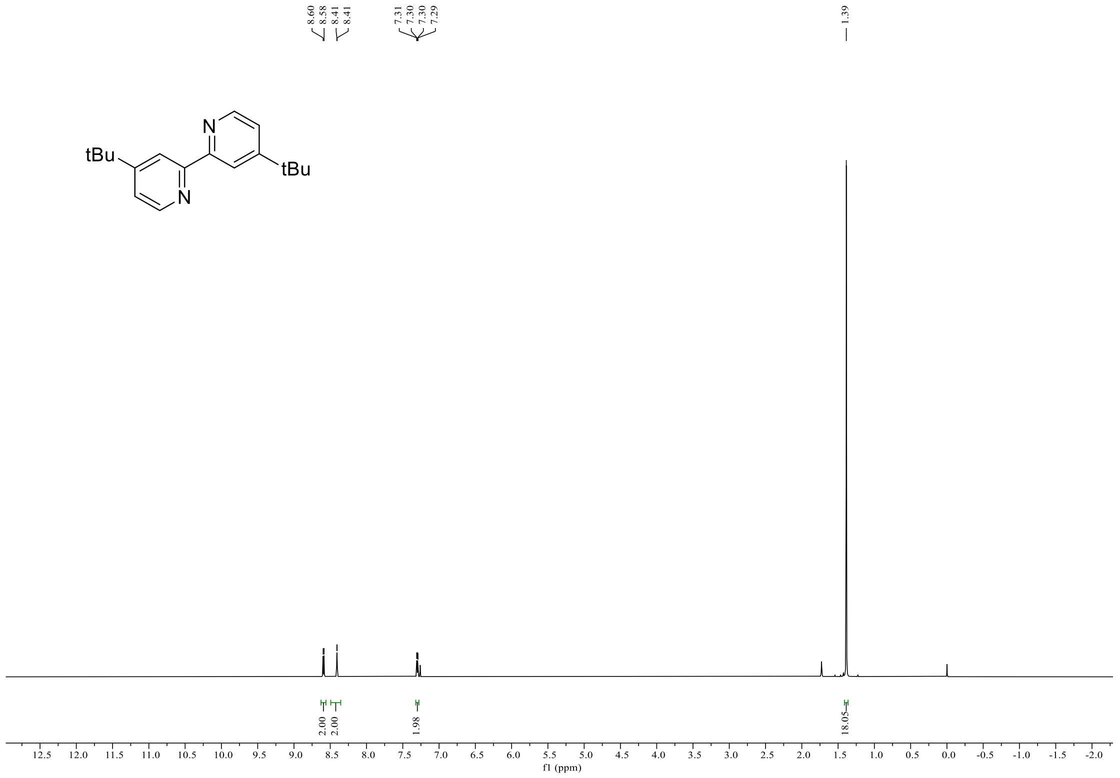

^{13}C NMR (101 MHz, $CDCl_3$) of 61

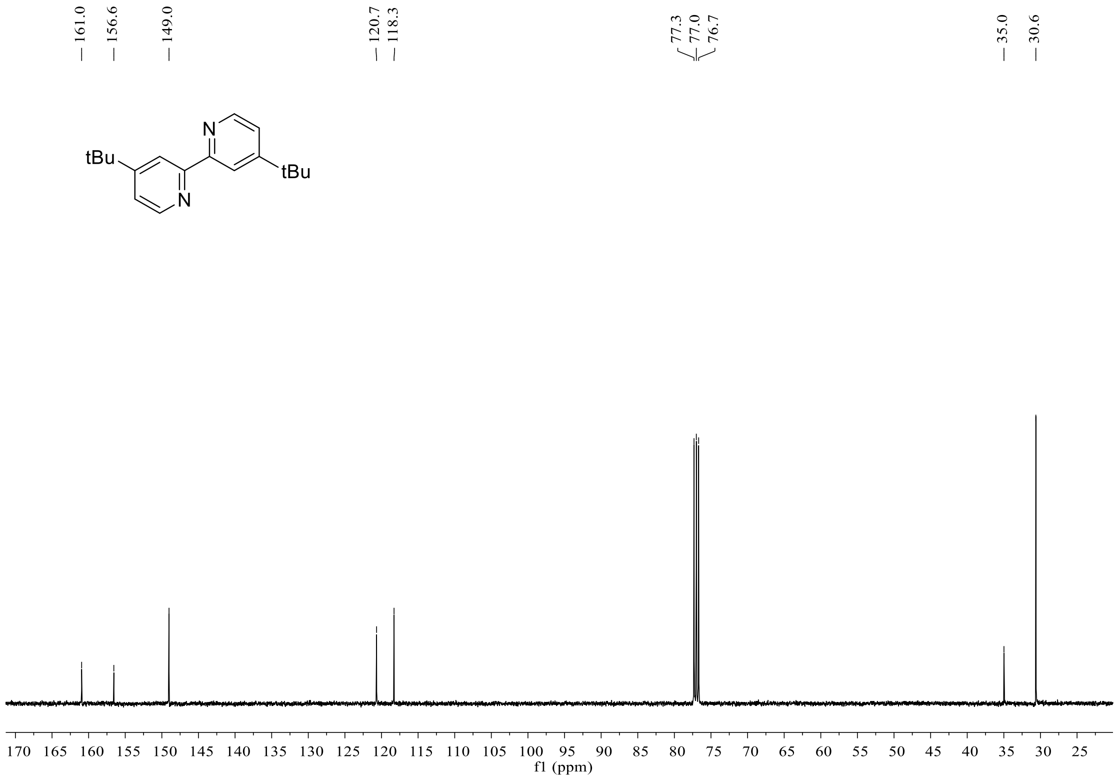

^{1}H NMR (400 MHz, $CDCl_3$) of 62[13a]

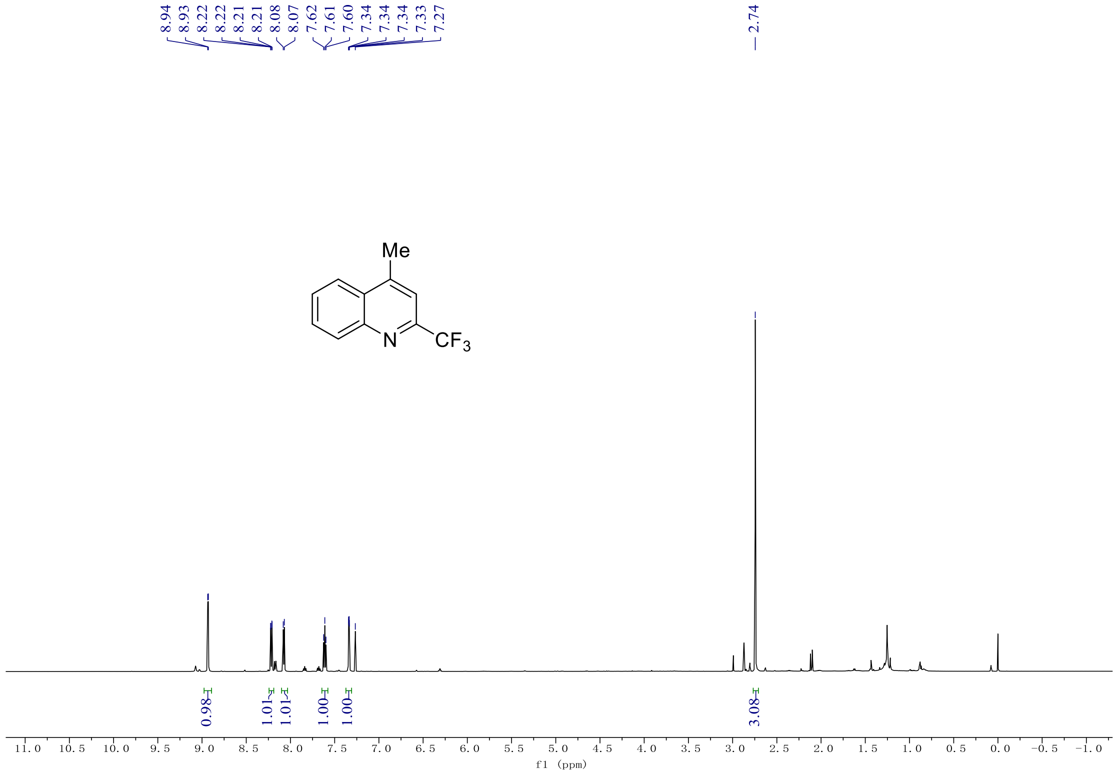

^{1}H NMR (400 MHz, $CDCl_3$) of 63

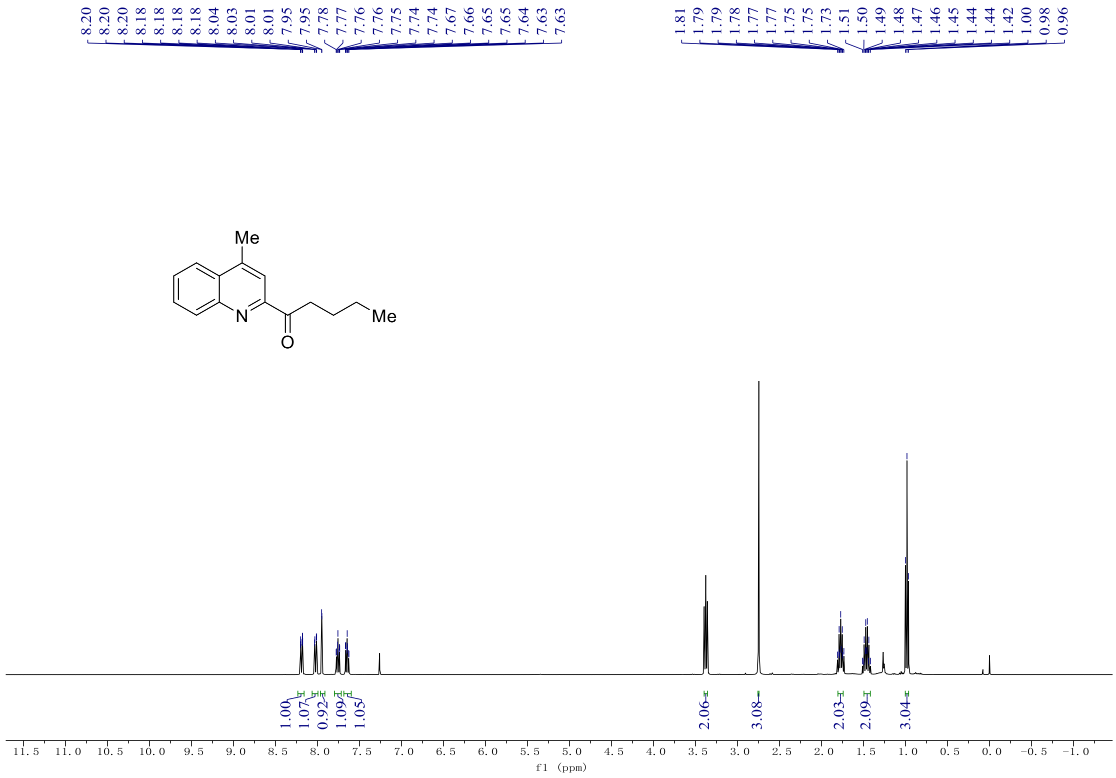

^{13}C NMR (400 MHz, $CDCl_3$) of 63

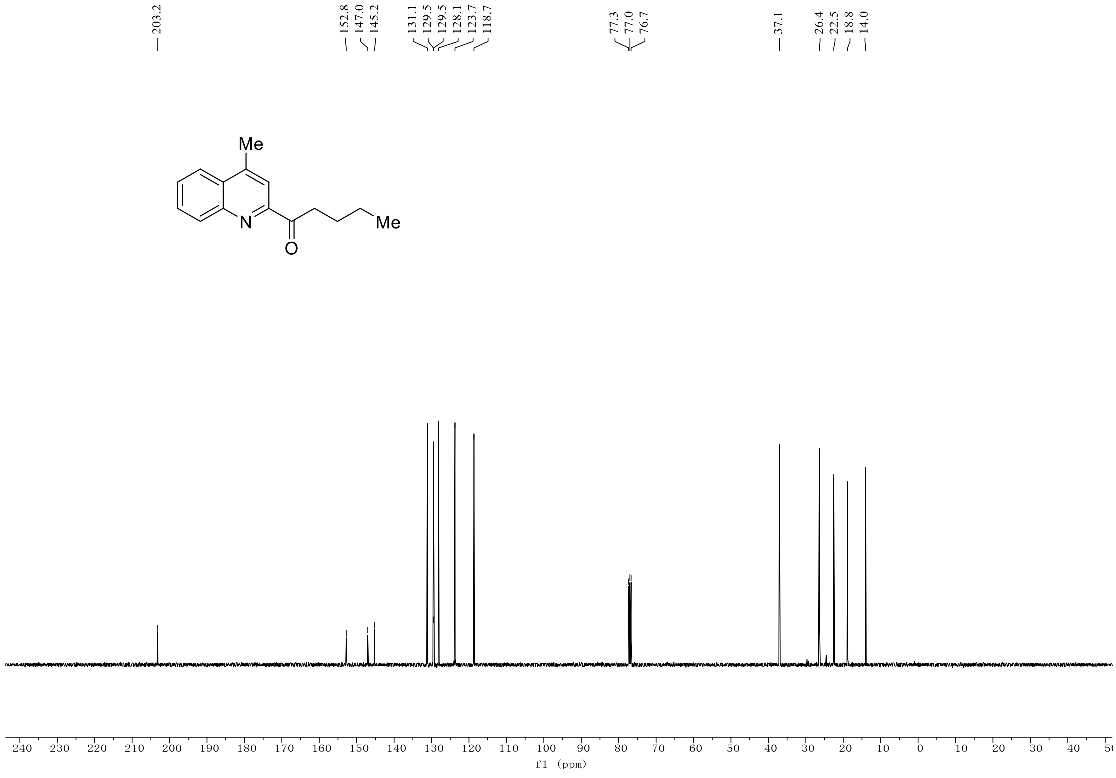

^{1}H NMR (400 MHz, CDCl$_3$) of 64[13c]

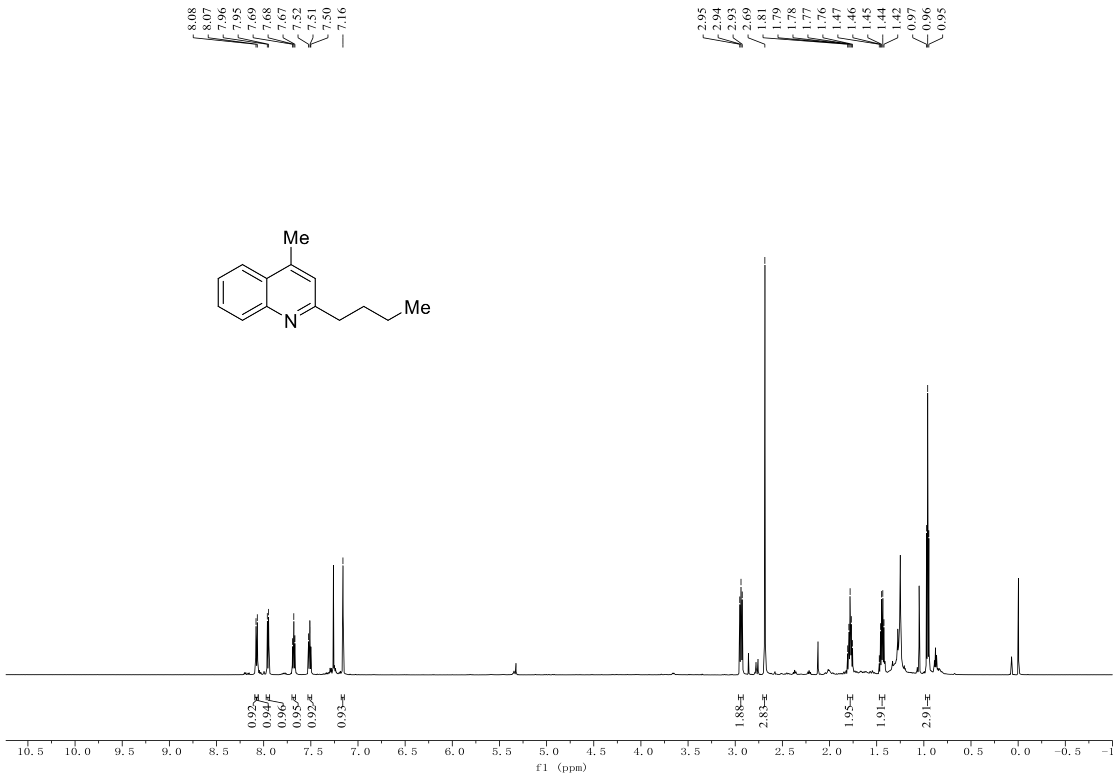

^{1}H NMR (400 MHz, CDCl$_3$) of 65[14]

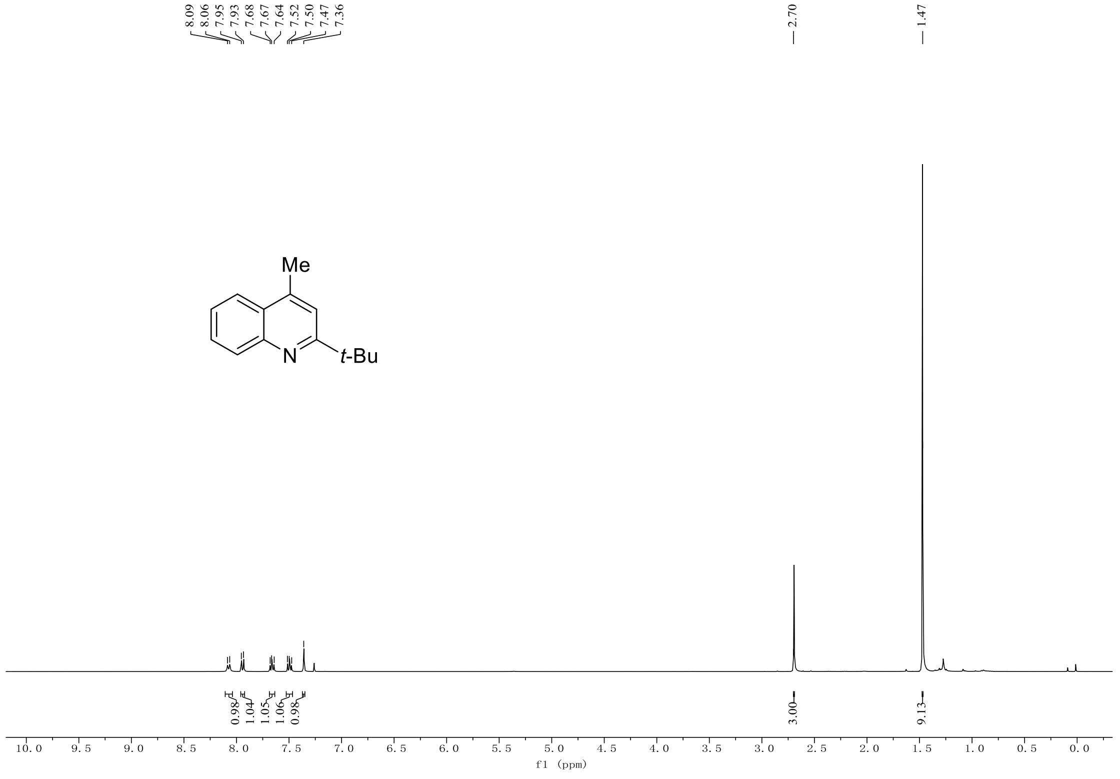

^{1}H NMR (400 MHz, $CDCl_3$) of 68

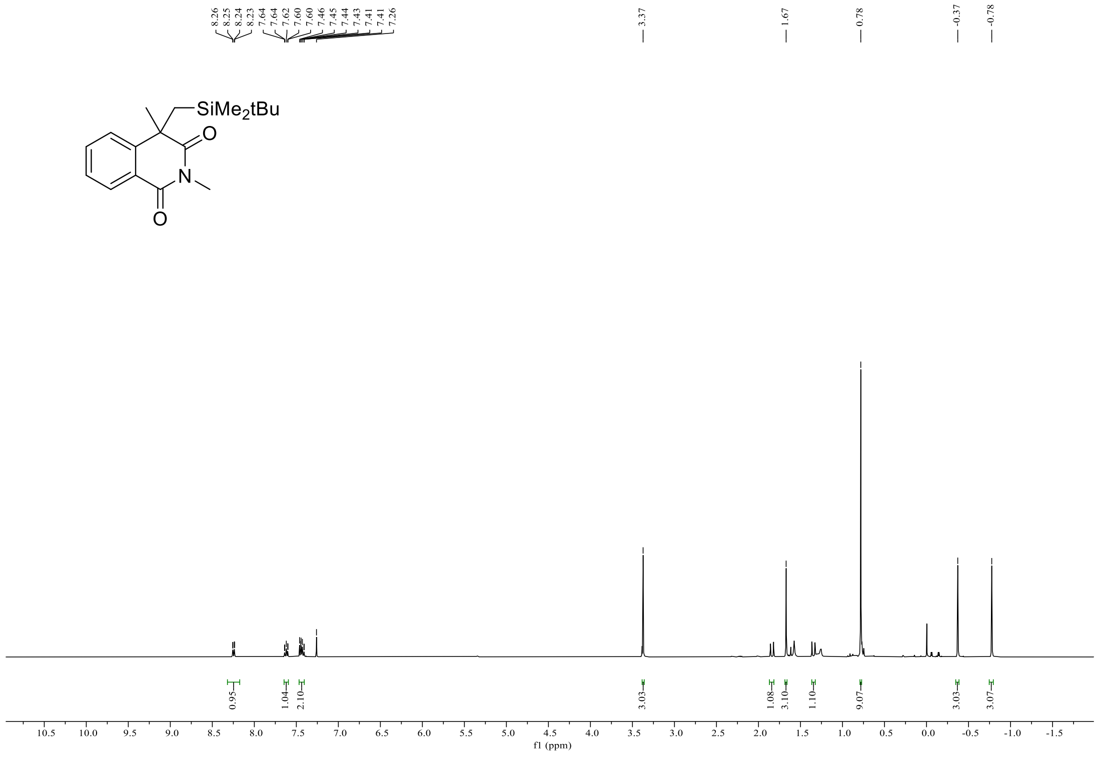

^{13}C NMR (101 MHz, $CDCl_3$) of 68

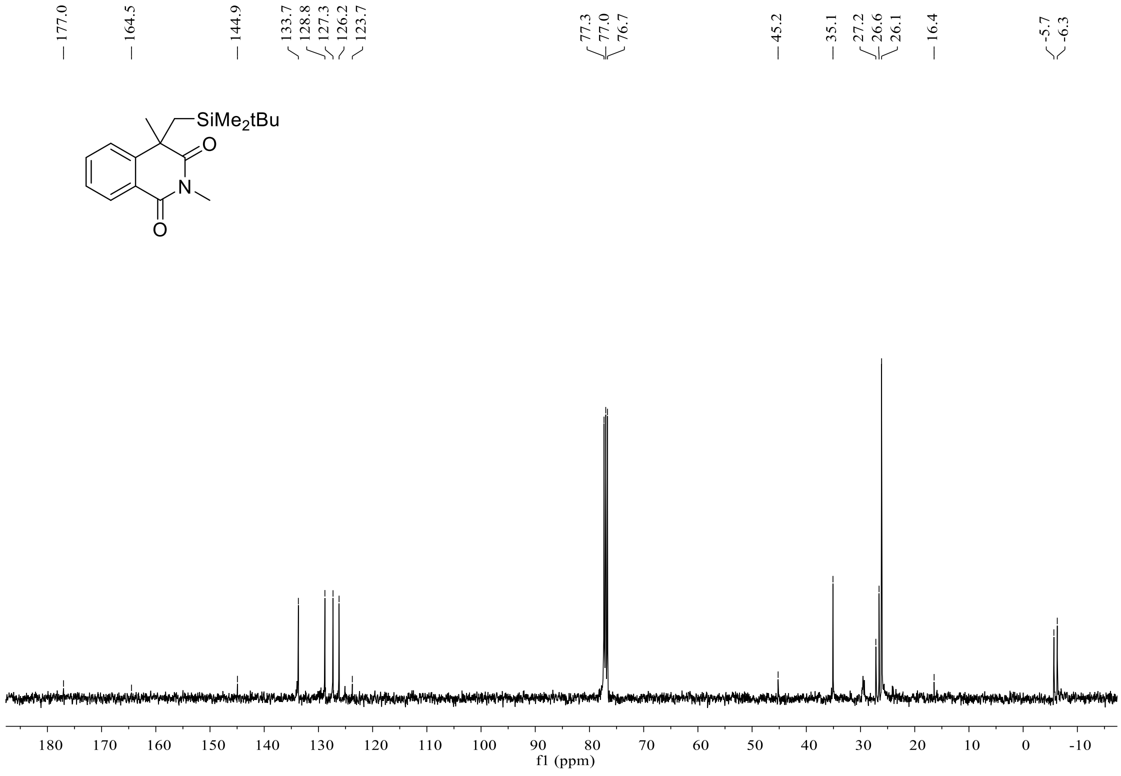

^{1}H NMR (400 MHz, CDCl$_3$) of 70

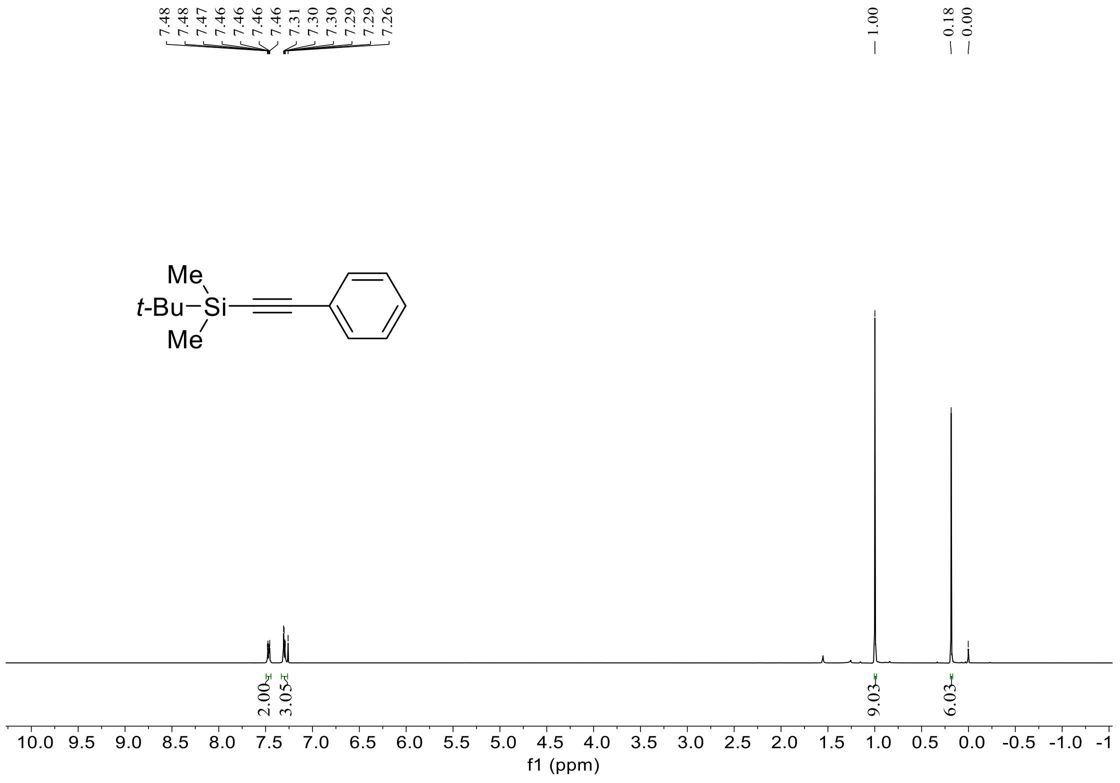

^{13}C NMR (101 MHz, CDCl$_3$) of 70

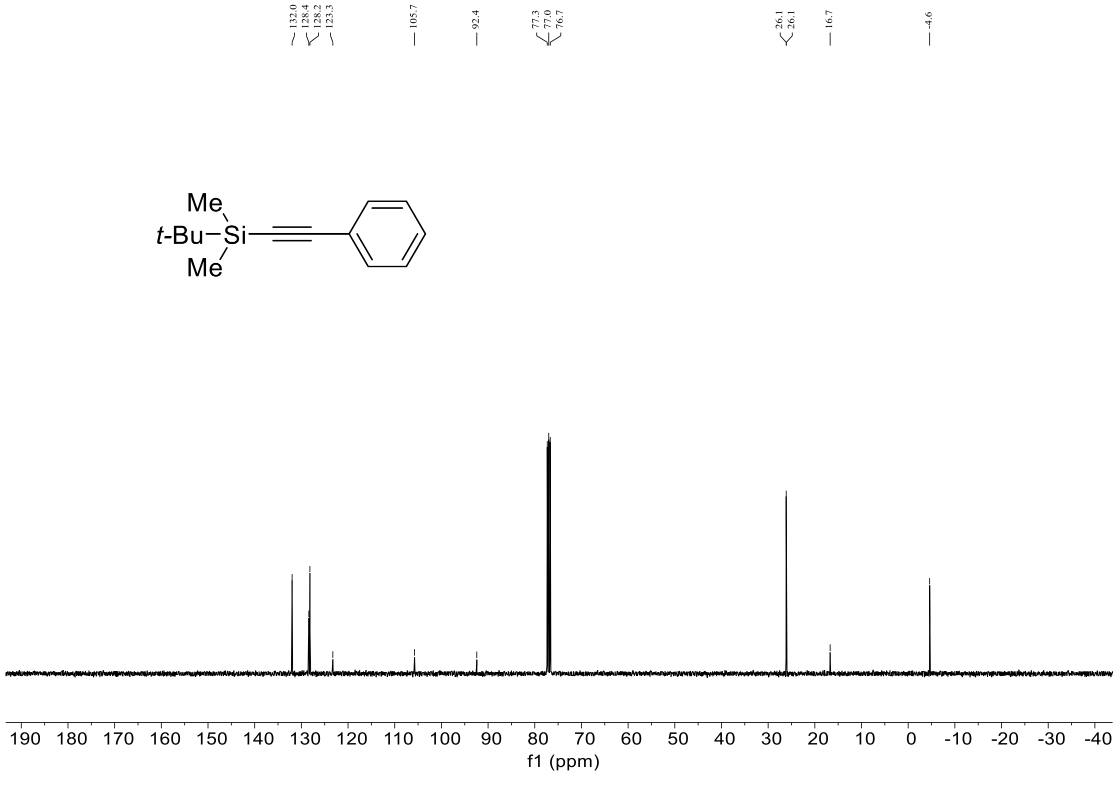

^{1}H NMR (400 MHz, $CDCl_3$) of 73

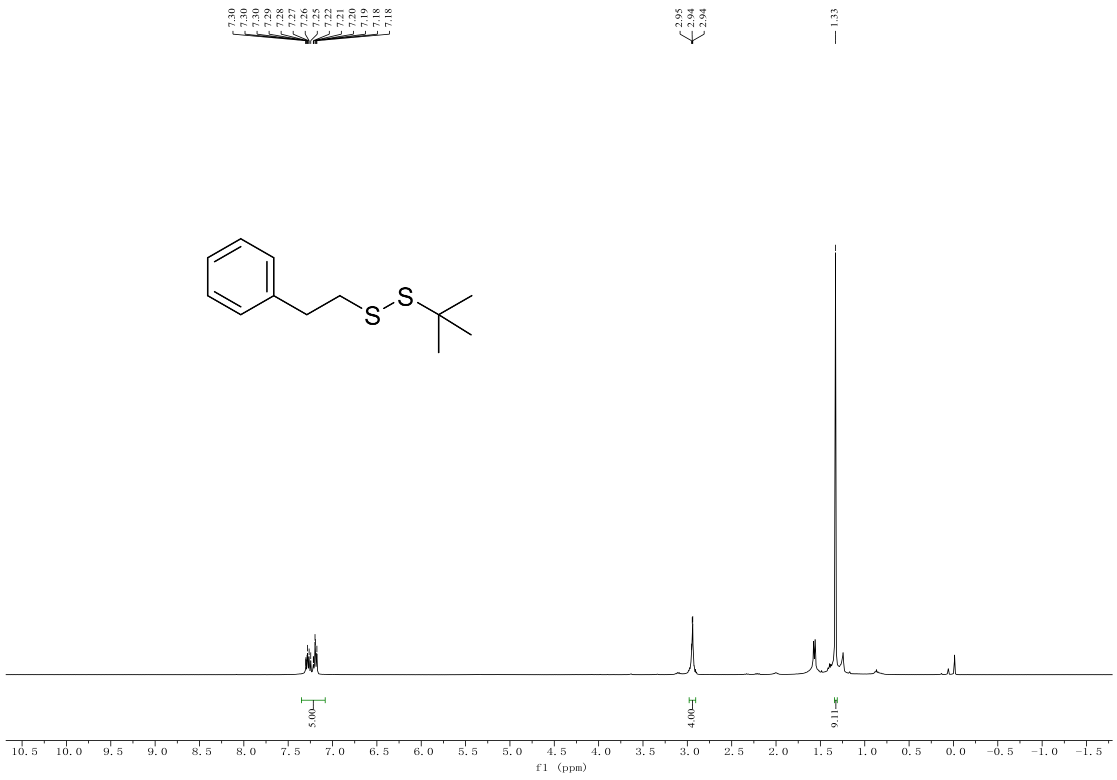

^{13}C NMR (101 MHz, $CDCl_3$) of 73

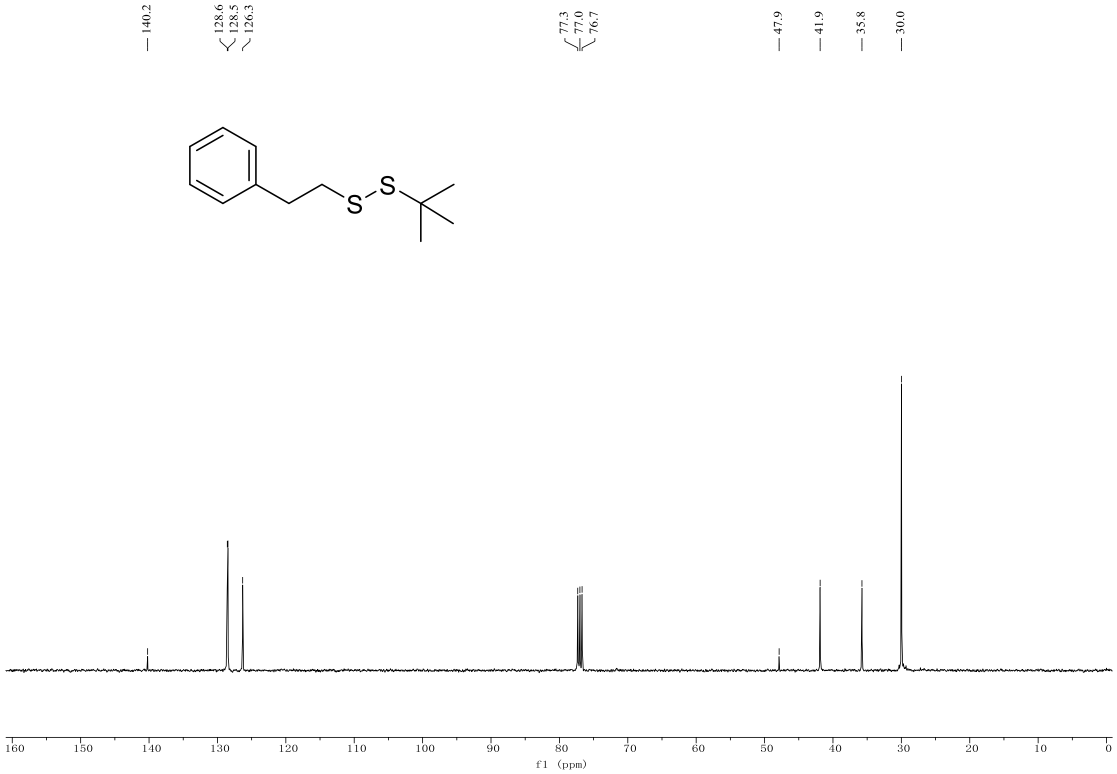

10. References

1. S.-H. Liu, P. Pan, H.-Q. Fan, H. Li, W. Wang and Y.-Q. Zhang, *Chem. Sci.* **2019**, *10*, 3817–3825.
2. H. Cai, C. Huang, C.-M. You and P. Xiao, China, CN115093440 A 2022-09-23.
3. Q.-H. Wan, Z.-W. Hou, X.-R. Zhao, X.-Y. Xie, and L. Wang, *Org. Lett.* **2023**, *25*, 1008−1013.
4. F. Rammal, D. Gao, S. Boujnah, A. A. Hussein, J. Lalevée, A.-C. Gaumont, F. M. Savary, and S. Lakhdar, *ACS Cata.* **2020**, *10*, 13710-13717.
5. Eunchan Jeong, Joon Heo, Seongho Jin, Dongwook Kim, and Sukbok Chang, *ACS Cata.* **2022**, *12*, 4898-4905.
6. W.-J. Zhang, Q. Lu, M.-S. Wang, Y.-J. Zhang, X.-F. Xia, and D.-W. Wang, *Org. Lett.* **2022**, *24*, 3797-3801.
7. C.-H. Dai, Y.-L. Zhan, P. Liu and P.-P. Sun, *Green Chem.*, **2021**, *23*, 314-319.
8. C. Jiang, Y. Liao, H. Li, S. Zhang, P. Liu, P. Sun, *Adv. Synth. Catal.* **2023**, *365*, 1205.
9. E. Davenport, D. E. Negru, G. Badman, D. M. Lindsay, W. J. Kerr, *ACS Catal.* **2023**, *13*, 11541–11547.
10. H. Jiang, J. Xie, A. Lin, Y. Cheng, and C. Zhu. *RSC Adv.* **2012**, *2*, 10496.
11. K. Wang, X. Liu, S. Yang, Y. Tian, M. Zhou, J. Zhou, X. Jia, B. Li, S. Liu, J. Chen. *Org. Lett.* **2022**, *24*, 3471.
12. X.-Y. Zhang, W.-Z. Weng, H. Liang, H. Yang, B. Zhang. *Org. Lett.* **2018**, *20*, 4686.
13. (a) J. Li, Y. Xiao, Z. Li, Y. Yan, G. Song, G. Li, J. Dong, C. Wang, T. Kang, D. Xue, *Angew. Chem. Int. Ed.* **2025**, *64*, e202511648; (b) H. Jiang, J. Xie, A. Lin, Y. Cheng, and C. Zhu, *RSC Adv.*, **2012**, *2*, 10496; (c) M. S. Santos, M. Cybularczyk-Cecotka, B. König, M. Giedyk, *Chem. Eur. J.* **2020**, *26*, 15323.
14. L. Mantry, P. Gandeepan, *J. Org. Chem.* **2025**, *90*, 6897;
15. M. J. Frisch, G. W. Trucks, H. B. Schlegel, G. E. Scuseria, M. A. Robb, J. R. Cheeseman, G. Scalmani, V. Barone, G. A. Petersson, H. Nakatsuji, X. Li, M. Caricato, A. V. Marenich, J. Bloino, B. G. Janesko, R. Gomperts, B. Mennucci, H. P. Hratchian, J. V. Ortiz, A. F. Izmaylov, J. L. Sonnenberg, Williams, F. Ding, F. Lipparini, F. Egidi, J. Goings, B. Peng, A. Petrone, T. Henderson, D. Ranasinghe, V. G. Zakrzewski, J. Gao, N. Rega, G. Zheng, W. Liang, M. Hada,

M. Ehara, K. Toyota, R. Fukuda, J. Hasegawa, M. Ishida, T. Nakajima, Y. Honda, O. Kitao, H. Nakai, T. Vreven, K. Throssell, J. A. Montgomery Jr., J. E. Peralta, F. Ogliaro, M. J. Bearpark, J. J. Heyd, E. N. Brothers, K. N. Kudin, V. N. Staroverov, T. A. Keith, R. Kobayashi, J. Normand, K. Raghavachari, A. P. Rendell, J. C. Burant, S. S. Iyengar, J. Tomasi, M. Cossi, J. M. Millam, M. Klene, C. Adamo, R. Cammi, J. W. Ochterski, R. L. Martin, K. Morokuma, O. Farkas, J. B. Foresman, D. J. Fox, Wallingford, CT, **2016**.

16. (a) B. Miehlich, A. Savin, H. Stoll, H. Preuss, *Chem. Phys. Lett.* **1989**, *157*, 200-206; (b) C. Lee, W. Yang, R. G. Parr, *Phys. Rev. B* **1988**, *37*, 785-789; (c) A. D. Becke, *J. Chem. Phys.* **1993**, *98*, 5648-5652.
17. (a) S. Grimme, S. Ehrlich, L. Goerigk, *J. Comput. Chem.* **2011**, *32*, 1456-1465; (b) S. Grimme, J. Antony, S. Ehrlich, H. Krieg, *J. Chem. Phys.* **2010**, *132*, 154104.
18. (a) F. Weigend, Phys. Chem. Chem. Phys. 2006, 8, 1057-1065; (b) F. Weigend, R. Ahlrichs, Phys. Chem. Chem. Phys. **2005**, *7*, 3297-3305.
19. (a) E. Caldeweyher, S. Ehlert, A. Hansen, H. Neugebauer, S. Spicher, C. Bannwarth, S. Grimme, *J. Chem. Phys.* **2019**, *150*, 154122; (b) E. Caldeweyher, C. Bannwarth, S. Grimme, *J. Chem. Phys.* **2017**, *147*, 034112.
20. A. V. Marenich, C. J. Cramer, D. G. Truhlar, *J. Phys. Chem. B* **2009**, *113*, 6378-6396.
